# A decennary update on applications of metal nanoparticles (MNPs) in the synthesis of nitrogen- and oxygen-containing heterocyclic scaffolds

**DOI:** 10.1039/d0ra02272a

**Published:** 2020-09-03

**Authors:** Tejas M. Dhameliya, Hiren A. Donga, Punit V. Vaghela, Bhoomi G. Panchal, Dipen K. Sureja, Kunjan B. Bodiwala, Mahesh T. Chhabria

**Affiliations:** L. M. College of Pharmacy Navrangpura Ahmedabad 380 009 Gujarat India tejas.dhameliya@lmcp.ac.in tmdhameliya@gmail.com +91 79 2630 4865 +91 79 2630 2746

## Abstract

Heterocycles have been found to be of much importance as several nitrogen- and oxygen-containing heterocycle compounds exist amongst the various USFDA-approved drugs. Because of the advancement of nanotechnology, nanocatalysis has found abundant applications in the synthesis of heterocyclic compounds. Numerous nanoparticles (NPs) have been utilized for several organic transformations, which led us to make dedicated efforts for the complete coverage of applications of metal nanoparticles (MNPs) in the synthesis of heterocyclic scaffolds reported from 2010 to 2019. Our emphasize during the coverage of catalyzed reactions of the various MNPs such as Ag, Au, Co, Cu, Fe, Ni, Pd, Pt, Rh, Ru, Si, Ti, and Zn has not only been on nanoparticles catalyzed synthetic transformations for the synthesis of heterocyclic scaffolds, but also provide an inherent framework for the reader to select a suitable catalytic system of interest for the synthesis of desired heterocyclic scaffold.

## Abstract 2

Metal nanoparticle (MNP)-catalyzed synthesis of nitrogen- and oxygen-containing heterocyclic scaffolds.

## Graphical abstract

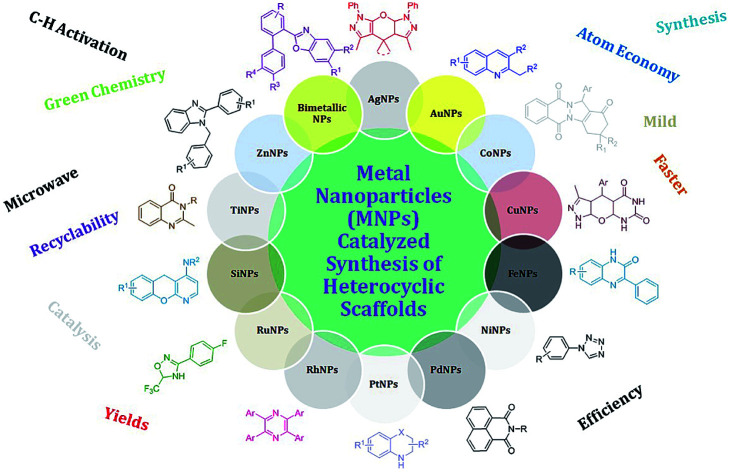

## 1 Introduction

**Fig. 1:**
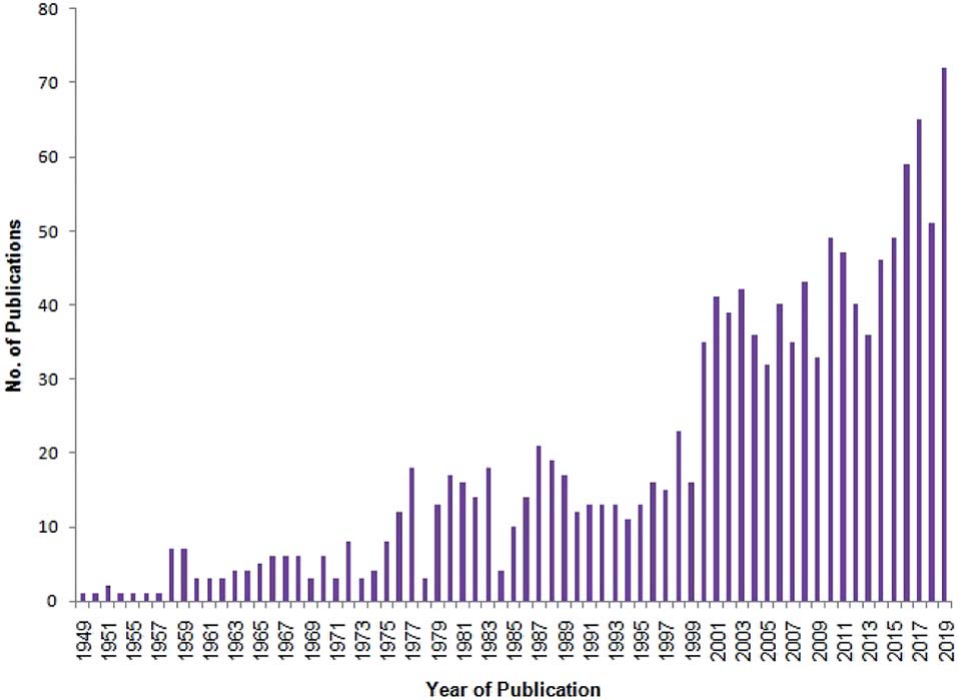
Number of publications *versus* corresponding year of publication for the search string ‘synthesis of heterocyclic scaffolds’ accessed by Sci-Finder on Nov 28, 2019.

Heterocycles containing nitrogen^1^ and oxygen^2^ have been found to be of much importance among the United States Food and Drug Administration (USFDA) approved drugs. Specifically, 59% of the USFDA-approved drugs contain a nitrogen-containing heterocycle,^1^ and the USFDA has approved 311 drug candidates assembled with an oxygen-containing heterocycle.^2^ Numerous heterocyclic scaffolds have been synthesized with modern advancements with an endeavor towards drug discovery paradigm.^3^ A recent analysis on structures of USFDA-approved drugs administered in combination with other drugs as reported by Njardarson *et al.* revealed that the major importance of these heterocycles in combination drugs is in variety of therapeutic uses.^4^ The rich literature^5–14^ on the synthesis of these USFDA-approved drugs is also gaining momentum since for the clinical use of a drug, the drug molecule has be synthesized on a pilot scale using a suitable toolbox of organic chemistry.^15^ A plot of the number of publications *versus* year of publication performed using Sci-Finder^16^ for the search string ‘synthesis of heterocyclic scaffolds’ revealed the increasing popularity of heterocyclic scaffolds synthesized *via* several organic transformations (Fig. 1).

**Fig. 2:**
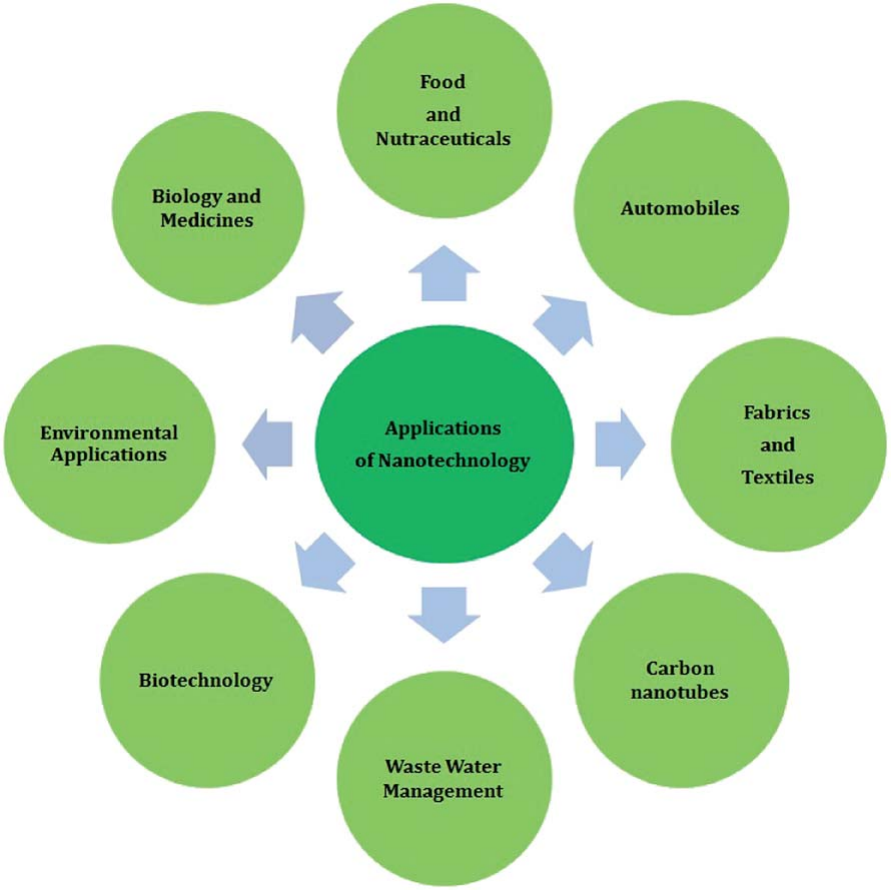
Recent applications of nanotechnology.

Nanomaterials are defined as materials with at least one of their dimensions in the nanometer range (1 nm to 100 nm). Recently, nanotechnology has advanced significantly, and thus has found substantial applications in therapeutics,^17^ food and nutraceuticals,^18^ wastewater management,^19^ biotechnology,^20^ biology and medicine,^21^ automobiles,^22^ fabrics and textiles,^23^ carbon nanotubes,^24^ and environmental applications^25^ (Fig. 2).

**Fig. 3:**
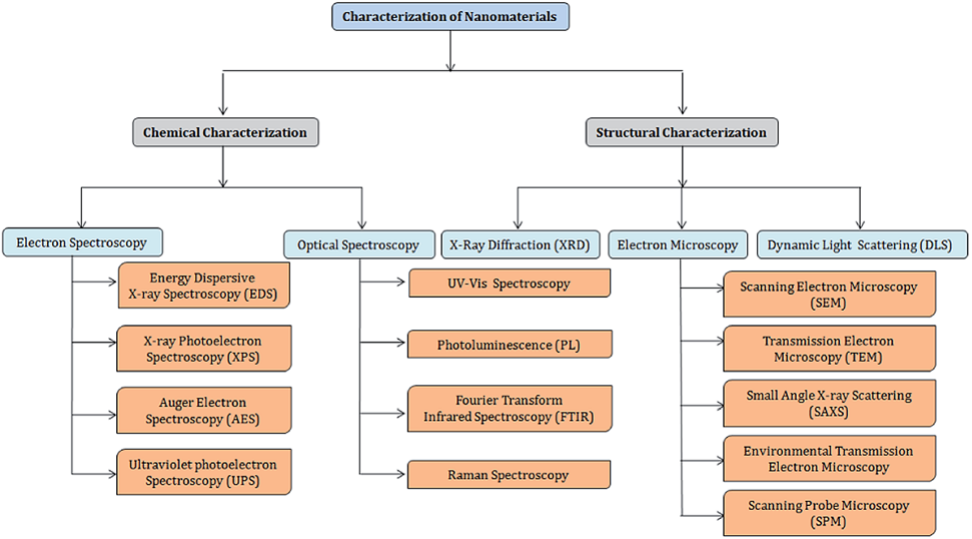
Basic techniques for the characterization of nano-materials. Chemical characterization includes optical spectroscopy such as optical absorption spectroscopy, which includes UV-Vis spectroscopy, photoluminescence (PL), Fourier transform infrared spectroscopy (FTIR), and Raman spectroscopy; and electron spectroscopy including energy dispersive X-ray spectroscopy (EDS), X-ray photoelectron spectroscopy (XPS), auger electron spectroscopy (AES), and ultraviolet photoelectron spectroscopy (UPS). Structural characterization involves X-ray diffraction (XRD); electron microscopic techniques such as scanning electron microscopy (SEM), transmission electron microscopy (TEM), small angle X-ray scattering (SAXS), environmental transmission electron microscopy (ETEM), and scanning probe microscopy (SPM); and dynamic light scattering (DLS) using a particle size analyzer.

A wide variety of techniques is available for the characterization of nanomaterials due to the advancement of sophisticated and simple spectrophotometric and robotic instrumentation. The basic techniques required for the characterization of nanomaterials, namely chemical and structural characterization, are presented in Fig. 3. The size of nanoparticles can be characterized using transmission electron microscopy (TEM), X-ray diffraction (XRD), dynamic light scattering (DLS), high-resolution transmission electron microscopy (HR-TEM), scanning electron microscopy (SEM), atomic force microscopy (AFM), inductively coupled plasma-mass spectrometry (ICP-MS), UV-visible spectroscopy (UV-Vis), matrix-associated laser desorption ionization (MALDI), and nuclear magnetic resonance (NMR). The shape of NPs can be characterized using TEM, HR-TEM, and AFM, while XRD, X-ray photoelectron spectroscopy (XPS), inductively coupled plasma-mass spectrometry (ICP-MS), and NMR can be used to confirm their chemical composition. The surface properties of NPs can be determined using the BET (Brunauer, Emmett and Teller) technique and liquid NMR, and their magnetic behavior can be tested using vibrating sample magnetometry (VSM) and Mössbauer spectroscopy.^26^

## 2 Nanocatalysis

Catalysis (Greek: to dissolve) is the terminology coined by Swedish chemist Jöns Jacob Berzelius in 1836, which means a substance awakens dormant affinities by its mere presence.^27^ Catalysis can be categorised into homogenous catalysis and heterogeneous catalysis. Heterogeneous catalysis is more favoured over homogenous catalysis since the former is beneficial for the easier isolation, purification and recycling of costly catalysts for subsequent transformations. Most nanoparticles (NPs) are heterogeneous catalysts, boosting the goals of synthetic chemist to co-align green chemistry approaches.^28^

Nanocatalysts have a particle size in the nm scale, and thus have a large surface area, which enables the interaction of chemical reactants *via* cooperative activation to bring them in closer proximity with each other. Nanocatalysis has been recently employed for wide applications in nanotechnology, productions of fuels from biomass, wastewater treatment, bioelectrocatalysis, environmental applications,^29^ sustainable applications in green synthesis,^30^ and organic transformations.^31^ Grönbeck *et al.* recently summarized the mechanistic insight of nanocatalysis *via* computational methods, including density functional theory (DFT) and other computation tools.^32^

The synthesis of nanocatalysts (NCs) is generally achieved using metals and transition metals from their congener inorganic salts, which are treated with solid supports or linkers to generate reactive functional groups on their extreme periphery. The catalytic activity of NPs is generally achieved using doping agents *via* their incorporation into metals and metallic oxides, Lewis acids and bases^33^ and ionic liquids.^34^

## 3 Scope and applications of NPs in the synthesis of heterocycles

**Fig. 4:**
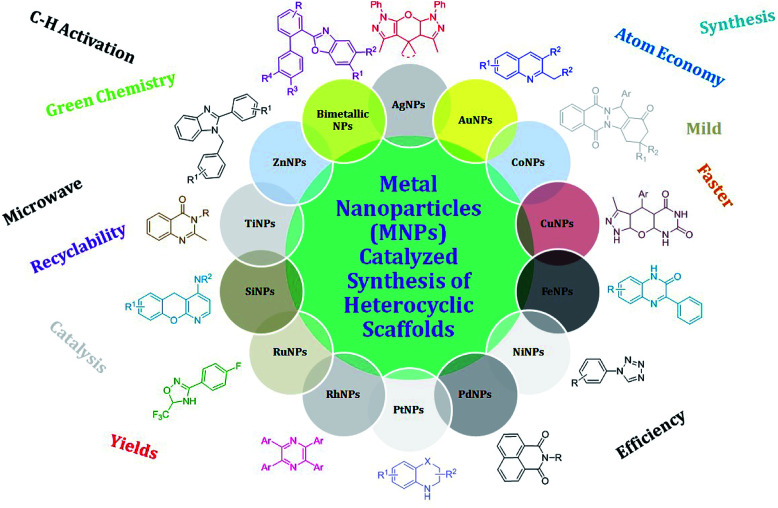
Metal nanoparticle (MNP)-catalyzed synthesis of nitrogen- and oxygen-containing heterocyclic scaffolds.

According to green chemistry, MNP-catalyzed organic transformations are the safest reactions, which do not affect the environment. Some of the important applications of MNPs and metal oxide NPs have been also demonstrated for different types of C–H activation, such as C(sp^3^)–H and C(sp^2^)–H functionalization,^35,36^ asymmetric C–C bond formation,^37^ biomedical applications,^38^ and various organic transformations.^39^ Shaaban *et al.* reviewed the applications of NPs with respect to heterocycles and fused heterocycles.^40^ Recently, Gómez *et al.* summarized the use of PdNPs for the synthesis of polyols *via* catalytic coupling and hydrogenation.^41^ Dandia *et al.* recently reported the synthesis of three-, five-, six- and seven-membered ring systems using nanocatalysts.^42^ Chaudret *et al.* recently covered the role of several MNPs in σ-H–H, σ-C–H, and σ-Si–H bond activation.^43^ Although there are numerous applications of NPs for a variety of organic transformations, in the present review we aimed to focus on the complete coverage of the applications of MNPs (metal nanoparticles) in the synthesis of heterocyclic scaffolds (Fig. 4) reported during the last ten years (2010 to the end of 2019). Thus, it is expected that this review article will give opportunistic insight to organic chemists to select suitable MNPs to achieve the synthetic transformation of choice in order to generate novel molecules, consequently enriching the synthetic armory of the toolbox of medicinal chemists to construct desired scaffolds.^44^

### 3.1 AgNP-catalyzed synthesis of heterocycles

**Scheme 1:**
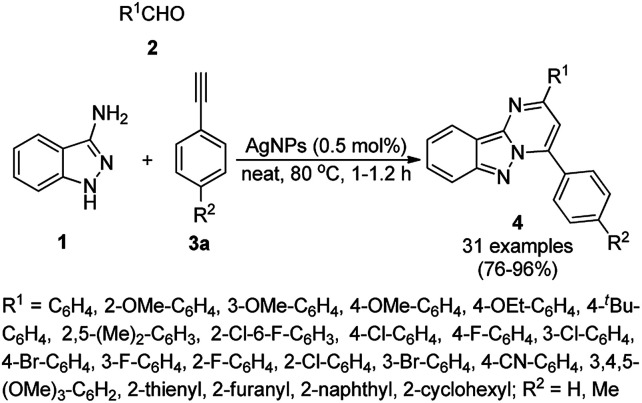
Synthesis of pyrimido[1,2-*b*]indazole derivatives (4) using AgNPs under solvent-free conditions.

AgNPs have been recognized to be important in the synthesis of heterocyclic scaffolds.^45,46^ Balwea *et al.* synthesized pyrimido[1,2-*b*]indazole derivatives (4) using silver nanoparticles (AgNPs) obtained from the plant extract of *Radix puerariae*. The extract of *Radix puerariae* powder was treated with AgNO_3_ in basic medium and the synthesis of AgNPs was monitored using UV-Vis spectroscopy. The synthesized AgNPs were well characterized *via* TEM, EDX, XRD, dynamic light scattering (DLS) and zeta potential measurements. The synthesis of 4 was achieved using one pot A^3^ coupling involving the reaction of three components, *i.e.* 3-aminoindazoles (1), aryl/heteroaryl/alicyclic aldehyde (2), and substituted phenyl acetylene (3a), using AgNPs (0.5 mol%), solvent-free conditions at 80 °C for 1 h (Scheme 1).^47^ They also demonstrated the successful use of the AgNP-catalyzed A^3^ coupling on the gram scale in 90% yield. The catalysts were recovered after the reaction, washed with triple-distilled water, activated and used for up to three consecutive runs.

**Scheme 2:**
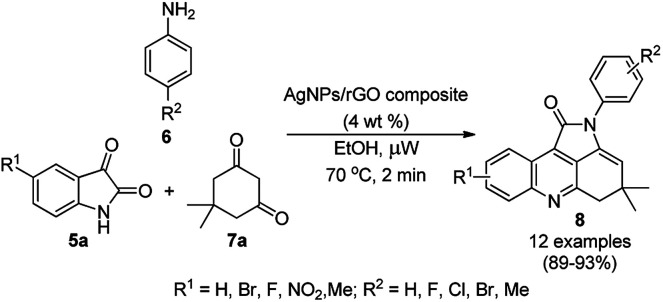
AgNP-catalyzed synthesis of pyrrolo[2,3,4-*kl*]acridin-1-ones (8).

Graphene-supported MNPs have been recently gaining tremendous momentum in the development of C–C and C–X coupling reactions.^48^ In this endevour, Dandia *et al.* reported the synthesis of pyrrolo[2,3,4-*kl*]acridin-1-ones (8, Scheme 2) using AgNP-decorated reduced graphene oxide (AgNPs/rGO).^49^ A method free from high temperature, pressure and toxic chemicals was used for the synthesis of the nanoparticles *via* the simultaneous reduction of graphene oxide (GO) and preparation of AgNPs on GO. The structural characteristics of the nanoparticles were confirmed using TEM, XRD, SEM, XPS, EDX, UV-Vis spectroscopy, cyclic voltammetry, and Raman and FT-IR spectroscopy. The application of these AgNPs was demonstrated for the synthesis of 8 from a three-component reaction involving substituted isatins (5a), anilines (6) and dimedone (7a). The effects of various conditions such as catalyst and solvent were studied under microwave irradiation at 70 °C. Subsequently, employing the optimized conditions, the authors reported the rapid synthesis of 8 in 89–93% yield within 2 min. They reported that ethanol was the best solvent because of the relatively higher dispersion of the catalyst and reactants. Furthermore, the recovered catalyst was successfully used for seven consecutive catalytic cycles without significant loss in the yield of 8.

**Scheme 3:**
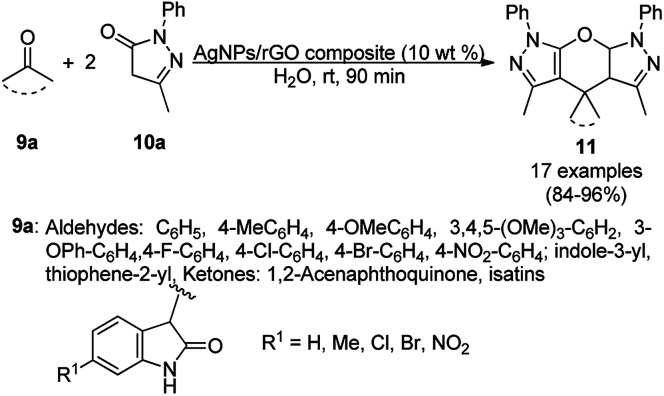
Chemoselective “on-water” synthesis of pyrano[2,3-c:6,5-c′]dipyrazol]-2-ones (11) catalyzed by AgNPs on GO composite.

Further green applications of an AgNP-decorated GO (graphene oxide) composite as a catalyst “on water” were demonstrated by Dandia *et al.* for the synthesis of pyrano[2,3-*c*:6,5-*c*′]dipyrazol-2-ones (11) at rt in excellent yields (Scheme 3).^50^ The catalytic potential of AgNPs was proposed by the authors due to their role as a Lewis acid catalyst, which enabled Knoevenagel condensation–Michael addition and cyclization. The SEM and TEM images of the recovered catalysts revealed the integrity of the catalyst, and thus no significant loss of catalytic activity was observed for up to seven catalytic cycles.

**Scheme 4:**
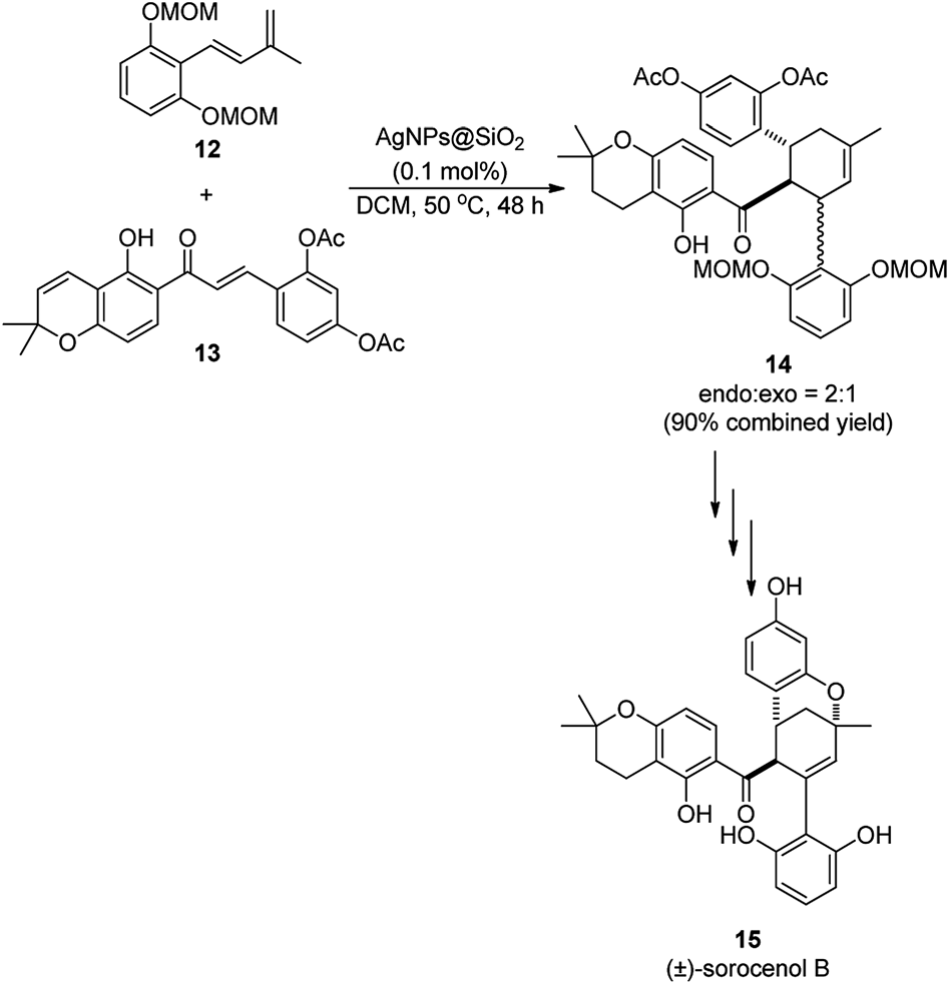
AgNP-catalyzed synthesis of cycloadduct (14) for the total synthesis of natural product (15).

Porco *et al.* reported the use of AgNP-catalyzed Diels–Alder [4 + 2] cycloaddition (Scheme 4) for the successful synthesis of cycloadduct (14) from chalcone (13) and diene (12), which led to the formation of *endo* and *exo* diastereoisomers in a 2 : 1 ratio.^51^ Subsequently, 14 was used in the total synthesis of (±)-sorocenol B (15), an anti-cancer natural product, through several synthetic steps. The required AgNPs were synthesized following their previously reported protocol^52^*via* the reduction of silver tetrafluroborate (AgBF_4_) using tetrabutyl ammonium borohydride (Bu_4_NBH_4_) with silica gel in dichloromethane (DCM).

**Scheme 5:**
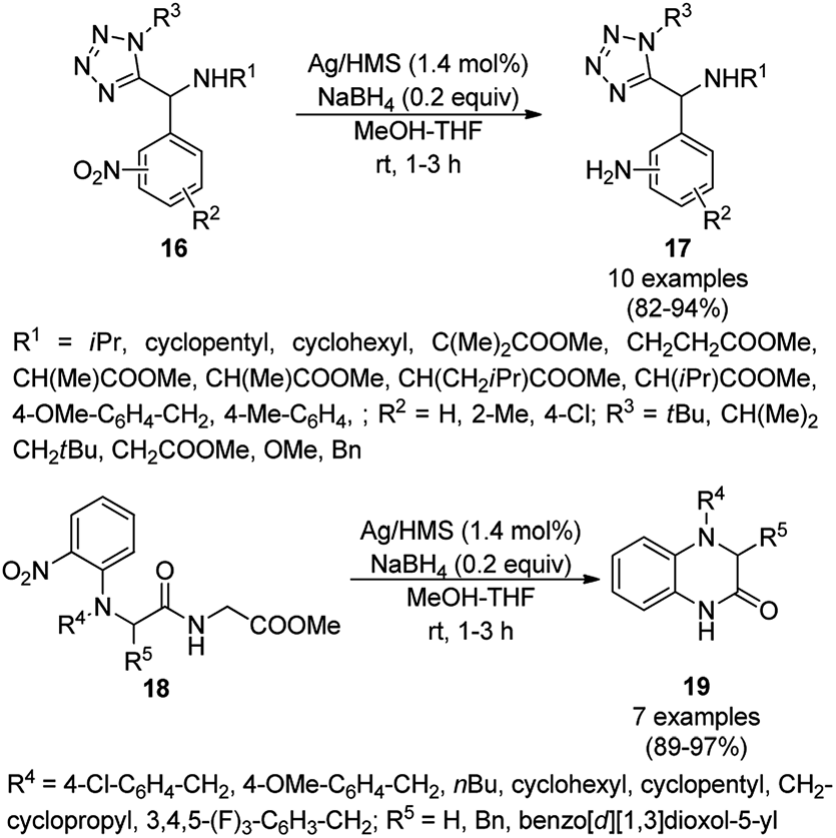
AgNP-catalyzed chemoselective reduction of nitroarenes (16) and synthesis of 19.

The AgNP-supported mesoporous silica (Ag/HMS)-catalyzed chemoselective reduction of tetrazoles containing nitroarenes (16) was reported by Lykakis *et al.* (Scheme 5) at a low catalytic loading with high functional group tolerance.^53^ When the same protocol was extended to the Ugi-Smiles product (18), it resulted in cyclisation with the synthesis of 19*via in situ* reduction–cyclization. The dihydroquinoxalnones (19) were claimed to inhibit soluble epoxide hydrolase, having anti-hypertensive and anti-inflammatory activity.

**Scheme 6:**
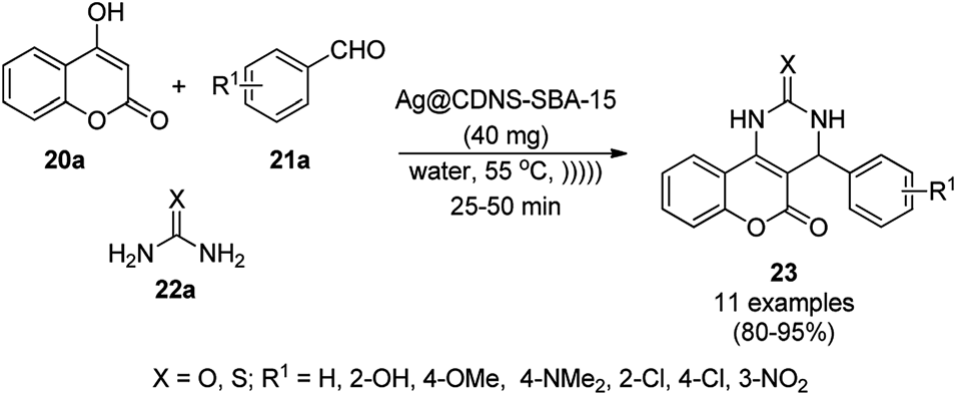
Synthesis of benzopyranopyrimidines (23) reported by Heravi *et al.*

Heravi *et al.* recently reported the catalytic use of bio-assisted AgNPs supported on an SBA-15/cyclodextrin nanosponge adduct (Scheme 6) for the synthesis of benzopyranopyrimidines (23) from 4-hydroxycoumarins (20a), substituted benzaldehydes (21a), and urea or thiourea (22a) under ultrasonication.^54^ The cyclodextrin sponge played the key role by bringing the reagents in closer proximity of the AgNPs for the catalysis. The catalyst was reused and recycled for up to four times without losing its catalytic activity.

**Scheme 7:**
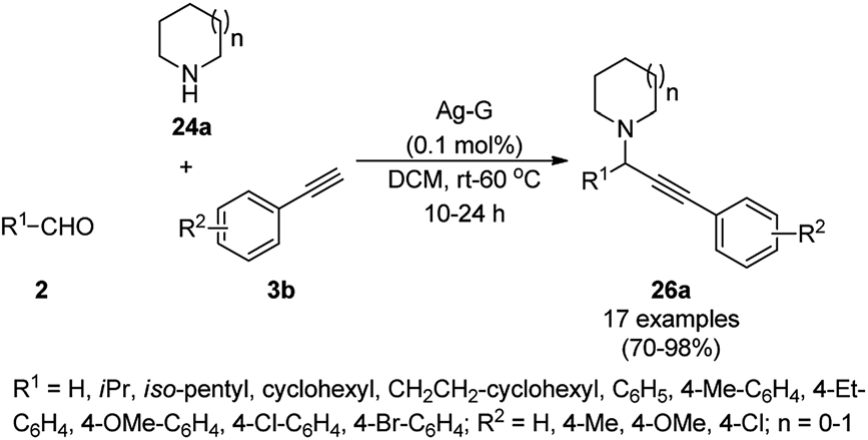
Silver–graphene nanocomposite-catalyzed synthesis of propargyl amines (26a).

**Scheme 8:**
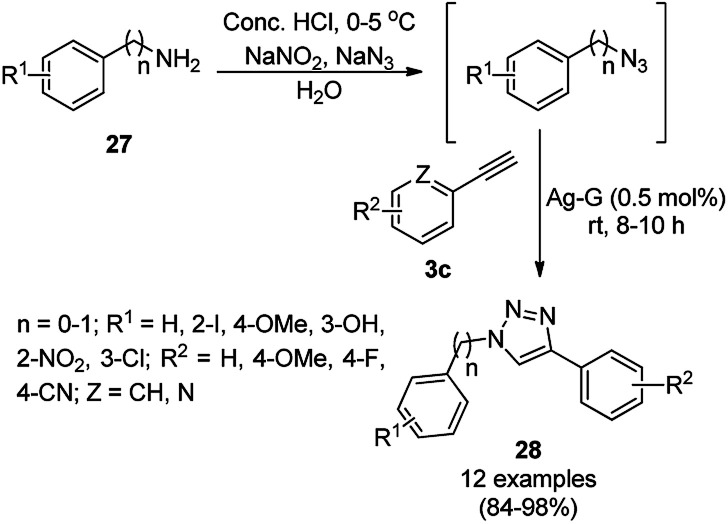
Click reaction catalyzed by silver–graphene nanocomposites.

Jana *et al.* synthesized silver–graphene nanocomposites from graphene oxide (GO), silica-coated AgNPs previously synthesized from [3-(2-aminoethylamino)propyl]trimethoxysilane (AEAPS), (3-mercaptopropyl)-trimethoxysilane (MPS) and silver acetate. The silver-graphene nanocomposite-catalyzed A^3^ coupling of aldehydes (2), alicyclic amines (24a) and alkynes (3b) for the synthesis of propargyl amines (26a) was achieved successfully in dichloromethane (Scheme 7).^55^ A similar catalyst was also found to catalyze the synthesis of 1,2,3-triazoles *via* the click reaction of *in situ* generated azides from diazotization (Scheme 8) of anilines or arylalkyl amines (27), followed by cycloaddition with terminal alkynes (3c). The same catalyst was recycled for the synthesis of 26a and 28 for up to five consecutive runs without loss in its catalytic activity.

**Scheme 9:**
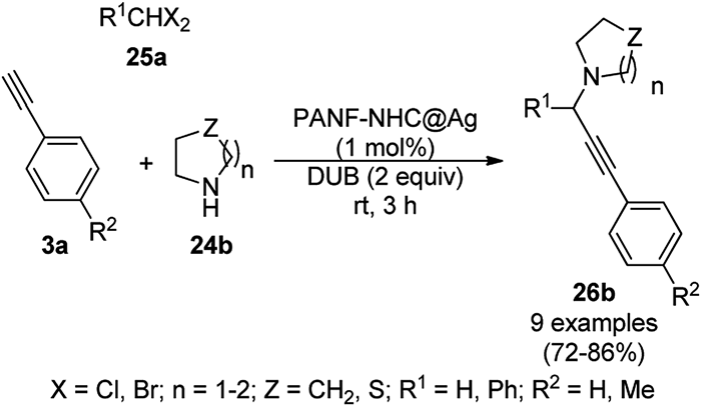
AHA coupling catalyzed by NHC-protected AgNPs.

The N-heterocyclic carbine (NHC)-protected AgNPs supported on polyacrylonitrile fiber (PANF-NHC@Ag)-catalyzed three-component coupling of amines (24b), halomethanes (25a), and alkynes (3a) (AHA coupling, Scheme 9) was successfully carried out by Tian *et al.*^56^ The catalyst was prepared *via* the amination of PANF with ethylenediamine followed by its complexation with previously prepared [*N*-benzyl-*N*′-(methoxycarbonyl methyl)imidazolin-2-ylidene]silver chloride and reduction with sodium borohydride to obtain active NPs. These AgNPs were recycled for up to ten cycles without appreciable loss in their catalytic potential. The same AgNP-catalyzed reaction was also checked for its applicability in flow chemistry. Compared to reported protocols for AHA coupling, the PANF-NHC@Ag-catalyzed protocol was claimed to be superior since it is operational at rt in shorter times with high reusability under solvent-free conditions in comparison with AuNPs,^57^ CuCl,^58,59^ CuI,^60^ nano In_2_O_3_,^61^ FeCl_3_,^62^ CoBr_2_,^63^ AgOAc^64^ and Au/CeO_2_.^65^

### 3.2 AuNP-catalyzed synthesis of heterocycles

**Scheme 10:**
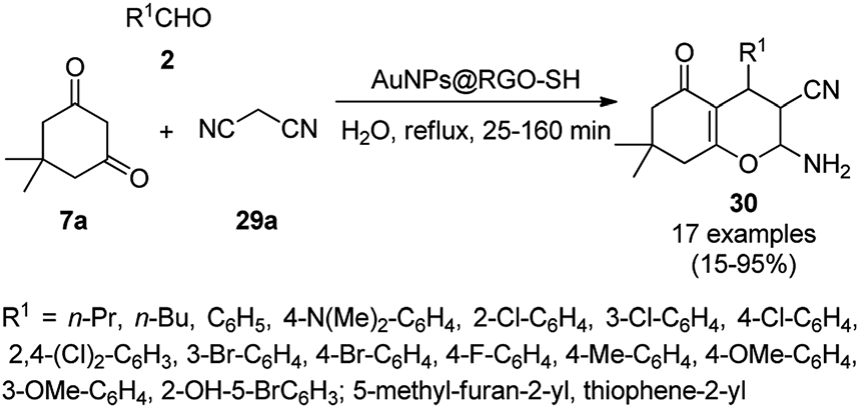
Synthesis of tetrahydro-4*H*-chromenes (30) catalyzed by AuNPs@RGO-SH.^70^

Tetrahydro-4*H*-chromenes show a broad spectrum of biological activity such as K^+^ channel activator,^66^ antimicrobial,^67^ insulin sensitizer,^68^ and anticancer activities.^69^ The green and effective synthesis of tetrahydro-4*H*-chromenes was reported by Naeimi *et al.* using gold NPs supported on thiol-functionalized reduced graphene oxide (AuNPs@RGO-SH) from substituted benzaldehyde (2), 7a and malononitrile (29a) in aqueous medium under reflux (Scheme 10).^70^ The self-developed catalysts were characterized using atomic force microscopy (AFM), field emission scanning electron microscopy (FE-SEM), FT-IR spectroscopy, thermal gravimetric analysis (TGA), and XRD. The catalytic potential of the catalysts was retained even after the sixth catalytic run, and it was found to yield 90% of 2-amino-4-(4-chlorophenyl)-7,7-dimethyl-5-oxo-3,4,5,6,7,8-hexahydro-2*H*-chromene-3-carbonitrile from the model substrates such as 4-chlorobenzaldehydes 7a and 29a after five catalytic reuses.

**Scheme 11:**
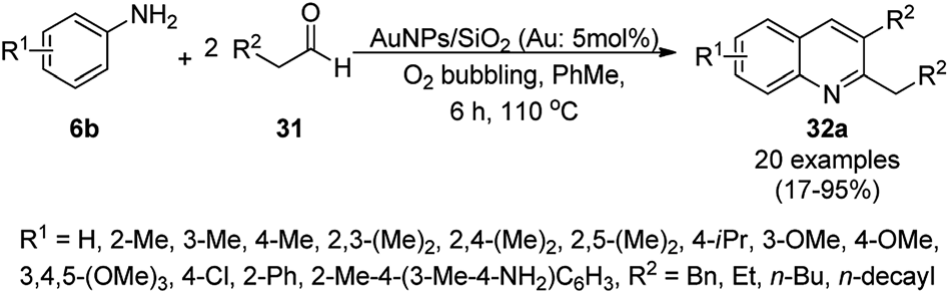
Aerobic oxidative cyclocondensation of arylamines (6b) with arylalkyl aldehydes (31) in the presence of silica-supported AuNPs.

The synthesis of polysubstituted quinolines (32a) was reported by Che *et al. via* the cyclisation of substituted anilines (6b) with arylalkyl/alkyl aldehydes (31) using SiO_2_-supported AuNPs under an oxygenated environment (Scheme 11).^71^ The synthesized AuNPs/SiO_2_ were characterized *via* XRD, XPS, TEM, selected area electron diffraction (SAED) analysis, EDX, and ICP-MS. The same protocol using AuNPs/SiO_2_ (5 mol%) as the catalyst and toluene as the solvent at 110 °C for 6 h under bubbling O_2_ was further extrapolated for the synthesis of nitrogen-containing polyheterocyclic compounds from aryl or heteroaryl amine and 3-phenyl propanal, resulting in 17–95% yield. In the mechanistic pathway, the AuNPs/SiO_2_ played the key role as Lewis acid catalysts and the AuNPs together with O_2_ enabled the oxidative conversion of 1,2-dihydroquinoline to quinolines.

**Scheme 12:**
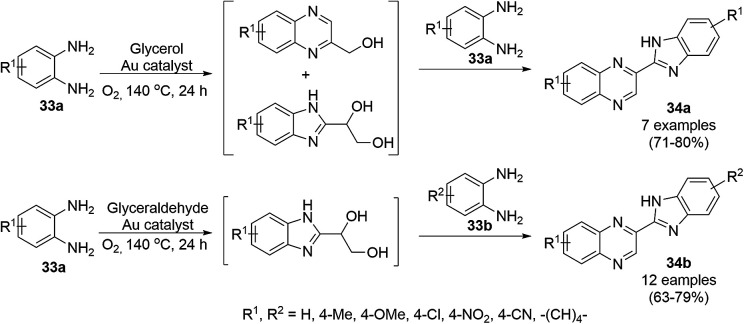
AuNP-catalyzed one-pot synthesis of benzimidazoylquinoxalines (34a/b) from *o*-phenylene diamine (33) and glycerol/glyceraldehyde.

Climent *et al.* reported the one-pot synthesis of benzimidazoylquinoxalines (34a/b) from *o*-phenylene diamine (33a/b) and glycerol/glyceraldehyde using gold NPs immobilized on nanoparticulate CeO_2_ (Au/CeO_2_) in a catalytic amount in the presence of air (Scheme 12).^72^ AuCl_4_·3H_2_O was treated with NaOH, a colloidal solution of CeO_2_ and water until the complete removal of chloride, which was confirmed by the AgNO_3_ test. Further, it has been reduced by 1-phenylethanol at 160 °C for 2 h. Finally, the nanoparticulate size of the synthesized NPs was confirmed by high-angle annular dark-field scanning transmission electron microscopy (HAADF-STEM). These NPs were successfully recycled for the synthesis of 34a/b*via* the oxidative coupling of glycerol with *o*-diaminobenzene with a slight loss in catalytic potential; however, the gold content in the recycled catalyst remained the same, which was confirmed by X-ray fluorescence.

**Scheme 13:**
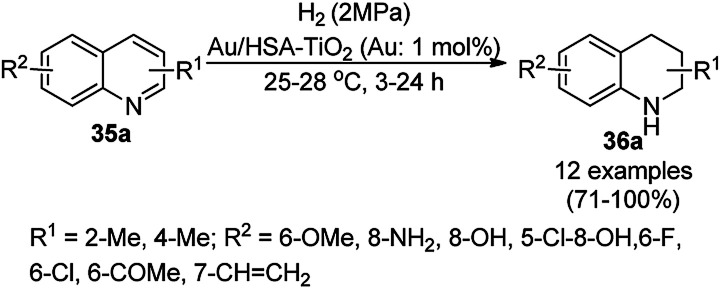
Chemoselective hydrogenation of quinolines (35a) catalyzed by Au NPs.

The chemoselective and regiospecific reduction of substituted quinolines (35a) was reported by Ren *et al.* using AuNPs supported on high surface area TiO_2_ (Au/HAS-TiO_2_) at 25–28 °C (Scheme 13).^73^ The prepared NPs were characterized *via* X-ray photoelectron spectroscopy (XPS), diffuse reflectance infrared Fourier transform spectroscopy (DRIFTS) and X-ray absorption near-edge structure (XANES). The same protocol was also found to be successful for the hydrogenation of nitrogen-containing heterocycles such as substituted quinolines, isoquinolines and other biologically significant heterocyclic compounds in excellent yields, as determined by GC. These self-developed reaction conditions were claimed by the authors to selectively reduce halogens, ketones, and olefins. The optimized protocol was reported to give a yield of 100% for the hydrogenation of 100 mmol of quinoline on a large scale.

**Scheme 14:**
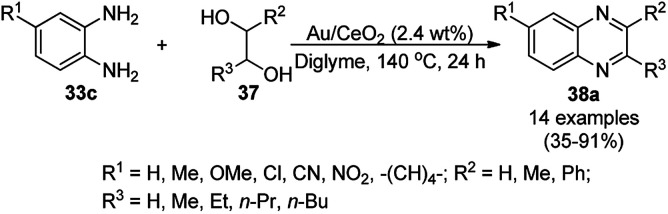
AuNP-catalyzed synthesis of quinoxalines (38a) from substituted *o*-phenylene diamine (33c) and glycols (37).

Iborra *et al.* reported the synthesis of quinoxalines (38a) from *o*-phenylene diamine (33c) and biomass-derived substituted glycols or vicinal diols (37) using AuNPs supported on CeO_2_ as the catalyst and diglyme as the solvent at 140 °C under base-free conditions (Scheme 14).^74^ The particle size of the synthesized NPs (3.5 nm) was revealed using high-angle annular dark-field scanning transmission electron microscopy (HAADF-STEM). The reusability of the catalyst was studied for up to four catalytic cycles; however, the catalytic activity was found to be reduced after the fourth cycle, as confirmed using % conversion to final compound.

**Scheme 15:**
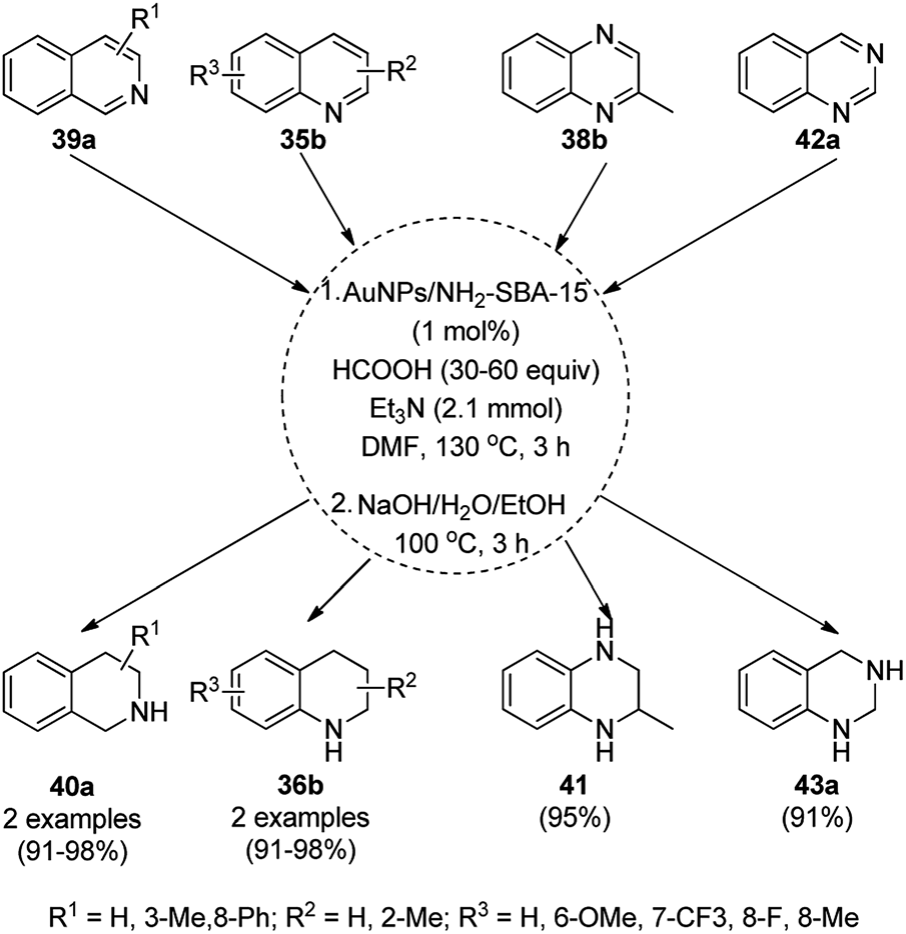
Hydrogenation of N-heterocyclic compounds (39a/35b/38b/42a) catalyzed by AuNPs supported on amino-functionalized sili*ca*.

The hydrogenation of N-heterocyclic compounds such as isoquinolines (39a), quinolines (35b), quinoxalines (38b), and quinazoline (42a) was proven to be successful using AuNPs supported on 3-aminopropyl-functionalized silica (AuNPs/NH_2_-SBA-15) as the catalyst in anhydrous DMF in formic acid (Scheme 15).^75^ The scope of the reaction was also extended for the reduction of acridine, 1,10-phenanthroline, phenanthridine and benzo[*h*]quinoline. The authors also extended it for the reductive formylation of the above heterocycles using an excess amount of formic acid and deuteration of 2-methyl quinolines using deuterated formic acid and DMF-*d*_7_. The authors speculated and proved that the reaction proceeded *via* protonation using formic acid, which underwent 1,2-addition followed by disproportionation to yield the hydrogenated product.

**Scheme 16:**
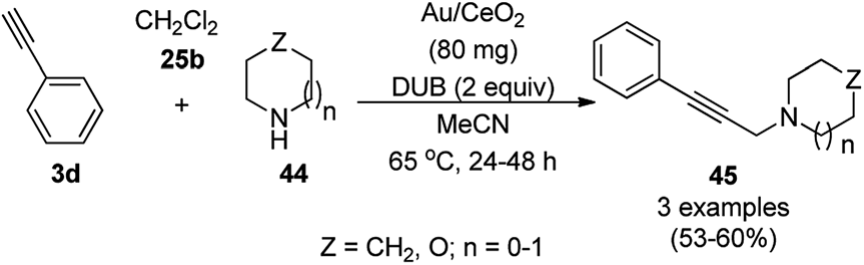
AHA coupling catalyzed by AuNPs for the synthesis of propargylamines (45).

AuNP-supported ceria-catalyzed AHA coupling was reported for the synthesis of propargylamines (45) *via* the three-component coupling reaction of phenyl acetylene (3d), dichloromethane (25b), and alicyclic amines (44, Scheme 16).^65^ However, a limited number of examples was screened for AHA coupling, where the catalyst was recycled only three times with a poor-moderate yield of 30–53%. Herein, from a mechanistic point of view, DCM reacts with amine to form a Mannich base, which *via* the elimination of HCl, forms chloromethanamine. The latter reacts with phenylacetylene adsorbed on the surface of the catalyst to give propargylamines. As evident from the literature reports, the mechanism for AuNP-catalyzed AHA coupling is completely different from than that of AgNPs (Scheme 9),^56^ where DCM first reacts with amine to form an iminium ion, which then interacts with the metal-phenyl acetylide complex to form propargylamines.

**Scheme 17:**
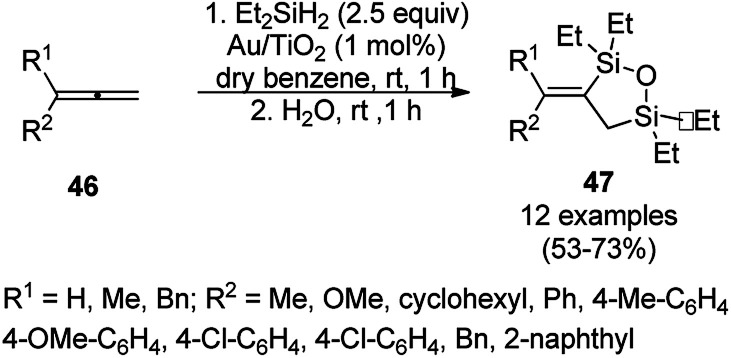
Synthesis of 3-alkylidene-1,2,5-oxadisilolanes (47) reported by Stratakis *et al.*

**Scheme 18:**
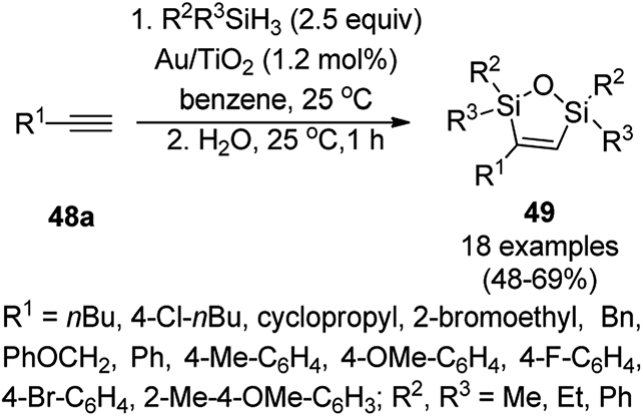
Au/TiO_2_-catalyzed synthesis of 2,5-dihydro-1,2,5-oxadisiloles (49) from alkynes (48a) and dihydrosilanes.

The AuNP-supported TiO_2_ (Au/TiO_2_)-catalyzed regioselective dehydrogenative 1,2-desilylation through the one-pot synthesis of novel 3-alkylidene 1,2,5-oxadisilolanes (47) from allenes (46) and diethyl dihydrosilane was reported (Scheme 17).^76^ Further, Stratakis *et al.* reported its use in C–C bond forming Hiyama-type reactions for the synthesis of aryl olefins. Further, they also reported the catalytic assistance of Au/TiO_2_ for the *cis*-1,2-dehydrogenative silylation of alkynes for the synthesis of 2,5-dihydro-1,2,5-oxadisiloles (49) (Scheme 18)^77^ from alkynes (48a) and dihydrosilanes in benzene at 25 °C followed by hydrolysis using water in a one-pot synthesis.

**Scheme 19:**
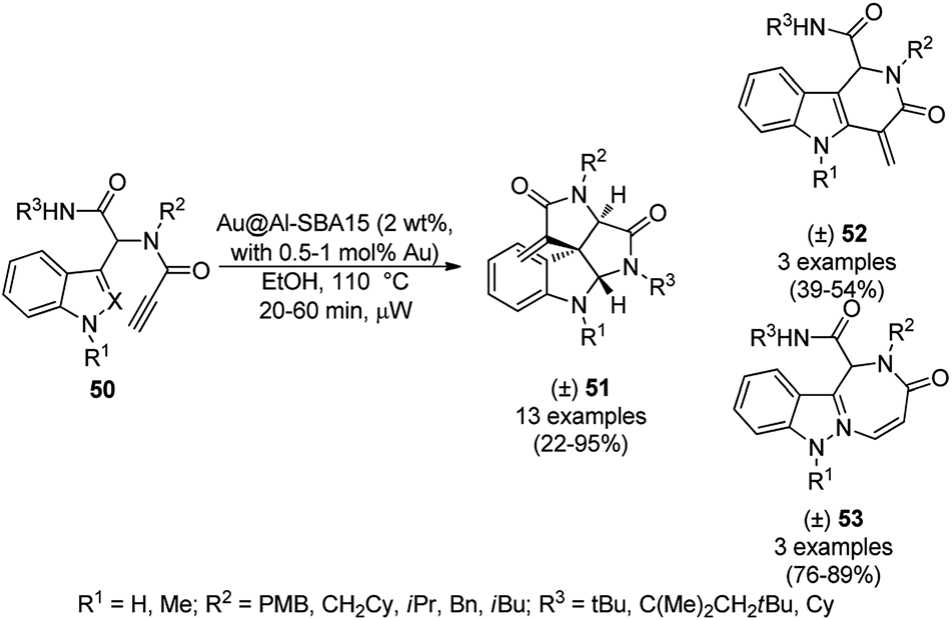
AuNP-catalyzed post-Ugi cycloisomerization of terminal alkynes (50).

Heterogeneous AuNP-immobilized Al-SBA15-catalyzed post-Ugi cycloisomerization for the synthesis of spiroindolines (50) and six and seven membered heterocycles (51 and 52) was achieved successfully by Eycken *et al.* from Ugi products having external alkynes without using ligands (Scheme 19).^78^ The NPs were obtained by ball milling Al-SBA15, which was previously synthesized from aluminium isopropoxide and tetraethyl orthosilicate, with AuNPs, and finally characterized *via* XRD, XPS, TEM, BET and ICP-AES. The same protocol for cycloisomerization was also investigated for internal alkynes. The catalyst was recycled for up to twelve times without loss of their catalytic potential and leaching of Au into the reaction mixture.

**Scheme 20:**
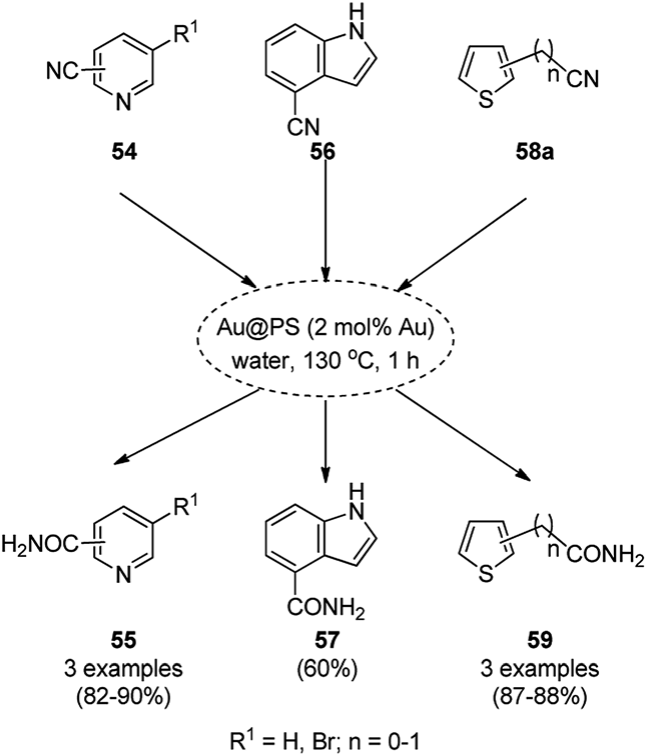
Catalytic hydration of cyanides (55/57/59) mediated under microwaves by Au@PS NPs.

AuNPs supported on polystyrene (Au@PS) were reported for the catalysis of base- and ligand-free hydration of heterocyclic cyanides (54/56/58a) to amides (55/57/59) in moderate to good yields at 130 °C (Scheme 20).^79^ The AuNPs were synthesized *via* a reduction–deposition approach from HAuCl_4_·3H_2_O and polystyrene resin followed by ion exchange with borohydride (BH_4_^−^) to obtain purple-colored Au@PS NPs. The fully characterized catalyst was reused and recycled for up to 8 times. Together with heterocyclic cyanides, the hydration of cyanoarenes was also achieved successfully with the AuNPs.

**Scheme 21:**
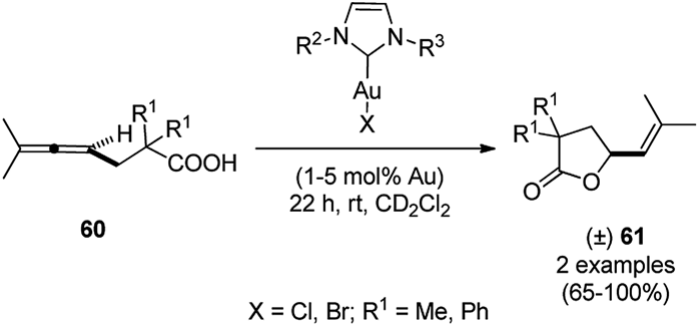
AuNP-catalyzed lactonization of allene-carboxylic acids (60).

For the first time, dendrimer-encapsulated NHC ligated AuNPs supported on silica were reported for the lactonization of allene-carboxylic acids (60) by Somorjai *et al.* (Scheme 21).^80^ They screened the following reaction with more than ten of these catalysts to study the effect of AuNPs on the lactonization of 60. The catalyst was recycled for up to four runs without loss of its catalytic stability.

### 3.3 CuNP-catalyzed synthesis of heterocycles

**Scheme 22:**
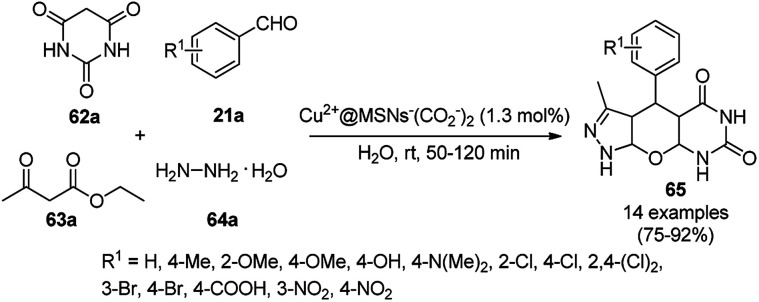
Cu_2_^+^@MSNs^−^(CO_2_)_2_-catalyzed synthesis of pyrazolopyranopyrimidine-5,7-diones (65).

A variety of synthetic transformations for the construction of organic compounds utilizing CuNPs was reviewed and summarized by Santra *et al.*,^81^ Wang *et al.*^82^ and by Das.^83^ In comparison with other transition metals, as the least toxic, CuNPs have the advantage of green nanocatalysts. The one-pot multicomponent and green synthesis of pyrazolopyranopyrimidine-5,7-diones (65) from barbituric acid (62a), substituted aromatic aldehydes (21a), ethyl acetoacetate (63a) and hydrazine hydrate (64a) was reported using Cu-immobilized mesoporous silica nanoparticles [Cu^2+^@MSNs^−^(CO_2_^−^)_2_] with a low catalytic loading (1.3 mol%) in aqueous conditions at rt (Scheme 22).^84^ The synthesized catalyst was well characterized *via* XRD, SEM, TEM, energy dispersive X-ray (EDX), thermal analysis (TGA-DTA) and FT-IR studies. The catalyst was recycled several times without obvious loss in its catalytic activity.

**Scheme 23:**
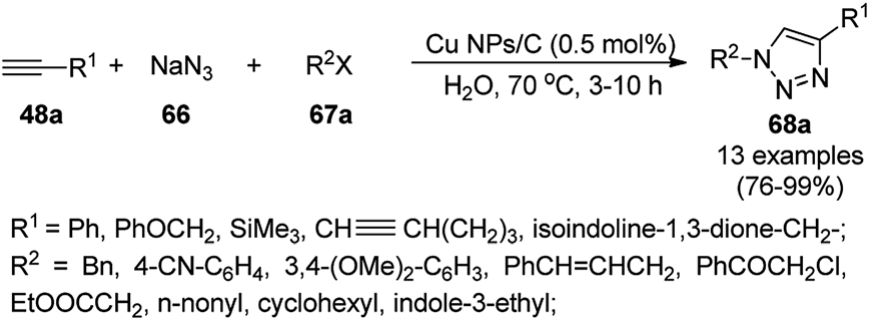
Synthesis of substituted triazoles (68a) using CuNPs.

Alonso *et al.* reported the synthesis of 1,2,3-triazoles (68a) from alkyl halide (66), sodium azide (67a) and substituted alkynes/acetylenes (48a) *via* multi-component Huisgen 1,3-dipolar cycloaddition (Scheme 23) at 70 °C in water using copper nanoparticles (CuNPs) on activated charcoal (0.5 mol%).^85^ For this model reaction, a variety of catalysts supports was tested, but activated carbon was chosen as the efficient support because of the shorter reaction time to produce triazoles. The nanoparticles on activated charcoal were characterized *via* ICP-MS, TEM, EDX, XPS, and selected-area electron-diffraction pattern (SAED). The versatility of copper catalysts through click chemistry was successfully demonstrated using azide precursors such as diazonium salt, aniline and epoxide.

**Scheme 24:**
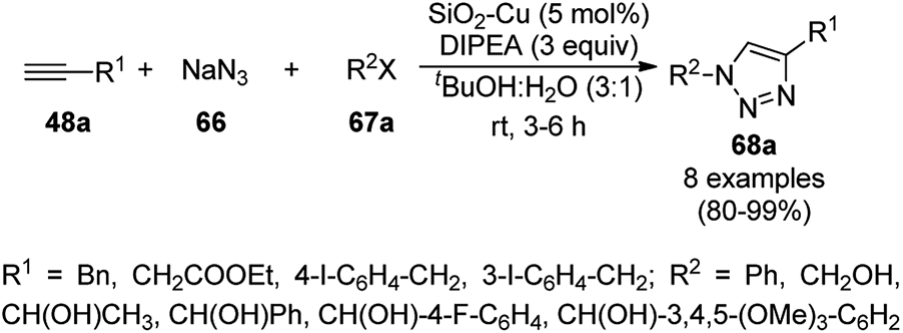
Synthesis of *N*-substituted triazoles (68a) catalyzed by CuNPs entrapped in a silica matrix.

Following a similar approach, Coelho *et al.* reported the synthesis of 68a*via* the Huisgen 1,3-dipolar cycloaddition of 48a, 66, and 67a catalyzed by sol–gel-entrapped copper in a silica matrix using di-isopropyl ethyl amine (DIPEA) in *tert*-butanol : water (3 : 1) at rt for 3–6 h (Scheme 24).^86^ These NPs were synthesized *via* a one-pot method involving a sol–gel process by immobilization of up to 9.4 wt% copper using copper iodide within a silica matrix, and they were characterized using SEM, TEM, EDS, and EPR. The hot filtration test revealed that leaching of the CuNPs occurred during the reaction, which could not proceed without the assistance of the catalyst. The insoluble heterogeneous CuNPs were involved in the catalytic cycle of Huisgen 1,3-dipolar cycloaddition, as confirmed by the three-phase test.

**Scheme 25:**
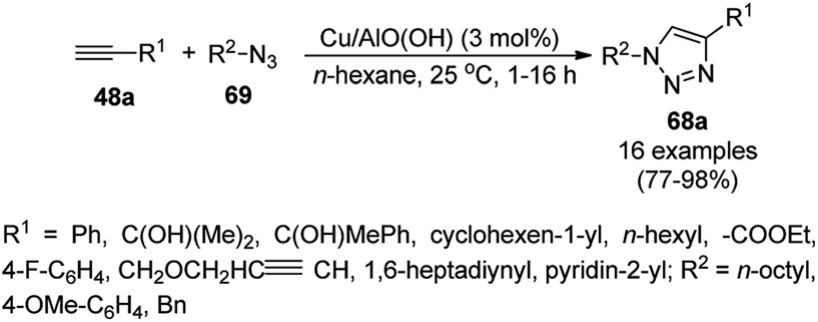
Synthesis of 1,4-disubstituted triazoles (68a) from alkyne (48a) and azide (69) catalyzed by CuNPs.

Park *et al.* reported the Huisgen [3 + 2] cycloaddition of terminal alkynes (48a) and substituted azides (69) without additives catalyzed by CuNPs in aluminum oxyhydroxide nanofibers using *n*-hexane as the solvent at rt (Scheme 25).^87^ The NCs were prepared from copper chloride (CuCl_2_·2H_2_O), Pluronic P123 as the stabilizer, and aluminum tri-*sec*-butoxide Al(*sec*-OBu)_3_, which were characterized *via* TEM, XPS, ICP, and nitrogen isotherms. The recycling of the nanocatalysts for the [3 + 2] cycloaddition was studied for up to five catalytic cycles with only 10% decay in catalytic potential, as studied using the model reaction of phenylacetylene and *n*-octyl azide in *n*-hexane for 12 h. ICP analysis confirmed that there was no significant leaching of Cu from the NPs in the reaction mixture during the reaction.

**Scheme 26:**
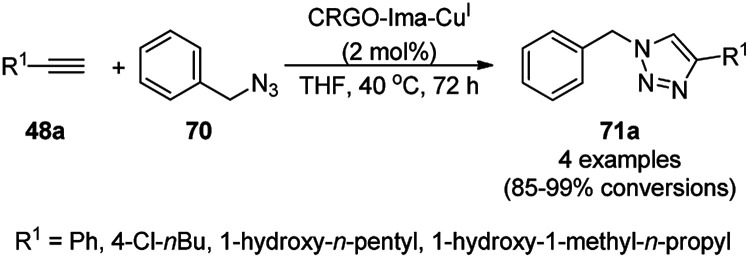
Synthesis of triazoles (70) catalyzed by CRGO-Ima-Cu^I^ NCs.

Cu(i) NPs supported on chemically reduced graphene oxide (CRGO-Ima-Cu^I^) were synthesized *via* sequential chlorination, azidation, and click reaction using 1-propyl-3-methylimidazolium bromide followed by loading of copper using tetrakis(acetonitrile)copper(i) hexafluorophosphate (Scheme 26).^88^ CRGO-Ima-Cu^I^ was further successfully employed in the Huisgen [3 + 2] cycloaddition of 48a with benzyl azides (70) for the synthesis of triazoles (71a) in 98–99% conversion. The catalyst was recycled for up to ten reuses with a good catalytic performance.

**Scheme 27:**
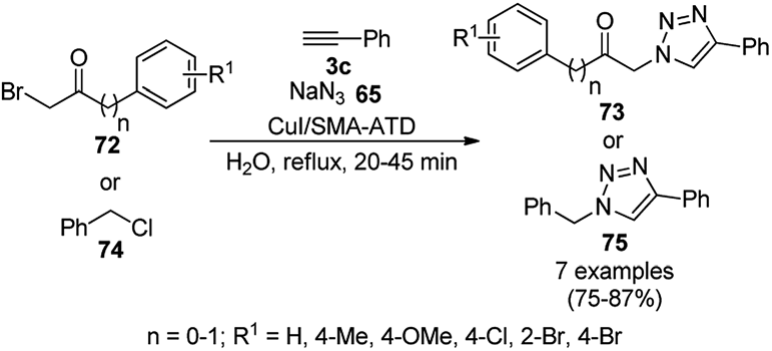
Click reaction for the synthesis of triazoles (73/75) catalyzed by CuNPs.

Heravi *et al.* reported the synthesis of triazoles (73/75) catalyzed by copper iodide (CuI) NPs immobilized on modified polystyrene-*co*-maleic anhydride (SMA, Scheme 27).^89^ SMA was modified by treatment with 4-amino-2-methyl-10*H*-thiene[2,3-][1,5]-benzodiazepine (ATD) hydrochloride, followed by the catalytic loading of copper using CuI. These CuNPs (CuI/SMI-ATD) were fully characterized *via* FT-IR, ^1^H NMR, SEM, TEM, EDAX and ICP-AES analysis. In the click reaction, from a mechanistic point of view, CuNPs take part *via* the formation of copper acetylide and preventing the conversion of Cu(i) into Cu(ii). The NPs were recycled for up to five times without loss in their catalytic activity. The stability of the complex formed with the NPs was studied at the M06/6-31G* level to reveal the immobilization of the CuNPs on the nitrogen site of the polymer-supported catalysts.

**Scheme 28:**
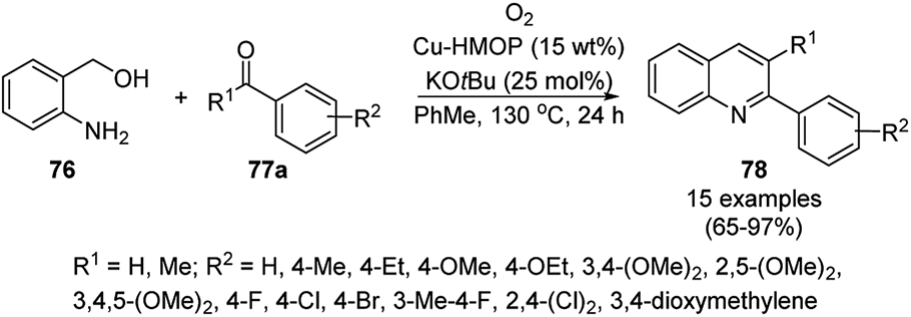
Frieldländer synthesis of quinolines (78) catalyzed by Cu-HMOP.

**Scheme 29:**
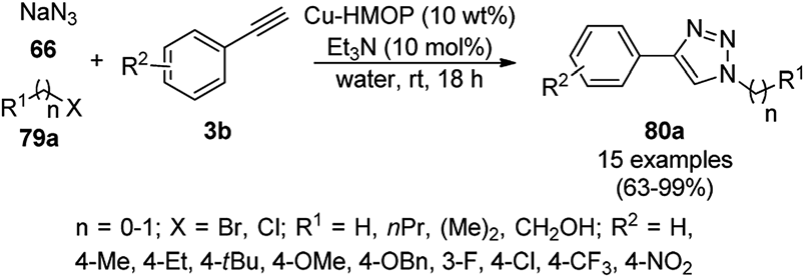
Click reaction for the synthesis of triazoles (80a) catalyzed by Cu-HMOP.

Copper-loaded hierarchical mesoporous organic polymer (HMOP) NC-catalyzed Frieldländer annulation for the synthesis of quinolines (78) was achieved from 2-amino benzyl alcohols (76) and aryl ketones (77a) under aerobic conditions (Scheme 28).^90^ Further, the same catalyst was used for the synthesis of 3-substituted 4-phenyl-1*H*-1,2,3-triazoles (80a) *via* the [3 + 2] cycloaddition of sodium azide (66) with substituted phenyl acetylene (3b), followed by nucleophilic substitution with alkyl halides (79a, Scheme 29). This protocol was also extended for the aerobic dehydrogenation of acyclic amines, 2,3-dihydroindoles and 1,2,3,4-tetrahydroquinolines. The catalyst was separated by centrifugation and recycled for five successive runs. The present protocol offers many advantages such as low catalytic loading, environmentally friendly nature, and use of a green solvent.

**Scheme 30:**
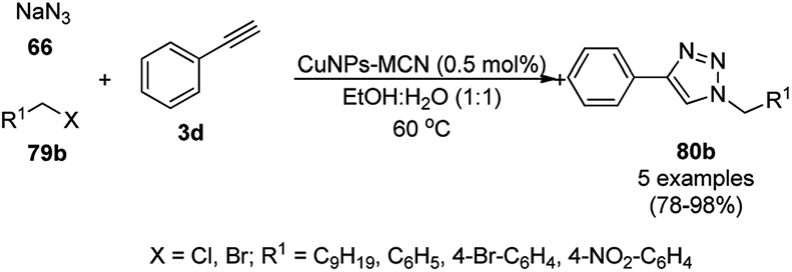
CuNP-catalyzed 1,3-dipolar cycloaddition for the synthesis of triazoles (80b).

**Scheme 31:**
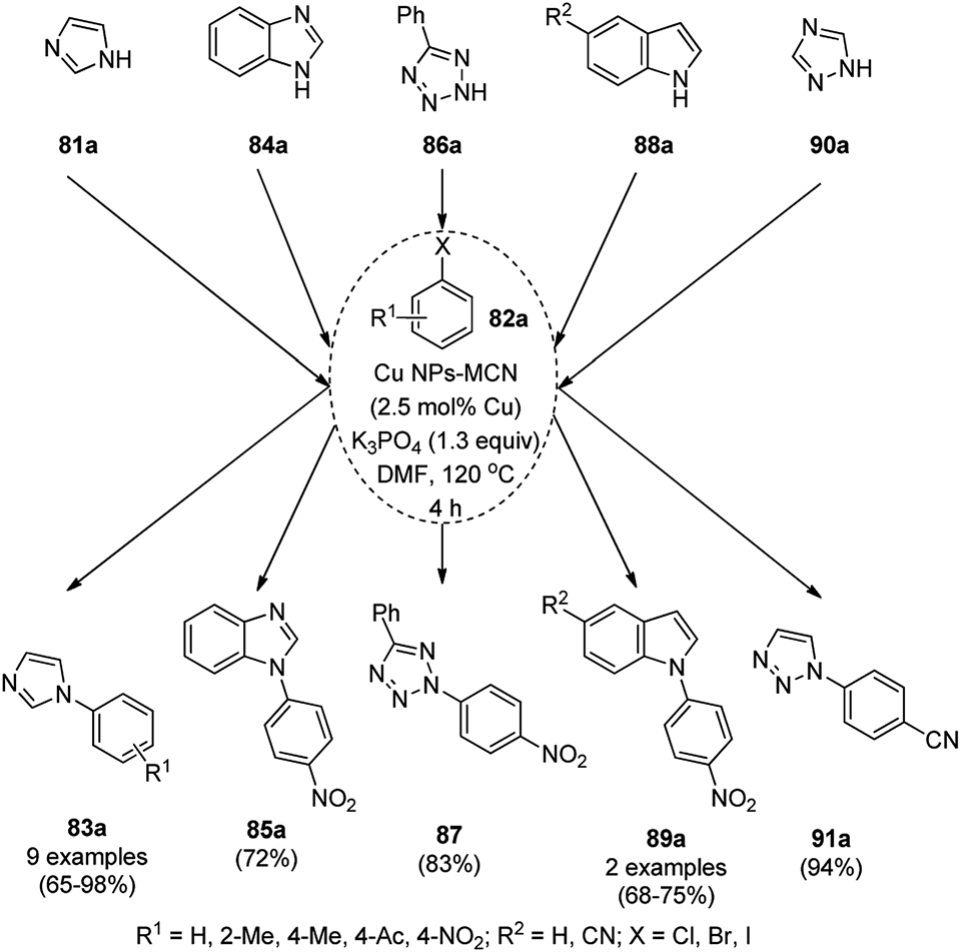
*N*-Arylation of N–H heterocycles reported by Dabiri *et al.*

Dabiri *et al.* reported the catalytic use of CuNPs supported on mesoporous carbon nitride (CuNPs-MCN) for the synthesis of 1,2,3-triazoles (80b) *via* the Huisgen 1,3-dipolar cycloaddition of sodium azide (66), alkyl halide (79b) and alkyne (3b) in 78–98% yield (Scheme 30).^91^ They also used the same catalyst for the *N*-arylation of NH-heterocycles such as imidazole (81a), benzimidazole (84a), 3-phenyl-1,2,4,5-tetrazole (86a), and 5-substituted indole (88a), and 1,2,4-triazole (90a, Scheme 31) *via* their reaction with substituted halobenzenes (82a) in DMF at 120 °C. The NPs were prepared *via* the treatment of MCN with copper(iii) nitrate (Cu(NO_3_)_2_·3H_2_O) following the addition of a reducing agent such as ascorbic acid. The excellent yields (96–98%) of 80b (R^1^ = Ph) synthesized *via* the 1,3-dipolar cycloaddition of 66, 3d and benzyl bromide during eight reuses demonstrated the stability of the CuNPs, which was also confirmed by Dabiri *et al. via* TEM of the CuNPs obtained at the end of the eighth run. The present protocol was found to give high yields in a short time compared to other reported protocols.^92–95^

**Scheme 32:**
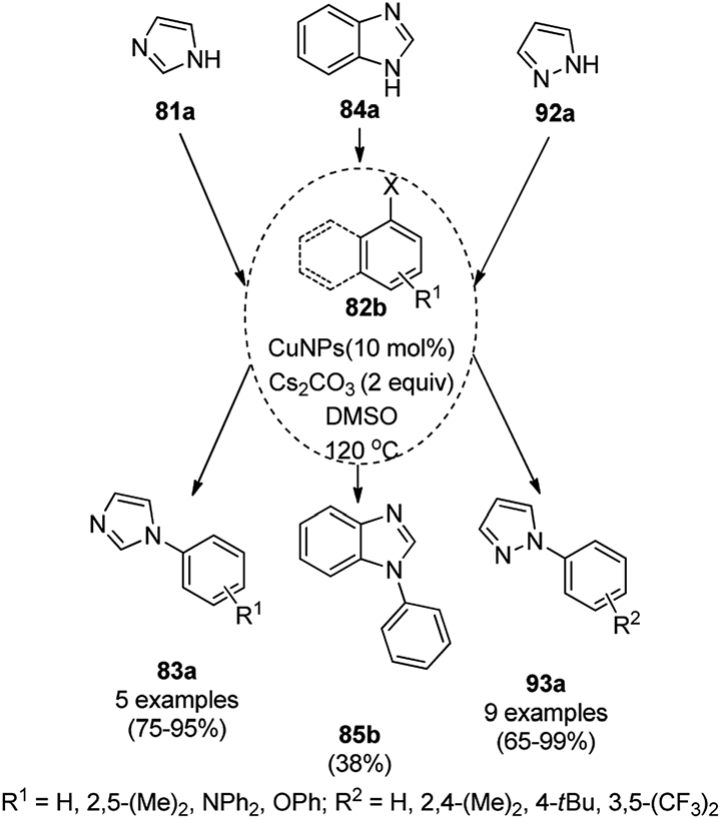
CuNP-catalyzed *N*-arylation of imidazoles (81a), benzimidazole (84a) and pyrazole (92a).

Yuan *et al.* reported the synthesis of *N*-arylated imidazoles/pyrazoles/benzimidazole (83a/85b/93a) from 5 or 6-5 membered N-containing heterocyclic compounds (81a/84a/92a) and aryl halides (82b) using copper nanoparticles in a catalytic amount (10 mol%) in DMSO at 120 °C using cesium carbonate as the base (Scheme 32).^96^ Nitrogen-rich copolymeric microsheets synthesized from melamine and cyanuric hydrochloride were treated with copper(ii) acetate and hydrazine hydrate to prepare the CuNPs. These NPs were further characterized *via* XPS, TEM, XRD and ICP-AES. The recyclability of the NCs was studied for up to five catalytic cycles; however, the catalyst gave a reduced yield of *N*-arylated imidazole of less than 50% after the fifth catalytic run.

**Scheme 33:**
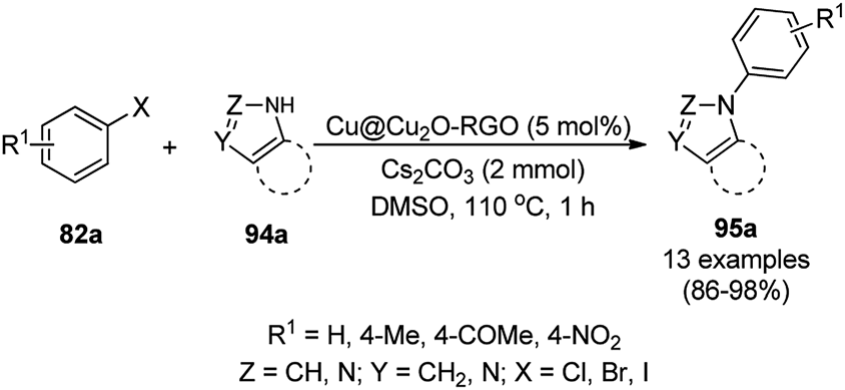
*N*-Arylation of imidazoles/triazoles/benzimidazoles (82a) catalyzed by CuNPs.

Similarly, Bazgir *et al.* reported the synthesis of *N*-arylated N-containing heterocycles (95a) using core–shell Cu@Cu_2_O NPs on reduced graphene oxide under aerobic conditions in a catalytic amount (5 mol%) using cesium carbonate as the base and DMSO as the solvent at 110 °C in 1 h (Scheme 33).^97^ The required CuNPs were synthesized *via* the reduction of reduced graphene oxide using l-ascorbic acid as the reducing agent followed by the catalytic loading of copper using copper sulfate (CuSO_4_) to obtain an actual catalytic loading of 26.2%, as confirmed by AAS. Further, the CuNPs were characterized *via* FT-IR and Raman spectroscopy, XRD, XPS, TEM, and EDX. Aryl iodides were found to yield more final product in comparison with aryl bromides and chlorides. Bazgir *et al.* claimed the efficient recyclability of the CuNPs, as evident from the 75% yield of the final product and 1.3% leaching of Cu at the end of the fifth catalytic run. The developed protocol was claimed to be simpler, less time-consuming and high yielding compared to reported works^92,98–100^ on the *N*-arylation of N–H heterocycles with aryl bromides catalyzed by heterogeneous copper catalysts.

**Scheme 34:**
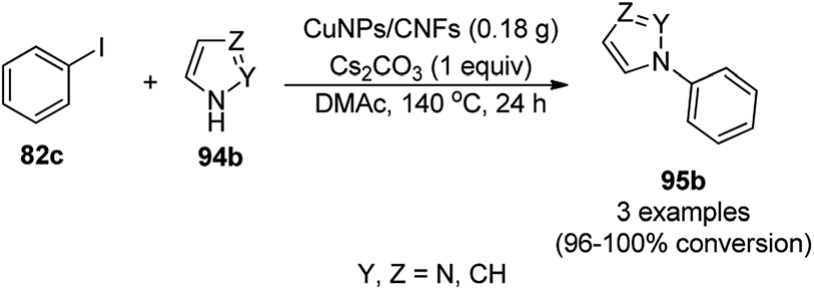
*N*-Arylation of N-containing heterocycles (94b) catalyzed by CuNPs.

Li *et al.* reported the easier, quicker and ligand-free synthesis of *N*-arylated azoles (95b) such as imidazole, pyrazole and pyrrole (94b) *via* Ullmann-type coupling using CuNPs supported on carbon nanofibers (CuNPs/CNF) using Cs_2_CO_3_ as the base and dimethylacetamide (DMAc) as the solvent at 140 °C for 24 h (Scheme 34).^101^ A similar protocol was also explored in detail for the *O*-arylation of iodobenzene with substituted phenols in moderate to excellent conversions. CuNPs/CNF was prepared *via* the reduction of copper nitrate followed by its treatment with carbon nanofibers processed at high temperature calcination and characterized using FESEM, XPS, and XRD. The recyclability of the catalyst was studied for five cycles, yielding 64% conversion to phenoxybenzene from iodobenzene (82c) and phenol.

**Scheme 35:**
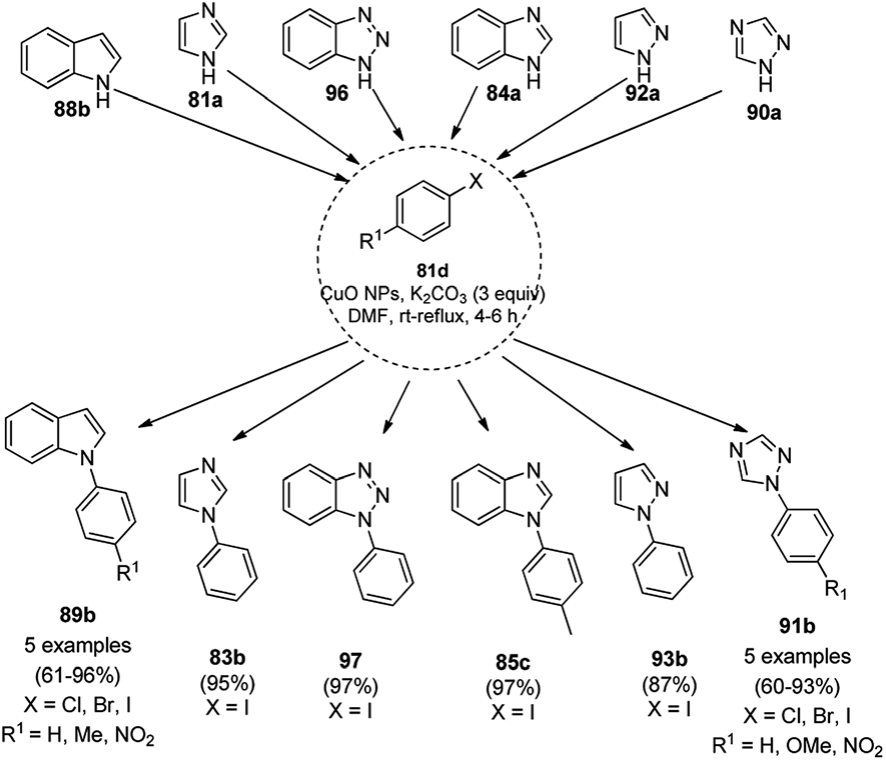
*N*-Arylation of azoles with haloarenes (81d) catalyzed by CuO NPs.

Nasrollahzadeh *et al.* reported the ligand-free *N*-arylation of azoles such as indoles (88b), imidazoles (81a), benzotriazole (96), benzimidazole (84a), pyrazole (92a), and 1,2,4-triazoles (90a) with haloarenes (82d) using CuO NPs as the catalyst, potassium carbonate as the base, and DMF as the solvent at rt to reflux conditions in moderate to excellent yields (Scheme 35).^102^ The green synthesis of CuO NPs was achieved *via* the treatment of the leaf extract of *Tamarix gallica* (family: Tamaricaceae), which contains several polyphenols as anti-oxidants, having reducing and anti-capping ability with an aqueous solution of CuCl_2_ at 70 °C. The structural integrity of the synthesized CuO NPs was confirmed *via* TEM, UV-Vis, FT-IR and powder XRD. The present protocol developed by Nasrollahzadeh *et al.*^102^ was tested for recycling of the catalyst with the reaction of iodobenzene and 90a, where the catalyst was recycled for up to five catalytic runs with only 4% of product loss in comparison with the first cycle.

**Scheme 36:**
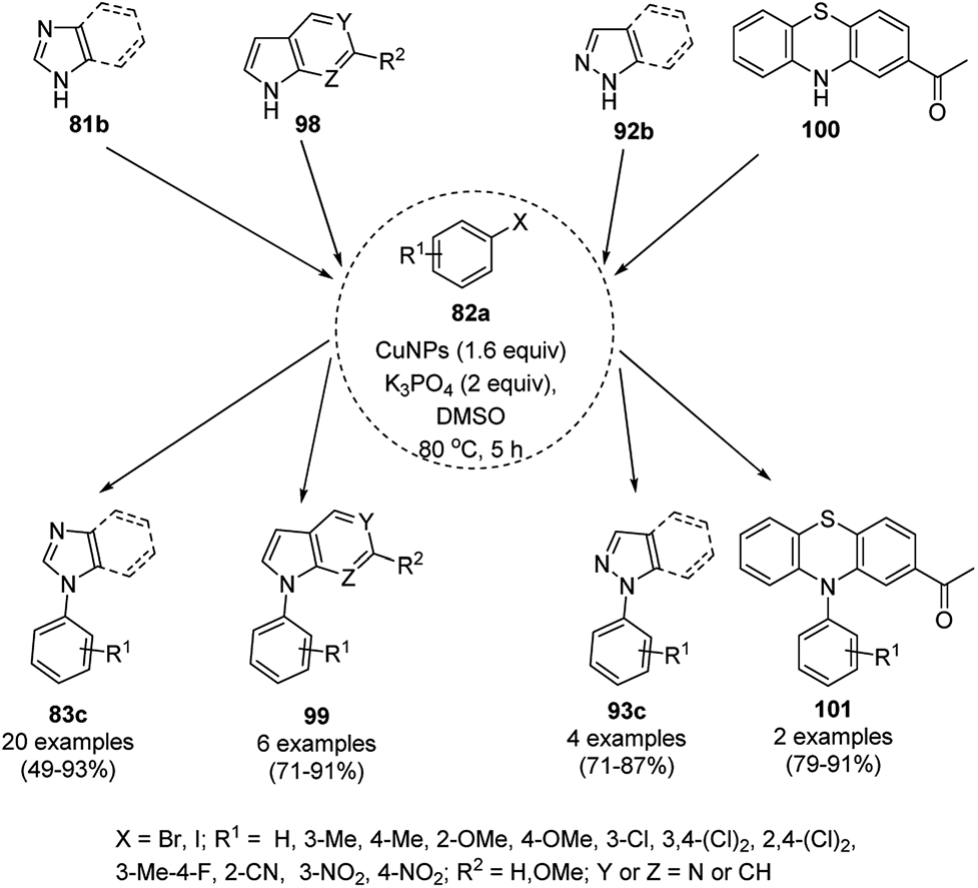
*N*-Arylation of N-containing heterocycles reported by Chattopadhyay *et al.*

The *N*-arylation of heterocyclic compounds such as imidazoles/benzimidazoles (81b), indoles/azaindoles (98), pyrazoles/benzopyrazoles (92b) and phenothiazine (100) catalyzed by CuNPs as NCs with aryl bromides/iodides using potassium phosphate as the base and DMSO as the solvent at 80 °C in 49–93% yield was reported by Chattopadhyay *et al.* (Scheme 36).^103^ The reaction selectively proceeded with aryl iodides in a competitive manner with the chlorides/fluorides present in the aryl halides. The optimized protocol was shown to have a wide substrate scope for azoles and azines.

**Scheme 37:**
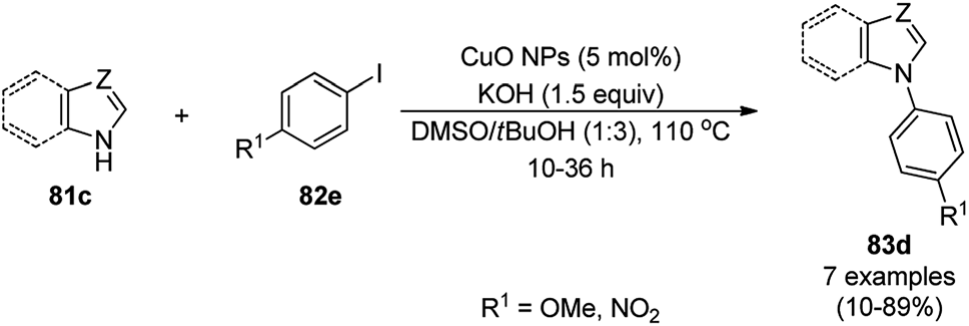
CuO NP-catalyzed C–N coupling of imidazoles, benzimidazoles, indoles (81c) with aryl iodides.

CuO NPs catalyzed the ligand-free C–N cross-coupling reactions of imidazoles, benzimidazoles, indoles (81c) with aryl iodides (82e) (Scheme 37) for the synthesis of *N*-arylated azoles (83d).^104^ The same protocol was also extended for the C–N, C–O and C–S cross-coupling reactions of amides, phenols and thiophenols with iodobenzenes. The reusability of the NCs was studied for the C–O cross-coupling reactions of phenol and iodobenzene for up to three cycles with yields of diphenyl ether of 95–97%.

**Scheme 38:**
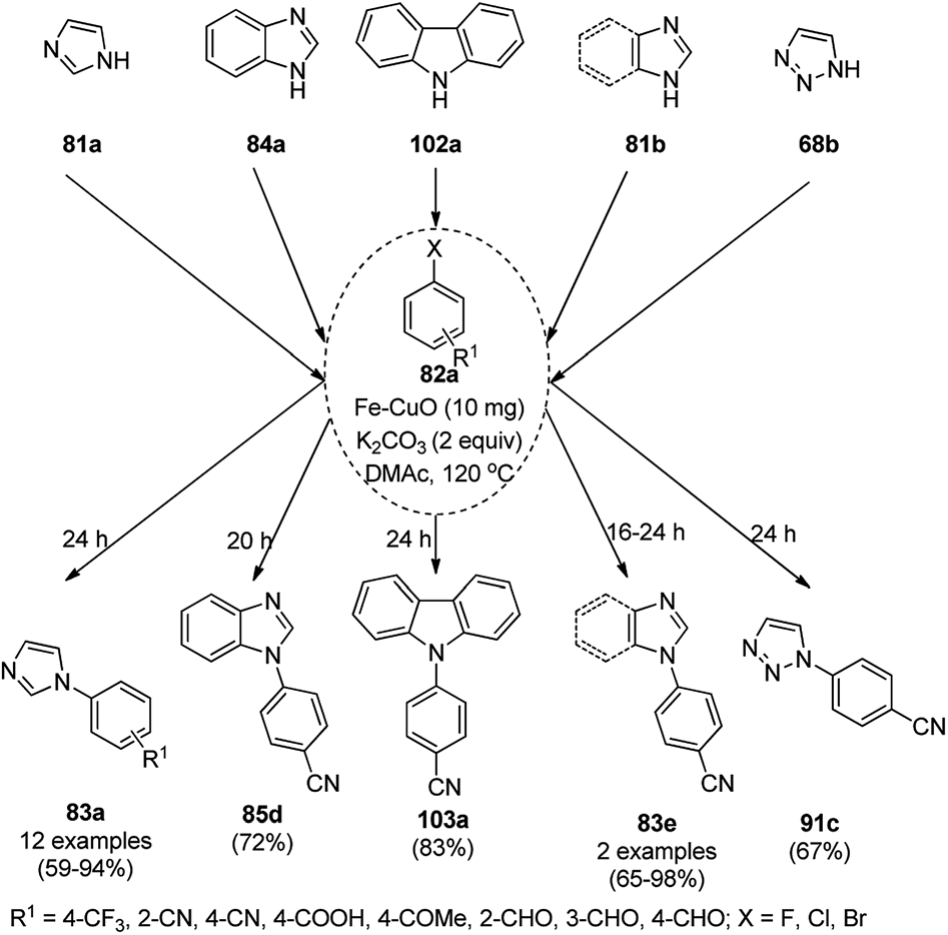
*N*-Arylation of heterocycles catalyzed by CuNPs.

The lepidocrocite (γ-FeOOH)-supported CuO NP (Fe–CuO)-catalyzed *N*-arylation of imidazoles (81a), benzimidazoles (84a), carbazoles (102a), pyrrole or indole (81a) and 1,2,3-triazoles (68b) was successfully achieved (Scheme 38) in dimethyl acetamide by Dhanuskodi *et al.*^105^ The magnetically retrievable NPs were prepared *via* the adsorption of Cu^2+^ from CuSO_4_·5H_2_O on previously synthesized lepidocrocite^106^ in alkaline solution. The catalyst was separated under an external magnetic field and recycled up to six times without loss in its catalytic activity. The higher yields observed with Fe–CuO with a particle size of 7 nm compared to that of 33 nm revealed that the larger number of active sites available with a higher surface area catalyze the *N*-arylation significantly.

**Scheme 39:**
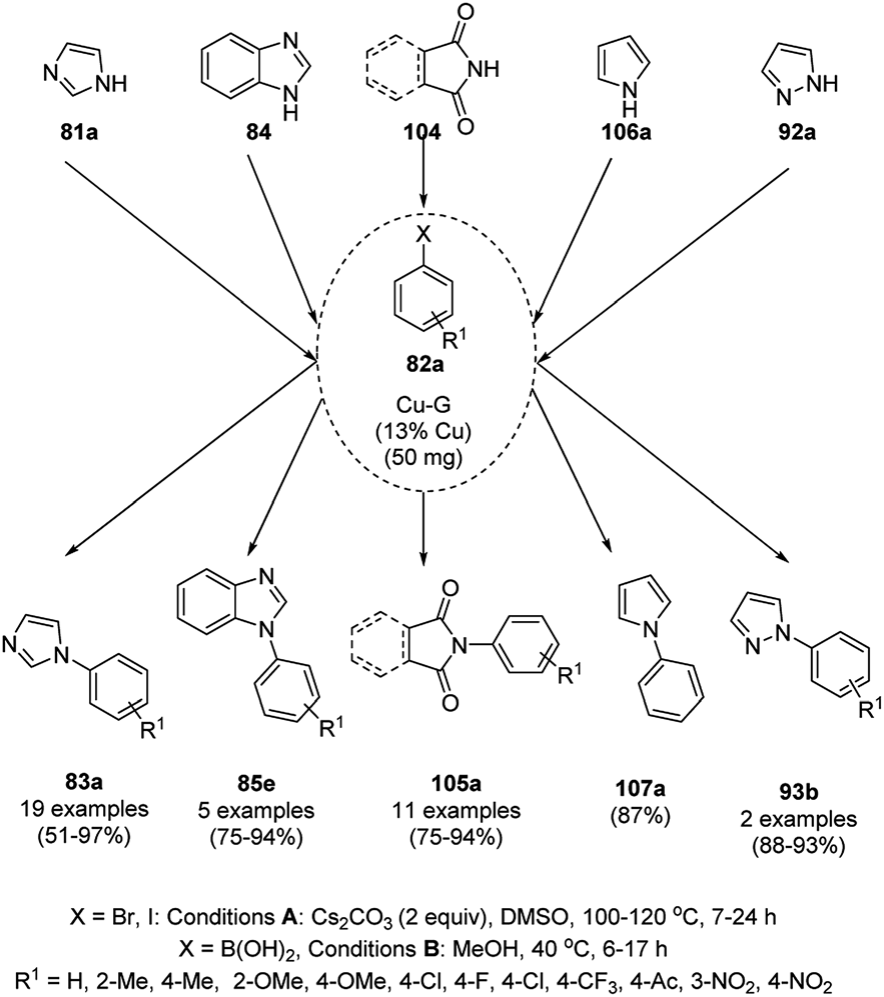
*N*-Arylation of heterocycles catalyzed by CuNPs.

CuNP-graphene-based nanocomposites were reported for the *N*-arylation of N–H heterocycles such as imidazole (81a), benzimidazoles (84a), succinimide or phthalimide (104), pyrrole (106a) and pyrazole (92a) by Jana *et al.* (Scheme 39) using haloarenes and aryl boronic acids (82a).^107^ The same catalyst was also explored for *O*-arylation using aryl halides and phenols. The CuNP-graphene-based nanocomposites were synthesized *via* the treatment of GO with copper acetate and hydrazine and characterized *via* UV-Vis, AFM, TEM, Raman spectra, and XPS. The catalyst was recycled after filtration for up to seven runs of *O*-arylation with no change in catalytic performance. The present protocol was claimed to be high yielding compared to the reported protocols for the *N*-arylation of imidazole with iodobenzene such as copper-exchanged fluorapatite (CUFAP),^108^ Cu_2_O,^109^ and nano-Cuo,^110^ and that of imidazole with phenyl boronic acids using copper-exchanged fluorapatite (Cu-FAP)^111^ and poly aniline-supported CuI (PANI-Cu).^112^

**Scheme 40:**
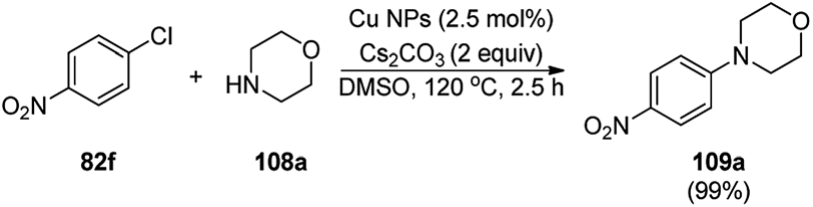
CuNPs catalyzed C–N coupling of *p*-chloro nitrobenzene (82f) with morpholine (108a).

Cuboctahedral-shaped CuNPs were prepared *via* the reduction of Cu^2+^ from copper sulphate (CuSO_4_) with sodium hydroxide, hydrazine hydrate as the reducing agent (Scheme 40) and poly(acrylic acid) (PAA) as the capping agent.^113^ The prepared NPs were used for the C–N coupling of *p*-chloro nitrobenzene (82f) with morpholine (108a) to C–N coupled product (109a). The same NPs were also used to catalyze the Mannich reaction among acetophenone, benzaldehyde and aniline.

**Scheme 41:**
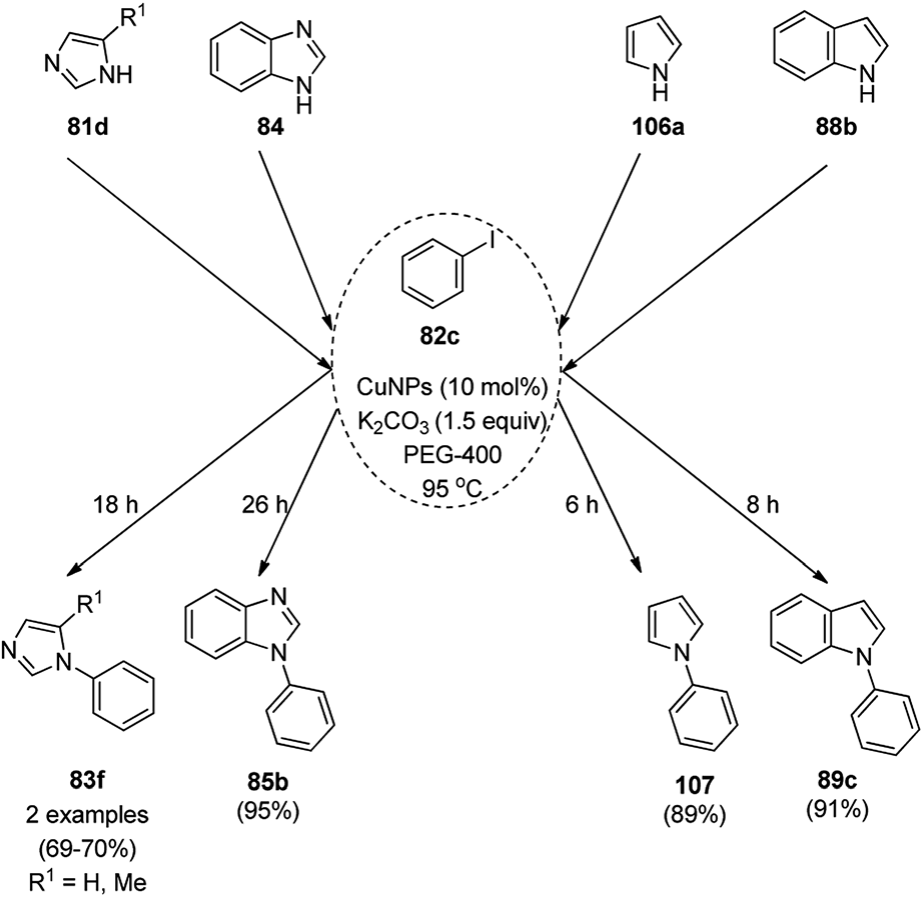
CuNP-catalyzed *N*-arylation of N–H heterocycles.

Kidwai *et al.* have reported C–N coupling of N–H heterocycles such as imidazoles (81d), benzimidazole (84a), pyrrole (106a) and indole (88b) with iodobenzene (82c, Scheme 41) in polyethylene glycol (PEG-400).^114^ CuNPs have been prepared by the treatment of micellar solution of CuSO_4_ and N_2_H_2_. It has been also found successful for the *N*-arylation of substituted anilines with haloarenes in moderate to excellent yields. Size screening of CuNPs for the synthesis of *N*-aryl aniline revealed that diminishing the particle of CUNPs increased the yields of *N*-arylated anilines.

**Scheme 42:**
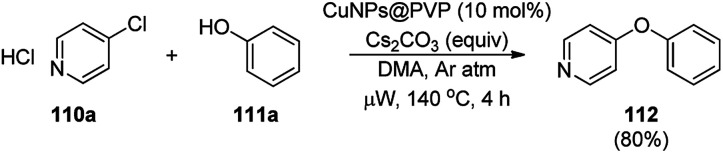
CuNP-catalyzed Ullmann coupling for the synthesis 4-pyridyl phenyl ether (112).

CuNPs protected by polyvinyl pyrrolidone (CuNPs@PVP) catalyzed Ullmann ether synthesis of 4-phenoxypyridine (112) from 4-chloropyridine hydrochloride (110a) and inactivated phenol (111a) was reported by Wheatley *et al.* (Scheme 42).^115^ The CuNPs were prepared from copper(ii) acetate and ploy(*N*-vinylpyrrolidone) and characterized *via* HRTEM, EDS, and PXRD. The higher yields achieved by microwave heating were attributed to the rapid energy consumption in one minute and uniform volumetric heating compared to conventional heating.

**Scheme 43:**
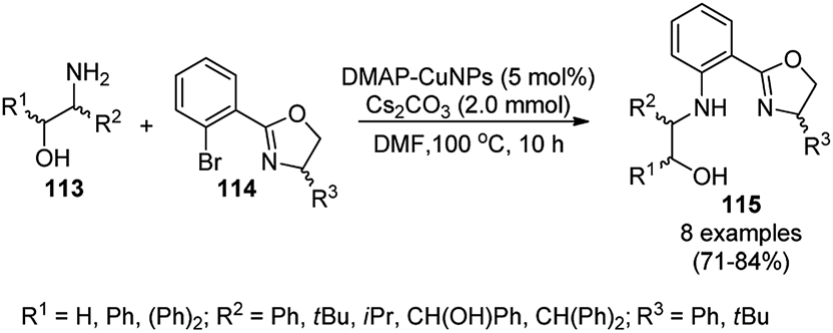
Ullmann coupling of β-aminoalcohols (113) with aryl bromides (114) catalyzed by DMAP-CuNPs.

Yuan *et al.* reported the C–N bond formation using β-aminoalcohols (113) and aryl bromides (114) containing an oxazole ring catalyzed by 4-dimethylaminopyridine (DMAP)-stabilized copper nanoparticles (DMAP-CuNPs) in the presence of cesium carbonate as the base and DMF as the solvent at 100 °C for 10 h (Scheme 43).^116^ The NPs were synthesized *via* the controlled decomposition of DMAP-Cu(acac)_2_-carbohydrazide complex (acac: acetylacetonate) in water-free ethanol and characterized *via* TEM and XPS. The DMAP-CuNPs catalyzed the reaction in a homogenous medium in DMF and could be recovered by making them insoluble *via* the addition of diethyl ether or toluene. The CuNP-catalyzed Ullmann coupling was found to be significant with aryl bromides and iodides rather than chlorides and fluorides. The recyclability of the DMAP-CuNPs was studied in the reaction between imidazole and phenyl bromide to form *N*-phenyl imidazole, where they maintained their catalytic activity. This protocol was also extended for the *N*-arylation of imidazoles/pyrazoles/benzimidazoles/trifluroacetamides with aryl bromides having substituted oxazole in good to excellent yields.

**Scheme 44:**
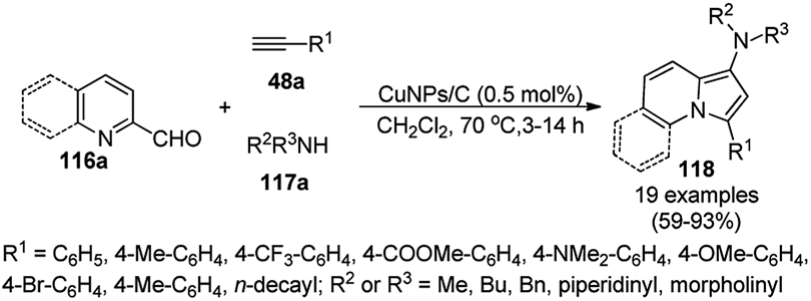
CuNP-supported activated charcoal-catalyzed synthesis of indolizines (118d).

**Scheme 45:**
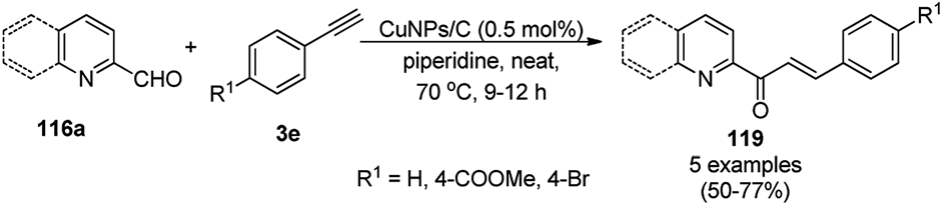
CuNP-supported activated charcoal-catalyzed synthesis of heterocyclic chalcones (119).

Alonso *et al.* reported the multicomponent synthesis of indolizines (118) from pyridine-2-carbaldehyde (116a), acetylenes (48a) and amine (117a) catalyzed by CuNPs supported on activated charcoal (0.5 mol%) using dichloromethane as the solvent at 70 °C (Scheme 44).^117^ The developed catalyst was also found to be successful for the synthesis of heterocyclic chalcone (119) from pyridine-2-carbaldehyde (116a) and phenyl acetylene derivatives (3e) in the presence of piperidine under neat conditions (Scheme 45). The CuNPs were prepared from copper(ii) chloride, lithium metal, and 4,4′-di-*tert*-butylbiphenyl in THF at rt following a reported procedure.^85^

**Scheme 46:**
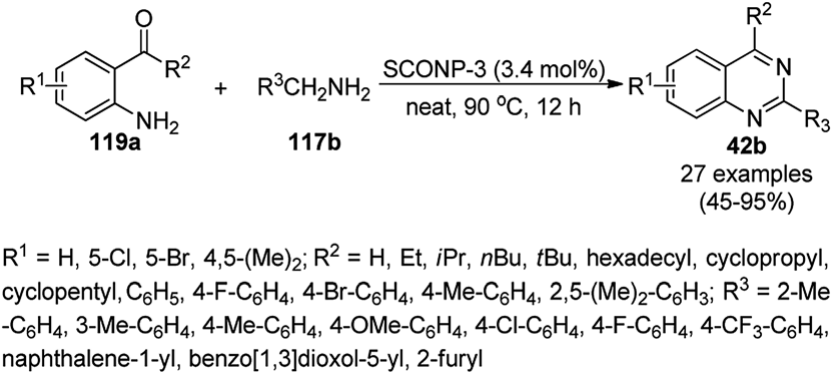
Synthesis of 2,4-disubstituted quinazolines (42b) from 2-aminobenzoketones (119a) and aryl or heteroaryl alkyl amine (117b).

Wang *et al.* reported the synthesis of 2,4-disubstituted quinazolines (42b) from 2-aminobenzoketones (119a) and aryl or heteroaryl alkyl amine (117b) at 90 °C under solvent-free conditions using copper oxide nanoparticles supported on kaolin (SCONP-3) as a heterogeneous catalyst in moderate to excellent yields (Scheme 46).^118^ SCONP-3 was prepared from Cu(NO_3_)_2_·3H_2_O and kaolin followed by its treatment with aqueous Na_2_CO_3_ and calcination at 350 °C. The recycling of the catalyst was demonstrated by Wang *et al.* for up to four catalytic cycles with 67% yield from the model reaction of 2-aminobenzophenone with benzylamine. Mechanistically, CuO NPs bring the coupling reagents close to each other and facilitate the cyclization *via* the coordination with the formed iminic bond.

**Scheme 47:**
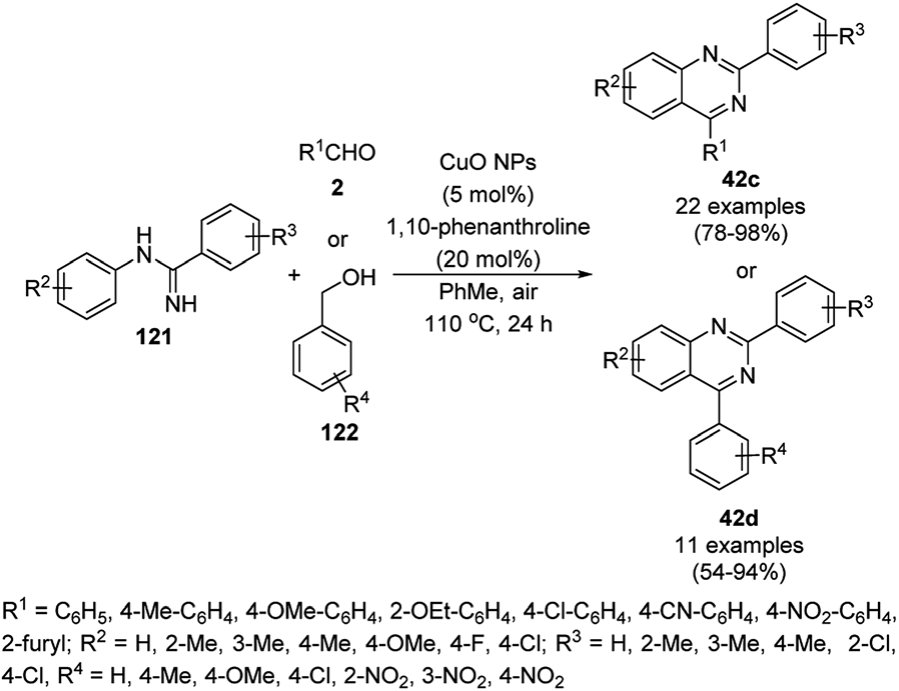
CuO NP-catalyzed synthesis of quinazolines (41c/d).

Zhang *et al.* also reported the synthesis of quinazolines (41c/d) catalyzed by CuO NPs *via* the oxidative coupling of substituted amidines (121) and substituted benzaldehydes (122) or benzyl alcohols (122) using 1,10-phenanthroline as the ligand and toluene as the solvent at 110 °C (Scheme 47).^119^ The developed protocol exhibited a wide scope with a variety of the substituents including electron-donating and withdrawing groups for the synthesis of quinazolines. The reaction with aryl aldehydes proceeded successfully for the synthesis of quinazolines, but did not occur with aliphatic aldehydes. The slight leaching of 2.7 ppm of Cu was observed using the model reaction between benzaldehyde and *N*-(4-chlorophenyl)benzimidamide, as confirmed by AAS. The XRD and TEM images of the NPs revealed that the catalyst maintained its integrity and morphology after three consecutive reuses, which allowed it to be employed for the next catalytic run without loss in catalytic activity.

**Scheme 48:**
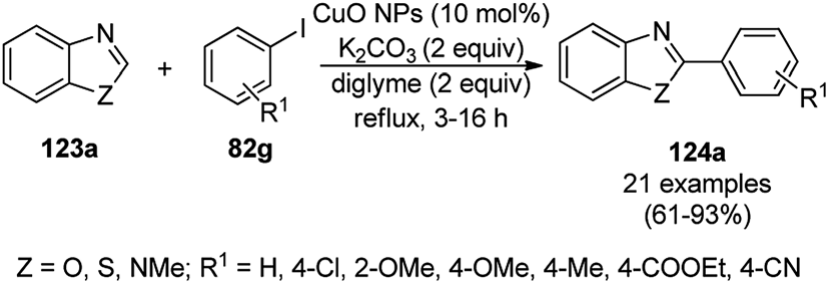
Synthesis of 2-phenylbenzazoles (124a) from benzazoles (123a) and aryl iodides (82g) catalyzed by CuO NPs.

Zhang *et al.* reported the synthesis of 2-phenyl benzazoles (124a) *via* the direct arylation of N-containing heterocycles from benzazoles (123a) and aryl iodides (82g) catalyzed by ligand-free CuO nanospindles using diglyme as the solvent and K_2_CO_3_ as a mild inorganic base under reflux in an argon environment (Scheme 48).^120^ The prepared CuO NPs were characterized *via* XRD and FE-SEM. The recyclability of the catalyst was studied using benzoxazole and iodobenzene as a model reaction for up to three catalytic runs, where the catalyst retained its morphology, as evident from the XRD and EDS spectra of the recycled NPs.

**Scheme 49:**
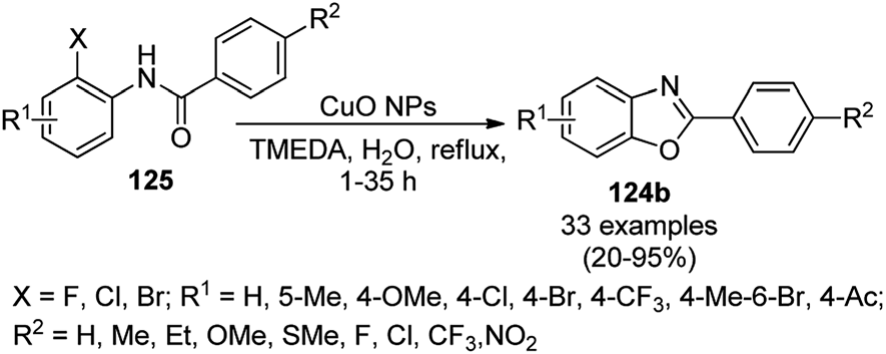
Synthesis of 2-arylbenzoxazoles (124b) from *o*-halobenzanilides (125) catalyzed by CuO NPs.

Patel *et al.* reported the green synthesis of 2-aryl benzoxazoles (124b) from *o*-halo benzanilides (125) using commercially available copper oxide (CuO) NPs in the presence of the organic base *N*,*N*,*N*′,*N*′-tetramethylethylenediamine (TMEDA) using water as the ultimate green solvent at 100 °C in 20–95% yield (Scheme 49).^121^ However, when 2-haloanilides (125) were treated with CuO NPs in the presence of an inorganic base such as caesium carbonate (CS_2_CO_3_), *o*-hydroxy phenylbenzamides were obtained as the major product. The recyclability of the catalyst was studied for up to five catalytic cycles without appreciable loss in its catalytic activity. However, a prolonged reaction time was required after the third and fifth catalytic cycles since agglomeration of the catalyst was observed.

**Scheme 50:**
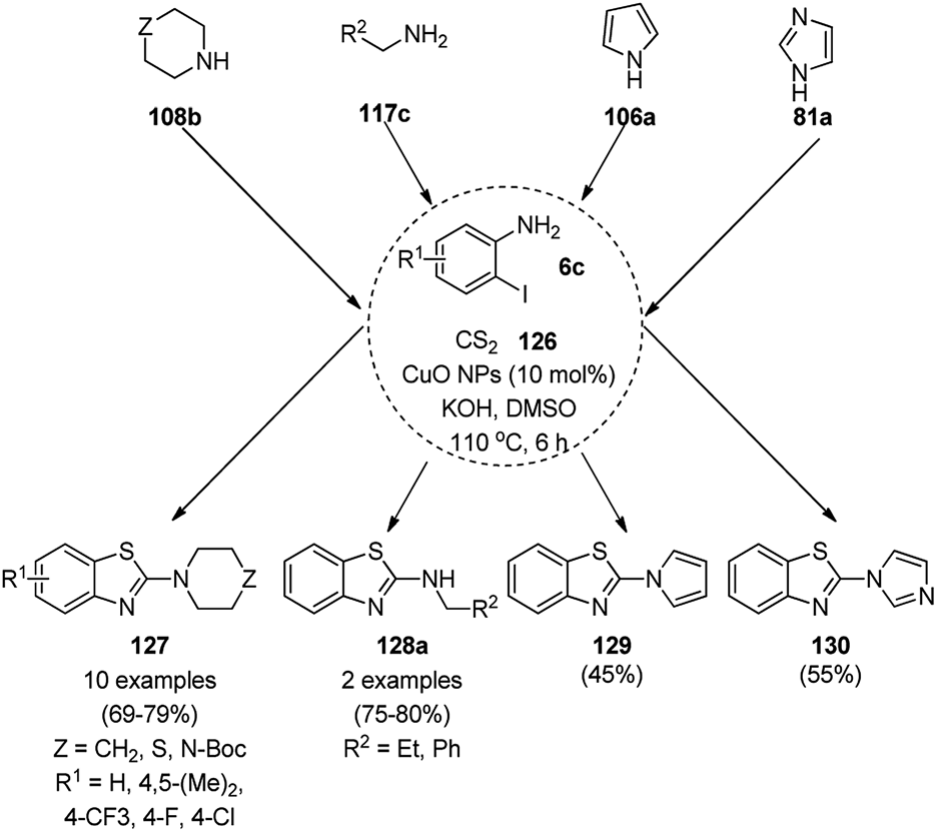
Cyclocondensation of 2-aminobenzothiazoles catalyzed by Cu(i) NPs.

Nageswar *et al.* reported the synthesis of 2-aminobenzo[*d*]thiazoles (127/128a/129/130) under ligand-free conditions from substituted *o*-iodoaniline (6c), carbon disulfide (126) and alicyclic/heteroaromatic/benzyl/aliphatic amines (108/117c/106a/81a) using Cu(i) NPs as the NC (10 mol%) and KOH as the alkali in DMSO at 110 °C in 55–80% yield (Scheme 50).^122^ This coupling was found to be effective to construct the C–S bond from carbon disulfide and diverse amines. The tandem process of cyclization started from *o*-iodoaniline and potassium dithiocarbamate formed from the reaction of the base, carbon disulfide and amine followed by C–S bond formation and aromatization *via* the removal of H_2_S gas. The NPs at the end of three catalytic cycles were found to maintain their nanoparticulate behavior (TEM) and only 8% of product loss was observed at the end of the third catalytic run for the model three-component reaction of *o*-iodoaniline, carbon disulfide and amines.

**Scheme 51:**
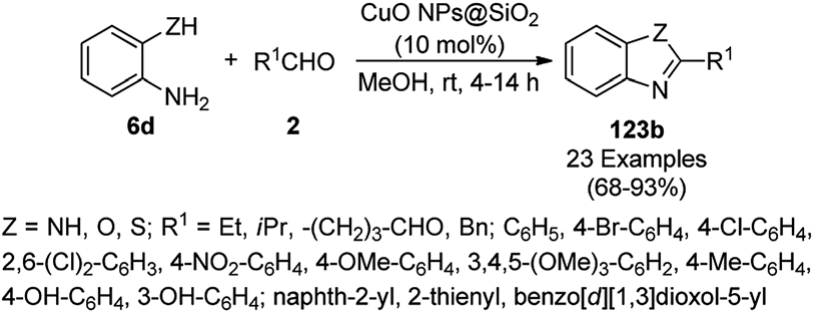
Synthesis of 2-substituted benzazoles (123b) from *o*-substituted anilines (6d) and aldehydes (2) catalyzed by heterogeneous NCs.

Mandal *et al.* reported the synthesis of 2-substituted benzazoles (123b) such as benzimidazoles/benzoxazoles/benzothiazoles from *o*-substituted anilines (6d), *viz.* 2-aminoanilines/2-aminophenols/2-aminothiophenols, and aryl/heteroaryl/aliphatic aldehydes (2) catalyzed by silica-supported CuO NPs using methanol as the solvent at rt in 68–93% yield (Scheme 51).^123^ In search of the best solid support, CuO NPs loaded on SiO_2_, montmarillonite, ZSM-5 and TiO_2_ were investigated, where the best results were observed with the CuO NPs loaded on silica. The recyclability of the catalyst was studied for up to five catalytic runs with significant yields of the 2-phenylbenzo[*d*]imidazoles between the reaction of *o*-phenylene diamine and benzaldehyde.

**Scheme 52:**
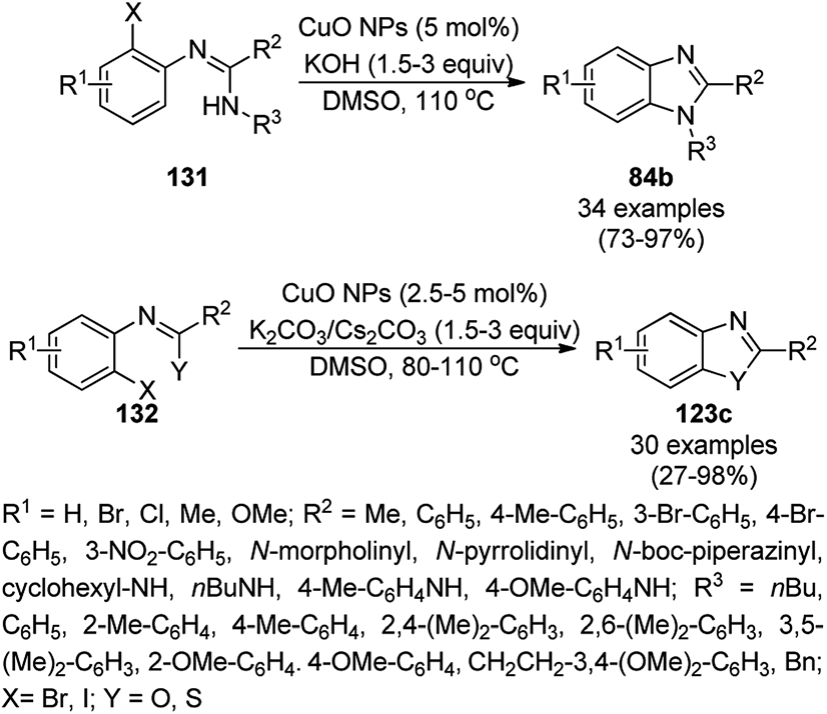
Intramolecular cyclization of *o*-haloarenes (131/132) catalyzed by CuO NPs for the synthesis of benzimidazoles (84b), benzothiazoles and benzoxazoles (123c).

CuO NPs catalyzed the ligand-free intramolecular cyclization of *o*-haloaryl derivatives (131) for the synthesis of benzimidazoles (84b) and benzothiazoles or benzoxazoles (123c) with a wide functional group tolerance (Scheme 52).^124^ The CuO NPs collected after centrifugation were recycled and reused for the synthesis of 2-phenyl benzoxazole for up to five runs without loss in their catalytic performance (97–100%) and texture (TEM and XRD). The supernatant collected during the cyclization of *o*-bromophenylbenzamide was subjected to AAS, which revealed zero leaching of Cu during the reaction.

**Scheme 53:**
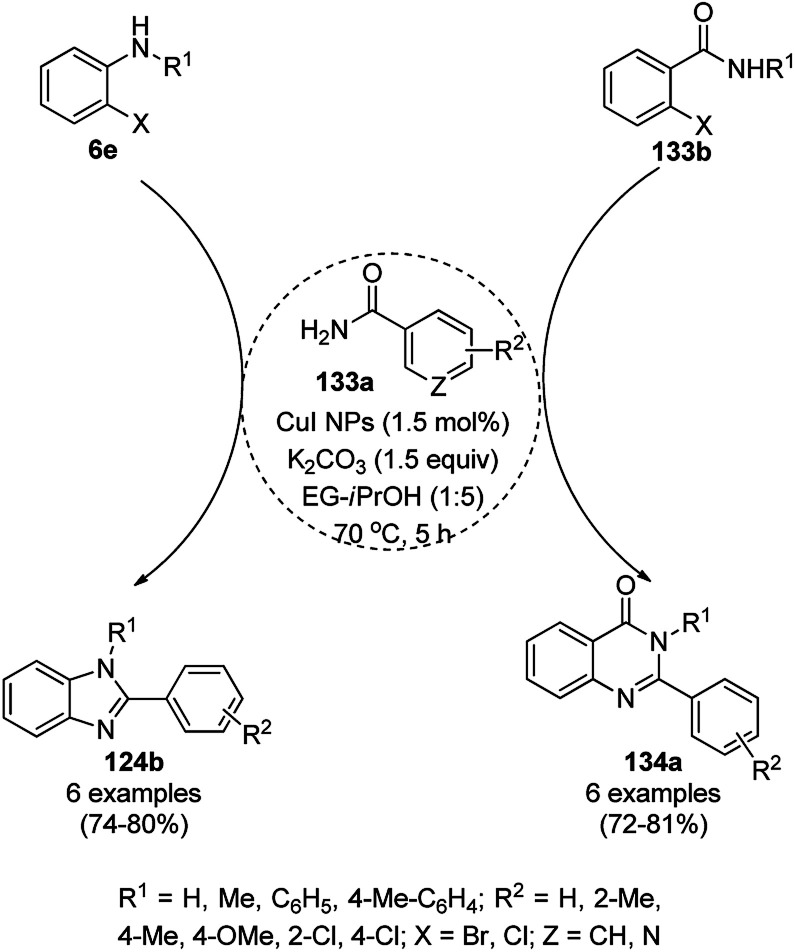
CuI NP-catalyzed synthesis of benzimidazoles (124b) and quinazolinones (134a).

The copper iodide NP-catalyzed intermolecular amidation-cyclization for the one-pot synthesis of benzimidazoles (124b) and quinazolinones (134a) was reported *via* the cyclocondensation of *o*-halo anilines (6e) or *o*-halo benzamides (133b) with substituted benzamides (133a, Scheme 53).^125^ The CuI NPs were prepared using copper acetate (Cu(OAc)_2_·H_2_O), dimethyl glyoxime and potassium iodide. The same catalyst was also explored for the synthesis of carboxamides and cyclic amides *via* the *N*-arylation of benzamides and succinimides with haloarenes. The recyclability of the CuI NPs was demonstrated for up to five reuses with 75–85% yield of *N*-phenyl benzamides.

**Scheme 54:**
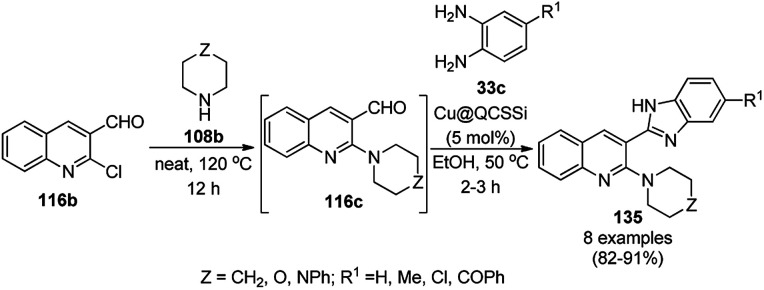
Synthesis of 3-(benzimidazol-2-yl)quinolines (135) catalyzed by Cu@QCSSi.

**Scheme 55:**
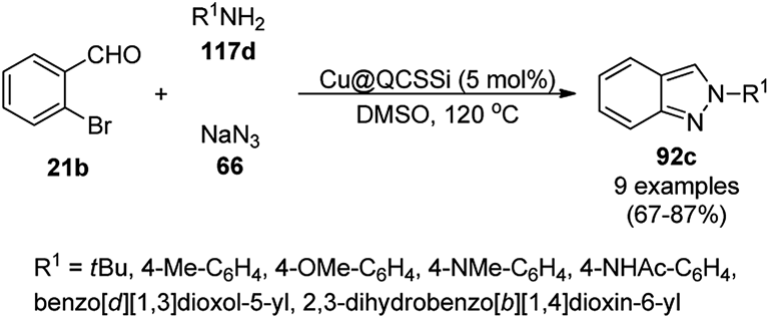
Synthesis of 2-substituted indazoles (92c) catalyzed by Cu@QCSSi.

A copper(ii) complex supported on amino-functionalized silica (Cu@QCSSi) catalyzed the cyclocondensation of *p*-substituted *o*-phenylene diamine (33c) with *in situ* formed 2-amino-substituted quinoline-3-carbaldehyde (116b) for the successful one-pot synthesis of 3-(benzimidazol-2-yl)quinolines (135) in good to excellent (Scheme 54).^126^ The intermediate 116c was obtained *via* the nucleophilic substitution of 2-chloroquinoline-3-carbaldehyde (116b) with alicyclic amines (108b). Further, the same catalyst was also explored for the synthesis of 2-substituted indazoles (92c) from 2-bromobenzaldehyde (21b), amines (117d) and sodium azide (66) in DMSO at 120 °C (Scheme 55). The required catalyst was synthesized by grafting 2-oxo-1,2-dihydroquinoline-3-carbaldehyde (QC) on amino-functionalized silica followed by complexation using copper acetate. To assess the recyclability of the catalyst, it was recycled and reused for up to seven times, giving 84–90% yield of product for the reaction among 116b, morpholine, and *o*-phenylene diamine.

**Scheme 56:**
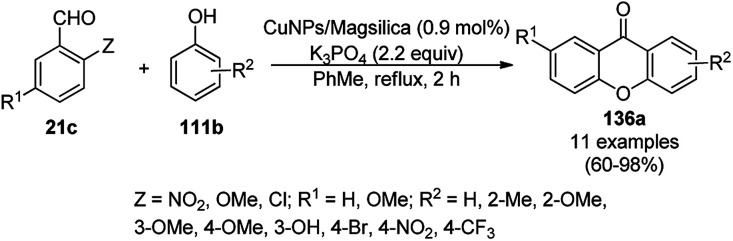
Synthesis of xanthones (136a) catalyzed by Cu-based magnetically recyclable NPs.

Gerbino *et al.* reported the ligand-free synthesis of xanthones (136a) from 2-substituted benzaldehydes (21c) and substituted phenols (111b) catalyzed by CuNPs supported on silica-coated maghemite (MagSilica) using potassium phosphate as the base and toluene as the solvent under reflux and argon in moderate to excellent yields (Scheme 56).^127^ The NCs could be recycled using an external magnet for up to four catalytic runs as the catalyst. Gerbino *et al.* reported about <50 ppb copper leaching, as confirmed by ICP-AES, making this method very economic.

**Scheme 57:**
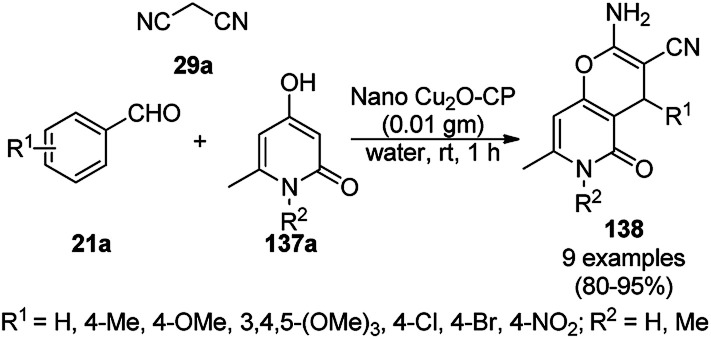
Synthesis of pyrano[3,2-*c*]pyridines (138) catalyzed by nano Cu_2_O-CP.

**Scheme 58:**
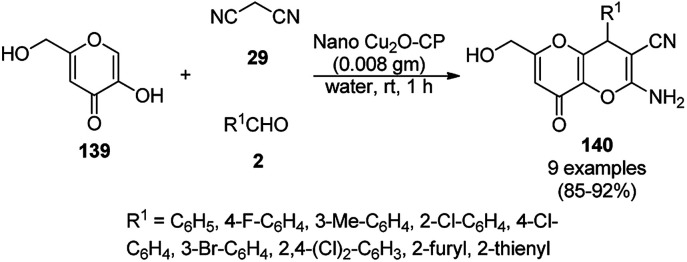
Synthesis of pyrano[3,2-*b*]pyranoes (140) catalyzed by nano Cu_2_O-CP.

Baghbanian S. M. reported the green synthesis of pyrano[3,2-*b*]pyranones (138, Scheme 57) and pyrano[3,2-*c*]pyridones (140, Scheme 58) catalyzed by Cu_2_O NPs supported on nanozeolite clintoptilolite (nano Cu_2_O-CP) under mild aqueous conditions at rt in good to excellent yields.^128^ Specifically, 138 and 140 were obtained from the reaction of aldehyde (21a or 2) and malononitrile (29a) with 4-hydroxypyridine-2-ones (137a) and Kojic acid (139), respectively. The NPs were synthesized from nanozeolite, such as clintoptilolite, and copper chloride (CuCl_2_), and well characterized *via* XRD, BET, SEM, TEM, TEM-EDS, and XPS. The CuNPs yielded a better synthetic yield of the final product in comparison with NiNPs in water rather than other solvents such as ethanol, DMF, toluene, and DCM. In the absence of catalyst, only a trace amount of product was observed even after a prolonged reaction time. The reaction has the advantage that it can tolerate various electron-withdrawing and donating substituents under the optimized reaction conditions. The reusability of the catalyst was studied for up to eight catalytic cycles with high efficiency, which is attributed to the strong interaction of Cu_2_O NPs with the zeolite.

**Scheme 59:**
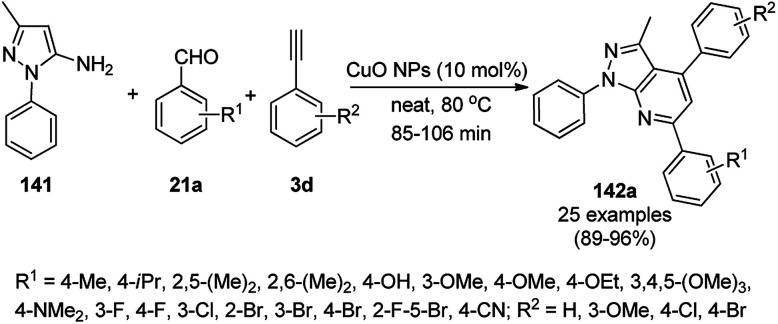
Synthesis of phenyl-1*H*-pyrazolo[3,4-*b*]pyridines (142a) catalyzed by CuO NPs.

Jeong *et al.* reported the commercially available CuO NP-catalyzed synthesis of phenyl-1*H*-pyrazolo[3,4-*b*]pyridines (142a) *via* the three-component domino reaction of 3-methyl-1-phenyl-1*H*-pyrazol-5-amine (141), substituted aryl carbaldehydes (21a), and aryl alkynes (3b) under solvent-free conditions at 80 °C in 89–96% yield (Scheme 59).^129^ During the course of the domino reaction, the CuO NPs act as a Lewis acid catalyst to promote the Diels–Alder reaction. The recyclability of the catalysts revealed that the catalyst could be recycled for up to four catalytic cycles without loss in its catalytic potential.

**Scheme 60:**
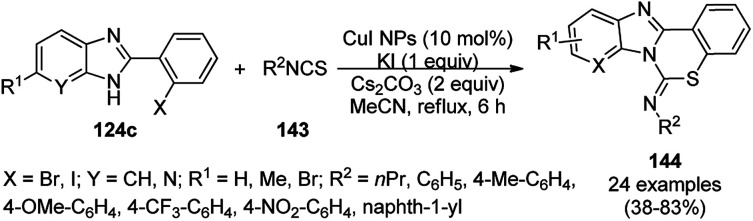
Synthesis of benzo[*e*]benzo[4,5]imidazo[1,2-*c*][1,3]thiazin-6-imines (144) using CuI NPs as the catalyst.

Wang *et al.* reported copper iodide nanocatalysts chelated to 1,10-phenanthroline for the synthesis of benzo[*e*]benzo[4,5]imidazo[1,2-*c*][1,3]thiazin-6-imines (144) from substituted 2-phenylbenzimidazoles (124c) and substituted isothiocyanates (143) using potassium iodide as the ligand, Cs_2_CO_3_ as the base and acetonitrile as the solvent under reflux (Scheme 60).^130^ The intramolecular S_N_Ar reaction to form the C(sp^2^)–S bond proceeds on the surface of nano copper iodide catalyst chelated to 1,10-phenanthroline to promote the oxidative addition and reductive elimination. A recycling experiment was performed to assess the caliber of the catalyst for reuse, where after the second run it was observed that the catalyst gave a yield of 76% of the final product.

**Scheme 61:**
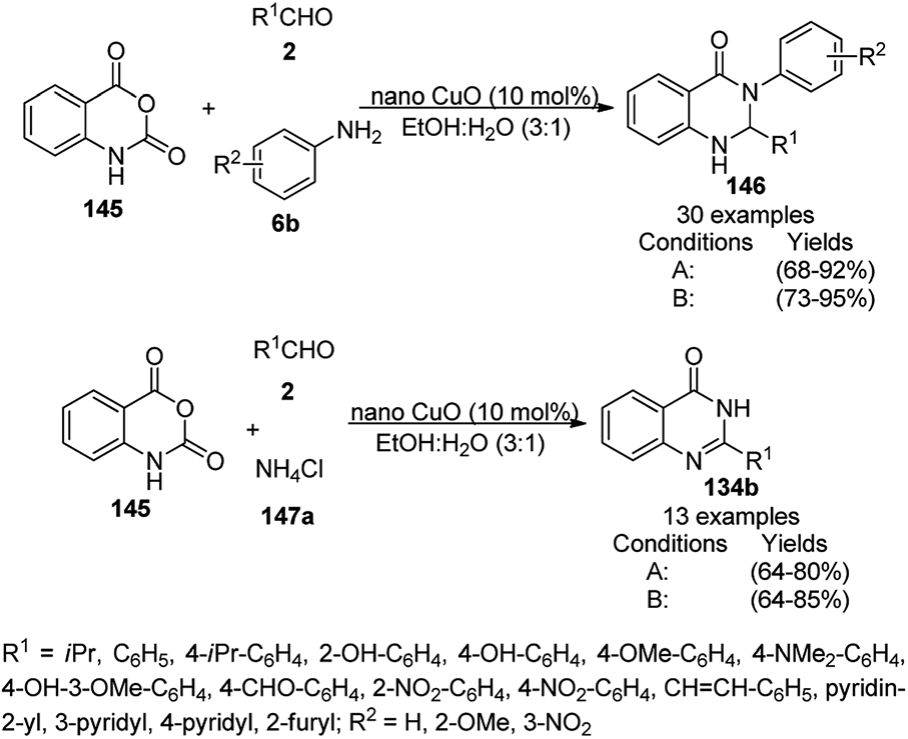
CuO NP-catalyzed synthesis of 2,3-dihydroquinazolin-4(1*H*)-ones (146) and quinazolin-4(3*H*)-ones (134b). Conditions A: reflux, 3 h; and conditions B: 60 °C, ultrasound, 10–30 min.

Zhang *et al.* reported the synthesis of 2,3-dihydroquinazolin-4(1*H*)-ones (146) and quinazolin-4(3*H*)-ones (134b) from isatoic anhydride (145), amine such as anilines (6b)/ammonium chloride (147a), and alkyl/aryl/heteroaryl aldehydes (2) catalyzed by nano CuO as a Lewis acid catalyst in aqueous ethanol (3 : 1) under reflux conditions (Scheme 61).^131^ They also synthesized all the compounds under ultrasonication and compared the yields of both reaction conditions. The developed protocol shows wide tolerance range for various functional groups. The recyclability of the catalysts was checked for up to four catalytic runs and the yields of the product was reported to range from 78–80% for the one-pot synthesis using 145, benzaldehyde and aniline.

**Scheme 62:**
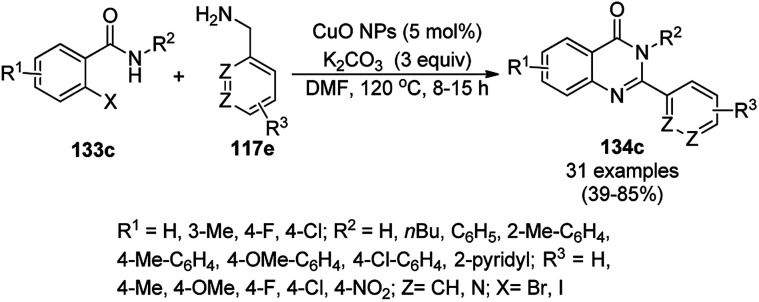
Synthesis of 2,3-disubstituted quinazolinones (134c) from 2-halobenzamides (133c) and arylalkyl amines (117e).

The CuO NP-catalyzed ligand-free and additive-free Ullmann coupling involving *N*-arylation followed by oxidative C–H amidation for the synthesis of 2,3-disubstituted quinazolinones was successfully achieved from 2-halobenzamides (133c) and arylalkyl amines (117e) in DMF (Scheme 62).^132^ The catalyst was separated by centrifugation and recycled successfully for up to three cycles for the synthesis of 134c in 39–85% yield. The decreased yield in the third cycle during reuses can be attributed to the agglomeration of the NPs during the course of the reaction (TEM).

**Scheme 63:**
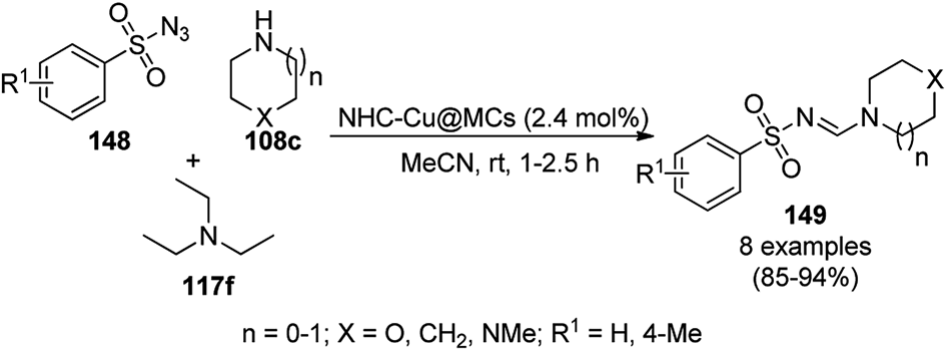
Three-component reaction for the synthesis of *N*-sulfonylformamidines (149) catalyzed by CuNPs.

Shahrisa *et al.* reported the synthesis of *N*-sulfonylformamidines (149) using an N-heterocyclic complex-copper complex supported on magnetic cellulose (NHC–Cu@MCs) from the three-component reaction among arylsulfonyl azide (148), secondary alicyclic amines (108c) such as morpholine, *N*-methylpiperazine, piperidine and pyrrolidine, and triethylamine (117f) in acetonitrile at rt in 85–94% yield (Scheme 63).^133^ The NCs were synthesized *via* the treatment of ionic liquid-grafted magnetic cellulose with copper iodide, potassium *tert*-butoxide in THF, and further well characterized *via* TGA, VSM, SEM, XRD, EDX, and FT-IR. The scope of the reaction was explored with aliphatic amines, but was found to be unsuccessful for aromatic amines. The recyclability of the catalyst recovered using an external magnet was demonstrated for up to five catalytic runs with the loss of 20% of final product at the end of the fifth catalytic run. Specifically, 149 acts as a methylene donor and oxidant for the conversion of Cu(ii) into Cu(i), which co-ordinates with the nitrogen of 149 for the elimination of diethylamine and 1,3-dipolar addition to proceed.

**Scheme 64:**
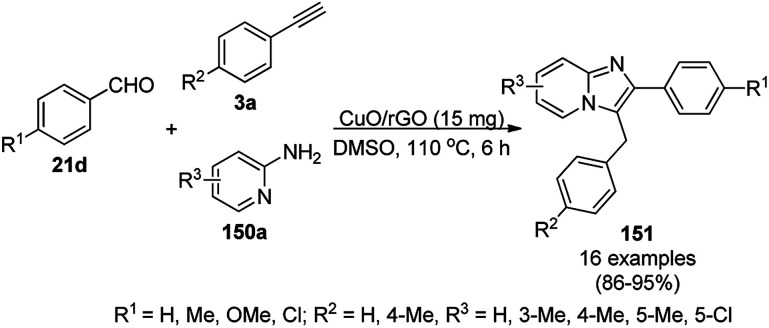
Synthesis of imidazo[1,2-*a*]pyridines (151) catalyzed by CuNPs.

CuO NPs immobilized on reduced graphene oxide sheets (CuO/rGO) catalyzed the cyclocondensation of 2-benzaldehydes (21d), phenyl acetylenes (3a) and aminopyridines (150a), providing an efficient approach for the synthesis of imidazo[1,2-*a*]pyridines (151, Scheme 64) without any additives.^134^ The Cu(ii) catalyst promoted Cu(ii)-mediated aminomethylation of terminal alkynes 3a to give the intermediate propargylamines. The present 2D-nano composites were synthesized according to previously reported protocols^135^ and found to be stable, as evident from the hot filtration tests and reuse of the catalyst for up to five times.

**Scheme 65:**
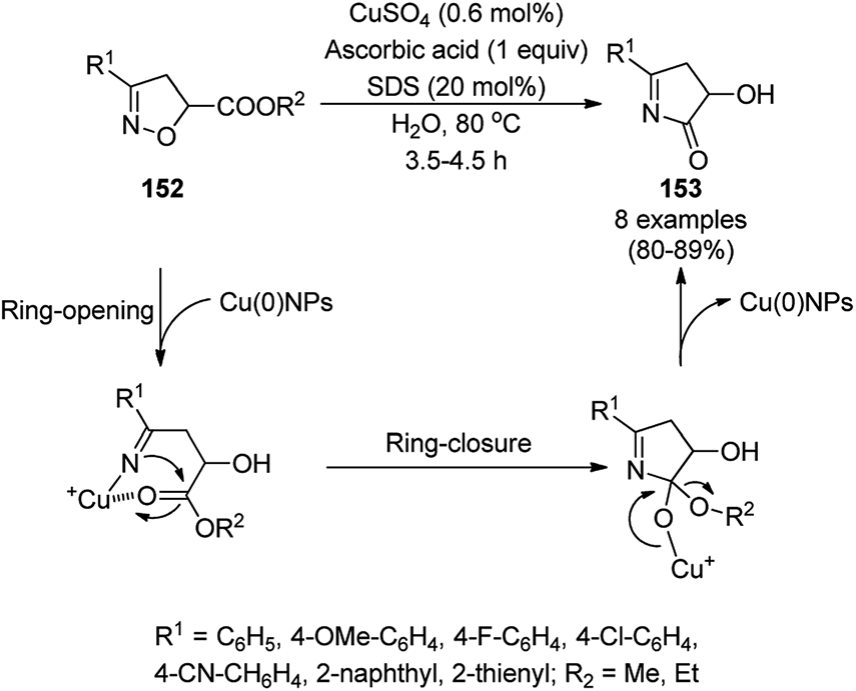
Cu(0)NP-catalyzed ring opening and domino cyclisation of *Δ*^2^-isoxazoline-5-esters (152).

Cu(0)NP-catalyzed ring opening followed by the domino or sequential cyclization (Scheme 65) of *Δ*^2^-isoxazoline-5-esters (152) resulted in the synthesis of novel γ-hydroxy pyrrolinone (153) in 80–89% yield.^136^ Maiti *et al.* has achieved this transformation using *in situ*-fabricated CuNPs generated from CuSO_4_·5H_2_O as the metal precursor, SDS (sodium dodecyl sulfonate) as the surfactant, and ascorbic acid as the reducing agent under aqueous conditions. The *in situ*-generated cooperative assemblies were characterized *via* SEM, UV-Vis, DLS, TEM and PXRD. However, the same Cu(0)NP-catalyzed protocol at 60 °C yielded ring-opened β-hydroxy ketones from 152 in 79–89% yield and carboxamides from carbonyl azides in 79–95% yield. A lowed efficiency was observed with the recovered Cu(0)NPs.

**Scheme 66:**
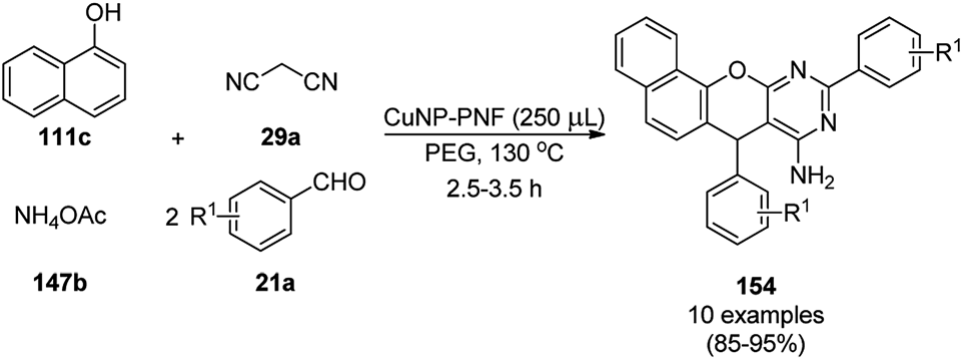
Synthesis of chromeno[2,3-*d*]pyrimidin-8-amines (154) catalyzed by CuNP-PNF.

**Scheme 67:**
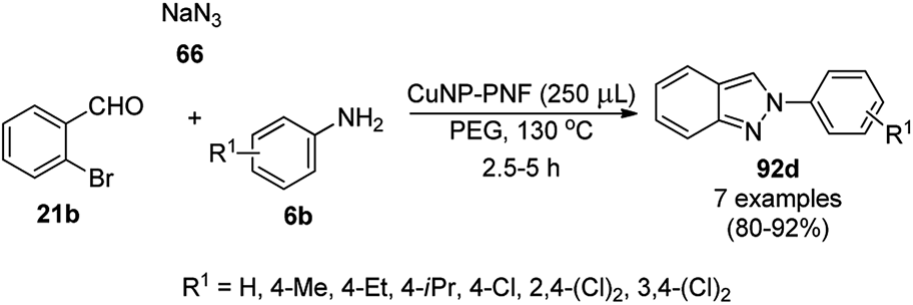
Synthesis of 2*H*-indazoles (92d) catalyzed by CuNP-PNF.

CuNPs decorated with peptide nanofibers (CuNP-PNF) catalyzed Knoevenagel condensation was reported for the synthesis of chromeno[2,3-*d*]pyrimidin-8-amines (154) from α-naphthol (111c), malononitrile (29a), ammonium acetate (147b) and benzaldehydes (21a) in polyethylene glycol (PEG, Scheme 66).^137^ The same protocol was also explored for the synthesis of 2*H*-indazoles (92d) from *o*-bromo benzaldehydes (21b), sodium azide (66) and substituted anilines (6b) in good to excellent yields (Scheme 67). PNF was synthesized *via* the self-assembly technique using histidine as a building block and CuCl as the metal precursor. CuNP-PNF played the key role in the synthesis of both heterocycles (154 and 92d) *via* co-ordination with the carbonyl oxygen of aldehydes (21a/b). The catalyst was recycled for up to four cycles for the Knoevenagel condensation of benzaldehyde, 111c, 29a and 147b.

**Scheme 68:**
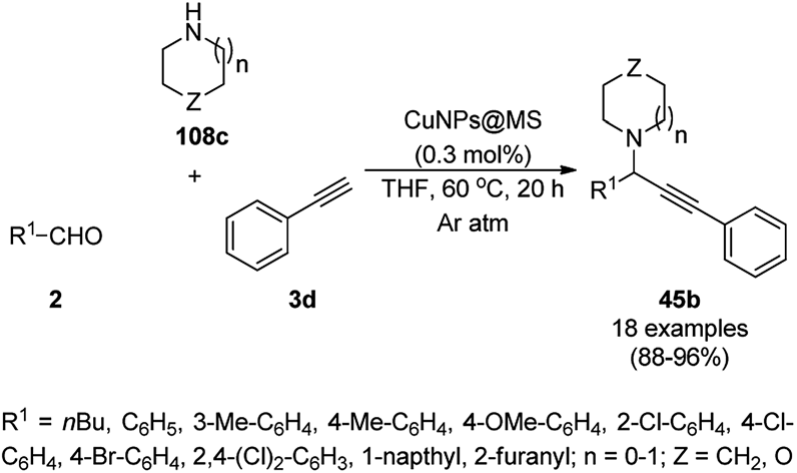
Synthesis of propargylamines (45b) catalyzed by CuNPs@MS.

Supported CuNPs on biodegradable starch microparticles (CuNPs@MS) catalyzed the A^3^ coupling of aldehydes (2), amines (108c) and alkynes (3d) for the synthesis of propargylic amines (45b) was reported by Gholinejad *et al.* (Scheme 68).^138^ The NCs were prepared *via* the treatment of previously prepared starch microparticles^139^ with Cu(OAc)_2_·H_2_O and sodium borohydride. The CuNPs could catalyze A^3^ coupling *via* the activation of 3d*via* the formation of copper acetylide. In the recycling experiment, at the end of the fifth run, a slight loss (10%) in the yield of the A^3^-coupled product of benzaldehyde, piperidine, and 3d was observed compared to that in the first catalytic run.

**Scheme 69:**
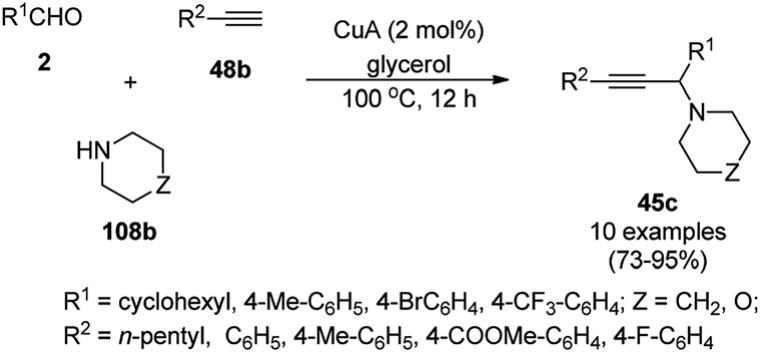
A^3^ coupling catalyzed by zero valent CuA.

Favier *et al.* reported the catalytic use of Cu(0)NPs (CuA) in glycerol for the synthesis of propargylic heterocyclic amines (45c) *via* the A^3^ coupling of terminal alkynes (48b), carbaldehydes (2) and amines (108b) such as morpholines and piperidines (Scheme 69).^140^ They synthesized the CuNPs *via* the decomposition of di-μ-hydroxobis[(*N*,*N*,*N*′,*N*′-tetramethylethylenediamine)copper(ii)]chloride [Cu(_2_-*N*,*N*,*N*′,*N*′-TMEDA)(μ-OH)]_2_Cl_2_ in glycerol in the presence of polyvinylpyrrolidone (PVP) as a stabilizer to get red-purple colloidal solutions. The multi-purpose use of the catalyst was proven by the formation of C–N bonds from haloarenes and amines, cross-dehydrogenative coupling of *tert*-amines with terminal alkynes, A^3^-cycloisomerisation-tandem reactions and ketone-aldehyde-alkyne (KA^2^) reaction. The catalytic phase containing the zero valent CuNPs was recycled for more than five times, preserving its catalytic potential.

**Scheme 70:**
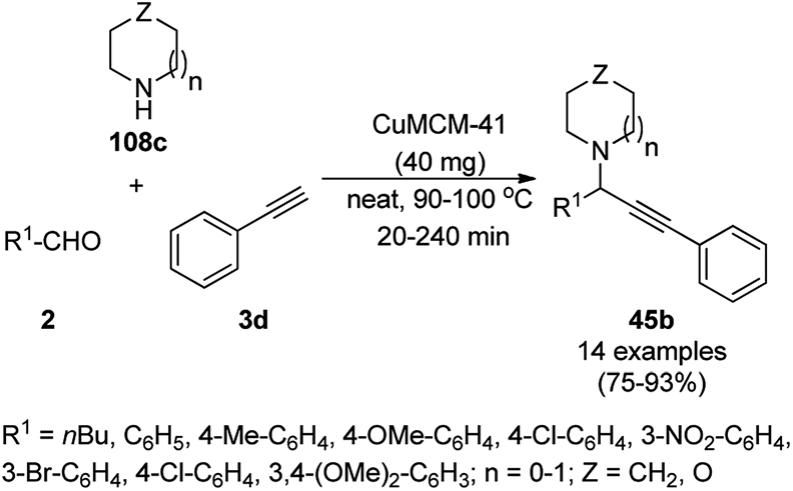
A^3^ coupling catalyzed by CuMCM-41.

Copper-modified spherical MCM-41 (CuMCM-41) NP-catalyzed A^3^ coupling for the synthesis of propargylamines (45b) from aldehydes, amines and phenyl acetylene (3d) was achieved under solvent-free conditions (Scheme 70) in good to excellent yields.^141^ The NCs were synthesized by the treatment of an aqueous solution of cetyltrimethyl bromide (CTAB) with tetraethyl orthosilicate (TEOS), copper acetate monohydrate and ammonia. The catalyst was recycled up to three times with a slight loss in catalytic performance due to the blockage of its active sites. The present protocol exhibits some advantages such as higher yield, shorted reaction time, moderate temperature, operational without inert atmosphere and solvent compared to the literature reports.^142–145^

**Scheme 71:**
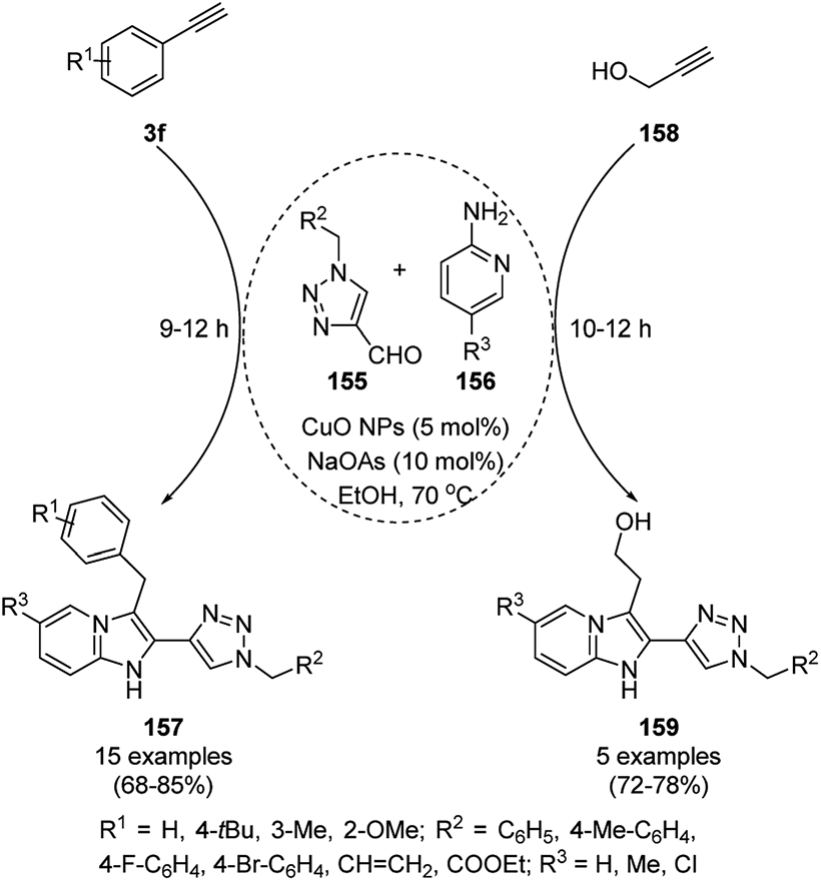
CuO NP-catalyzed synthesis of 2-triazolyl-imidazo[1,2-*a*]pyridines (157/159).

CuO NP-catalyzed A^3^ coupling-5-*exo*-dig cyclization for the successful synthesis of 2-triazolyl-imidazo[1,2-*a*]pyridines (157) from 1-alkyl-1,2,3-triazole-4-carbaldehyde (155), amidine (156) and phenyl acetylene (3f) was achieved by Khan *et al.* (Scheme 71) using sodium ascorbate as a reducing agent.^146^ The same CuO NP-catalyzed protocol was also explored for the synthesis of 2-(2-(1-alkyl-1,2,3-triazol-4-yl)-imidazo[1,2-*a*]pyridin-3-yl)ethanol (159) from propargylic alcohol (158). The NPs were reused for up to five times with moderate to good yields (68–82%).

**Scheme 72:**
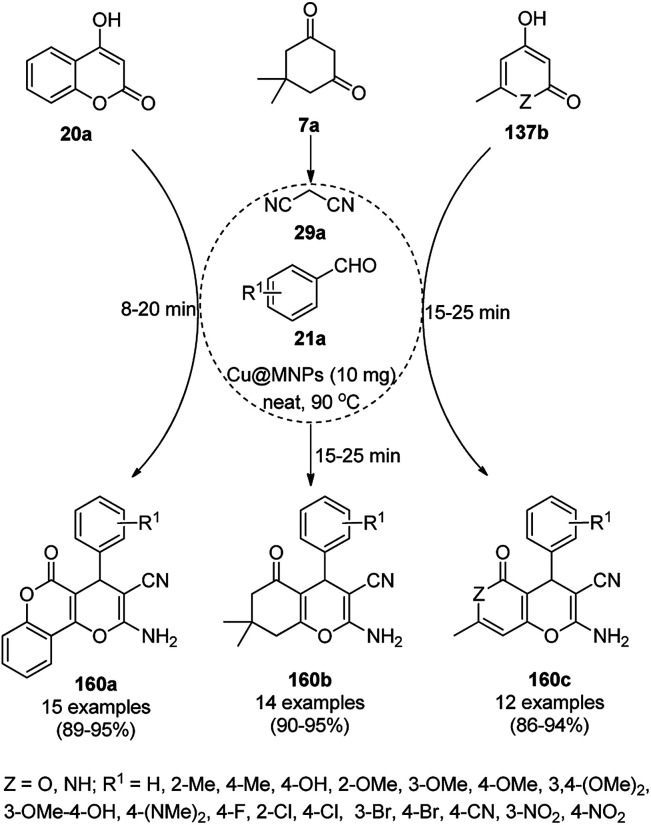
Synthesis of 2-amino-4*H*-chromenes (160a/b/c) catalyzed by Cu@MNPs.

The copper complexed magnetic NP (Cu@MNP)-catalyzed synthesis of 2-amino-3-cyno-4*H*-pyrans (160a/b/c, Scheme 72) was reported by Jimenez *et al. via* the Knoevenagel–Michael-cyclization of malononitrile (29a) and substituted benzaldehydes (21a) under solvent-free conditions with 4-hydroxy coumarins (20a), dimedone (7a) and C–H-activated acids (137b), respectively.^147^ The catalyst was recycled for up to five runs with negligible loss in its catalytic activity for the model reaction among 20a, 29a and 4-chlorobenzaldehyde. After coating of Fe_3_O_4_ NPs with TEOS, 3-chloropropyltriethoxysilane (CPTES), MNPs bonded with propyl chloride were obtained, which were subsequently treated with diethylenetriamine, piperidine, and [Cu(salal)_2_] to obtain a salicylic-chelated ligand, and the final NPs were characterized *via* FTIR, TGA, VSM, EDX and XRD. The present protocol (Scheme 72) was claimed to be superior in terms of shorter reaction times, lower catalytic loading, and avoiding toxic organic solvents and tedious separation protocols compared with literature reports for the synthesis of 160a (R^1^ = 4-Cl) catalyzed by CuO NPs,^148^ dendrimer core of oxo-vanadium phthalocyanine MNPs (MNP@AVOPc),^149^ urea,^150^ potassium phthalimide-*N*-oxyl (POPINO),^151^ SiO_2_ NPs,^152^ t-ZrO_2_ NPs,^153^ and ZnO NPs.^154^

**Scheme 73:**
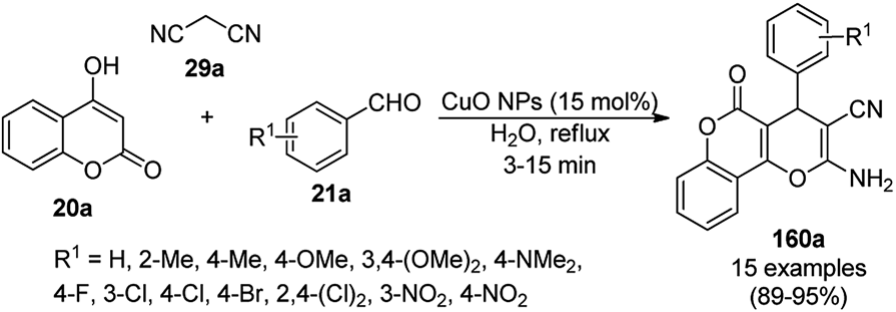
CuO NP-catalyzed synthesis of 3,4-dihydropyrano[*c*]chromenes (160a).

The CuO NP-catalyzed condensation of three components such as 4-hydroxycoumarin (20a), malononitrile (29a) and benzaldehydes (21a) was reported by Mehrabi *et al.* for the rapid synthesis of 3,4-dihydropyrano[*c*]chromenes (160a, Scheme 73).^148^ The NPs were synthesized *via* the sonochemical treatment of an alkaline solution of Cu(CH_3_COO)_2_·2H_2_O and polyvinyl alcohol (PVA). The products were purified *via* recrystallization, avoiding tedious chromatography.

**Scheme 74:**
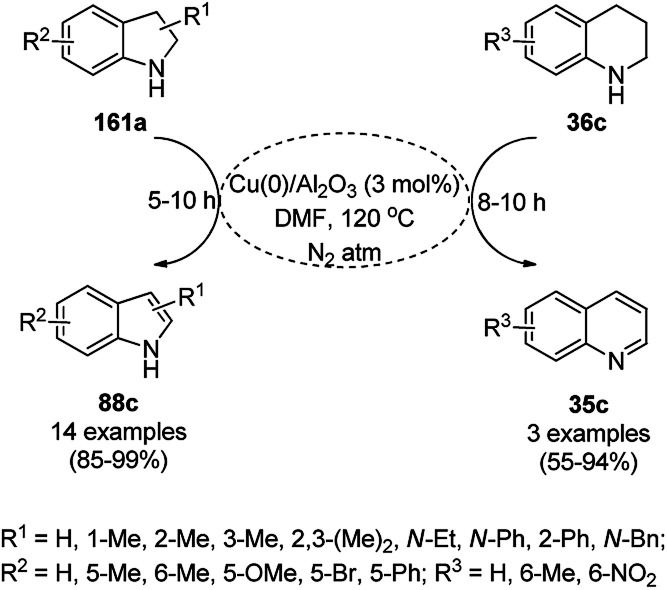
Dehydrogenation of indolines (161a) and 1,2,3,4-tetrahydroquinolines (36c).

CuNPs from copper aluminium hydrotalcite (Cu/Al_2_O_3_) catalyzed the dehydrogenation of indolines (161a) and 1,2,3,4-tetrahydroquinolines (36c) in the report by Likhar *et al.* (Scheme 74).^155^ They have also reported the use of the same catalyst for the dehydrogenation of diverse amines and alcohols with significantly high turnover numbers (TON) and turnover frequency (TOF). The CuNPs were obtained *via* the co-precipitation of copper nitrate and aluminium nitrate followed by calcination at 473 K and chemical reduction in a Parr hydrogenator. The catalyst was reused with a regular catalytic performance during five consecutive runs.

### 3.4 CoNP-catalyzed synthesis of heterocycles

**Scheme 75:**
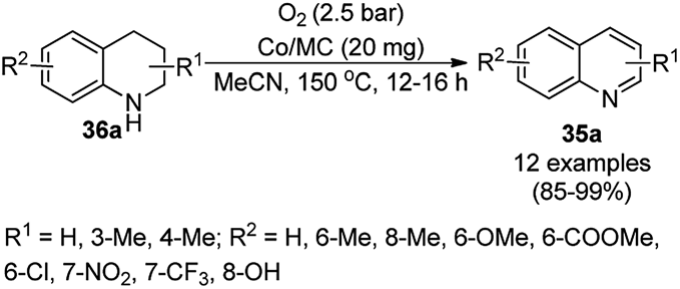
Aerobic dehydrogenation of 1,2,3,4-tetrahydroquinolines (36a) catalyzed by nitrogen-doped carbon-supported CoNPs.

Zhang *et al.* recently reported the oxidative dehydrogenation of tetrahydroquinolines (36a) using nitrogen-doped carbon-supported cobalt nanoparticles (Co/MC) in acetonitrile at 150 °C under 2.5 bar O_2_ in 85–99% yield (Scheme 75).^156^ The catalyst was prepared from 2,4-dihydroxybenzoic acid, hexamethylenetetramine, melamine, Pluronic P123 and 1,6-hexanediamine, which were dispersed in ammonium hydroxide solution having Co(NO_3_)_2_·6H_2_O. The final catalyst was well characterized *via* TEM, XRD, XPS, ICP-AES, Raman spectroscopy, nitrogen physisorption measurements, and electron paramagnetic resonance (EPR). The oxidative dehydrogenation of tetrahydroquinolines with electron-donating groups was found to have higher activity than that with electron-withdrawing groups. The catalyst played a vital role *via* the formation of the radical oxyanion (˙O_2_^−^), which became instrumental species for the aromatic oxidation. The present sustainable and economical approach exhibits the advantages of high catalytic potential, operational without additives, and sufficiently stability and recyclability for up to five reuses in comparison with reported protocols.^157–159^

**Scheme 76:**
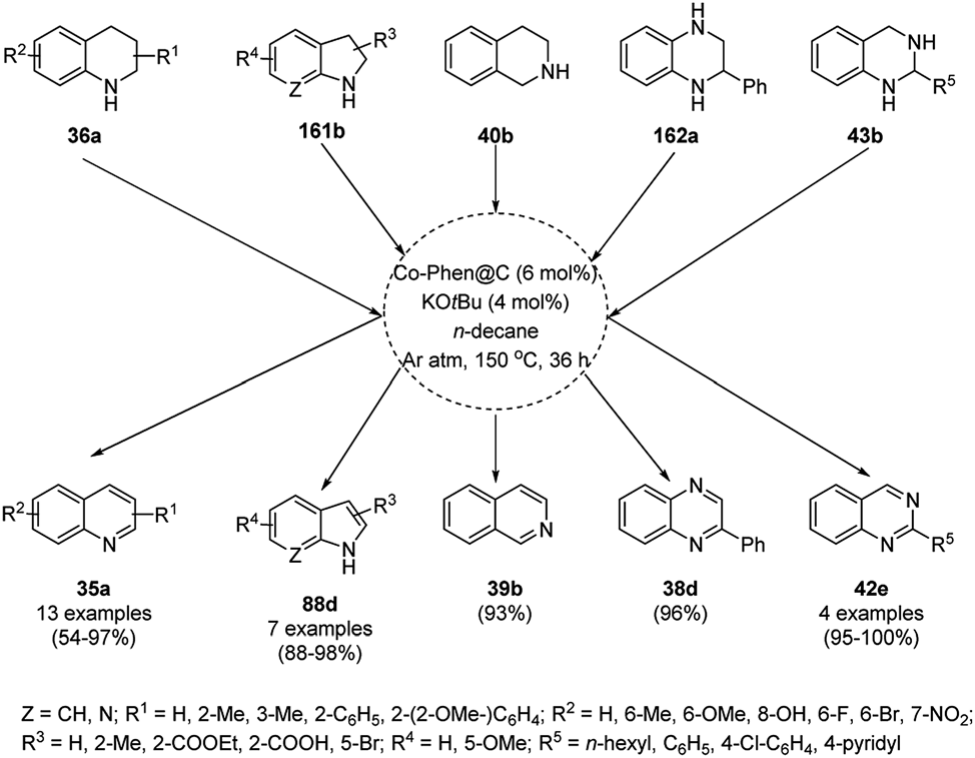
Dehydrogenation of aza-heterocycles catalyzed by Co-Phen@C.

**Scheme 77:**
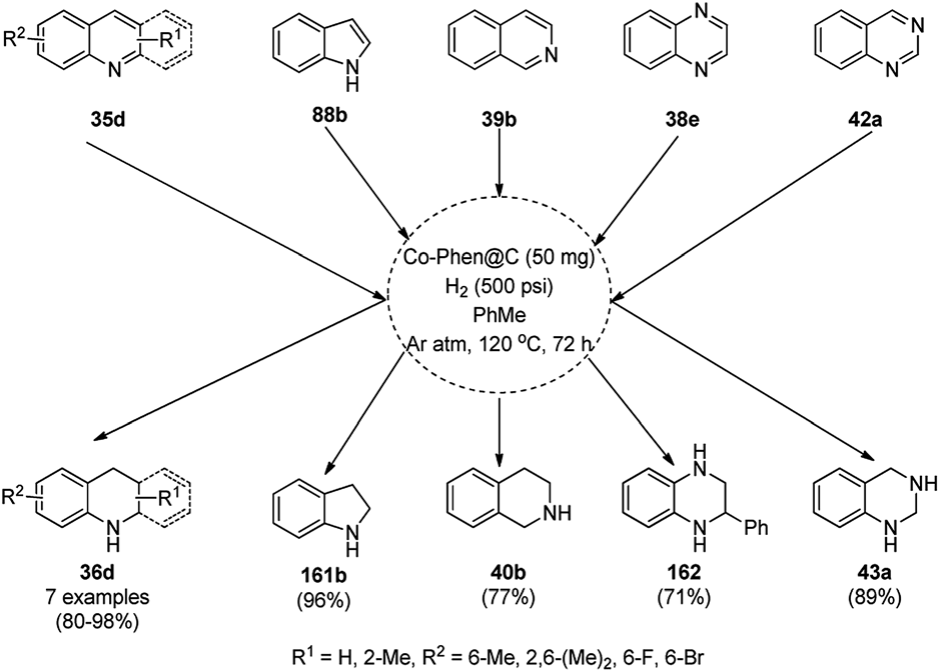
Hydrogenation of aza-heteroaromatic compounds catalyzed by Co-Phen@C.

Balaraman *et al.* reported a Co-phenanthroline complex adsorbed on GO (Co-Phen@C) as a NC in the acceptorless dehydrogenation of saturated aza-heterocycles to N-heteroaromatics for the synthesis of quinoline (35a), indoles (88d), isoquinolines (39b), quinoxalines (38d), and quinazolines (42e) using potassium *tert*-butoxide as the base (Scheme 76).^160^ They synthesized the required catalyst *via* the sonochemical treatment of Co(ii) acetylacetonate and 1,10-phenanthroline to obtain a Co-phenanthroline complex followed by its adsorption on exfoliated GO. The robustness of the catalyst was studied in a recycling experiment, where it was recycled up to six cycles without any decay in the yield of N-heteroaromatics. The complete dehydrogenation of the partially dehydrogenated heterocycles to N-heteroaromatics revealed that the reaction proceeded through partial dehydrogenation followed by isomerization and complete dehydrogenation. Further, the use of the same catalyst was also explored for the hydrogenation of N-heteroaromatics (Scheme 77) in toluene at 120 °C.

**Scheme 78:**
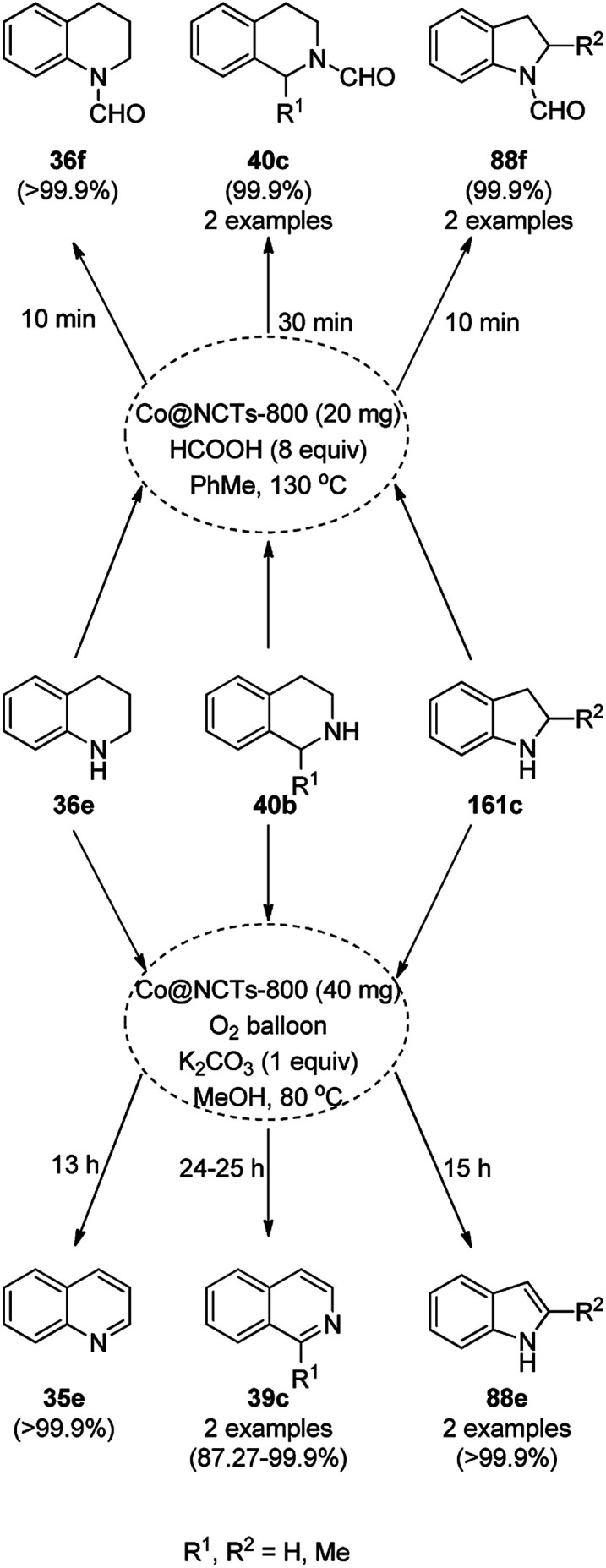
Oxidative dehydrogenation of heterocycles (36e/40b/161c) and their formylation catalyzed by Co@NCTs-800. % Conversion is summarized in parentheses.

**Scheme 79:**
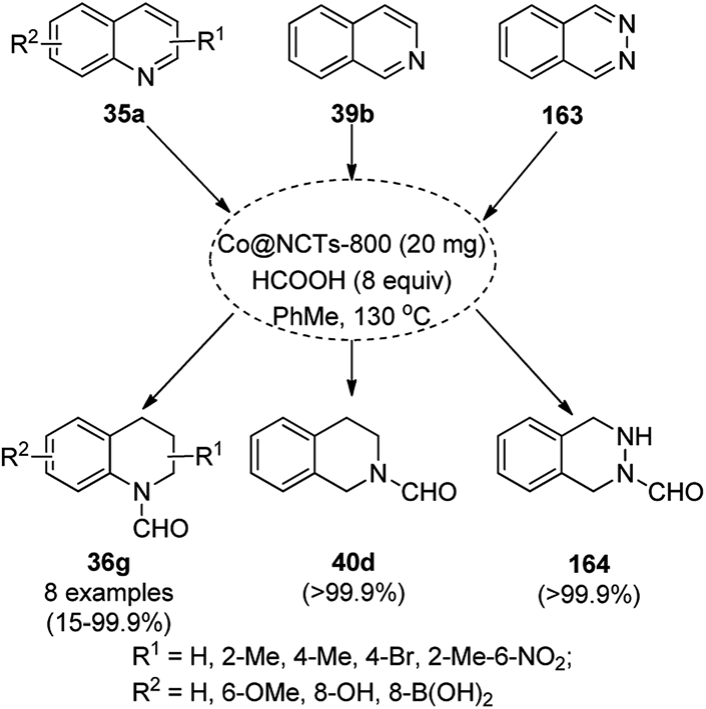
Oxidative dehydrogenation of N-heteroaromatics. % Conversion is summarized in parentheses.

CoNPs encapsulated in nitrogen-doped CNTs Co@NCTs-800 catalyzed the oxidative dehydrogenation of partially saturated heterocycles such as 1,2,3,4-tetrahydro quinolines (36e), 1,2,3,4-tetrahydroisoquinoline (40b), and 2,3-dihydroindole (161c, Scheme 78) in excellent conversions into 35e/39b/88e, respectively, with methanol as the solvent and potassium carbonate as the base.^161^ The fine tuning of the reaction conditions in toluene using the same catalyst and higher equivalents of formic acid also enabled the formylation of 36e/40b/161c (Scheme 78) in excellent conversions into 36f/40c/88f, respectively. Under the latter conditions, the catalytic transfer hydrogenation of heteroaromatics such as quinolines (35a), isoquinoline (39c), and phthalazine (163) (Scheme 79) was achieved successfully. The required catalysts were synthesized from dicyanodiamide and cobalt(ii) acetylacetonate and their treatment at various temperatures under inert atmosphere. The recyclability of the catalyst was demonstrated for up to ten cycles without loss in its catalytic activity.

**Scheme 80:**
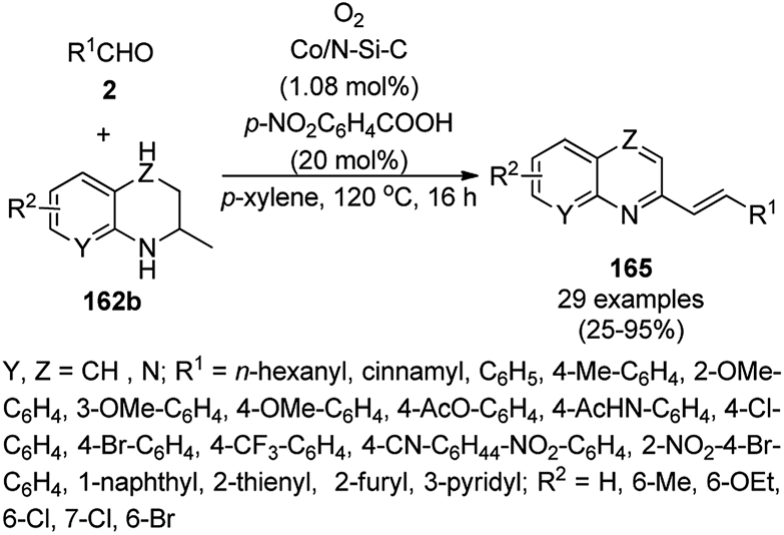
Dehydrogenative coupling of amines with aldehydes catalyzed by CoNCs.

Cobalt nanocatalysts (CoNCs) supported on nitrogen-silica-doped carbon (Co/N–Si–C) catalyzed the dehydrogenative coupling of carbaldehydes (2) with cyclic amines (162b) for the synthesis of quinolines/quinoxalines (165) in the report by Zhang *et al.* using molecular oxygen as the oxidant and *p*-nitro benzoic acid as the additive (Scheme 80).^162^ The CoNCs were prepared from Co(OAc)_2_·4H_2_O and 1,10-phenanthroline followed by their treatment with TEOS and loading on the support. The recycled catalyst was reused six times for the model reaction between benzaldehyde and 2-methyl-1,2,3,4-tetrahydroquinoline, where the fresh and reused catalysts possessed almost similar particle sizes of 0.35 nm and 0.42, respectively (TEM). The controlled experiments eliminated the probability of the dehydrogenation of 2 to 2-methylquinolines following the coupling of amines to support the actual mechanism, which is partial dehydrogenation followed by coupling and complete dehydrogenation to finally give 165.

**Scheme 81:**
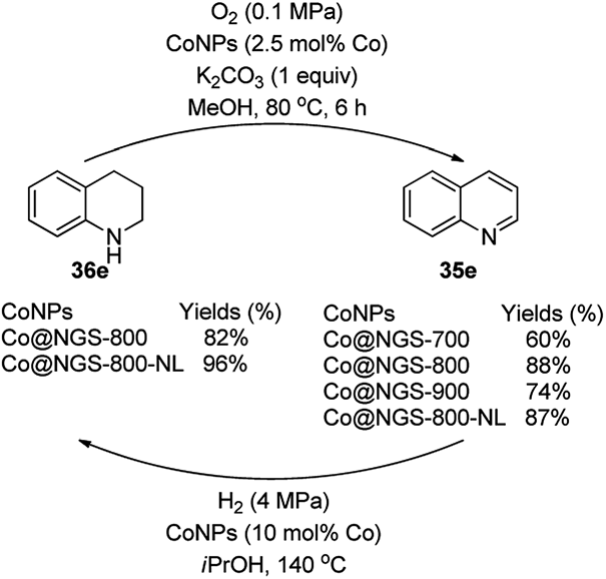
Dehydrogenation and hydrogenation catalyzed by Co@NPGS.

Li *et al.* reported the use of CoNPs supported on *N*-doped graphene cells (Co@NGS-800) as a bifunctional catalyst for the dehydrogenation of 1,2,3,4-tetrahydroquinoline (36e) and hydrogenation of quinoline (35e) in excellent conversion and high activity (Scheme 81).^163^ The same organic transformations were studied using various CoNPs encapsulated in *N*-doped graphene cells (Co@NGS-700, Co@NGS-800, and Co@NGS-900) obtained by pyrolysis at 700 °C, 800 °C and 900 °C, respectively. The Co@NGS-800 catalyst was recycled twelve and six times for the dehydrogenation of 36e, and hydrogenation of 35e, respectively, without loss in selectivity.

**Scheme 82:**
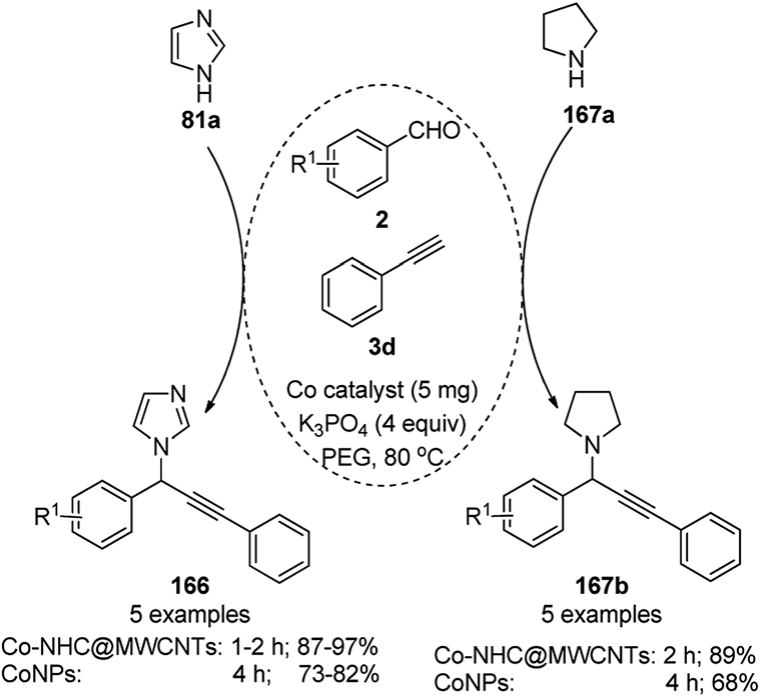
Synthesis of propargylamines catalyzed by CoNPs.

CoNPs and cobalt N-heterocyclic carbene grafted on multi-walled CNTs (Co–NHC@MWCNTs) catalyzed the synthesis of propargylamines (166/167b) from benzaldehydes (2), alkynes (3d) and amines (81a/167a) successfully (Scheme 82).^164^ Hajipour *et al.* synthesized the catalyst by grafting 1,4-diaminobenzene to obtain aniline@MWCNTs. Subsequently, it was treated with aniline, formaldehyde, and glyoxal to obtain an imidazolium salt followed by the loading of Co using CoCl_2_ to obtain the final NPs. The CoNPs were prepared *via* the neutralization of copper sulphate in aqueous NaOH to obtain a black powder. Greater yields of 166 and 167b were obtained in a shorter time with Co–NHC@MWCNTs than CoNPs. The present methodology was compared with other reported methods for the three-component reaction involving benzaldehyde, pyrrolidine, phenylacetylene catalyzed by nano Ag_2_O,^165^ Fe_2_O_3_,^166^ and CuNPs,^167^ and it was found that this method is the most suitable in terms of catalytic loading, reaction temperature and time and yield of 167b.

**Scheme 83:**
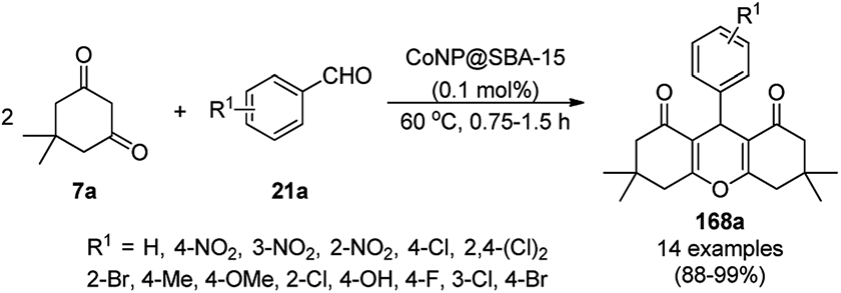
Synthesis of 1,8-dioxo-octahydroxanthenes (168a).

The synthesis of 1,8-dioxo-octahydroxanthenes was achieved using CoNPs supported on silica (CoNP@SBA-15)^168^ as a Lewis acid catalyst *via* the cyclocondensation of dimedone (7a) and benzaldehydes (21a) under aqueous conditions (Scheme 83).^169^ The stability of the CoNPs was established by their ten consecutive reuses for the reaction between 7 and benzaldehyde to obtain 168a in 88–99% yield. The said protocol was also compared with other reported protocols such as nano-TiO_2_,^170^ CuS quantum dots,^171^ and FeNPs.^172^

**Scheme 84:**
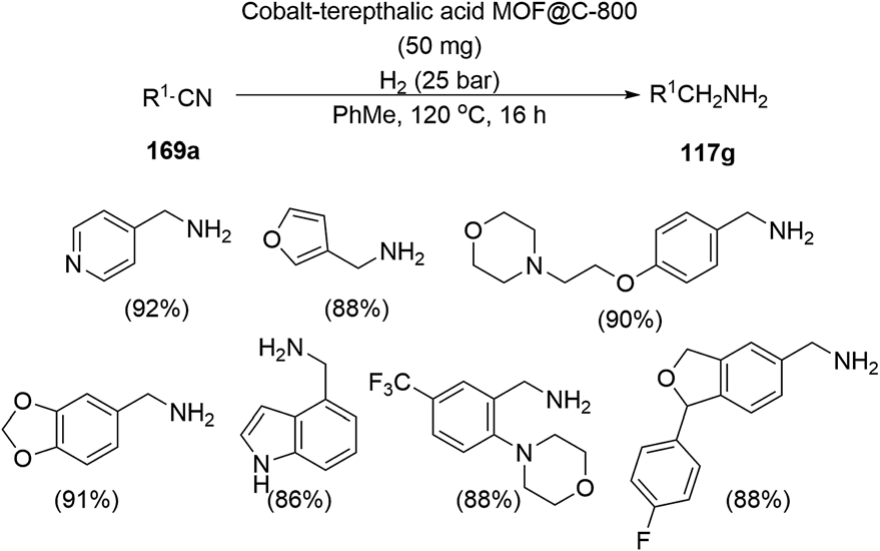
Synthesis of methylamines (117g) from cyanides (169a) catalyzed by CoNPs.

**Scheme 85:**
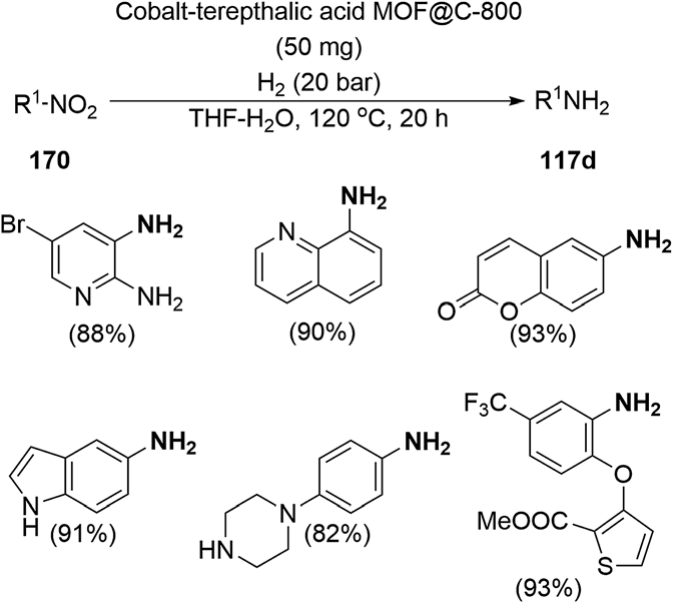
CoNP-catalyzed reduction of nitro-containing heterocycles (170) to aromatic amines (117d).

A CoNP-based NC (cobalt-terephthalic acid MOF@C-800) were reported for the hydrogenation of cyanide (169a) to primary amine (117g) in toluene at 120 °C (Scheme 84).^173^ This catalyst was synthesized *via* the treatment of cobalt(ii) nitrate hexahydrate with terephthalic acid *via* a solvothermal process. It was also used for the conversion of nitro-heteroaromatics (170) to heteroaryl amines (117d, Scheme 85). The stability of the catalyst was proven by its scaling on a multigram scale and reuse for up to five runs.

**Scheme 86:**
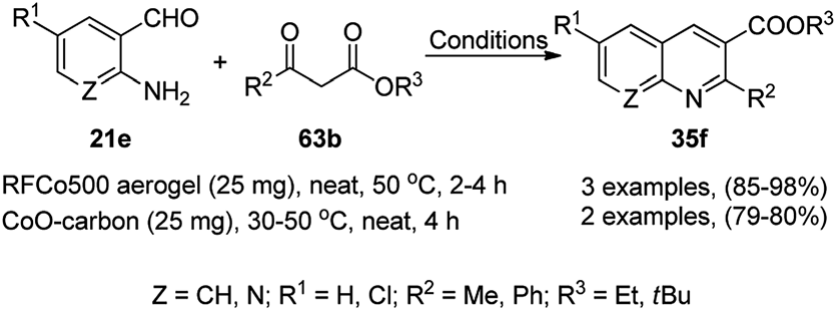
Friedländer annulation catalyzed by CoNPs catalyzed by RFCo500 aerogel and CoO-carbon.

The cobalt(0)-doped carbon aerogel (RFCo500 aerogel)-catalyzed Friedländer annulation for the synthesis of quinolines (35f) from 2-amino-5-chlorobenzaldehyde (21e) and β-keto esters (63b) was achieved by Pérez-Mayoral (Scheme 86) under solvent-free conditions in good to excellent yields.^174^ The RFCo500 catalyst was synthesized from resorcinol, formaldehyde, organic aerogels and cobalt acetate. The catalyst was recycled for up to three runs with 20% loss in its catalytic activity compared to the first run. Another application of cobalt oxide NPs supported on different carbon supports was reported by Pérez-Mayoral *et al.* (Scheme 86) for the synthesis of quinolines (35f) using similar starting materials.^175^ CoO-carbon was synthesized *via* the treatment of cobalt nitrate or acetate with a carbon support.

### 3.5 FeNP-catalyzed synthesis of heterocycles

**Scheme 87:**
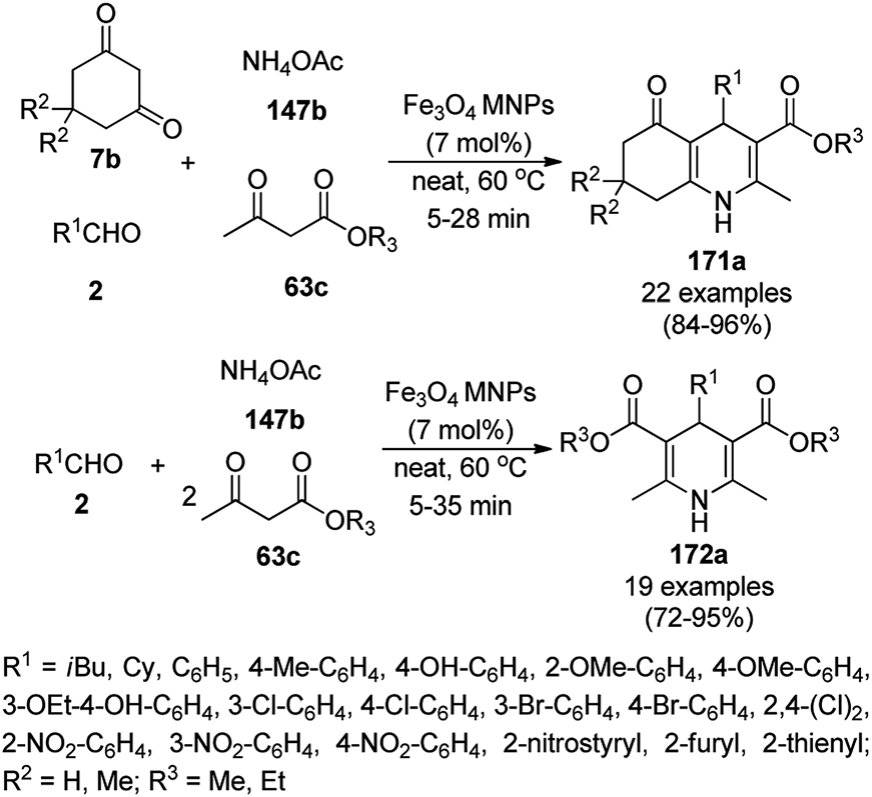
Fe_3_O_4_ MNP-catalyzed synthesis of polyhydroquinolines (171a) and 1,4-dihydropyrimidines (172).

Magnetically retrievable FeNPs have been reported for the synthesis of various bioactive heterocycles^176^ and several organic transformations such as hydrogenation, dehydrogenation, Friedel–Crafts reactions, C–C bond formation and borylation.^177^ Fe_3_O_4_ MNP-catalyzed Knoevenagel–Michael-cyclization for the synthesis of polyhydroquinolines (171a) was successfully achieved by Nasr-Esfahani *et al.* using cyclic diones (7b), carbaldehydes (2), alkyl acetoacetate (63c) and ammonium acetate (147b, Scheme 87) under solvent-free conditions.^178^ The same protocol was also explored for the synthesis of 1,4-dihydropyrimidines (172a) from 2, 63c and 147b. The MNPs were prepared *via* the treatment of ferrous and ferric salts in the presence of ammonium hydroxide to obtain black-colored Fe_3_O_4_ MNPs. The catalyst was recycled up to five times without loss in its catalytic efficiency. Two years later, they also reported the applications of modified magnetic acidic NCs for the synthesis of bulky heterocyclic compounds.^179^ Recently, they also reported the synthesis of 1,4-dihydropyrano[2,3-*c*]pyrazoles using Fe_3_O_4_@SiO_2_ NPs grafted on nanobentonite and functionalized with organic and inorganic linkers and sulfonic acids in aqueous ethanol.^180^

**Scheme 88:**
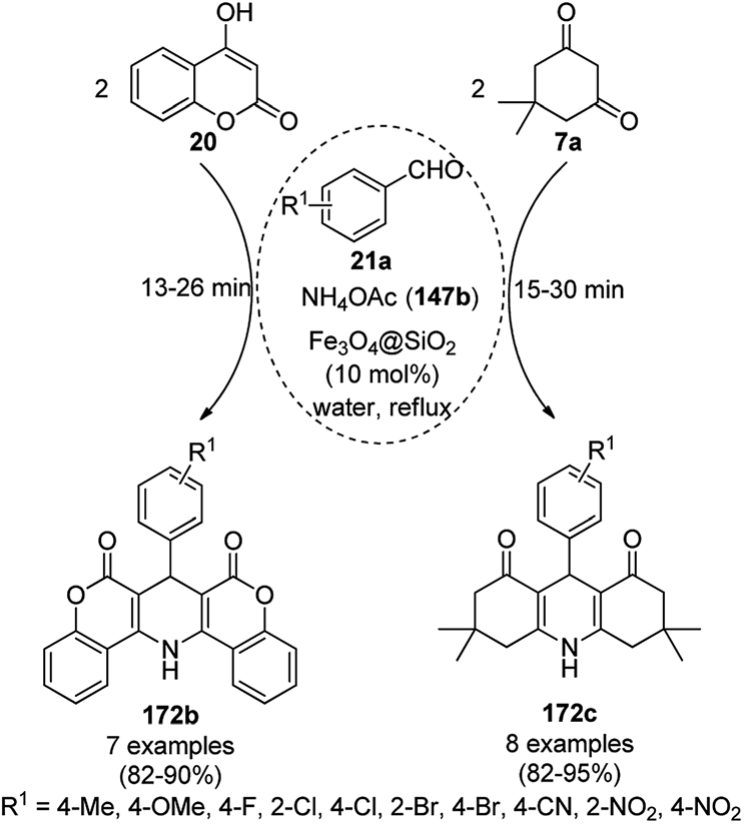
“On water” chemistry for the synthesis of 1,4-dihydropyrimidines (172b/c).

The Knoevenagel–Michael-cyclization *via* green “on water” chemistry for the one-pot synthesis of 1,4-dihydropyrimidines (172b/c) was successfully achieved using Fe_3_O_4_@SiO_2_ MNPs, which were synthesized by loading silica on Fe_3_O_4_ NPs (Scheme 88).^181^ The air- and moisture-stable catalysts were separated using an external magnet and reused up to five times with 85–92% yield.

**Scheme 89:**
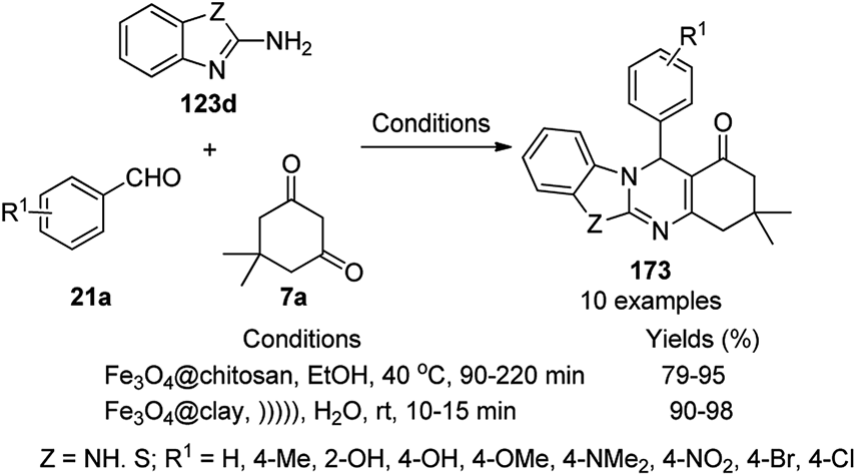
Iron oxide NP-catalyzed synthesis of imidazo or thiazolopyrimidines (173).

In 2015, Maleki and co-workers reported Fe_3_O_4_ NPs supported on chitosan (Fe_3_O_4_@chitosan), which were prepared *via* the sol–gel method, for the Knoevenagel–Michael-cyclization green synthesis of tetracyclic benzimidazo or benzothiazolopyrimidines (173) *via* the condensation of 2-aminobenzimidazole or 2-aminobenzothiazole (123d), substituted benzaldehydes (21a) and dimedone (7a).^182^ The next year in 2016, the same research group reported for the first time the catalytic use of Fe_3_O_4_@clay for the rapid synthesis of 173 in water at rt (Scheme 89) under ultrasonic conditions with high yield.^183^ The ultrasonic treatment of a mixture of ferrous and ferric salts with a solution of clay in ammonium nanoclay yielded Fe_3_O_4_@clay NPs. The catalytic potential of the NPs was realized when individual clay or Fe_3_O_4_ or ultrasonic treatment could not yield 173 in good yield. Only 7% loss in yield was noted at the end of the sixth cycle when recycling Fe_3_O_4_@clay compared to the first run. In the same year, following a similar approach, the synthesis of 173 was reported by Javanshir *et al.* using Fe_3_O_4_ NPs as a green catalyst, which were prepared using Irish moss (red edible algae) in ethanol under reflux.^184^

**Scheme 90:**
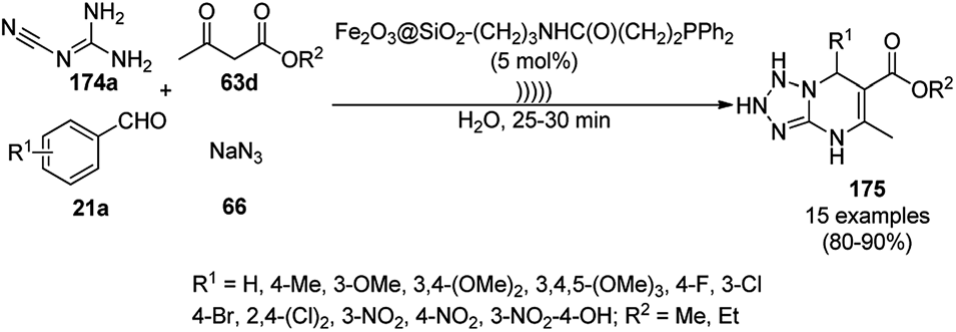
Synthesis of tetrazolopyrimidines (175).

Maleki *et al.* also reported the multi-component green synthesis of tetrazolopyrimidines (175) from cyano-guanidine (174a), 66, substituted benzaldehyde (21a), and methyl/ethyl acetoacetate (63d) catalyzed by Fe_2_O_3_@SiO_2_-(CH_2_)_3_NHC(O)(CH_2_)_2_PPh_2_ in water under ultrasonication in good to excellent yields (Scheme 90).^185^ The Fe_3_O_4_ NPs were prepared using FeCl_2_ and FeCl_3_ in aqueous ammonia and oleic acid, which were further reacted with ((3-aminopropyl)triethoxysilane) APTMS and 3-(diphenylphosphine)propionic acid to obtain the final NPs. The structural analysis of the NPs were performed *via* FE-SEM, EDX, TEM, TGA, DTG, and FT-IR. The NPs were recycled for six consecutive cycles with significant yields of the final product. The catalyst promoted Knoevenagel condensation followed by Michael addition by activating the carbonyl group of the ketone/aldehyde substrate.

**Scheme 91:**
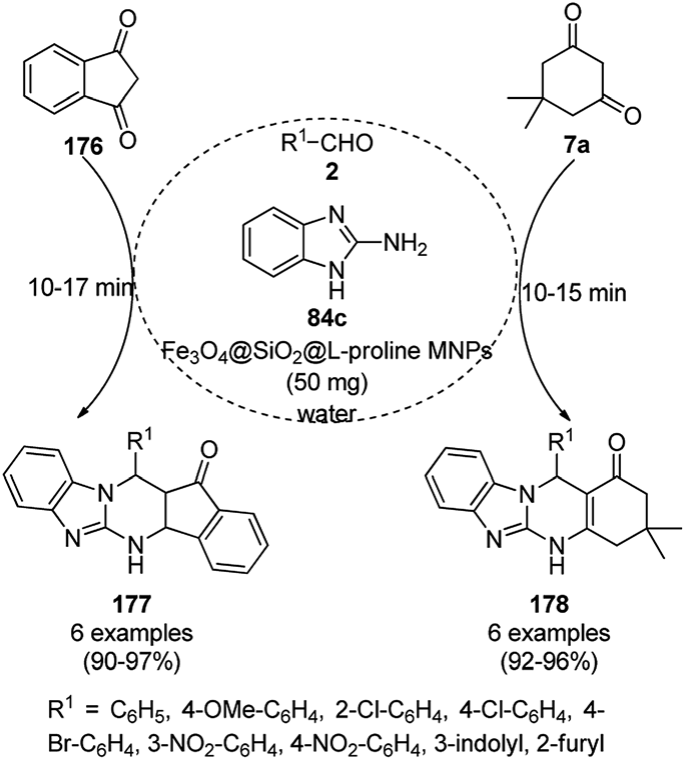
Synthesis of benzoimidazo[1,2-*a*]pyrimidines (177) and tetrahydrobenzo[4,5]imidazo [1,2-*d*]quinazolin-1(2*H*)-ones (178).

Fe_3_O_4_@SiO_2_ NPs functionalized with l-proline (Fe_3_O_4_@SiO_2_@l-proline) catalyzed the synthesis of benzoimidazo[1,2-*a*]pyrimidines (177) and tetrahydrobenzo [4,5]imidazo[1,2-*d*]quinazolin-1(2*H*)-ones (178) (Scheme 91).^186^ The treatment of Fe_3_O_4_@SiO_2_ NPs with l-proline in toluene under reflux provided the final Fe_3_O_4_@SiO_2_@l-proline NPs, which were recycled up to ten times. The Gram-scale applicability of this reaction was assessed successfully with 4-nitrobenzaldehyde and 176 and 84c.

**Scheme 92:**
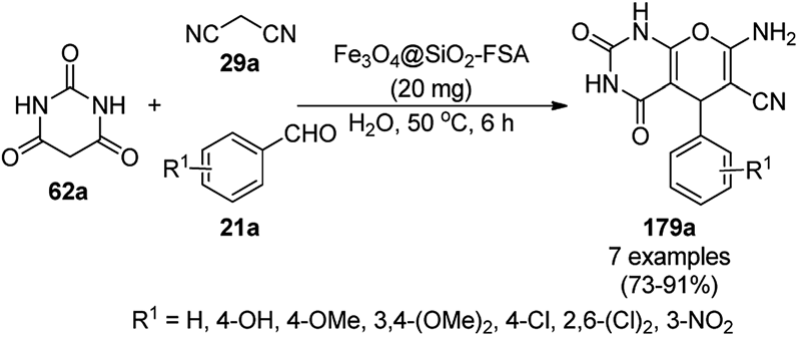
Knoevenagel–Michael-cyclization of barbituric acid (62), malononitrile (29) and substituted benzaldehydes (21a).

Similar to the former approach (Scheme 89), the Knoevenagel–Michael-cyclization of barbituric acid (62a), malononitrile (29a) and substituted benzaldehydes (21a) was reported by Heydari and co-workers (Scheme 92)^187^ using silica-coated Fe_3_O_4_ NPs tagged with formamidine sulfonic acid (Fe_3_O_4_@SiO_2_-FSA). The catalyst was recycled up to eight times following the principles of green chemistry. The NCs were obtained *via* the successive treatment of Fe_3_O_4_@SiO_2_ NPs with sulfonyl chloride and formamidine sulfonic acid.

**Scheme 93:**
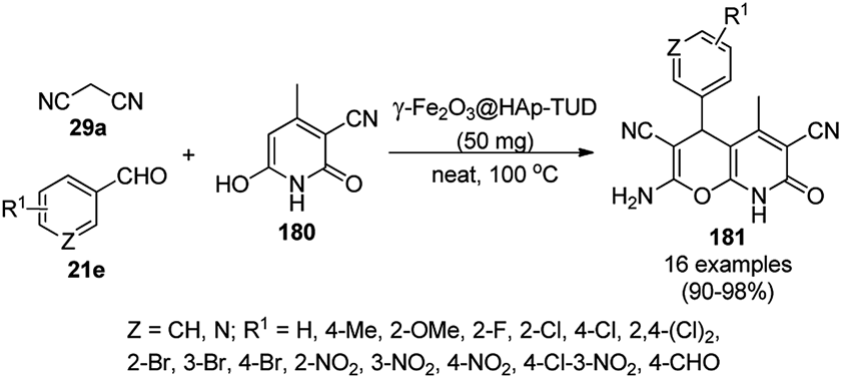
Synthesis of pyrano[2,3-*b*]pyridines (181) catalyzed by γ-Fe_2_O_3_/Hap MNPs.

Thiourea dioxide-decorated hydroxyapatite-supported γ-Fe_2_O_3_ NP (γ-Fe_2_O_3_@HAp-TUD)-catalyzed Knoevenagel–Michael-cyclization for the synthesis of pyranopyridines (181) was achieved successfully *via* the one-pot three-component reaction of malononitrile (29a), substituted benzaldehydes (21e) and 3-cyano-6-hydroxy-4-methyl-pyridin-2(1*H*)-ones (180) under solvent-free conditions (Scheme 93).^188^ The typical process for the preparation of NPs involved the coating of hydroxyapatite on Fe_2_O_3_ NPs followed by functionalization with hexamethylene-1,6-diisocyanate and thiourea dioxide. The present catalyst offered the advantages of high yields in shorter reaction times, avoiding the use of solvent compared other thiourea-catalyzed protocols.^189–191^

**Scheme 94:**
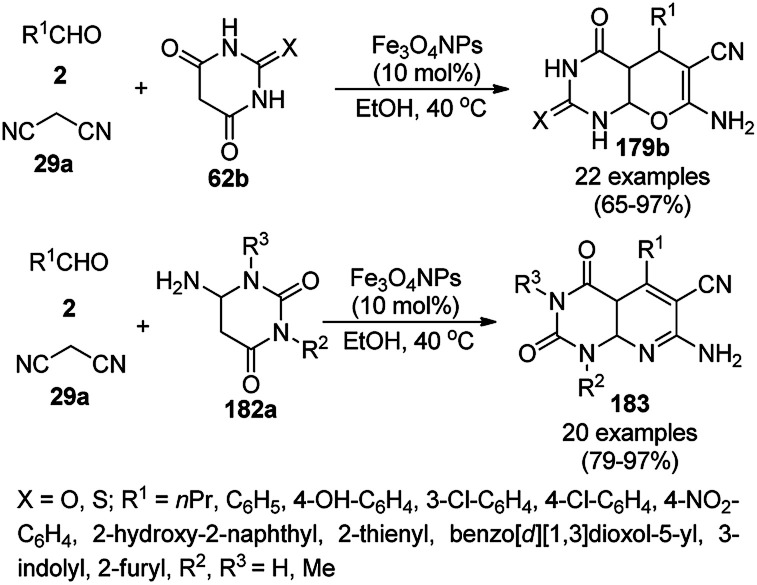
Synthesis of pyrano[2,3-*d*]pyrimidines (179b), and pyrido[2,3-*d*]pyrimidines (182) catalyzed by iron nanoparticles.

Kidwai *et al.* reported the three-component one-pot synthesis of pyrano[2,3-*d*]pyrimidines (179b)/pyrido[2,3-*d*]pyrimidines (182) from the reaction of substituted arylaldehyde (2), 29a and barbituric acid or thiobarbituric acid (62b)/uracil or *N*,*N*′-dimethyl uracil (182a) in ethanol at 40 °C *via* domino Knoevenagel–Michael condensation (Scheme 94).^192^ The nanoferrite particles were synthesized from FeSO_4_·7H_2_O and Fe_2_(SO_4_)_3_ using ammonium hydroxide, and the nanoparticulate structure of the compound was confirmed by XRD, TEM and FTIR. Kidwai *et al.* also explored the same protocol for the synthesis of spirooxindoles using isatins, 29a and cyclic ketones. The catalyst was recycled for up to four catalytic runs with a loss of 3% of yield of the product compared to that obtained in the first catalytic run. Later, in 2016, urea (fertilizer)-based ionic liquid (IL)-stabilized Fe_3_O_4_ MNPs were successfully used as catalysts for the synthesis of 179b in excellent yields without the use of any solvent at 60 °C.^193^ Further, in 2017, Zarei *et al.* reported the synthesis of 183 using silica-bonded *S*-sulfonic acid-supported ferromagnetic MNPs without solvent at 100 °C using 2, 29a and 2,4-diamino-6-hydroxypyrimidines.^194^ Maleki *et al.* also reported the similar synthesis of 179b using biodegradable and eco-friendly catalysts such as cellulose-based nanocomposites anchored on Fe_3_O_4_ in water at rt in 76–98% yield.^195^

**Scheme 95:**
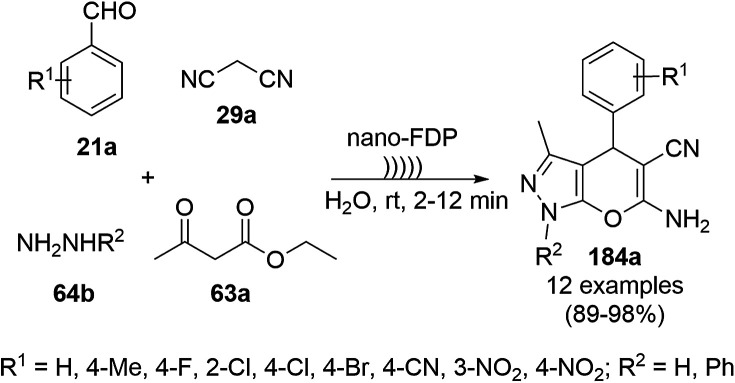
Nano-FDP-catalyzed synthesis of dihydropyrano[2,3-*c*]pyrazoles (184a).

**Scheme 96:**
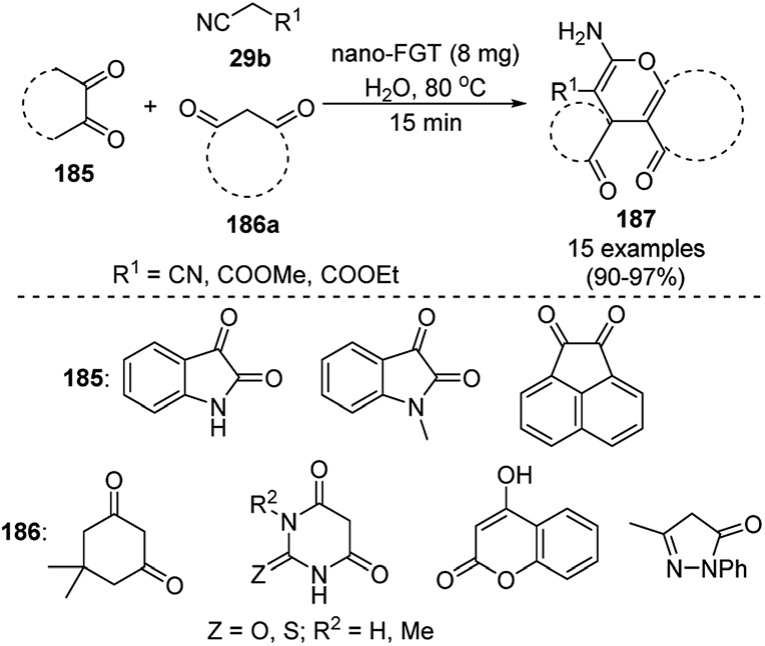
Nano-FGT catalyzed synthesis of spirooxindoles (187).

Pal *et al.* reported the one-pot multicomponent synthesis of dihydropyrano[2,3-*c*]pyrazoles (184a) catalyzed by paramagnetic NPs such as nano-Fe_3_O_4_-DOPA-l-proline (nano-FDP) from substituted arylaldehydes (21a), 29a, 63a and hydrazine/phenyl hydrazine (64b) in water at rt in 89–98% yield (Scheme 95).^196^ The NCs were prepared *via* the treatment of FeNO_2_ and Fe_2_SO_4_ with NH_4_OH followed by coating of the formed Fe_3_O_4_ NPs to form Fe_3_O_4_-DOPA nanoparticles, which were further reacted with ^*t*^BOC-protected l-proline using DIPEA and HBTU to form Fe_3_O_4_-DOPA-BOC-l-proline nanoparticles. These NPs were deprotected using trifluoroacetic acid (TFA) to yield the final NPs nano-FDP. The NPs were characterized *via* FT-IR, EDX, SEM, and TEM. The l-proline present on nano-FDP catalyzed the formation of the cyano-imine intermediate *via* Knoevenagel condensation with aldehyde and malononitrile followed by condensation with pyrrolidinone to undergo cyclocondensation to form the target compounds. The catalyst recycling study revealed only the loss of 22% of product at the end of the fifth catalytic run. Further, Pal *et al.* also reported the catalytic use of Fe_3_O_4_ NPs loaded with glutathiones (nano-FGT) for the rapid synthesis of spirooxindoles (187) from cyclic 1,2-diones (185), malononitrile or cyano methyl or ethyl carboxylate (29b) and active methylene compounds (186a) using water as the solvent *via* Knoevenagel–Michael-cyclization (Scheme 96).^197^ Previously, Varma *et al.* reported the catalytic applicability of the same nano-FGT to catalyze the Paal–Knorr reaction for the synthesis of *N*-substituted pyrroles from tetrahydro-2,5-dimethoxyfuran and amines under aqueous conditions.^198^

**Scheme 97:**
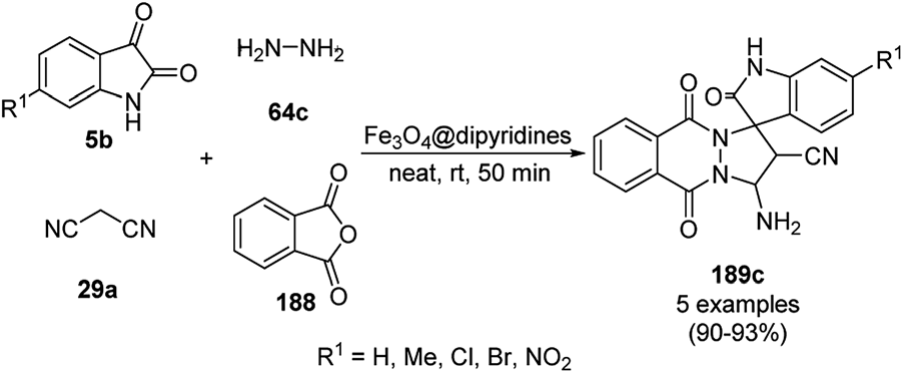
One-pot synthesis of pyrazolophthalazinyl spirooxindoles (189c) catalyzed by Fe_3_O_4_@dipyridines.

Sadeghzadeh *et al.* synthesized pyrazolophthalazinyl spirooxindoles (189c) using Fe_3_O_4_/SiO_2_/propyltriethoxysilane/methylene dipyridine NPs from 6-substituted isatins (5b), 64c, 29a and phthalic anhydride (188) under solvent-free conditions in 90–93% yield (Scheme 97).^199^ The NPs were synthesized by coating prepared Fe_3_O_4_ NPs on tetraethyl orthosilicate (TEOS) and their further treatment with 3-chloropropyltriethoxysilane followed by methylene dipyridine. The synthesized NPs were characterized *via* FT-IR, XRD, TGA, and TEM (30–50 nm). The catalyst was recycled several times without appreciable loss in its catalytic activity. Sadeghzadeh *et al.* also studied the amount of leached catalyst during ten consecutive reruns with the catalyst, and the present protocol exhibited the advantage of increasing the yield of the final product.

**Scheme 98:**
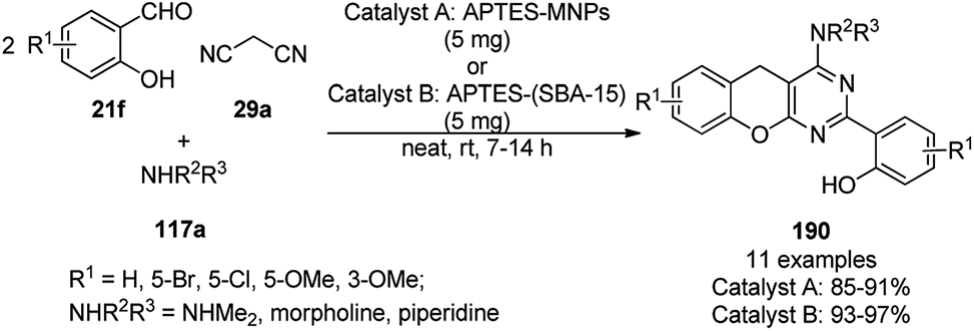
Synthesis of chromeno[2,3-*d*]pyrimidines (190) catalyzed by Fe_3_O_4_ NPs.

Shaterian *et al.* reported 3-aminopropyltriethoxysilane (APTES) coated on magnetic Fe_3_O_4_ nanoparticles [APTES-MNPs; catalyst A] or mesoporous silica SBA-15 [APTES-(SBA-15); catalyst B] as new catalysts for the synthesis of chromeno[2,3-*d*]pyrimidine derivatives (44c) from the reaction of substituted salicylaldehydes (21f), 29a and secondary amines (117a) under solvent-free conditions at room temperature in 85–91% yield (Scheme 98).^200^ The role of the catalyst can be attributed to the formation of the imidine intermediate from the cyano intermediate formed by Knoevenagel condensation and Pinner reaction. The green applicability of the catalyst was proven by the recycling of the catalyst for up to five catalytic cycles without considerable decay in its activity. The reported catalysts such as lithium perchlorate (LiOCl_4_),^201^ and ionic liquid [bmim][BF_4_],^202^ for the synthesis of chromeno[2,3-*d*]pyrimidines [190; X, R^1^ = H, R^2^–R^3^ = –(CH_2_)_2_–*O*–(CH_2_)_2_] *via* the model reaction among salicylaldehyde, malononitrile, and morpholine were found to be inferior in terms of yield and reaction time. Further, they have recently reported the catalytic use of (3-oxo-[1,2,4]triazolidin-1-yl)bis(butane-1-sulfonic acid) anchored on γ-Fe_2_O_3_ NCs for the synthesis of spiro indeno[1,2-*b*]quinoxalines using amines, isatoic anhydride, ninhydrin, and *o*-phenylene diamine.^203^

**Scheme 99:**
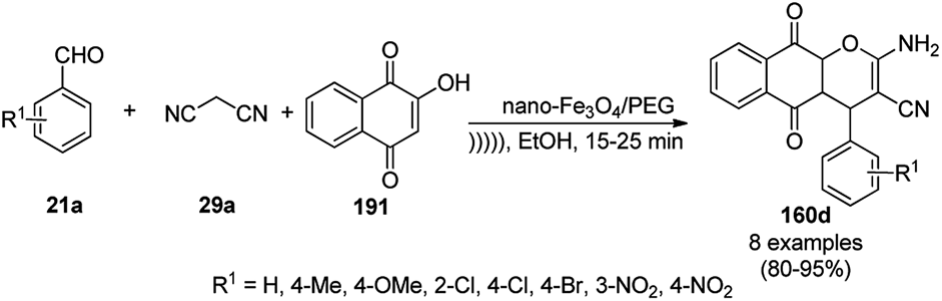
Synthesis of benzo[*g*]chromenes (160d) using nano-Fe_3_O_4_/PEG.

Safaei-Ghomi *et al.* reported the synthesis of benzo[*g*]chromenes (160d) in the presence of a catalytic amount of Fe_3_O_4_/PEG NPs under ultrasonic conditions in ethanol at rt in 80–95% yield (Scheme 99).^204^ They synthesized Fe_3_O_4_ NPs from FeCl_3_·6H_2_O and FeCl_2_·4H_2_O in the presence of ammonium hydroxide at 80 °C followed by the ultrasonic-mediated treatment with sodium oleate and PEG-400 to obtain the final NPs. The structural integrity of the NPs was confirmed *via* XRD, FE-SEM, FT-IR, TGA, DLS, and VSM. The NCs were recycled five times to prove their green synthetic applications. The catalyst played a key role *via* the activation of cyanide (29a) and carbonyl of the substituted aromatic aldehyde (21a) to promote the Knoevenagel condensation.

**Scheme 100:**
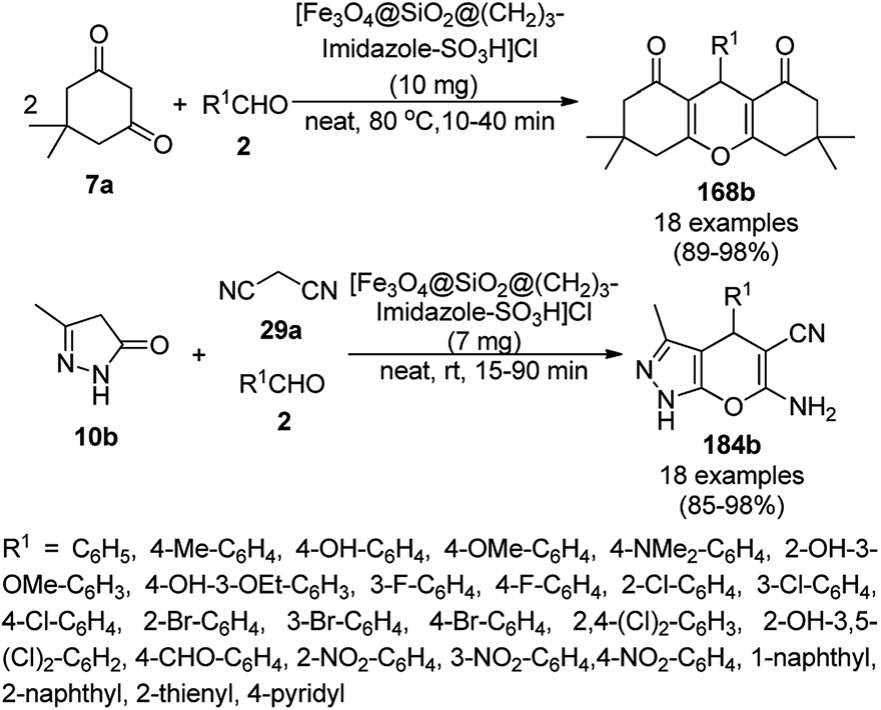
Synthesis of 1,8-dioxo-octahydroxanthene (168b) and dihydropyrano[2,3-*c*]pyrazole derivatives (184b) catalyzed by MNPs.

Zolfigol *et al.* reported the synthesis of 1,8-dioxo-octahydroxanthenes (168b) from dimedone (7a) and substituted benzaldehydes (2) under solvent-free conditions at 80 °C catalyzed by an imidazole-based IL stabilized on SiO_2_-coated Fe_3_O_4_ MNPs as a heterogeneous acid catalyst (Scheme 100).^172^ The same protocol was also explored for the synthesis of dihydropyrano[2,3-*c*]pyrazoles (184b) from 29a, substituted arylaldehyde (2) and 3-methyl-1*H*-pyrazol-5(4*H*)-ones (10b) at rt in good to excellent yields. Zolfigol *et al.* synthesized Fe_3_O_4_ from FeCl_3_ and Na_2_SO_3_ and further treated it with tetraethyl orthosilicate (TEOS) and (3-chloropropyl) triethoxysilane. Finally, Fe_3_O_4_@SiO_2_@(CH_2_)_3_Cl was reacted with imidazole and chlorosulfonic acid to obtain the final MNPs, which were fully characterized *via* spectroscopic techniques such as FT-IR, and TGA, DTA, EDX, XRD, SEM, TEM and AFM analysis. The developed protocol exhibits the advantage of short reaction time, good yield and low catalytic loading.^205^ The nitrogen adsorption–desorption isotherms using Brunauer–Emmett–Teller (BET) analysis of Fe_3_O_4_ and the MNPs revealed that the MNPs have a higher surface area compared to Fe_3_O_4_. The recyclability of the catalyst was demonstrated by the authors for up to six catalytic cycles without appreciable loss in its catalytic activity.

**Scheme 101:**
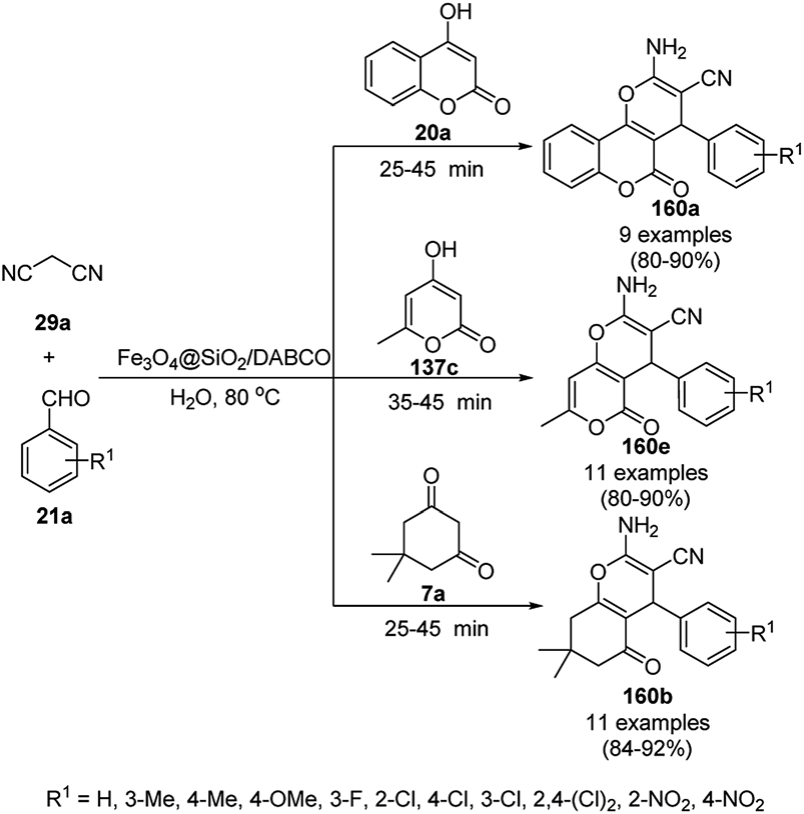
Synthesis of 4*H*-benzo[*b*]pyran derivatives (160a/e/b) catalyzed by Fe_3_O_4_@SiO_2_/DABCO.

Kiasat *et al.* reported the one-pot synthesis of pyran annulated heterocyclic compounds (160a/e/b) under aqueous conditions catalyzed by double-charged MNPs composed of diazabicyclo[2.2.2]octane chloride silica hybrid, Fe_3_O_4_@SiO_2_/DABCO at 80 °C (Scheme 101) *via* the three-component reaction of substituted aldehydes (21a), malononitrile (29a) and 1,3-diketone (20a/137c/7a).^206^ The MNPs were prepared following an improved chemical co-precipitation method^207^ using FeCl_2_·4H_2_O and FeCl_3_·6H_2_O. Bis(*n*-propyltrimethoxysilane)-1,4-diazoniabicycle [2.2.2]octane chloride (BPTDABCOCl) was synthesized by the authors using DABCO and 3-chloropropyltrimethoxysilane (CPTMS).^208^ Subsequently, the Fe_3_O_4_ MNPs and BPTDABCOCl were reacted at 80 °C in water–ethanol co-solvent *via* the sol–gel process to obtain the target double-charged MNPs. The synthesized MNPs were well characterized *via* FT-IR, XRD, SEM, TEM, VSM, TGA, and DTA. Since they were magnetic in nature, the MNPs were recovered using a magnet and reused.

**Scheme 102:**
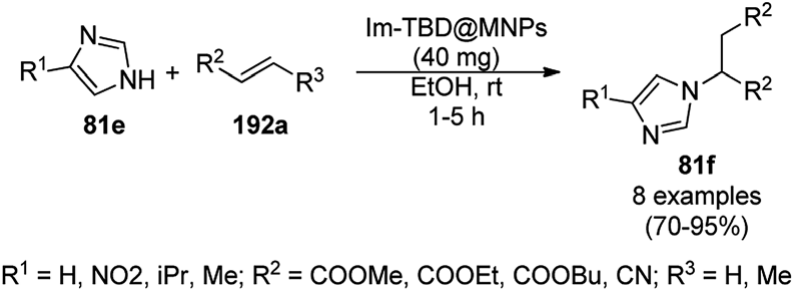
Synthesis of *N*-alkylated imidazoles (81f) catalyzed by Im-TBD@MNPs.

Xu *et al.* reported the catalytic use of *N*-alkylated imidazoles (81f) *via* the aza-Michael addition of substituted imidazoles (81e) with alkenes (192a) using 1,5,7-triazabicyclo[4.4.0]dec-5-ene (TBD)-modified magnetite NPs (Im-TBD@MNPs) under mild conditions (Scheme 102).^209^ The same catalyst was also explored for the aza-Michael addition of amines to α,β-enones and for the synthesis of *N*,*N*′-substituted ureas successfully. The target NPs were prepared *via* the treatment of Fe_3_O_4_@SiO_2_ NPs with CPTES, 3-(3-chloropropyl)imidazole and TBD. These reactions could be catalyzed by the ionic counterparts of Im-TBD@MNPs.

**Scheme 103:**
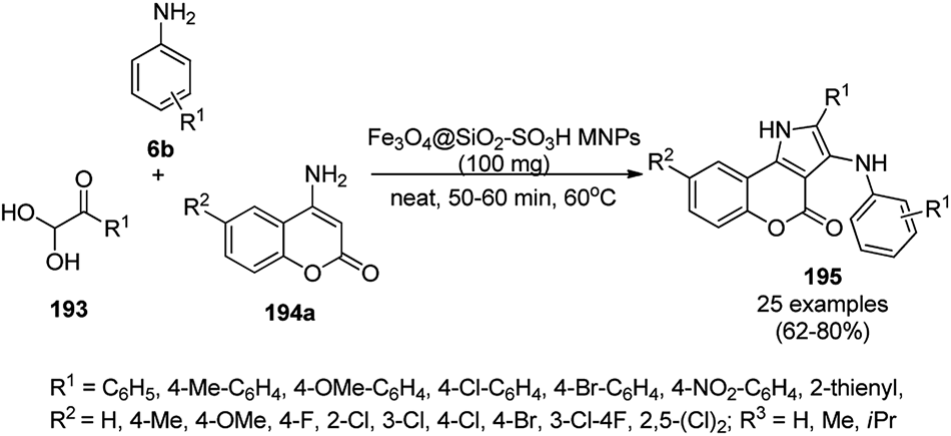
Synthesis of arylamine-substituted chromeno[4,3-*b*]pyrrol-4(1*H*)-ones (195).^213^

**Scheme 104:**
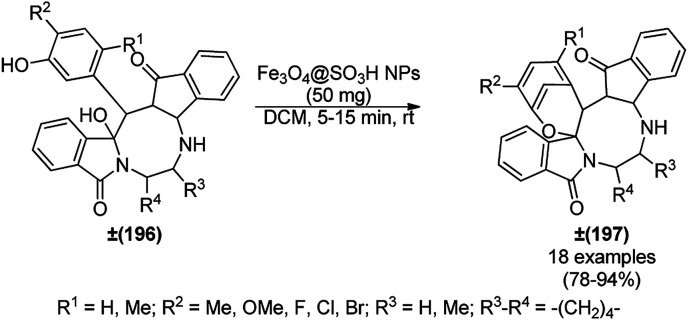
Fe_3_O_4_@SO_3_H NP-catalyzed synthesis of diaza-cyclooctanoids (197).

Indole- and pyrrole-fused coumarin derivatives have been reported as HIV-1 integrase inhibitors,^210^ anti-cancer agents^211^ and immunomodulators.^212^ Pramanik *et al.* reported the synthesis of functionalized pyrrole-fused coumarins (195) using Fe_3_O_4_@SiO_2_–SO_3_H as the catalyst from the fusion of the aryl or heteroaryl glyoxal monohydrates (193), arylamines (6b) and 4-aminocoumarins (194a, Scheme 103). The reusability of the Fe_3_O_4_@SiO_2_–SO_3_H MNPs *via* magnetic separation was found to be altered after the seventh catalytic run.^213^ Previously, they also reported the catalytic use of Fe_3_O_4_@SO_3_H NPs for the synthesis of dihydrofuran-based cyclooctanoids (197) *via* the dehydration of cyclooctanoids (196) at rt in a shorter reaction time (Scheme 104).^214^ Shiri *et al.* reported the use of the same catalyst for the green synthesis of pyrrolo[1,2-*a*]pyrazines *via* the three-component reaction of ethylenediamine, dimethyl acetylenedicarboxylate, and β-nitrostyrene.^215^

**Scheme 105:**
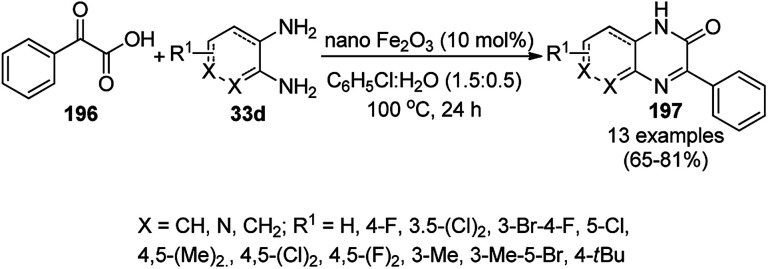
Reactions of 2-oxo-2-phenylacetic acid (197) with substituted benzene-1,2-diamines (33d) in the presence of superparamagnetic Fe_2_O_3_ NPs.^216^

The reaction between α-keto acids such as 2-oxo-2-phenylacetic acid (196) and various aryl-1,2-diamines (33d) produced 3-phenylquinoxalin-2(1*H*)-one (197) and 2-phenyl benzo[*d*]imidazoles. Phan *et al.* reported the selective synthesis of 197 over 2-phenyl benzo[*d*]imidazoles employing superparamagnetic Fe_2_O_3_ nanoparticles (NPs) as a recyclable heterogeneous catalyst (Scheme 105).^216^ Several attempts were made using a mixture or combination of solvents, and C_6_H_5_Cl : H_2_O (1.5 : 0.5) was found to be the best condition for the selective synthesis of 197. These catalytic nanoparticles were recovered by magnetic decantation and their activity was maintained up to the ninth catalytic cycle. The XRD studies of these nanoparticles also revealed that the structure of the catalyst was retained even after the ninth catalytic run compared to the fresh unused magnetic NPs.

**Scheme 106:**
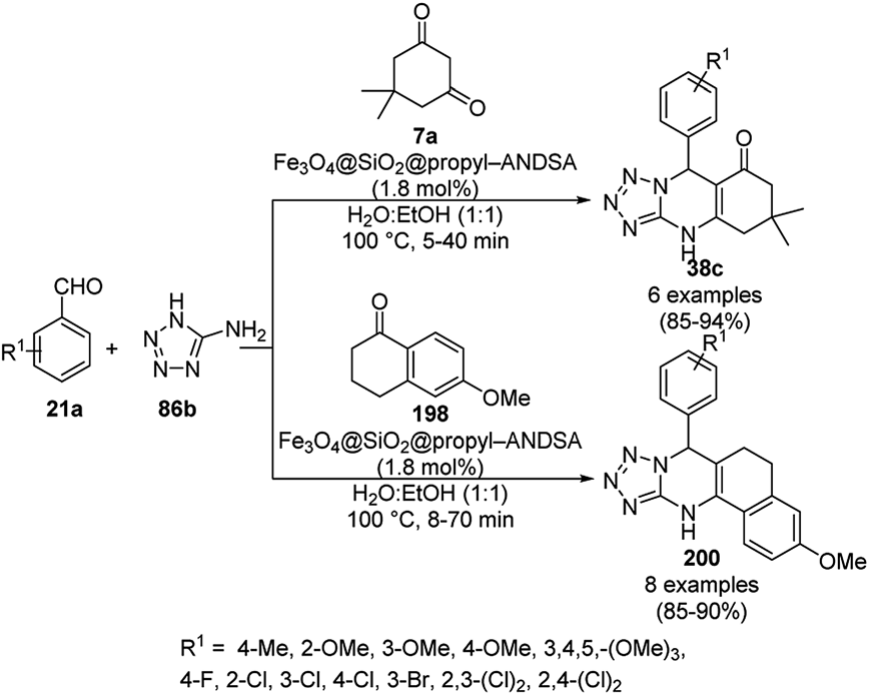
Fe_3_O_4_@SiO_2_@propyl–ANDSA-catalyzed one-pot synthesis of tetrahydrobenzo[*h*]tetrazolo[5,1-*b*]quinazolines (199) and tetrahydrotetrazolo[1,5-*a*]quinazolines (200).

Ghorbani-Vaghei *et al.* reported the one-step, green and mild synthesis of tetrahydrobenzo[*h*]tetrazolo[5,1-*b*]quinazolines (199) and tetrahydrotetrazolo[1,5-*a*]quinazolines (200) from 5-aminotetrazole (86b), aldehydes (21a), and 6-methoxy-3,4-dihydronaphthalen-1(2*H*)-one (198) or dimedone (7a) in the presence of 7-aminonaphthalene-1,3-disulfonic acid-functionalized magnetic Fe_3_O_4_ nanoparticles, *viz.* Fe_3_O_4_@SiO_2_@propyl–ANDSA (1.8 mol%), as the catalyst in H_2_O : EtOH (1 : 1) at 100 °C (Scheme 106).^217^ The catalyst was obtained *via* the co-precipitation of Fe_3_O_4_, followed by silica coating using tetraethyl-orthosilicate (TEOS) and (3-chloropropyl)-triethoxysilane to give chloro-functionalized Fe_3_O_4_@SiO_2_@Cl MNPs, which were further reacted with 7-aminonaphthalene-1,3-disulfonic acid (ANDSA) to yield the final NPs. These NPs were finally characterized *via* SEM, TEM, FTIR, EDX, TGA, and VSM.

**Scheme 107:**
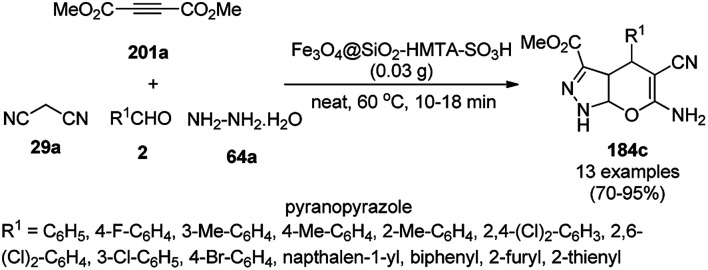
Synthesis of pyranopyrazole derivatives (184c) using Fe_3_O_4_@SiO_2_-HMTA-SO_3_H as the catalyst.

Ghorbani-Vaghei *et al.* also reported the synthesis of new magnetic catalysts Fe_3_O_4_@SiO_2_-HMTA-SO_3_H by reacting silica-coated Fe_3_O_4_ magnetic nanoparticles with (3-chloropropyl)triethoxysilane, hexamethylenetetramine and chlorosulfonic acid. Using this catalyst with a low loading, pyranopyrazole derivatives were synthesized by fusing four components, acetylene dicarboxylate (201a), malononitrile (29), 2, and 64a, without the use of solvent in a shorter reaction time (10–18 min, Scheme 107).^218^ The authors claimed the superiority of the Fe_3_O_4_@SiO_2_-HMTA-SO_3_H-catalyzed synthesis considering its faster and greener reaction with good yields and feasible recovery of the catalyst assisted by an external magnet compared to other reported protocols.^219–221^ However, the recyclability of the catalytic system was found to decrease significantly after the fourth catalytic cycle. Salehzadeh *et al.* reported the catalytic use of a molybdenum Schiff-base complex supported on Fe_3_O_4_@SiO_2_ NPs for the synthesis of pyranopyrazoles using different approaches mediated by benzaldehyde, malononitrile, and 3-methyl-1-phenyl-2-pyrazolin-5-one under solvent-free conditions at rt.^222^ Recently, they also reported pyridinium tribromide ionic liquid-supported silica coated ferrite NPs for the efficient synthesis of 4-phenyl pyrimidines from triethoxy methane, ammonium acetate and substituted acetophenones.^223^

**Scheme 108:**
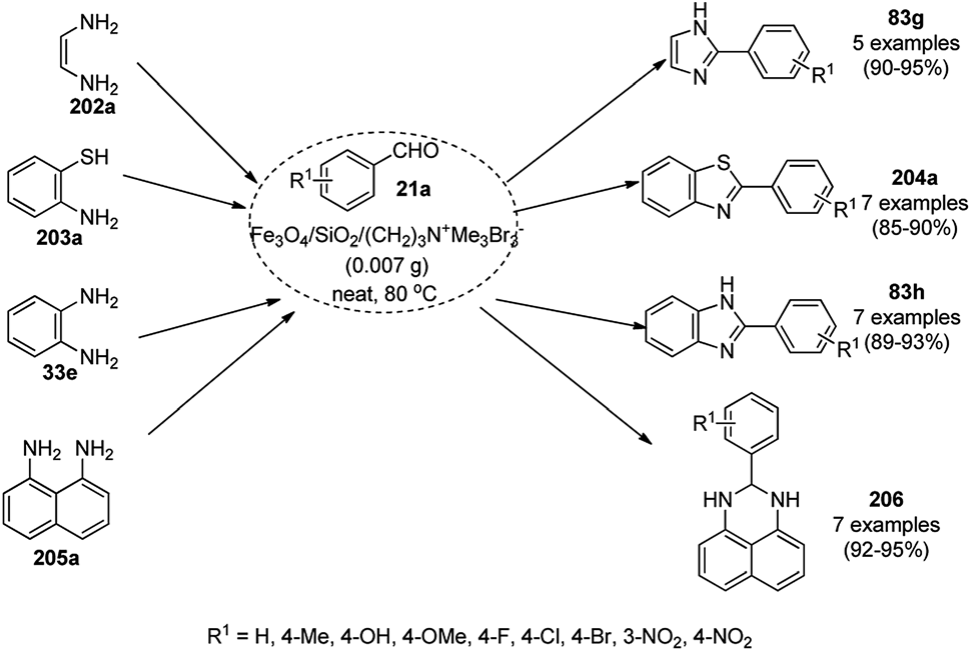
Fe_3_O_4_/SiO_2_/(CH_2_)_3_N^+^Me_3_Br_3_-catalyzed synthesis of 2-aryl imidazoles (83g)/benzothiazoles (204a)/benzimidazoles (83h)/spermidine (206) derivatives.

Yavari *et al.* reported the synthesis of 2-aryl imidazoles (83g)/benzothiazoles (204a)/benzimidazoles (83h)/spermidine (206) derivatives *via* cyclocondensation with aromatic aldehydes catalyzed by functionalized magnetic core NPs (Fe_3_O_4_/SiO_2_/(CH_2_)_3_N^+^Me_3_Br_3_) under solvent-free conditions at 80 °C in 89–95% yield (Scheme 108).^224^ The NPs were prepared *via* the co-precipitation of ferrous (FeSO_4_·7H_2_O) and ferric salts (FeCl_3_·7H_2_O) with NH_4_OH followed by coating with silica using the Stöber process, grafting with aminopropylsilanes and coupling with methyl iodide and tribromide (KBr/Br_2_). The prepared NPs were characterized *via* TEM, XRD, FTIR, and VSM. The recyclability of the catalyst was studied up to seven catalytic runs without any catalytic decay.

**Scheme 109:**
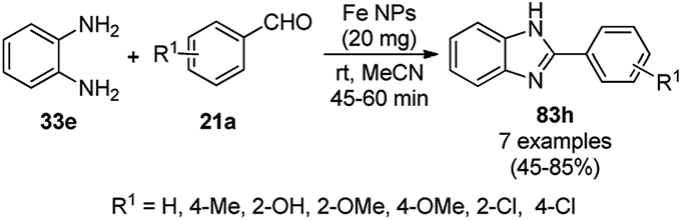
Synthesis of 2-aryl benzimidazoles (83h) from *o*-phenylene diamine (33e) and aryl aldehydes (21a) catalyzed by Fe NPs.

Kumar *et al.* fabricated the iron oxide NPs for the preparation of 2-arylbenzimidazoles (83h) *via* the cyclocondensation of *o*-phenylene diamine (33e) and various substituted benzaldehydes (21a) in 45–85% yield (Scheme 109).^225^ The iron oxide NPs were prepared from FeCl_3_·2H_2_O and aqueous extract of *Passiflora tripartita* var. mollissima fruit. The flavonoid glycosides present in the extract led to the formation of Fe_3_O_4_ NPs *via* the oxidation of Fe(OH)_3_, as characterized by the formation of a dark black-colored mixture. These solid NPs were further characterized *via* TEM (25 nm), DLS, FT-IR, XRD, and UV-Vis. The reusability of the catalyst was tested up to five times following the separation of the FeNPs *via* magnetic separation, and washing with ethanol followed by activation.

**Scheme 110:**
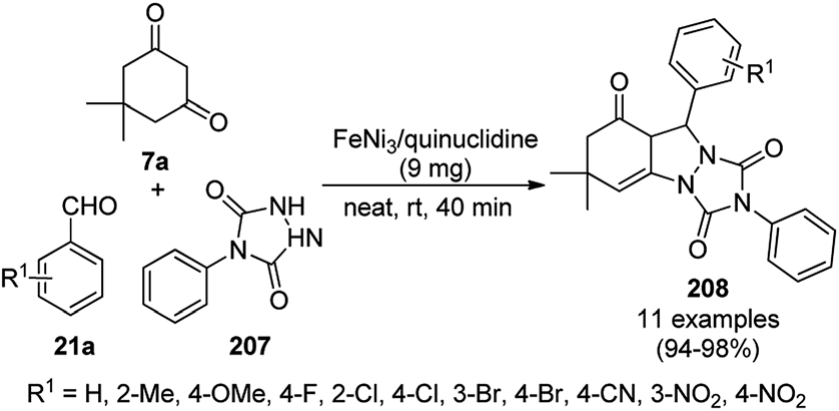
Green one-pot synthesis of triazolo[1,2-*a*]indazole-triones (208) catalyzed by FeNi_3_/quinuclidine.

Sadeghzadeh *et al.* reported quinuclidin-3-thiol supported on propylsilane-functionalized silica-coated FeNi_3_ nanoparticles (FeNi_3_/quinuclidine) as a novel catalyst for the synthesis of triazolo[1,2-*a*]indazole-triones (208) from dimedone (7a), substituted benzaldehyde (21a), and 4-phenylurazole (207) under solvent-free conditions in 40 min in excellent yields (Scheme 110).^226^ The catalyst was prepared *via* the co-precipitation method using FeCl_2_·4H_2_O and NiCl_2_·6H_2_O salts in NH_4_OH followed by treatment with TEOS to obtain FeNi_3_/SiO_2_ NPs. These NPs were then reacted with 3-chloropropyltriethoxysilane and quinuclidin-3-thiol to obtain the final NPs. The structural integrity of the final NPs was confirmed *via* powder XRD, TEM, FTIR, TGA, and VSM. The green applicability of these NCs was demonstrated by recycling them eight times in organic transformations. The role of the catalyst was proposed by Sadeghzadeh *et al.* to mediate the formation of hydrogen bonds between the nucleophilic nitrogen of quinuclidine and hydrogen of 7a and urazole.

**Scheme 111:**
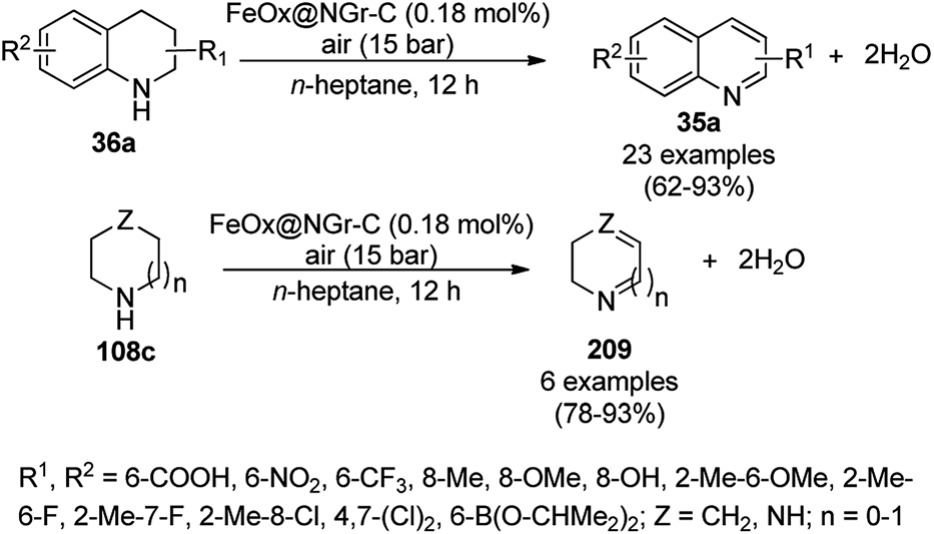
Oxidation of tetrahydroquinoline, piperidine and piperazine catalyzed by FeO_*x*_@NGr-C.

Beller *et al.* formulated a new type of catalyst iron oxides covered with nitrogen (1,10-phenathroline) doped-graphene shells supported on carbon FeO_*x*_@NGr-C for the synthesis of substituted quinolines (36a) *via* the aerial oxidation of 1,2,3,4-tetrahydroquinolines (108c) in *n*-heptane (Scheme 111).^227^ A similar protocol was also extended for the synthesis of pharmaceutically relevant intermediates and oxidation of secondary alkyl amines to imines. The catalyst was synthesized using iron acetate and 1,10-phenanthroline as the nitrogen doping agent and graphene precursor, followed by pyrolysis and selective leaching, and the NCs were characterized *via* HRTEM, HAADF, XPS, and XRD. The recycling of the catalyst was been studied for up to five cycles with a gradual decrease in its catalytic activity.

**Scheme 112:**
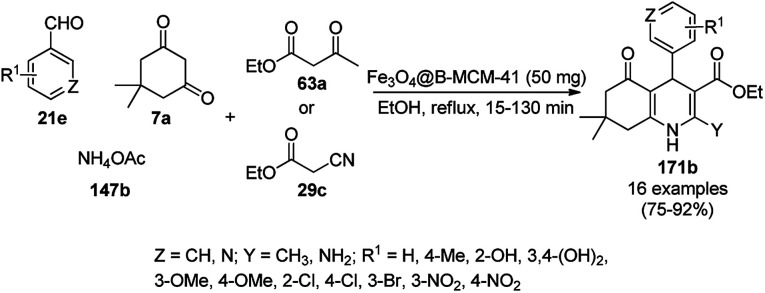
Synthesis of polyhydroquinolines (171b) using Fe_3_O_4_@B-MCM-41 as a new catalyst.

Abdollahi-Alibeik *et al.* reported the use of Fe_3_O_4_@B-MCM-41 for the synthesis of polyhydroquinolines (171b) from 7a, aromatic aldehyde (21e), ethyl acetoacetate (63a) or active methylene compounds (29c), and ammonium acetate (147b) under reflux in ethanol in 75–92% yield (Scheme 112).^228^ Fe_3_O_4_ NPs were prepared *via* co-precipitation using FeCl_2_·4H_2_O and FeCl_3_·H_2_O, which were further treated with cetyltrimethylammonium bromide (CTAB), boric acid, aqueous ammonia, and tetraethyl orthosilicate followed by calcination at high temperature to obtain the final NPs, Fe_3_O_4_@B-MCM-41. The NPs were further characterized *via* VSM, FT-IR, SEM, TEM, XRD and BET analysis. The recyclability of the catalyst was studied for up to three times without any decay in its activity.

**Scheme 113:**
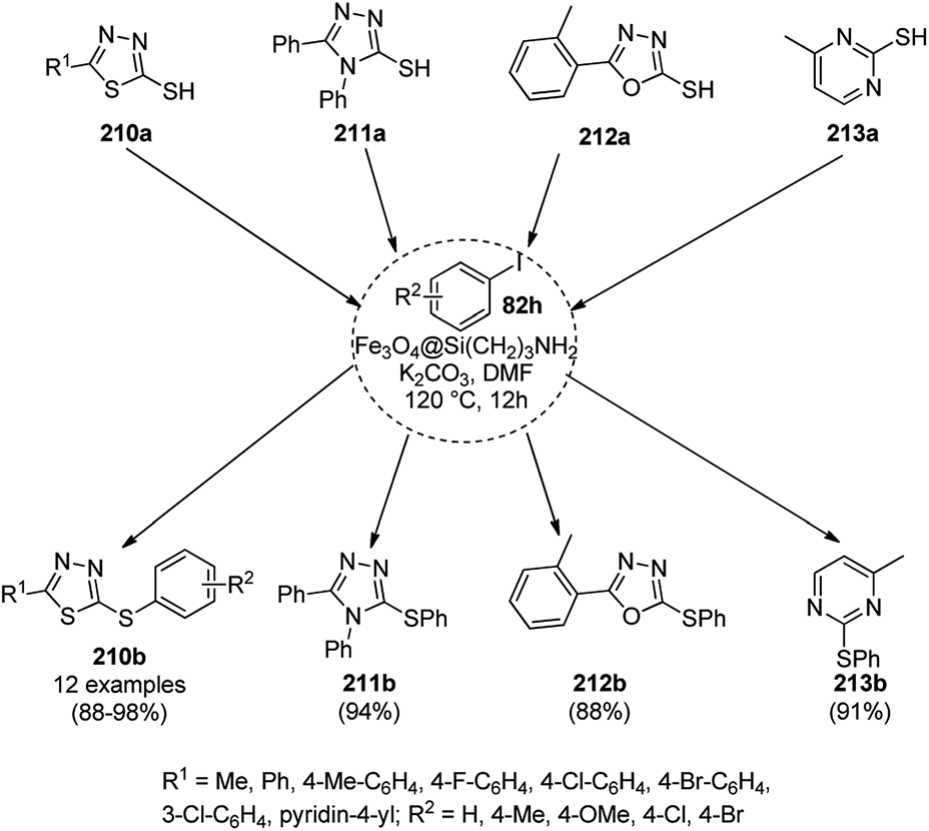
*S*-Arylation of heteroaromatic thiols using aryl iodides (82h) catalyzed by Fe_3_O_4_@Si(CH_2_)_3_NH_2_ NPs.

Yanhong Liu *et al.* synthesized Fe_3_O_4_@Si(CH_2_)_3_NH_2_ NPs from ferric aminopropyltriethoxysilane acetoacetate, oleylamine and benzyl ether to obtain Fe_3_O_4_ NPs, followed by coating with APTES. The nano-behavior of the synthesized NPs was confirmed *via* TEM, XRD, XPS and FT-IR. These NPs were applied for the *S*-arylation of substituted thiols containing 1,3,4-thiadiazole (210a), 1,3,4-triazole (211a), 1,3,4-oxadiazole (212a) and pyrimidine (213a) with aryl iodide (82h) using Fe_3_O_4_@Si(CH_2_)_3_NH_2_ NPs in DMF at 120 °C in excellent yields (Scheme 113).^229^ The magnetic NPs were recycled for up to five catalytic runs successfully, where only 5% yield of the final product was lost compared to that of the first catalytic run.

**Scheme 114:**
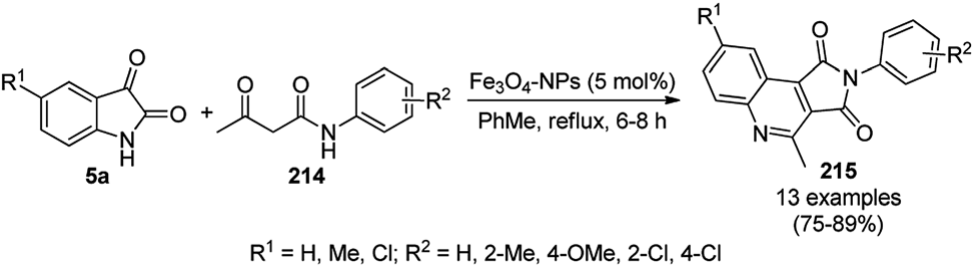
Synthesis of pyrrolo[3,4-*c*]quinoline-1,3-diones (215) using Fe_3_O_4_ NCs.

Basavegowda *et al.* synthesized ferromagnetic Fe_3_O_4_ NPs following a green synthetic approach involving the ultrasonication of an Fe_2_O_3_ solution and leaf extract of *Perilla frutescens*.^230^ The change in color of the reaction mixture of iron oxide and leaf extract of *P. frutescens* from light red to dark brown indicated the reduction of Fe^3+^ to Fe^2+^ by the flavanoids and phenolic compounds present in the leaf extract. These NPs were characterized *via* UV-Vis, SAM, TEM, XRD, XPS, VSM, EDRX, TGA and FT-IR. The Fe_3_O_4_ nanoparticle-catalyzed synthesis of various pyrrolo[3,4-*c*]quinoline-1,3-diones (215) with β-ketoarylamides (214) and substituted isatins (5a) under reflux in toluene is highlighted in the following scheme (Scheme 114). The reuse of the NPs was demonstrated in the recycling experiment for up to five catalytic runs with 86% yield of the synthetic target.

**Scheme 115:**
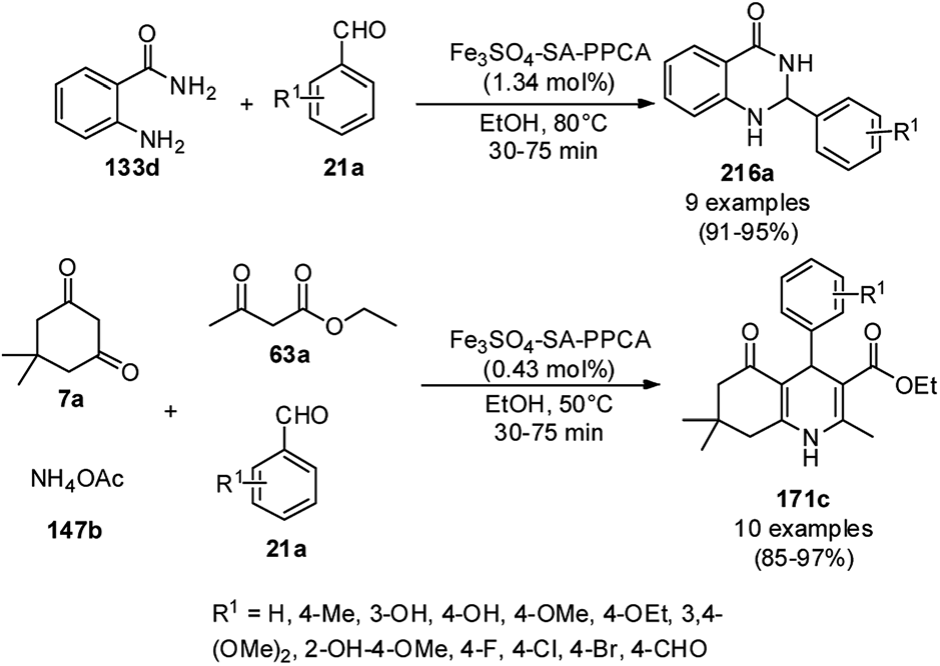
Synthesis of 2,3-dihydroquinazolin-4(1*H*)-ones (216a) and polyhydroquinolines (171c).

Ghorbani-Choghamarani *et al.* synthesized piperidine-4-carboxylic acid (PPCA)-functionalized Fe_3_O_4_ nanoparticles (Fe_3_O_4_-PPCA) *via* the co-precipitation of iron oxide in the presence of PPCA followed by the grafting of chlorosulfonic acid to obtain the final NPs (Fe_3_O_4_-SA-PPCA).^231^ The NPs were employed for the synthesis of 2,3-dihydroquinazolin-4(1*H*)-ones (216a) from anthranilamide (133d), benzaldehyde (21a) in EtOH at 80 °C in 91–95% yield (Scheme 115) and explored for the synthesis of polyhydroquinolines (171c) from 7a, 63a, aromatic aldehydes (21a) and 147b at 50 °C in 85–97% yield. The commercial applicability of the catalyst was explored with respect to recycling of the MNPs for up to six catalytic reruns. Further they also compared previously reported catalytic protocols for the synthesis of 216a^232–234^ and 171c^235–238^ derivatives, and claimed that the Fe_3_O_4_-SA-PPCA-catalyzed protocol is a transitional metal-free approach with higher yields in shorter times.

**Scheme 116:**
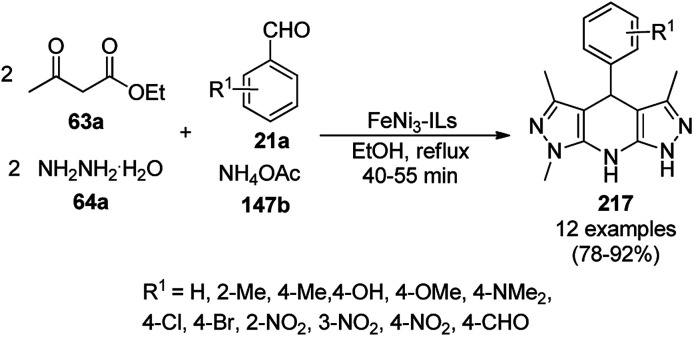
FeNi_3_-ILs catalyzed synthesis of the tetrahydrodipyrazolo pyridines (217).

Safaei-Ghomi *et al.* reported the synthesis of FeNi_3_-ILs MNPs by capping of nano FeNi_3_ generated *via* the co-precipitation of FeCl_2_·4H_2_O and NiCl_2_·6H_2_O with tetraethyl orthosilicate (TEOS) followed by chlorosulfonic acid and ethanolamine.^239^ Further, they characterized the NPs *via* SEM, XRD, FT-IR, and VSM. The synthesis of tetrahydrodipyrazolo pyridines (217) was performed *via* the multicomponent reaction (MCR) among 63a, 64a, substituted arylaldehyde (21a) and 147b under reflux in ethanol (Scheme 116). The catalyst could be recycled several times for the synthesis of tetrahydrodipyrazolo pyridines without appreciable loss in the yield of the final product. The catalyst played a vital role *via* interaction with the substrate using its free hydroxyl and ammonium ions. Further, in the same year, they also reported the synthesis of pyrans using Fe_3_O_4_ NPs supported on polyhedral oligomeric silsesquioxane NCs in ethanol.^240^

**Scheme 117:**
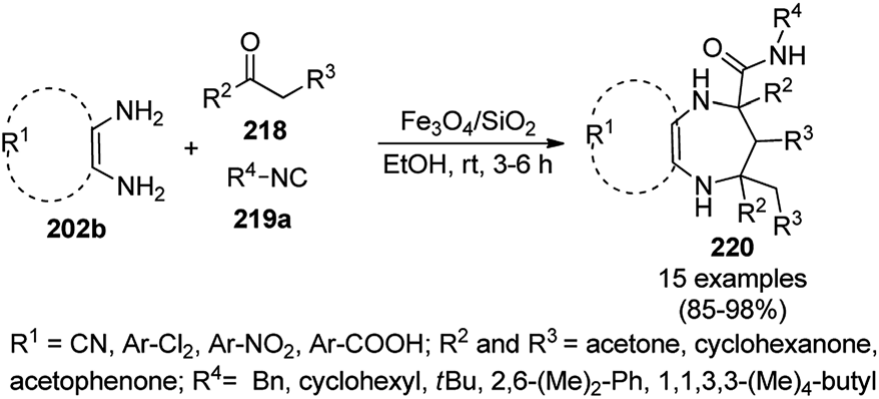
Synthesis of diazepines (220) catalyzed by Fe_3_O_4_/SiO_2_ NPs.

Ali Maleki reported the silica-supported magnetic MNP (Fe_3_O_4_/SiO_2_)-catalyzed synthesis of benzodiazepines or diazepines (220) from 1,2-diamine (202b), linear/cyclic ketone (218) and isocyanide (219a) in ethanol at rt in 85–98% yield (Scheme 117).^241^ The MNPs were prepared using Fe_3_O_4_ NPs and TEOS, and characterized *via* TEM. Using asymmetric *o*-phenylene diamine, Ali Maleki reported the regioselective synthesis of benzodiazepine with the formation of a single isomer. The recycling of the NPs for up to six subsequent runs for the synthesis of the target compound using the recovered catalyst from *o*-phenylene diamine, acetone, benzoyl isocyanide demonstrated the stability of these NPs with respect to their catalytic potential.

**Scheme 118:**
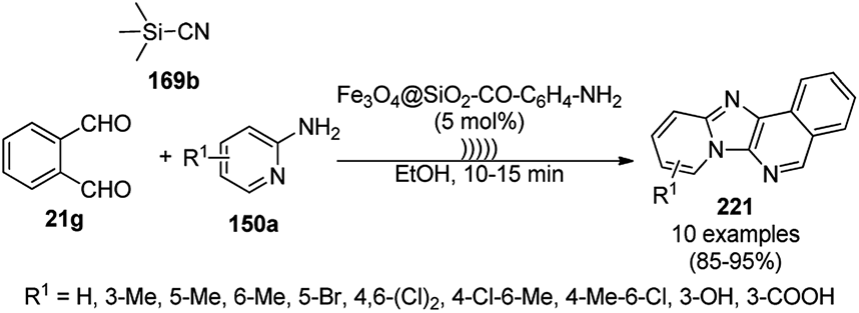
Synthesis of pyrido[2′,1′:2,3]imidazo[4,5-*c*]isoquinolines (221) reported by Maleki *et al.*

Maleki *et al.* reported Fe_3_O_4_@SiO_2_–CO–C_6_H_4_–NH_2_ as a new catalyst for the ultrasonic wave-mediated, rapid synthesis of pyridoimidazoisoquinolines (221) from *o*-phthalaldehyde (21g), trimethyl silane cyanide (169b) and 2-amino pyridines (150a) in ethanol in excellent yields (Scheme 118).^242^ The Fe_3_O_4_@SiO_2_ NPs were prepared and functionalized with 4-amino benzoyl chloride, which characterized *via* SEM, EDX, TGA and DTA. The heterogeneous catalyst was recycled consecutively for up to five runs without decay in its catalytic activity.

**Scheme 119:**
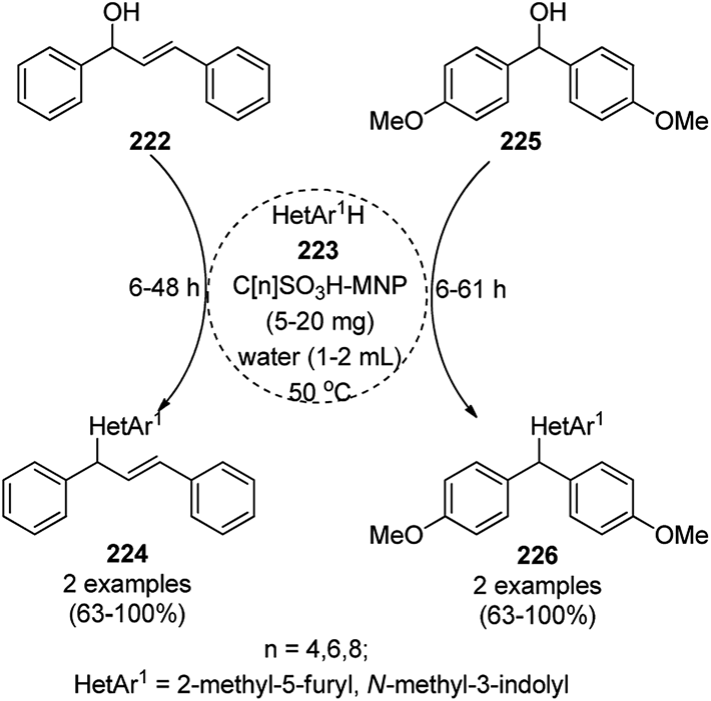
Fe_3_O_4_ NP-decorated calix[*n*]arene sulfonic acid-catalyzed nucleophilic substitution of alcohols.

Yilmaz and Sayin reported the synthesis of Fe_3_O_4_ NP-decorated calyx[*n*]sulfonic acid C[*n*]SO_3_H-MNP by adorning Brønsted acidic calix[*n*]arenes with [3-(2,3-epoxypropoxy)-propyl]-trimethoxysilane-coated Fe_3_O_4_ NPs. Further, these NCs were used for the nucleophilic substitutions of *sec*-alcohols such as (*E*)-1,3-diphenylprop-2-en-1-ol (222) and bis(4-methoxyphenyl)methanol (225) with heterocyclic scaffolds (223) such as 2-methylfuran and *N*-methylindole (Scheme 119) in water.^243^ The catalyst was separated by magnetic decantation and recycled several times.

**Scheme 120:**
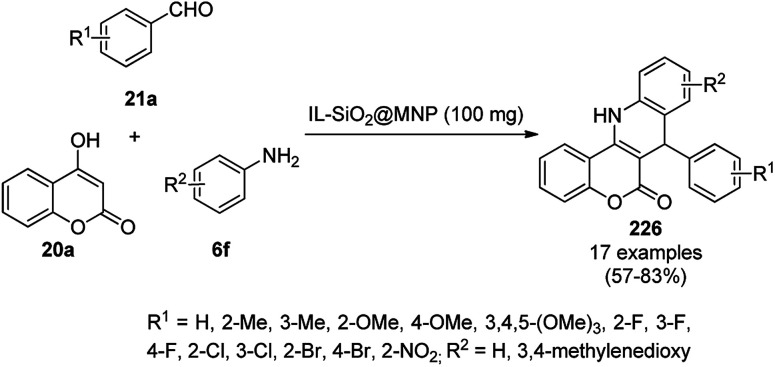
Synthesis of 6*H*-chromeno[4,3-*b*]quinolin-6-ones (226).

Iron oxide NPs prepared *via* co-precipitation of Fe^2+^ and Fe^3+^ salts in alkaline medium were loaded with silica and 1-butyl-3-(3-trimethoxypropyl)-1*H*-imidazol-3-ium chloride to prepare IL-loaded superparamagnetic MNPs (IL-SiO_2_@MNP, Scheme 120) by Mahadvi *et al.*^244^ These recyclable MNPs were used for the synthesis of 6*H*-chromeno[4,3-*b*]quinolin-6-ones (226) *via* the multi-component reaction of 4-hydroxycoumarin (20a), aniline or 3,4-methylenedioxyaniline (6f) and substituted benzaldehydes (21a). Further, they reported the synthesis of pyrano[3,2-*c*:5,6-*c*′]dichromene-6,8-diones from 4-hydroxy coumarin and benzaldehydes using DABCO-modified super-paramagnetic iron oxide NPs (SPIONs) in aqueous ethanol at 80 °C.^245^

**Scheme 121:**
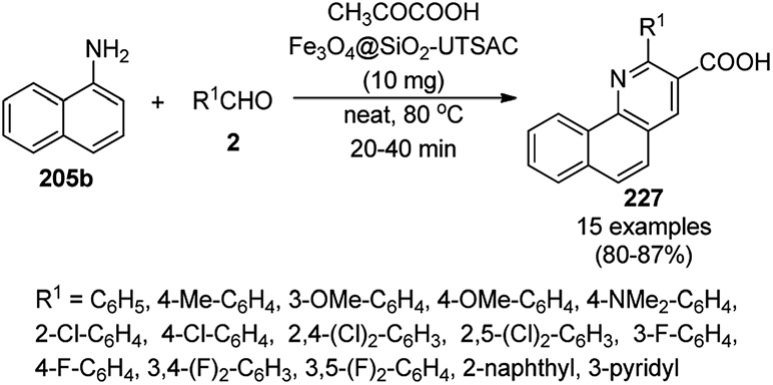
Synthesis of 2-aryl-quinoline-4-carboxylic acids (227) catalyzed by Fe_3_O_4_@SiO_2_-UTSAC.

The interesting synthesis of 2-aryl-quinoline-4-carboxylic acids (227) was reported by Zolfigol and co-workers (Scheme 121).^246^ The condensation of α-naphthyl amine (205b) with aryl aldehydes (2) formed an imine intermediate, which reacted with pyruvic acid to undergo cyclization–dehydration assisted by silica-coated Fe_3_O_4_ MNPs ligated with urea-based thiazolium sulfonic acid chloride (Fe_3_O_4_@SiO_2_-UTSAC). Fe_3_O_4_@SiO_2_ MNPs prepared by the treatment of Fe_3_O_4_ NPs with tetraethyl orthosilicate (TEOS) were treated with a urea-based ligand followed by salt formation using chlorosulfonic acid to furnish the final NPs. The catalysts were successfully recycled for up to six runs using an external magnet.

**Scheme 122:**
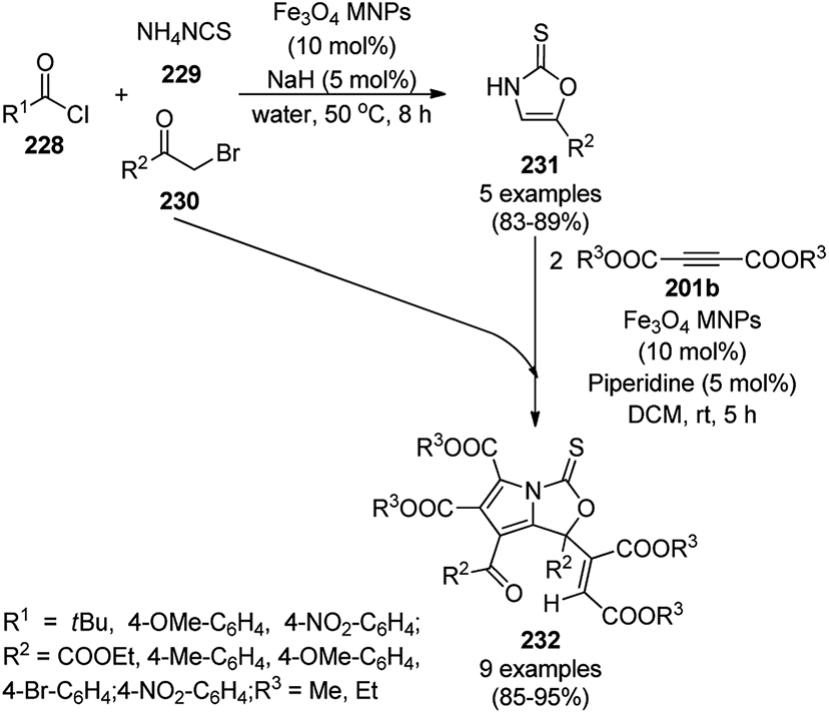
Fe_3_O_4_ MNP-catalyzed synthesis of 1*H*-pyrrolo-[1,3]-oxazoles (232).

Fe_3_O_4_ MNPs, which were prepared *via* the reduction of ferrous and ferric salt aqueous extracts of dried clover leaves, were found to catalyze the synthesis of oxazoles (231) from the cyclocondensation of acyl chloride (228), ammonium thiocyanate (229) and substituted α-bromo ketones (230, Scheme 122).^247^ Further, 231 together with 230 and activated acetylenic dicarboxylates (201b) were used in the MNP-catalyzed synthesis of 1*H*-pyrrolo-[1,3]-oxazoles (232), which showed promising anti-oxidant activity.

**Scheme 123:**
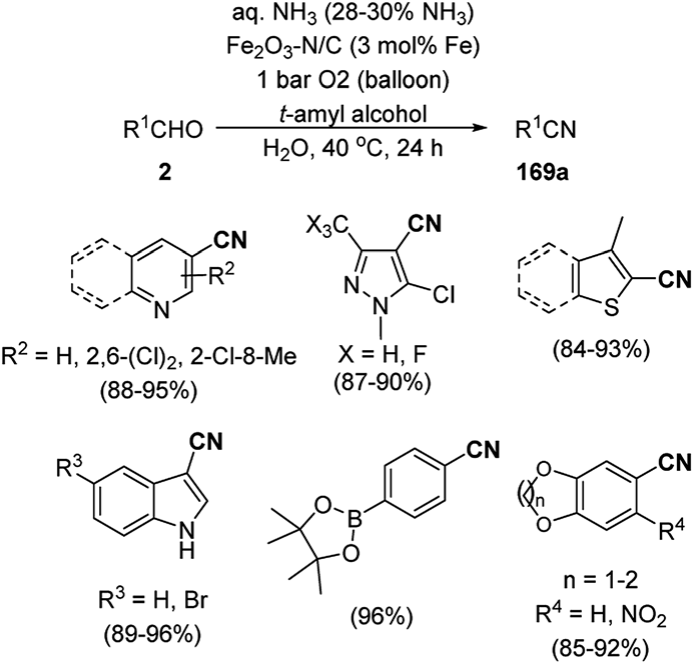
Synthesis of cyano-heterocycles (169a) from heterocyclic carbaldehydes (2) catalyzed by Fe_2_O_3_–N/C.

Jagadeesh and colleagues developed a catalytic protocol involving iron oxide NP nitrogen-doped graphene layer (Fe_2_O_3_–N,C)-catalyzed aerial oxidation for the green synthesis of nitriles (169a) from carbaldehydes (2) (Scheme 123) under aqueous conditions using aqueous ammonia and *t*-amyl alcohol.^248^ It was also explored for the synthesis of amides using heterocyclic amides at 120 °C and 10 bar in air from 2. The NCs were synthesized *via* the deposition of Fe(ii)(1,10-phenanthroline)_3_(OAc)_2_ on organic Vulcan XC 72R following their previously reported protocol.^249^ Further, the scalability of this protocol was assessed using 1–5 g of 2 successfully. The durability of the catalysts was demonstrated in a recycling experiment for up to six re-runs with consistent catalytic action without leaching of iron (ICP).

**Scheme 124:**
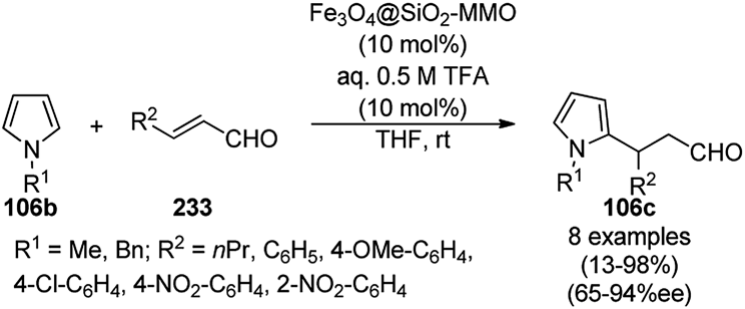
Friedel–Crafts alkylation of pyrroles (106b) using α,β-enal compounds (233).

Pericàs *et al.* anchored the first generation alkynyl Macmillan catalyst on azide-linked Fe_3_O_4_@SiO_2_ NPs *via* CuI-catalyzed azide alkyne [3 + 2] cycloaddition (CuAAC) or click reaction and reported its catalytic use for the Friedel–Crafts alkylation of pyrrole (106b) with α,β-enal derivatives (233) (Scheme 124) to synthesize pyrrol-2-propanals (106c) in moderate to excellent enantiomeric excess (ee).^250^ The quantitative recovery of the MNPs under an external magnetic field enabled the recycling of the NCs for up to six runs, but a reduction in catalytic performance was observed, which was attributed to the loss of its structural integrity.

**Scheme 125:**
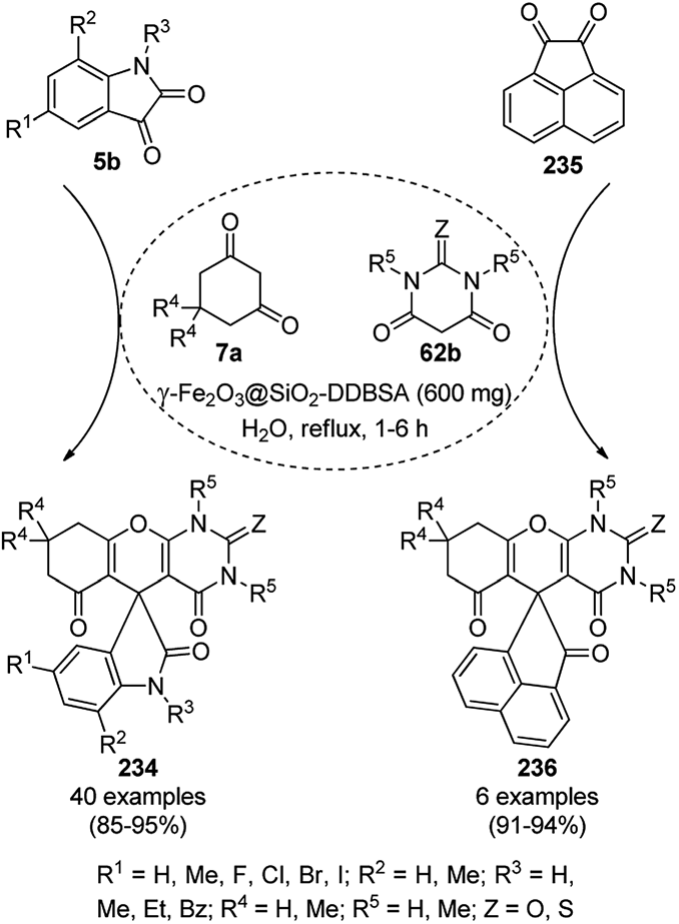
One-pot synthesis of spiro[chromeno[2,3-d]pyrimidine-5,3′-indoline] (234) and spiro[acenaphthylene-1,5′-chromeno[2,3-*d*]pyrimidine] (236).

Silica-coated γ-Fe_2_O_3_ anchored with dodecyl benzene sulfonic acid (γ-Fe_2_O_3_@SiO_2_-DDBSA) was used as a catalyst in the one-pot synthesis of spiro[chromeno[2,3-d]pyrimidine-5,3′-indoline] (234) or spiro[acenaphthylene-1,5′-chromeno[2,3-*d*]pyrimidine] (236) *via* the condensation of cyclic diones (7c), (thio)barbituric acids (62b) and *N*-substituted isatins (5b) or acenaphthenequinones (235, Scheme 125) in aqueous solution under reflux.^251^ γ-Fe_2_O_3_ coated with silica was functionalized with dodecyl benzene sulfonic acid *via* ultrasonication. The highly recyclable catalyst was reused for up to six runs, maintaining its structural integrity (TEM and ICP-AES).

**Scheme 126:**
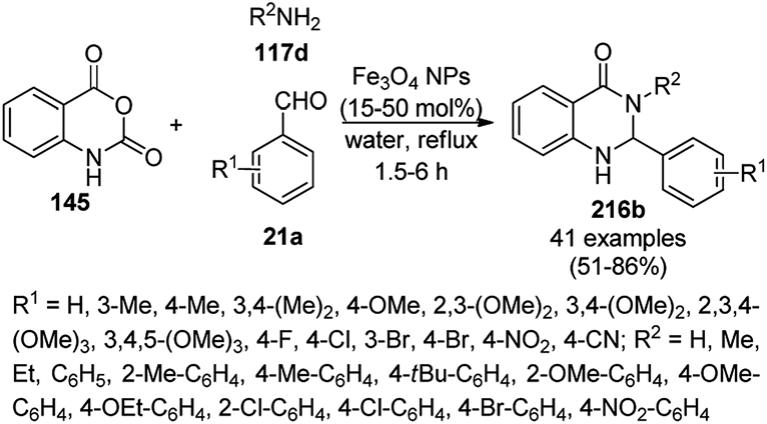
Synthesis of 2,3-dihydroquinazoli-4(1*H*)-ones (216b) catalyzed by Fe_3_O_4_ MNPs.

The Fe_3_O_4_ NP-catalyzed cyclocondensation of isatoic anhydride (145), aliphatic and arylamines (117d) and aromatic carbaldehydes (21a) *via* sequential ring opening-decarboxylation-cyclization led to the synthesis of diverse 3-dihydroquinazoli-4(1*H*)-ones (216b) in water (Scheme 126).^252^ The catalyst was separated using a magnetic stirrer and demonstrated to be durable for up to five runs, resulting in 75–80% yield of 216b (R^1^, R^2^ = Ph).

**Scheme 127:**
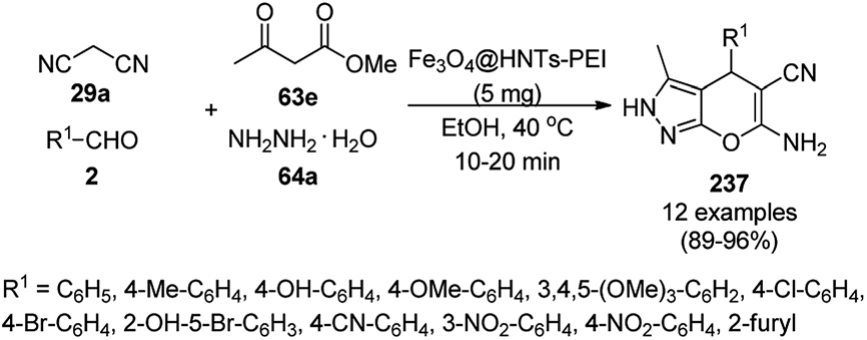
Fe_3_O_4_@HNTs-PEI-catalyzed synthesis of dihydropyrano[2,3-*c*]pyrazoles (237).

Halloysite nanotubes, natural aluminosilicate clay, were loaded with Fe_3_O_4_ NPs followed by linking with 3-chloropropyltrimethoxysilane (CPTMS) and poly(ethylene imine) to obtain poly(ethylene imine)-tagged silica-coated ferric oxide loaded on halloysite nanotubes (Fe_3_O_4_@HNTs-PEI). These NPs were used as catalysts in the organic transformation of malononitrile (29a), aldehydes (2), hydrazine hydrate (64a) and methyl acetoacetate (63e) into dihydropyrano[2,3-*c*]pyrazoles (237, Scheme 127).^253^ The catalyst was recycled up to eight times with 90–96% product yield. The amino-functionalized catalyst played the key role in HB formation or ionic interaction with the reactants or formed intermediates.

**Scheme 128:**
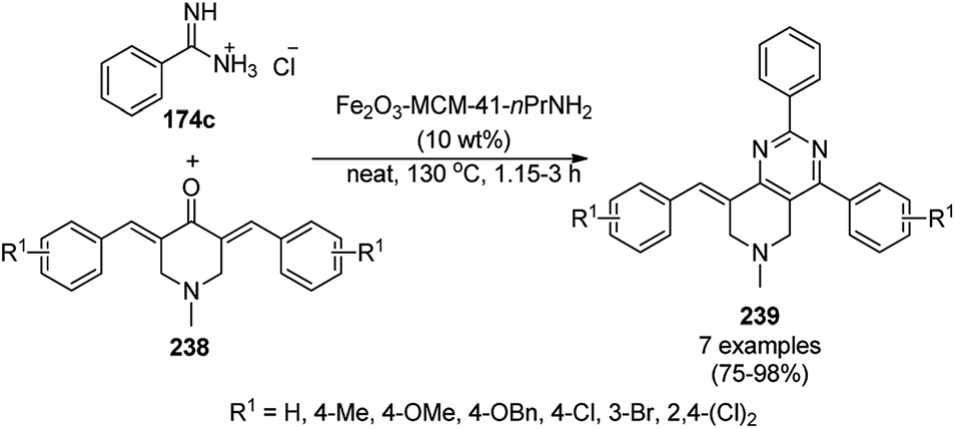
Synthesis of 2,4-diphenylpyrido[4,3-*d*]-pyrimidines (239) catalyzed by nanocatalysts.

Shadjou and Hasanzadeh reported the catalytic use of amino-tagged silica coated with Fe_2_O_3_ NPs for the synthesis of 2,4-diphenylpyrido[4,3-*d*]-pyrimidines (239) from benzamidine hydrochloride (174c) and 3,5-dibenzylidenepiperidin-4-one (238) (Scheme 128).^254^ The catalyst was recycled up to five times without loss in its catalytic performance. Here, the catalyst could catalyze the synthetic transformation by acting as a base and helping in cyclization and aromatization. The reaction performed in the presence of alkali in EtOH yielded some dihydropyrimidine derivatives; however, the critical role of the catalyst in this organic transformation was not clarified.

**Scheme 129:**
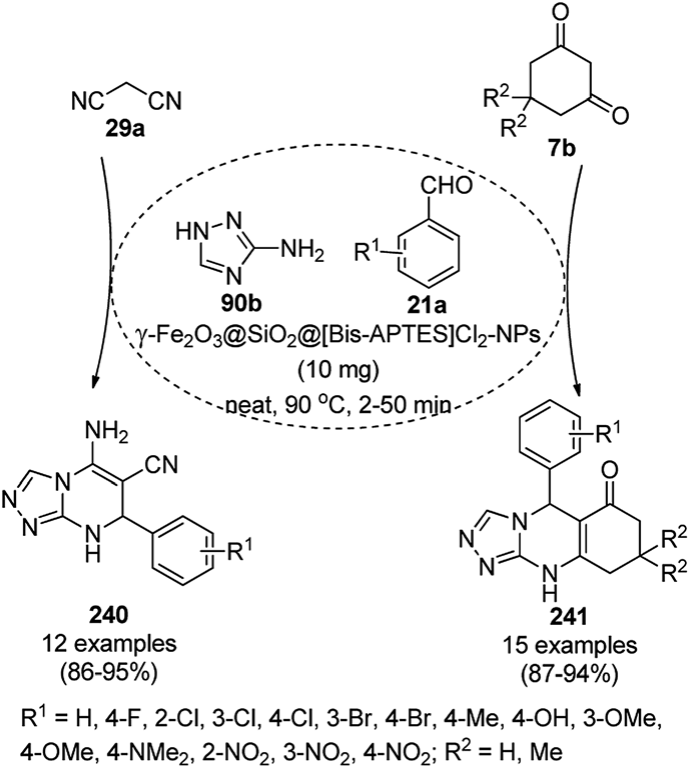
Synthesis of 1,2,4-triazolopyrimidines (240) and quinazolinones (241).

Recently, Shirini *et al.* reported γ-Fe_2_O_3_@SiO_2_ NPs tagged with bis-[(3-aminopropyl)triethoxysilane]dichloride (γ-Fe_2_O_3_@SiO_2_@[Bis-APTES]Cl_2_-NPs) as a recyclable catalyst with a low catalytic loading for the solvent-free synthesis of 1,2,4-triazolopyrimidines (240) and quinazolinones (241) *via* the condensation of 2-amino-1,2,4-triazole (90b) with benzaldehydes (21a) and malononitrile (29a) or cyclic diones (7b, Scheme 129).^255^ The γ-Fe_2_O_3_@SiO_2_@[Bis-APTES]Cl_2_-NPs were obtained by coating silica-coated magnetic NPs with bis-[(3-aminopropyl)triethoxysilane]dichloride obtained from 3-APTES with 1,4-dichlorobutane.

**Scheme 130:**
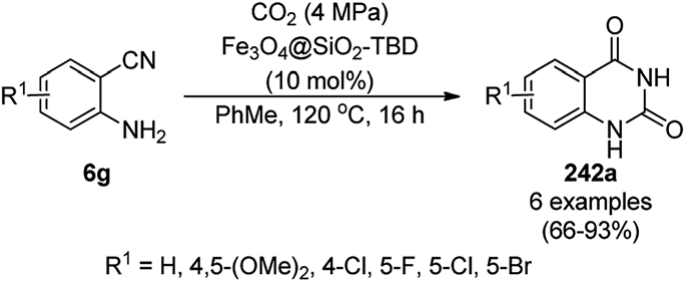
Carboxylation of 2-aminobenzonitriles (6g) catalyzed by TBD@Fe_3_O_4_.

Silica-coated Fe_3_O_4_ NPs tagged with the organic superbase 1,5,7-triazabicyclo[4.4.0]dec-5-ene (Fe_3_O_4_@SiO_2_-TBD) catalyzed the fixation of CO_2_ with 2-aminobenzonitriles (6g) for the synthesis of quinazoline-2,4(1*H*,3*H*)-diones (242a) in 66–93% yield (Scheme 130).^256^ Fe_3_O_4_@SiO_2_ NPs were linked with TBD using 3-glycidyloxypropyltrimethoxysilane to obtain the final NPs. These magnetite NPs were successfully reused for up to four runs with 23% loss in the yield of 242a compared to the first run. The reaction proceeded *via* the base-promoted carbonylation of 6g followed by intramolecular cyclization to yield 242a.

**Scheme 131:**
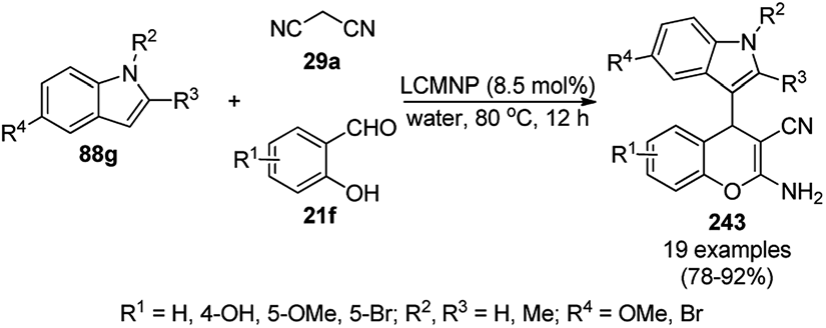
LCMNP-catalyzed synthesis of 243.

**Scheme 132:**
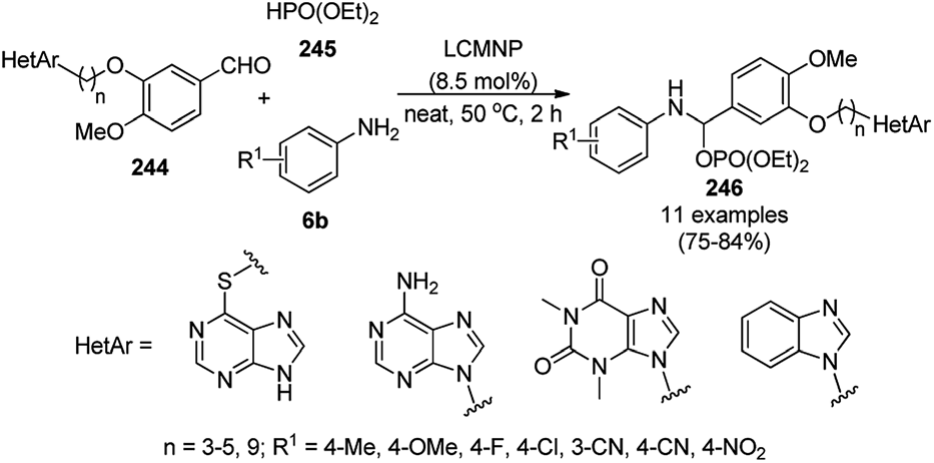
Synthesis of α-aminophosphates (246) catalyzed by LCMNP.

Panahi *et al.* synthesized an l-cysteine-tagged magnetite NP (LCMNP) catalyst *via* the vinylation of Fe_3_O_4_@SiO_2_ NPs followed by grafting with l-cysteine in the presence of azobisisobutyronitrile (AIBN) as a reducing agent. Using this catalyst, the three-component reaction of substituted indoles (88g), malononitrile (29a) and substituted salicylaldehydes (21f) for the synthesis of 2-amino-4*H*-chromenes (243, Scheme 131),^257^ and Kabachnik–Fields reaction of substituted aldehydes (244), diethyl phosphonates (245) and anilines (6b) for the synthesis of anti-cancer α-aminophosphonates possessing the nitrogenous heterocycles theophylline, benzimidazole, and adenine (246, Scheme 132) were successfully achieved in reasonable yields.^258^ They also evaluated 246 for its anti-cancer activity against the Jurkat cancer cell line.

**Scheme 133:**
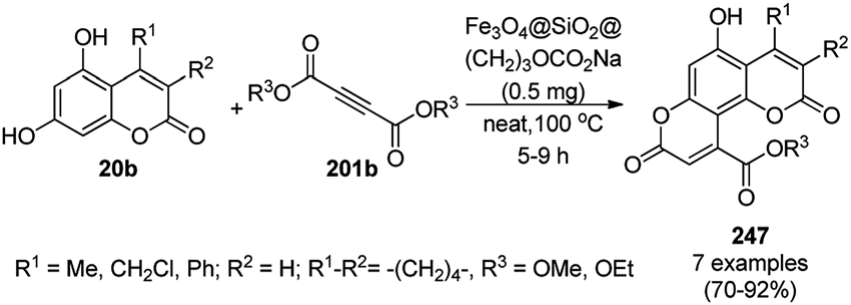
Synthesis of pyranocoumarins (247).

Sodium carbonate-tagged silica-coated Fe_3_O_4_ NP [Fe_3_O_4_@SiO_2_@(CH_2_)_3_OCO_2_Na]-catalyzed nucleophilic substitution for the synthesis of pyranocoumarins (247) was achieved using 5,7-dihydroxycoumarins (20b), and dialkyl acetylene dicarboxylates (201b) (Scheme 133) in 70–92% yield.^259^ Silica-coated FeNPs were treated with 3-chloropropyltriethoxysilane followed by loading sodium carbonate to obtain black-colored NPs. The catalyst was recycled successfully for up to five runs using an external magnet.

**Scheme 134:**
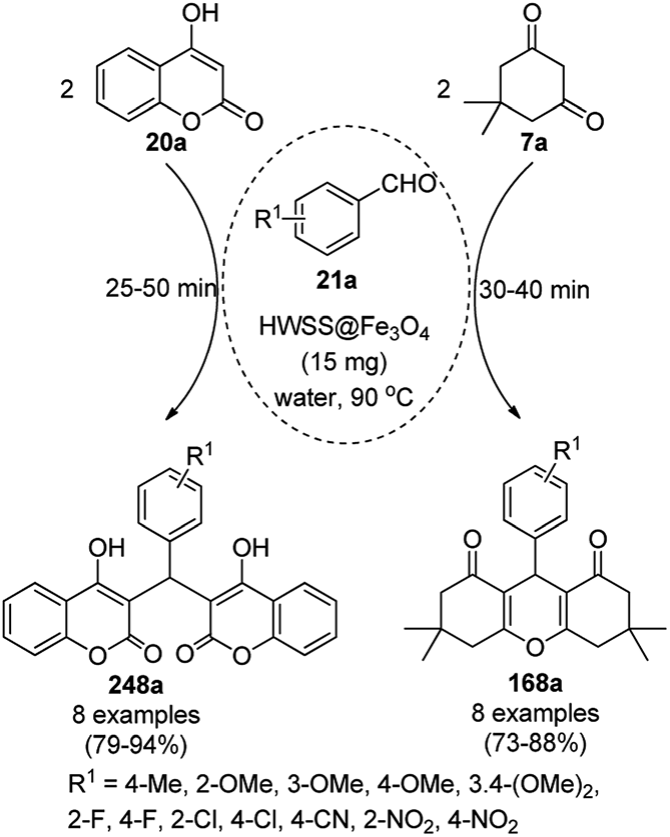
“On-water” synthesis of 248a and 168a.

Fe_3_O_4_ NPs encapsulated in hot water-soluble starch (HWSS@Fe_3_O_4_) were prepared *via* the treatment of Fe_3_O_4_ NPs with hot water-soluble starch and employed in the “on-water” synthesis of 3,3′-(arylmethylene)-bis-(4-hydroxycoumarin-3-yl)s (248a) or 1,8-dioxooctahydroxanthenes (168a) *via* the condensation of benzaldehydes with two equivalents of 4-hydroxy coumarins or dimedone (Scheme 134).^260^ The catalytic potential in this work was attributed to the proton exchange with the reactants.

### 3.6 NiNP-catalyzed synthesis of heterocycles

**Scheme 135:**
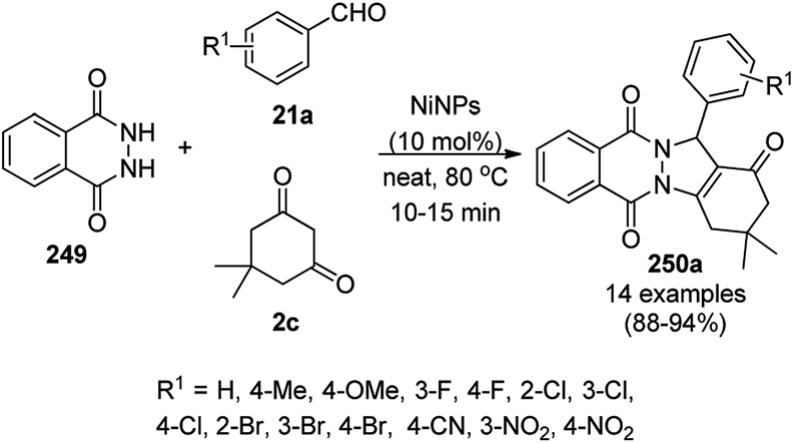
NiNPs catalyzed synthesis of 2*H*-indazolo[2,1-*b*]phthalazine-triones (250a).

NiNPs as NCs were employed in the synthesis of 2*H*-indazolo[2,1-*b*]phthalazine-triones (249) *via* Knoevenagel–Michael-cyclisation from phthalhydrazine (89a), dimedone (2c) and benzaldehydes (21a) (Scheme 135).^261^ The NiNPs were synthesized *via* the reduction of NiCl_2_·6H_2_O with hydrazine hydrate as a reducing agent in a water-in-oil micro-emulsion stabilized by CTAB.^262^ The catalyst was separated *via* centrifugation and recycled up to six cycles. The target compounds (250a) were reported to act as fluorescent probes, as established by the photophysical stability studies.

**Scheme 136:**
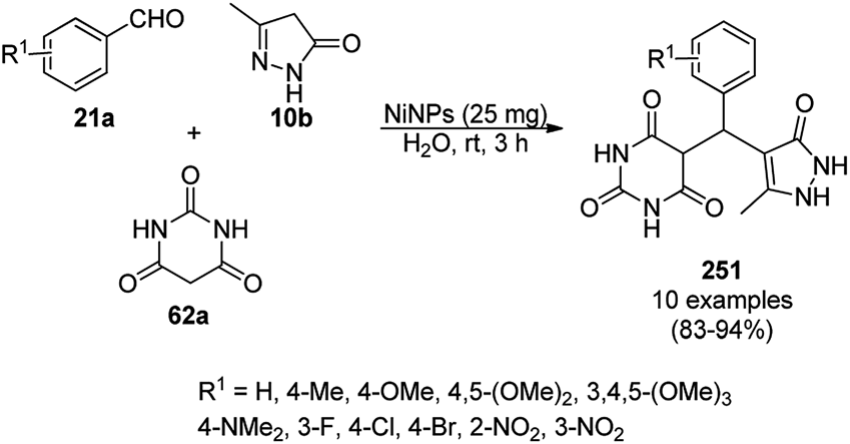
Synthesis of pyrazol-4-yl-methyl-pyrimidine-2,4,6(1*H*,3*H*,5*H*)-triones (251).

**Scheme 137:**
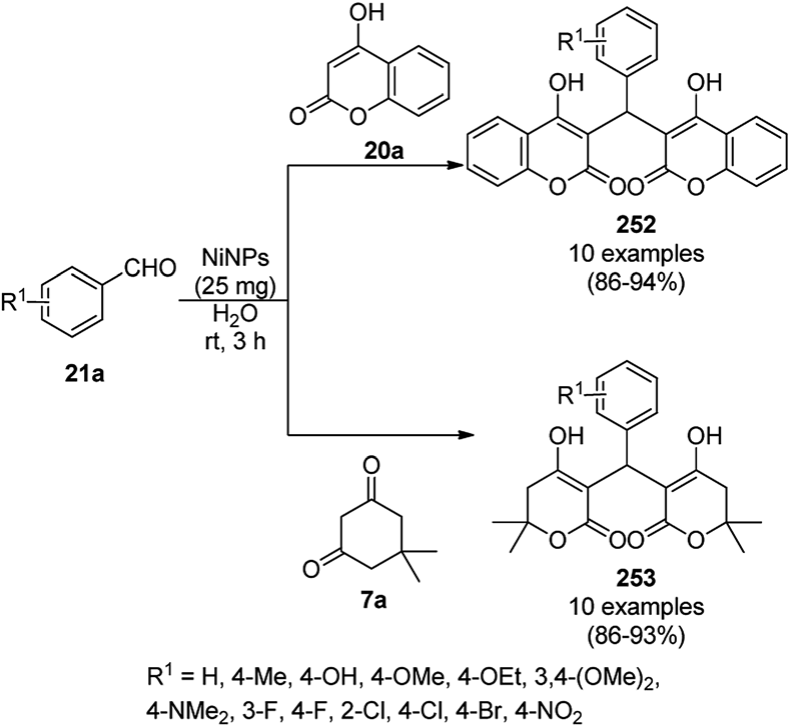
Synthesis of bis(4-hydroxy-2H-chromen-2-one) (252) and bis(3-hydroxy-5,5-dimethylcyclohex-2-enone) (253) catalyzed by NiNPs.

The NiNP-catalyzed synthesis of pyrazol-4-yl-methyl-pyrimidine-2,4,6(1*H*,3*H*,5*H*)-triones (251) from benzaldehydes (21a), 3-methyl-1*H*-pyrazol-5(4*H*)-one (10b) and barbituric acid (62a, Scheme 136) was achieved successfully *via* Knoevenagel condensation under green conditions at rt.^263^ The same NiNP-catalyzed protocol was also applied for the synthesis of anti-bacterial bis(4-hydroxy-2*H*-chromen-2-one) (252) or bis(3-hydroxy-5,5-dimethylcyclohex-2-enone) (253) from 21a and 20a or 7a, respectively (Scheme 137), in good to excellent yields. The NiNPs were synthesized *via* the chemical reduction of nickel chloride hexahydrate and hydrazine hydrate in alkaline medium. The catalysts were recycled up to six times, giving yields 76–92% in the model reaction among 4-chlorobenzaldehyde, 10b and 62a.

**Scheme 138:**
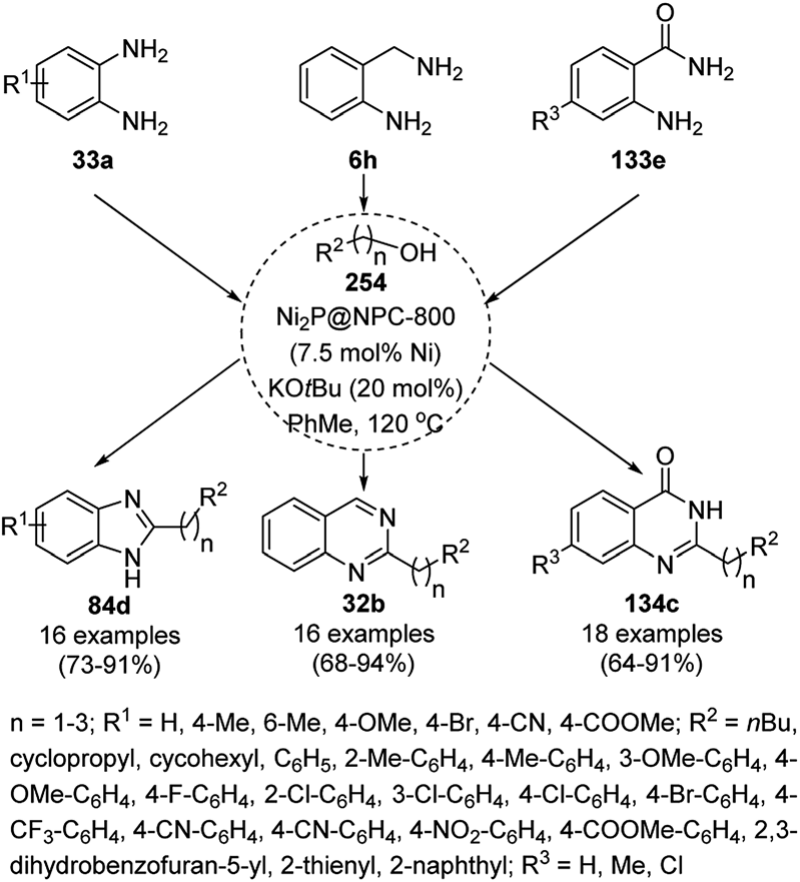
Synthesis of benzimidazoles (84d), quinazolines (32b) and quinazolinones (134c) catalyzed by NiNPs.

The Ni_2_P NP-supported biomass-derived N,P co-doped porous carbon (Ni_2_P@NPC-800)-catalyzed cross-dehydrogenative coupling of alcohols (254) with diamines (33a or 6h) or o-amino benzamides (133e) was reported for the synthesis of benzimidazoles (84d), quinazolines (32b) and quinazolinones (134c), respectively (Scheme 138).^264^ The catalyst was prepared *via* the hydrothermal and pyrolysis treatment of the biochar of bamboo shoots with Ni(OAc)_2_ and phytic acid as Ni and P sources, respectively. The control experiments gave insight into the reaction mechanism, in which the catalyst assists in the oxidation of alcohol to aldehydes and aromatization to the final compounds. The high stability of the catalyst was evident from its five times reuse without obvious loss in Ni content.

**Scheme 139:**
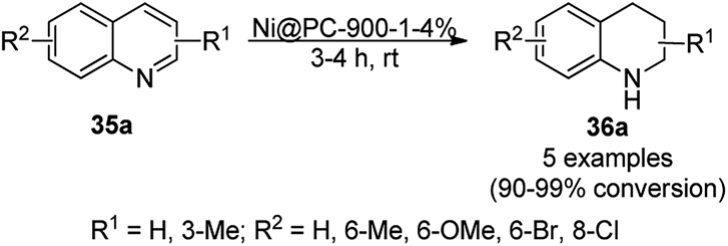
Hydrogenation of quinoline catalyzed by Ni@PC.

Zheng *et al.* synthesized novel Ni@PC-900-1-4% NCs *via* a starch-assisted confinement strategy by pyrolysis at 900 °C for 1 h having 4% Ni content for the hydrogenation of quinoline derivatives (35a) (Scheme 139) and synthesis of 1,2,3,4-terahydroquinolines (36a).^265^ The catalyst was recycled eight times without loss in its catalytic activity. The encapsulated NiNPs were anchored by the hydroxyl groups of polymeric starch, preventing the loss of Ni metal.

**Scheme 140:**
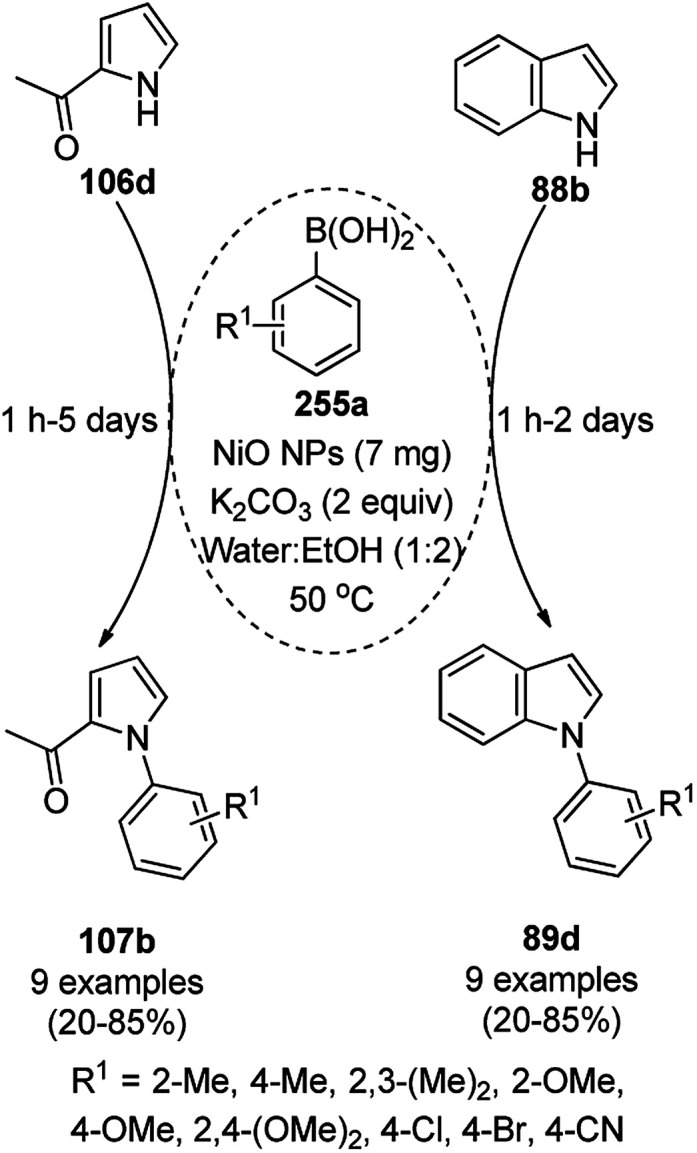
C–N cross-coupling of 106d and 88b with phenyl boronic acids catalyzed by NiO NPs.

The NiO NP-catalyzed C–N cross-coupling of 2-acetyl pyrrole (106d) and indole (88b) with substituted phenyl boronic acids (255a) was achieved in aqueous ethanol (Scheme 140) under mild conditions.^266^ A mixture of quercetin as the capping agent, NiCl_2_·6H_2_O as the metal precursor, and urea as the source of hydroxyl ions upon reaction with water was treated in a hydrothermal autoclave to obtain metal oxide NPs. The hydroxyl groups of quercetin enabled the encapsulation of the NiO NPs together with the formation of a supramolecular assembly. The NiO NPs were separated by centrifugation and recycled for up to six runs without a noticeable reduction in their catalytic performance for the synthesis of *N*-arylated heterocycles.

**Scheme 141:**
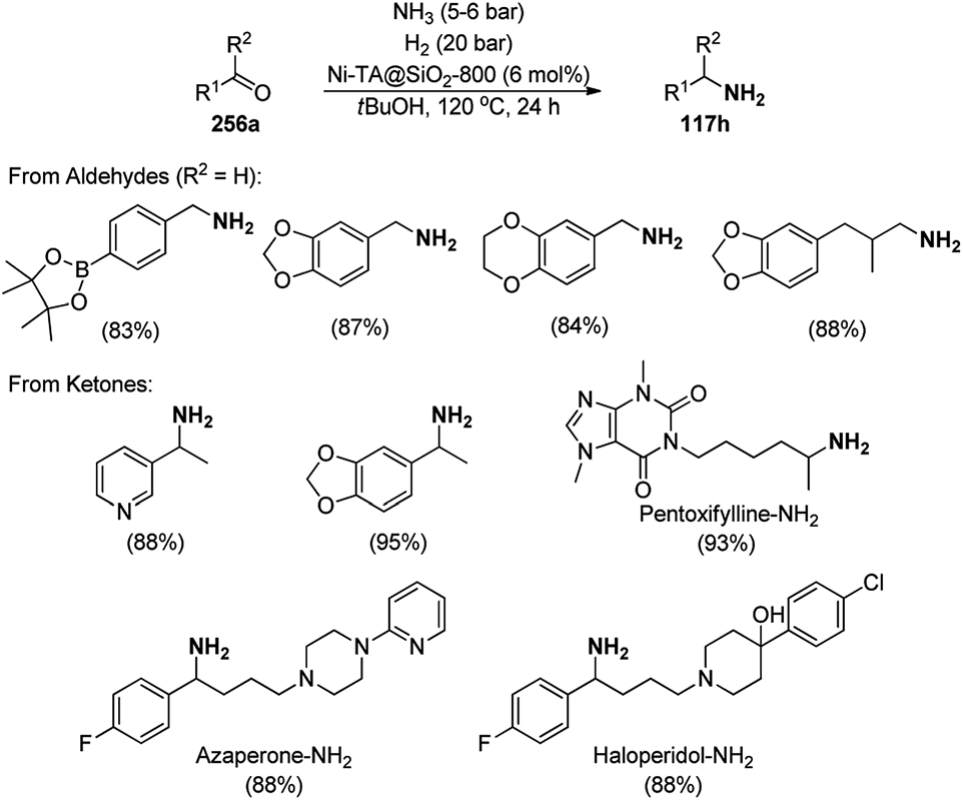
Reductive amination of aldehydes or ketones (256a) to primary amines (117h).

NCs composed of silica-supported nickel nitrate–tartaric acid (Ni-TA@SiO_2_-800) obtained by pyrolysis at 800 °C was reported as a novel catalyst for the reductive amination of aldehydes and ketones (256a) using ammonia and molecular hydrogen for the synthesis of primary amines (117h, Scheme 141) by Beller *et al.*^267^ The NPs were synthesized *via* the treatment of Ni(NO_3_)_2_·6H_2_O with tartaric acid and silica under a solvothermal process followed by pyrolysis at 800 °C. This protocol was also extended for the incorporation of amines into pharmaceutical and steroidal motifs. The stability of the catalyst as tested on a scale of up to 25 g and recycled for up to ten runs without appreciable loss in its catalytic performance.

**Scheme 142:**
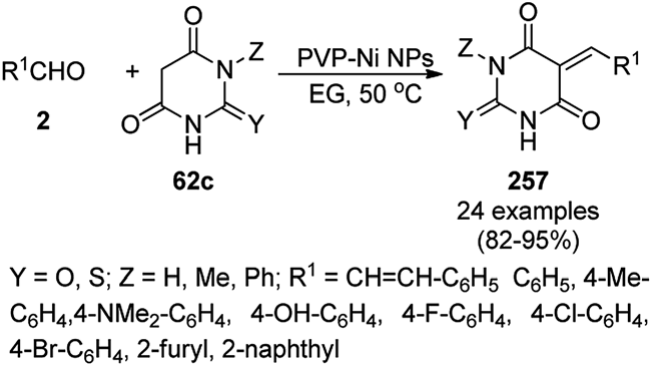
Knoevenagel condensation of aldehydes (2) and barbituric acids (62c) catalyzed by NiNPs.

Khurana *et al.* reported the polyvinyl pyrrolidone (PVP) nickel nanoparticle (NiNP)-catalyzed Knoevenagel condensation of aryl aldehyde (2) with barbituric acids (62c) in good to excellent yields using ethylene glycol (EG) as the solvent at 50 °C (Scheme 142).^268^ NiNPs were prepared *via* the reduction of nickel chloride hexahydrate NiCl_2_·6H_2_O with sodium borohydride (NaBH_4_) in EG and PVP. The metal NPs were characterized *via* TEM, HRTEM, quasi-electron light scattering (QLES), UV-Vis, EDAX, and XRD. The reusability of the NPs was demonstrated for up to six cycles; however, the authors observed an increase in the size of the NPs after the third catalytic run, as confirmed through QELS data.

**Scheme 143:**
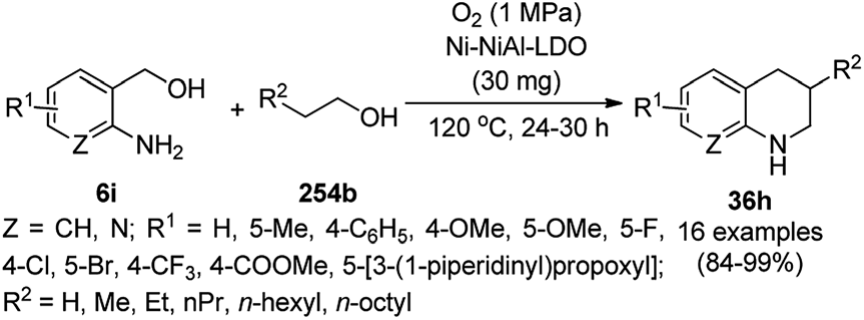
Synthesis of 1,2,3,4-terahydroquinolines (36h) catalyzed by Ni–NiAl-LDO NPs.

NiNPs supported on Ni- and Al-containing layered double oxides (Ni–NiAl-LDO) catalyzed the formation of C–C and C–N bonds in a similar fashion for the synthesis of 1,2,3,4-tetrahydroquinolines (36h) in the report by He *et al.* from 2-amino benzyl alcohols (6i) and alcohol (254b, Scheme 143) in the absence of base and molecular hydrogen.^269^ The NiNPs were synthesized using Ni(NO_3_)_2_·6H_2_O, Al(NO_3_)_3_·6H_2_O and urea *via* the urea precipitation method.^270^ The cyclocondensation of *o*-amino benzaldehyde and acetaldehyde formed by the dehydrogenation of *o*-amino benzyl alcohol (6i) and ethanol (254b) led to the formation of quinoline, which upon catalytic hydrogen transfer yielded 36h. He *et al.* screened NiNPs having a particle size in the range of 3.0–7.8 nm and found that the highest yield of 36h was obtained with the smallest particle size (3.0 nm).

### 3.7 PdNP-catalyzed synthesis of heterocycles

**Scheme 144:**
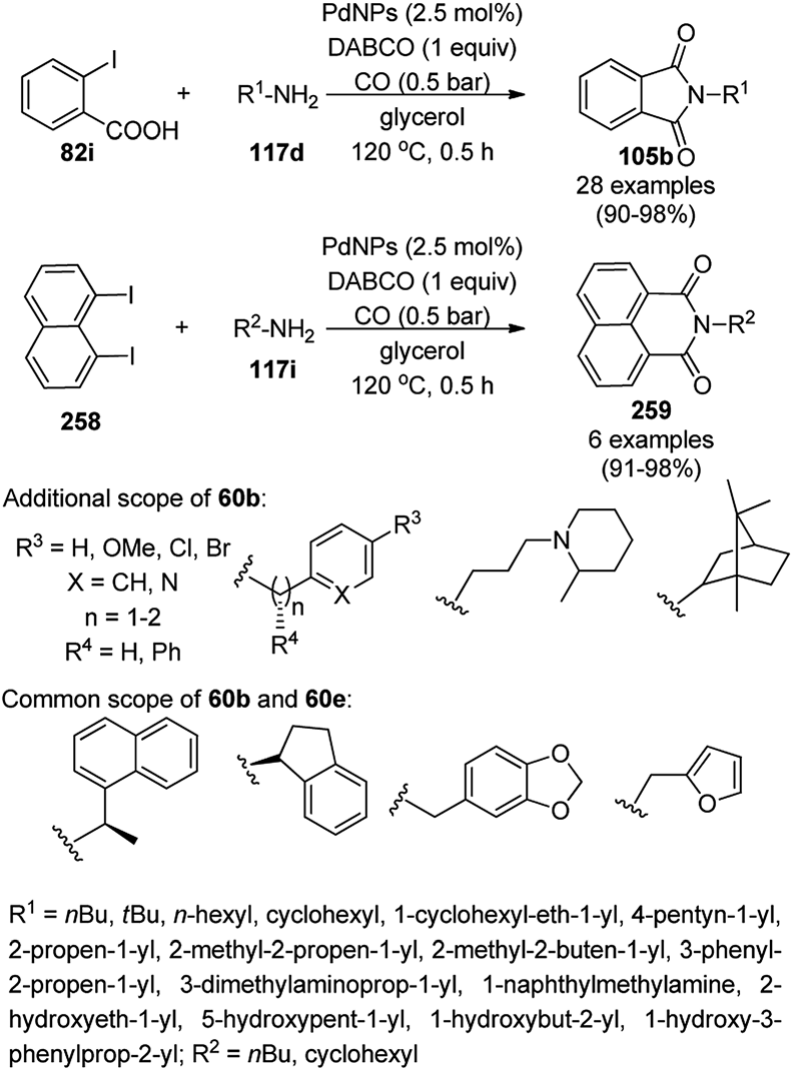
Pd-catalyzed carbonylative cyclization for the synthesis of *N*-substituted isoindol-1,3-diones (105b) and isoquinolin-1,3-diones (259).

The synthesis of *N*-substituted phthalimides (105b) was carried out with *o*-iodo benzoic acid (82i), substituted primary amine (117d) and carbon monoxide (CO) in the presence of DABCO (1,4-diazabicyclo[2.2.2]octane) using palladium nanoparticles (PdNPs) as the catalyst in glycerol (Scheme 144).^271^ The PdNPs were stabilized by tris(3-sulfophenyl)phosphine trisodium salt (TPPTS). The scope of the catalyst was extended for the synthesis of naphthalimides (259), isoindole-1-ones, tetrahydroisoquinolin-1,3-diones, (*Z*)-3-(arylmethylene)isoindolin-1-one and (*Z*)-1-methylene-1,3-dihydroisobenzofurans. The authors have claimed that the activity of the catalyst was maintained for up to ten cycles upon its recovery.

**Scheme 145:**
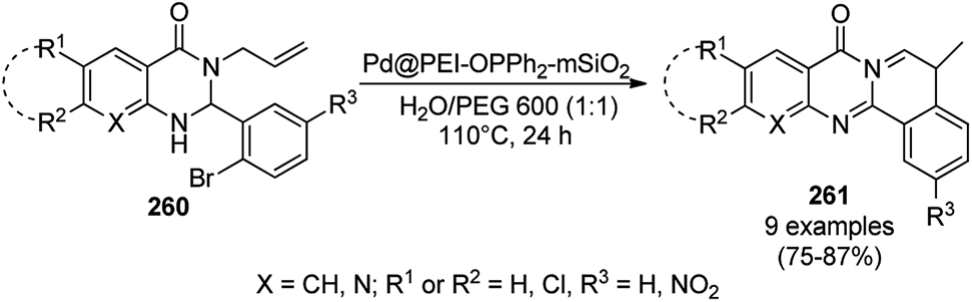
Synthesis of 5-methyl-13,13*a*-dihydro-8*H*-isoquinolino[1,2-*b*]quinazolin-8-one derivatives (261) from 3-allyl-2-(2-bromophenyl)-2,3-dihydroquinazolin-4(1*H*)-one (260).

Quinazolinone derivatives show various biological activities such as antitumor, antimicrobial, anti-inflammatory, epidermal growth factor receptor (EGFR), and tyrosine kinase inhibitory activities.^272^ The Mizoroki–Heck reaction was reported to form the carbon–carbon bond using a Pd@Ph_2_PO-PEI-mSiO_2_ catalyst to synthesize 5-methyl-13,13a-dihydro-8*H*-isoquinolino[1,2-*b*]quinazolin-8-one (261) from 3-allyl-2-(2-bromophenyl)-2,3-dihydroquinazolin-4(1*H*)-one (261) using H_2_O–PEG 600 (1 : 1) as a co-solvent at 110 °C (Scheme 145).^273^ The superparamagnetic NPs were coated with silica followed by the further functionalization of silica with PEI-silane and Pd loading using palladium acetate. These NPs were characterized *via* HRTEM, VSM, TGA, FT-IR, and ICP-AES analysis. PEI plays a dual role as a water dispersant and organic base. The recycling of the Pd@Ph_2_PO-PEI-mSiO_2_ catalyst was tested in 10 sequential reactions for the preparation of 5-methyl-8*H*-isoquinolino[1,2-*b*]quinazolin-8-one without significant loss in its activity due to its high stability.

**Scheme 146:**
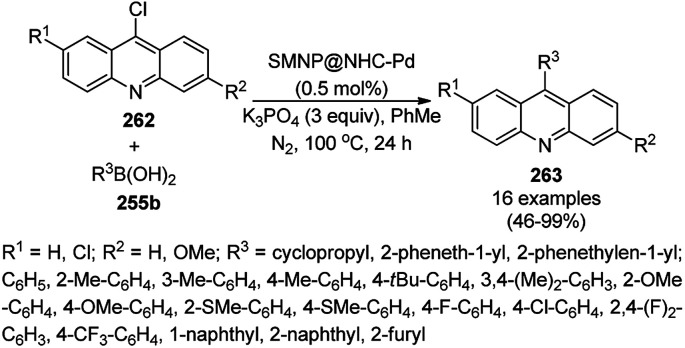
Synthesis of 9-phenylacridine (263) from 9-chloroacridine (262) using SMNP@NHC–Pd as the catalyst.

The Suzuki–Miyaura cross-coupling of substituted 9-chloroacridine (262) with aryl boronic acids (255b) was reported by Tu *et al.* in the presence of silica-coated magnetic nanoparticles supported on N-heterocyclic carbene-palladacycle (SMNP@NHC–Pd) in a low catalytic loading using K_3_PO_4_ as a base in toluene under an N_2_ atmosphere at 100 °C for 24 h to give 9-aryl acridine (263, Scheme 146).^274^ The nanoparticles were reused five times without significant loss in their catalytic performance. In contrast, this Suzuki–Miyaura cross-coupling reaction was attempted using various Pd catalysts such as Pd(OAc)_2_, PdCl_2_ and Pd_2_(dba)_3_, but no significant yield of 9-aryl acridine was observed.

**Scheme 147:**
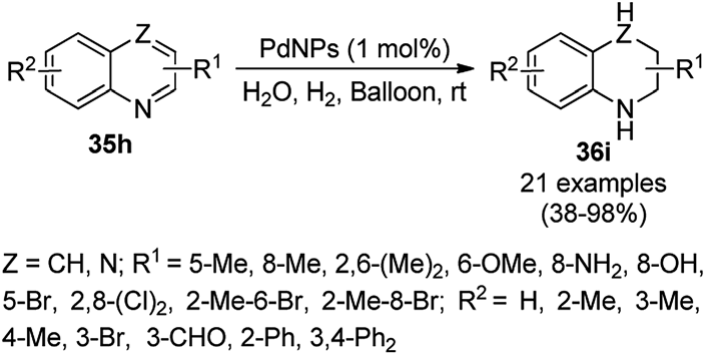
Synthesis of 1,2,3,4-tetrahydroquinolines or 1,2,3,4-tetrahydroquinoxalines (36i) from quinolines and quinoxalines (35h) using PdNPs.

PdNPs supported on an organic framework were also reported to have applications in the catalytic hydrogenation of N-heterocyclic compounds.^275^ Accordingly, Wu *et al.* reported the efficient hydrogenation of substituted quinolines and quinoxalines (35h) catalyzed by PdNPs stabilized by carbon-metal bonds in aqueous solution under mild conditions at rt. The synthesis of the PdNPs was carried out *via* the *in situ* reduction of Pd(OAc)_2_ by NaBH_4_ after the addition of 1,1′-binaphthyl,-2,2′-bis(diazonium tetrafluoroborate) to THF-MeOH (Scheme 147). The dispersion of these NPs was confirmed through TEM and HR-TEM. Further, they were characterized using FT-IR, ICP, XPS, and XRD. This catalyst was demonstrated to catalyze the reaction without a significant loss in yield for up to five catalytic cycles. In the presence of water as the solvent, the rate of hydrogenation was accelerated since water forms the key hydrogen bridges between the nitrogen of the heterocycles and PdNPs, bringing them in close proximity with each other. This demonstrated the synergistic effects of particle size and water medium to accelerate the hydrogenation reaction.^276^

**Scheme 148:**
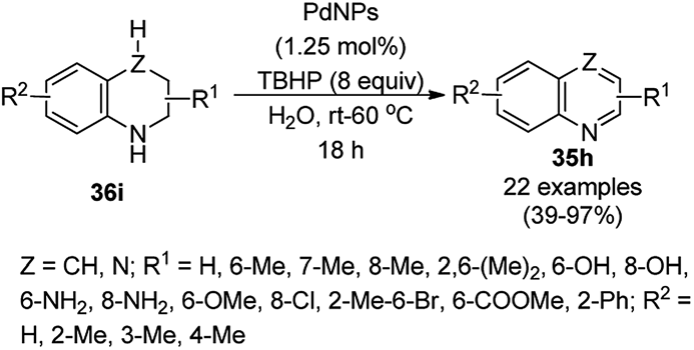
Synthesis of quinolines or quinoxalines (35h) catalyzed by PdNPs.

Wu *et al.* reported the catalytic use of PdNPs for the dehydrogenation of 1,2,3,4-tetrahydroquinolines and 1,2,3,4-tetrahydroquinoxalines (36i) for the synthetic construction of quinolines and quinoxalines (35h, Scheme 148) using *tert*-butyl hydroperoxide as an oxidizing agent in water.^277^ They were also found to be successful for the synthesis of acridines, 1,10-phenanthroline and benzoquinolines from their hydro-counterparts.

**Scheme 149:**
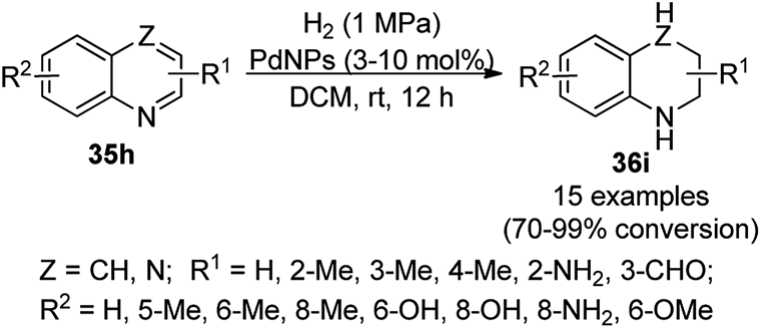
Hydrogenation of quinoline and quinoxalines (35h) catalyzed by PdNPs.

Metal-carbon stabilized PdNPs were reported by Wu *et al.* for the catalytic hydrogenation of quinoline and quinoxalines derivatives (35h) to synthesize 1,2,3,4-tetrahydroquinolines or quinoxalines (36i, Scheme 149) in good to high conversion.^278^ The synthesis of the NPs was achieved using a catalytic loading of Pd using Pd(OAc)_2_ on the diazotized product of mono-acetylated 1,1′-binaphthyl-2,2′-diamine in the presence of sodium borohydride as a reducing agent. Further, the particulate integrity of the NPs was characterized *via* TEM and ICP.

**Scheme 150:**
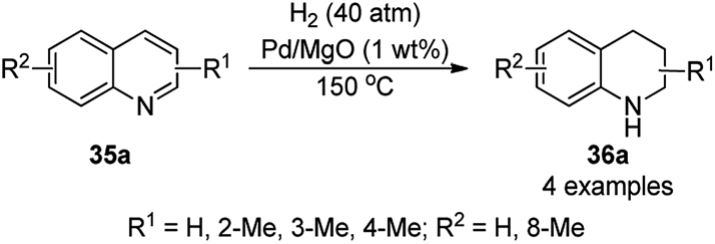
Pd/MgO-catalyzed hydrogenation of quinolines (35a) using a Parr hydrogenator.

The PdNP-anchored MgO (Pd/MgO)-catalyzed hydrogenation of quinolines (35a) was reported by Delgado *et al.* (Scheme 150) using a Parr hydrogenator with a high TOF (250–310 h^−1^).^279^ They also used these NPs as catalysts for the hydrogenation of alkenes and biodiesel. MgO and disodium tetrachloropalladate (Na_2_PdCl_4_) were mixed followed by chemical reduction using sodium borohydride to synthesize NPs, which were characterized *via* ICP-AES, TEM, XRD, and XPS. The catalyst was recycled up to three times for the catalytic hydrogenation of cyclohexene.

**Scheme 151:**
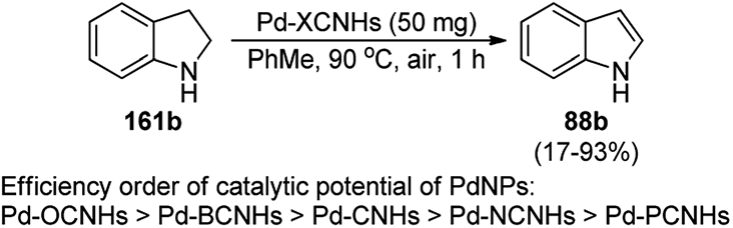
Catalytic dehydrogenation of indolines (161b) catalyzed by PdNPs supported on CNHs.

Shi *et al.* reported the catalytic dehydrogenation of indolines (161b) to indoles (88b) in toluene at 90 °C using a catalytic amount (50 mg, Scheme 151) of PdNPs anchored on heteroatom such as oxygen, boron, nitrogen and phosphorus doped carbon nanohorns (Pd-XCNHs, X = O, B, N, and P).^280^ The heteroatom-doped CNHs (XCNHs) were loaded with K_2_PdCl_4_ followed by chemical reduction using sodium tetraborohydride to obtain Pd-XCNHs, which were characterized *via* TEM, ICP, XPS, BET, XRD, FT-IR, and TGA. However, authors did not study the recyclability of the catalyst to reveal its durability.

**Scheme 152:**
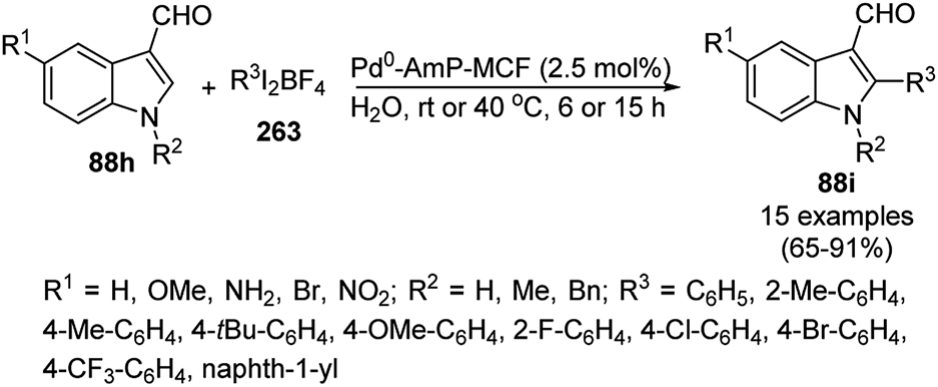
PdNP-catalyzed synthesis of 2-phenyl indoles (88i) *via* C–H activation.

Olofsson *et al.* reported the synthesis of C-2 selective arylation of substituted indoles (88h) using PdNPs supported on amino-functionalized mesocellullar foam (Pd0-AmP-MCF) and diaryliodonium tetrafluoroborate salts (263) under aqueous conditions at rt or 40 °C for 6 or 15 h (Scheme 152).^281^ They optimized the synthesis of substituted indoles using various diphenyliodonium salts (263), solvents and reaction times. The optimized catalytic loading of PdNPs was found to be 2.5 mol%, yielding the product in 91%. This reaction was found to be successful with different electron-donating and withdrawing groups. The reaction was studied with diphenyl, arylphenyl and diaryl iodonium tetrafluoroborate salts; however, in the case of arylphenyl iodonium tetrafluoroborate salts, phenyl was transformed into indole in lower yields. The catalyst was recovered and recycled up to three times with a gradual decrease in its catalytic activity.

**Scheme 153:**
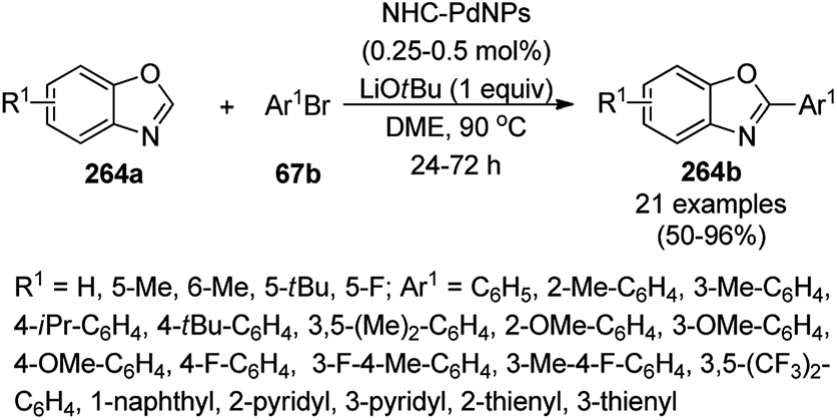
Direct C-2 arylation of benzoxazole (264a) catalyzed by PdNPs.

The C–H activation of heterocycles such as indoles, benzazoles, and adenosine can be easily achieved using PdNPs, which are considered active species rather than moribund species.^282^ The Csp^2^–H activation of benzo[*d*]oxazoles (264b) was reported by Li *et al.* to be catalyzed by pincer NHC-nitrogen-phosphine-chelated Pd(ii)complexes (NHC-PdNPs) (Scheme 153) using lithium *tert*-butoxide as a base in dimethoxyethane (DME) as the solvent at 90 °C.^283^ This protocol was performed using an ultra-low amount of catalyst without the requirement of Cu additive and excess base. Mechanistically, 2-isocyanophenolate formed by deprotonation and ring-opening of 264a underwent complexation with the Pd(ii) species, intramolecular nucleophilic addition and reductive elimination to yield 264b.

**Scheme 154:**
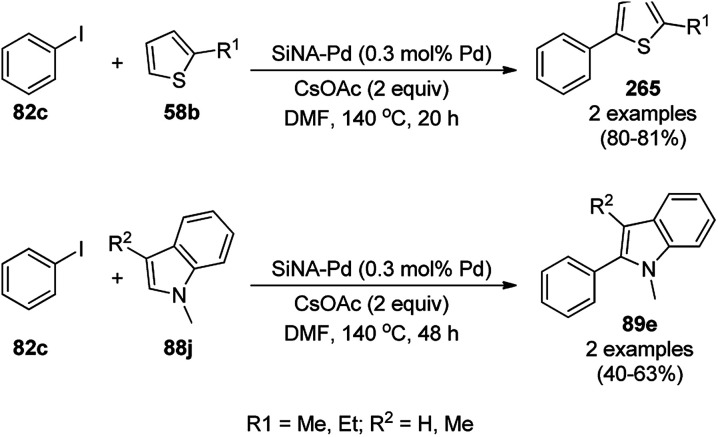
C–H activation of thiophenes (58b) and indoles (88j) reported by Yamada *et al.*

Yamada *et al.* reported the C–H activation of thiophenes (58b) and indoles (88j) catalyzed by silicon nanowire array-stabilized palladium nanoparticles (SiNA-PD) as a heterogeneous catalyst from iodobenzene (82c) as the coupling agent, DMF as the solvent and caesium acetate as the base (Scheme 154).^284^ For the preparation of the nanocatalyst, p-type Si wire was treated with H_2_SO_4_, and H_2_O_2_ following the loading of AgNPs using AgNO_3_. The silicon nanowire array (SiNA) was generated *via* the treatment of Si wafers loaded with AgNPs with HF. Subsequently, SiNA was treated with potassium tetrachloropalladate (K_2_PdCl_4_) for the loading of Pd. The prepared NPs were characterized *via* SEM, SEM/EDX, XPS, TEM, XANES, and FTEXAFS. The authors have also explored the use of the developed catalyst for Mizoroki–Heck coupling, hydrogenation of olefin, hydrogenolysis of nitro-aryls, and hydrosilylation of α,β-enones.

**Scheme 155:**
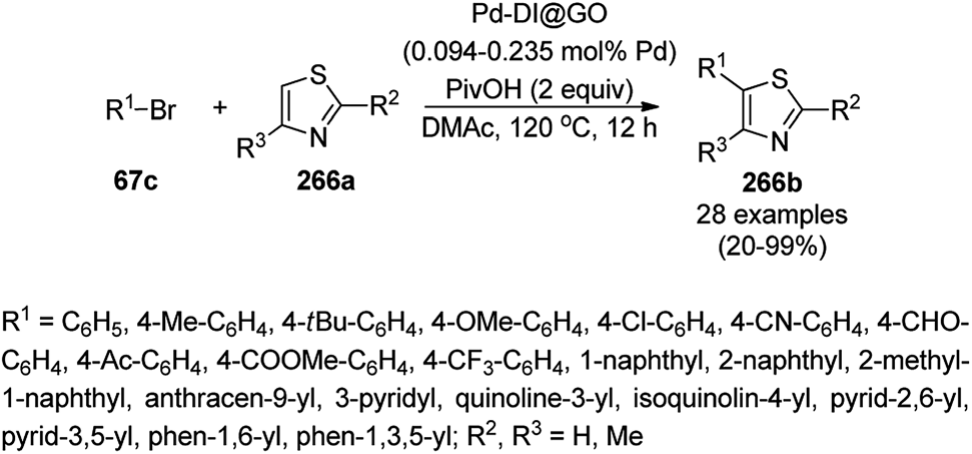
Direct C–H arylation of thiazoles (266a) with bromoarenes (67c) catalyzed by Pd-DI@GO.

Li *et al.* reported the catalytic use of a palladium diamine complex supported on GO (Pd-DI@GO) for the direct C–H activation of thiazole (266a) with bromoarenes (67c, Scheme 155) for the synthesis of substituted thiazoles (266b).^285^ The same catalyst was also explored for the Suzuki coupling of bromoarenes with substituted phenyl boronic acids in moderate to excellent yields. The catalyst was separated *via* filtration or centrifugation and recycled up to four times without loss in its catalytic performance for the Suzuki coupling reaction. However, after the fourth catalytic run, its activity decreased dramatically because of the agglomeration of the GO layers, diminishing the contact between the PdNPs and the reactants (SEM).

**Scheme 156:**
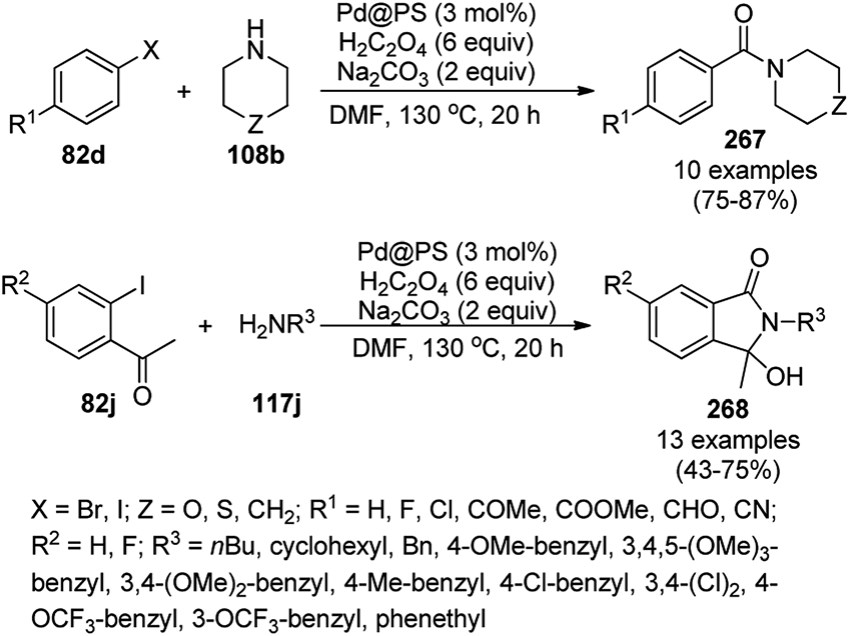
Aminocarbonylation of aryl halides (82d/82j) catalyzed by PdNPs.

Das *et al.* reported the novel synthesis of heterocyclic amides (267) using oxalic acid as an *ex situ* source of a CO gas double layer-vial (DLV) system *via* the aminocarbonylation of aryl halides (82d) with amines (117j) using a palladium nanocatalyst immobilized on polystyrene (Pd@PS) in DMS at 130 °C and Na_2_CO_3_ as the base (Scheme 156).^286^ This protocol involving Pd@PS was further explored for the synthesis of iso-indolinone derivatives (268) from *o*-iodo acetophenones (82j) and aryl alkyl or alkyl amines (117j). Aminocarbonylation of aniline with iodobenzene produced *N*-phenyl benzamide in 73% yield using DLV single vessel with screw cap (*ex situ*) conditions, whereas *in situ* formation of CO yielded the target compound in 10% yield together with the formation of side products. The Pd@PS NPs were synthesized *via* a reduction and deposition approach using Amberlite IRA 900 Cl^−^ resin and palladium acetate Pd(OAc)_2_, and characterized *via* SEM and TEM. These NPs were recycled for up to four catalytic cycles without loss in their catalytic performance, as evident from the TEM image of the recycled NCs after the fourth catalytic run.

**Scheme 157:**
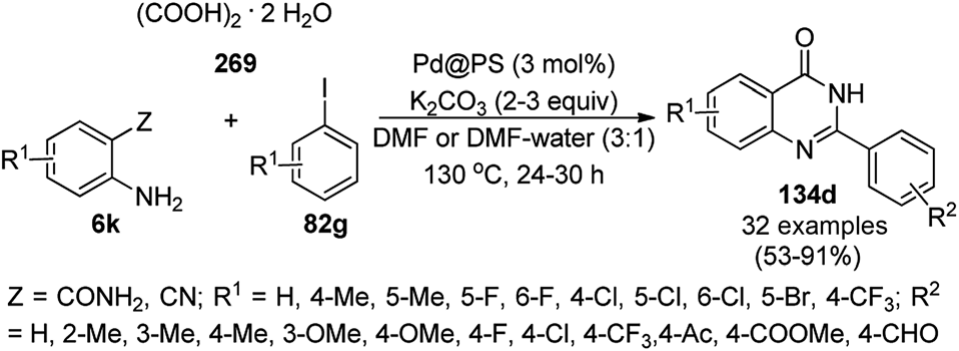
Synthesis of 2-aryl quinazolinones (134d) catalyzed by Pd@PS.

Further, Das *et al.* reported the use of the same Pd@PS catalyst for the synthesis of 2-aryl quinazolinones (134d) using *o*-aminobenzamides or *o*-aminobenzonitriles (6j), oxalic acids (269) as the carbon monoxide (CO) donor and aryl iodides (82g, Scheme 157).^287^ This protocol was reported to be free from the use of an autoclave and CO gas under pressurized conditions.^288–291^ While assessing the durability of the catalyst, 16% loss in the yield of 134d (R^1^, R^2^ = H) was observed in the fifth run compared to that in the first run (91% yield).

**Scheme 158:**
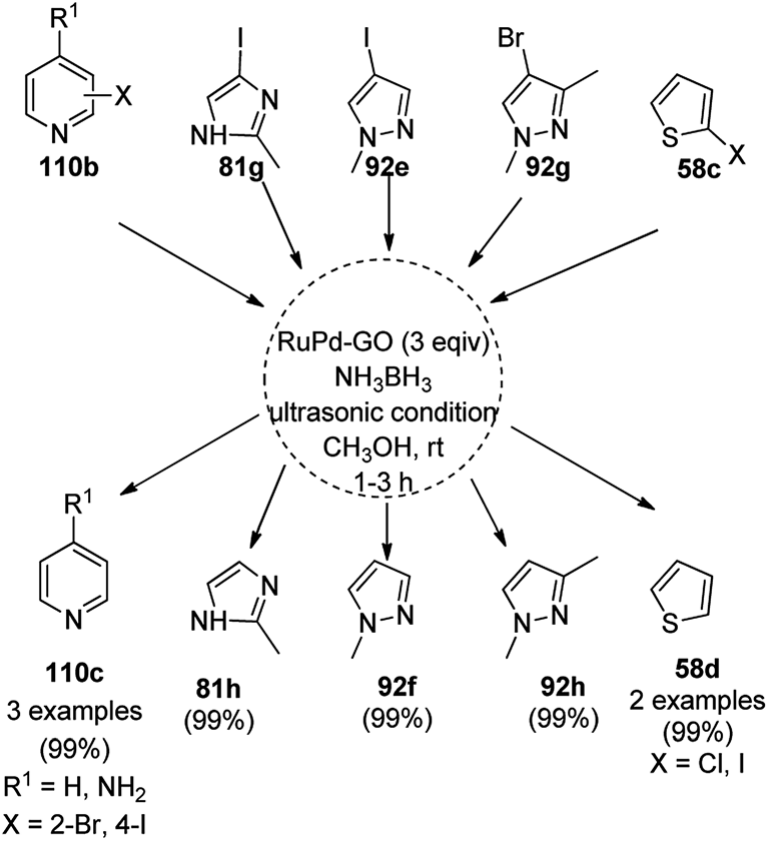
Dehalogenation of aryl halides using PdNPs.

To reduce the toxicity of halogenated compounds, dehalogenation is a key requirement. Accordingly, Göksu *et al.* reported Ru/Pd NPs supported on monodispersed graphene oxide (RuPd-GO) as a highly efficient catalyst for the synthesis of pyridine (110b), imidazoles (81g), pyrazole (92e/g) and thiophene (58c) under ultrasonic conditions in methanol at rt in the presence of ammonium borane (Scheme 158).^292^ A similar protocol was also explored for dehalogenation of other haloarenes. The synthesis of NPs was achieved using graphene oxide, RuCl_3_, and PdCl_2_ in ethanol at 100 °C *via* a microwave-assisted method, which was further characterized *via* various methods such as TEM, HRTEM, XRD, and XPS. The catalyst was recycled several times during the synthetic operations. The comparison of this RuPd-GO catalyzed protocol with reported works^293–295^ on the debromination of bromobenzene revealed that the present protocol exhibits the advantage of higher yields, as confirmed by GC, together with the use of less solvent, shorter reaction time, and lower temperature.

**Scheme 159:**
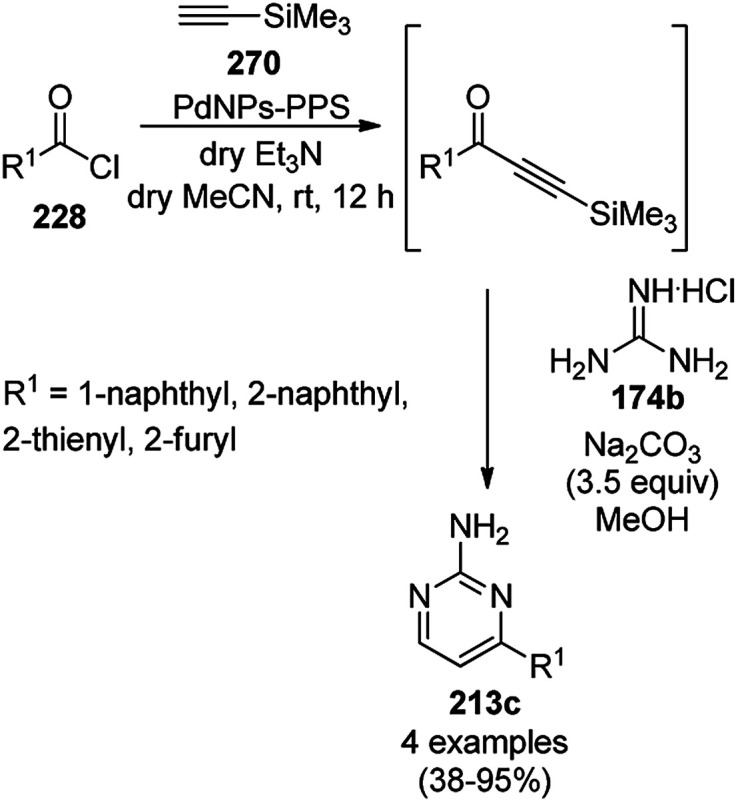
One-pot synthesis of N-containing heterocyclic compounds (213c) catalyzed by PdNPs.

**Scheme 160:**
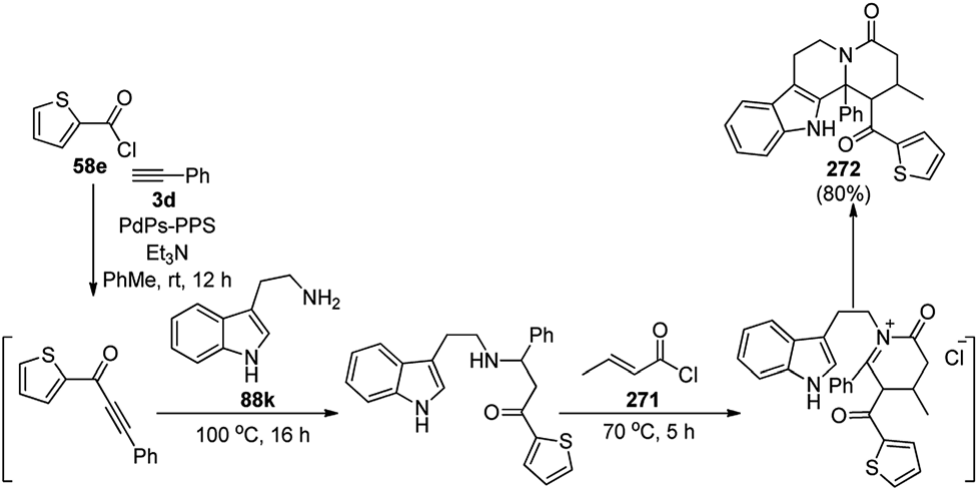
One-pot synthesis of tetrahydro-β-carboline compounds (272) catalyzed by PdNPs.

Mandal *et al.* reported copper-free Sonogashira coupling for the one-pot synthesis of 2,4-disubstituted pyrimidines (213c/272) catalyzed by PdNPs loaded on a polymer matrix of PPS [poly(1,4-phenylene sulfide)] from aryl or heteroaryl carbonyl chloride (228) and terminal acetylenes (270) in acetonitrile and triethylamine as the base to form a ynone intermediate, which was further reacted with guanidine hydrochloride (174b) as the coupling reagent and sodium carbonate in methanol to yield the final product (213c, Scheme 159).^296^ The synthesized ynone intermediate was further explored for the synthesis of tetrahydro-β-carboline compounds (272, Scheme 160) *via* its treatment with tryptamine (88k) followed by methacryloyl chloride (271) *via* amination, aza-annulation, and Pictet–Spengler reaction of the intermediate (Scheme 160). The PdNPs were synthesized by loading Pd using Pd(OAc)_2_ at 95 °C in PhMe with PPS and been further characterized *via* XPS, TEM, and powder XRD. The reusability of the catalyst was studied with thiophene-2-carbonyl chloride (58e) and phenyl acetylene (3d) for up to four catalytic runs, and its catalytic activity was found to decrease gradually.

**Scheme 161:**
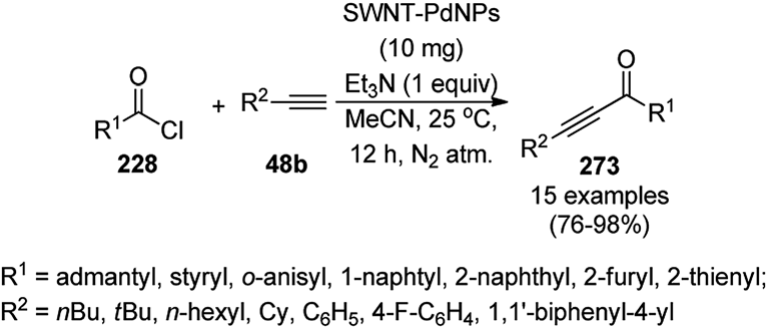
Acyl Sonogashira reaction catalyzed by SWNT-PdNPs.

**Scheme 162:**
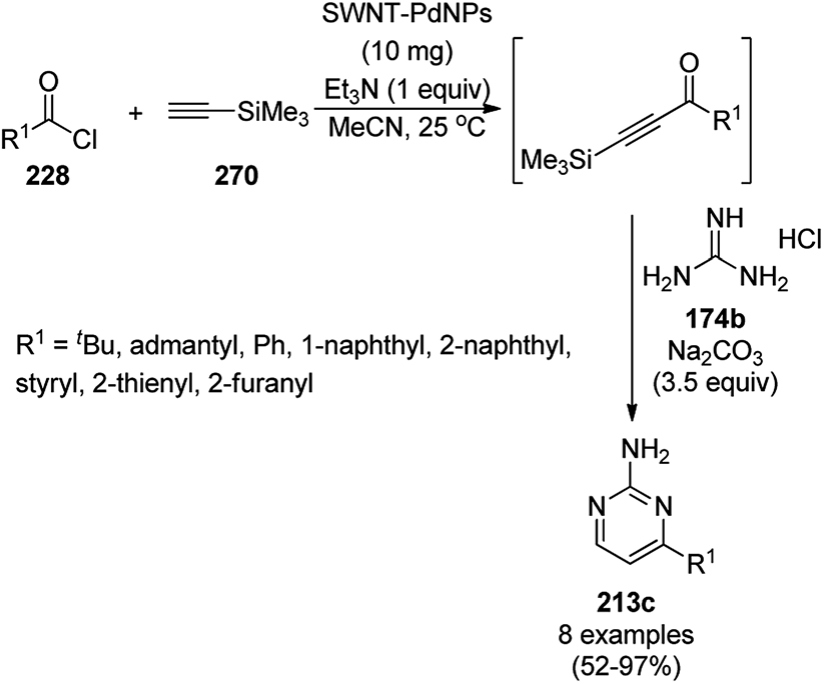
One-pot synthesis of 2,4-disubstituted pyrimidines (213c).

The PdNP-anchored single-walled CNT (SWNT-PdNPs)-catalyzed acyl Sonogashira reaction for the synthesis of ynones (273) from acyl chloride (228) and alkyne (48b) in acetonitrile was successfully achieved by Mandal *et al.* (Scheme 161).^297^ The same catalyst was also used for the synthesis of TMS-ynones and explored for the one-pot synthesis of 2-aminopyrimidines (213c) from the reaction among 228, trimethylsilyl alkynes (270) and guanidine hydrochloride (174b) in moderate to excellent yields (Scheme 162) following a similar approach as that in Scheme 159. SWNT-PdNPs was obtained *via* the pyrolysis of carboxylic acid-functionalized SWNT with Pd salts such as palladium acetate Pd(OAc)_2_ in DMF at 95 °C for 4 h. The recovered catalyst was reused up to seven times with slight loss in its activity.

**Scheme 163:**
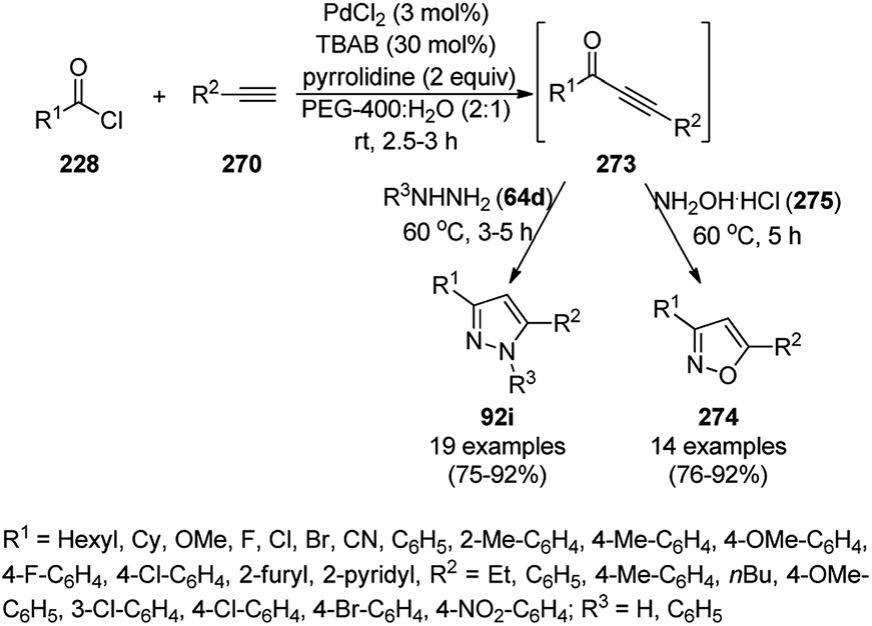
Synthesis of pyrazoles (92i) and oxazoles (274) catalyzed by *in situ*-formed PdNPs.

*In situ*-formed PdNP-catalyzed copper-free acyl Sonogashira coupling followed by intramolecular 5-*endo*-dig cyclization was reported for the one-pot synthesis of substituted pyrazoles (92i) and isoxazoles (274, Scheme 163) in PEG-water.^298^ The treatment of PdCl_2_ with PEG-400 resulted in the formation of Pd(0)NPs, which in turn reacted with 228 and 270 in the presence of TBAB (tetrabutyl ammonium bromide) as a phase transfer catalyst or stabilizer and pyrrolidine as a base to form a ynone intermediate (273). In the same pot, the addition of substituted hydrazine (64d) or hydroxylamine hydrochloride (275) led to the regioselective synthesis of 92i or 274, respectively, *via* palladation with regenerated Pd(0)NPs. The catalyst was recycled up to five times with consistent yields of 92i (R^1^, R^3^ = Ph; R^2^ = *p*-anisyl). Hg and CS_2_ poisoning tests revealed that homogenous catalysis was likely to be predominant in the synthesis of 92i and 274.

**Scheme 164:**
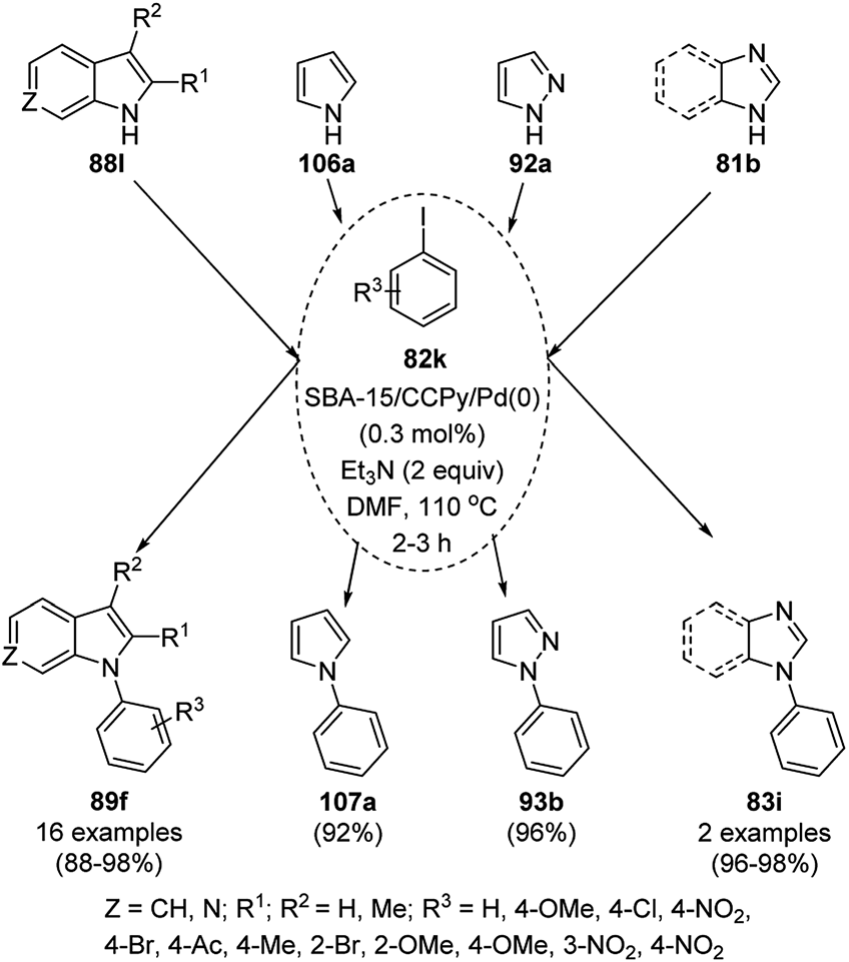
*N*-arylation of N-containing heterocycles catalyzed by Pd(0)NPs.

The catalytic use of SBA-15-functionalized melamine-pyridine group-supported Pd(0)NPs [SBA-15/CCPy/Pd(0)NPs] paved the way for the successful *N*-arylation of N-containing heterocycles such as indoles and azaindoles (88l), pyrrole (106a), pyrazole (92a), and imidazole/benzimidazole (81b) using iodobenzenes (82k) as aryl coupling partners and triethylamine as the base at 110 °C (Scheme 164).^299^ Ullmann coupling was reported with this catalyst without the requirement of an inert atmosphere and it was recycled up to seven times without loss in its catalytic activity. The attractive attributes of this protocol such as superior catalytic activity, ease of recovery, stability of catalysts as proven by the hot filtration test, and selective *N*-arylation *versus* C-arylation make it a green and sustainable protocol.

**Scheme 165:**
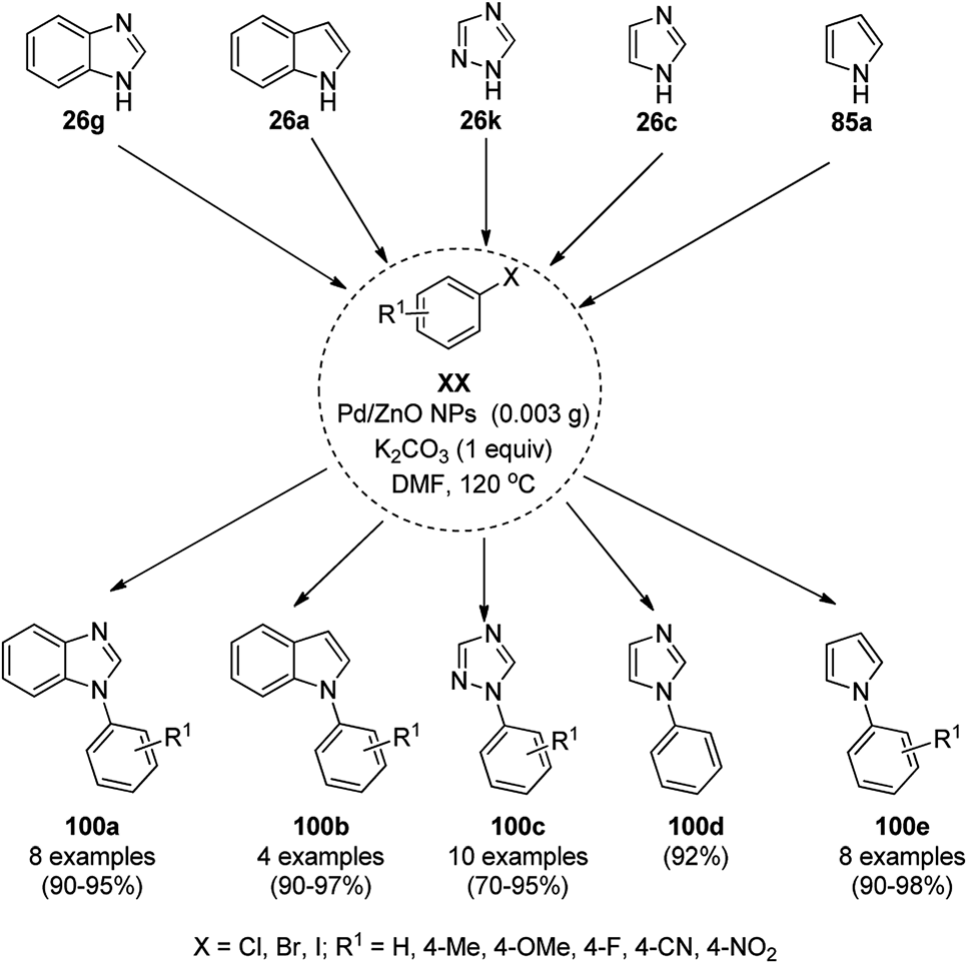
C–N coupling of azoles with aryl halides catalyzed by Pd/ZnO NPs.

Hosseini-Sarvari *et al.* reported palladium supported on ZnO NPs (Pd/ZnO NPs) as a new catalyst for the synthesis of *N*-arylated heterocyclic compounds from N-containing heterocycles such as benzimidazoles (26g), indoles (26a), triazoles (26k), imidazoles (26c), and pyrroles (85a) in DMF as the solvent and K_2_CO_3_ as the base (Scheme 165).^300^ The Pd/ZnO NPs were prepared *via* the co-precipitation of Zn(NO_3_)_2_·6H_2_O and Pd(NO_3_)_2_·2H_2_O with NaOH and characterized *via* SEM, XPS, TEM, TGA, ICP, XRD and AAS. The same protocol was also extended for the *O*-arylation of substituted phenols with haloarenes. The reusability of the Pd/ZnO NPs was studied for the *O*-arylation of NPs for up to five catalytic runs, and the catalyst retained its catalytic activity and nanoparticulate integrity (XRD).

**Scheme 166:**
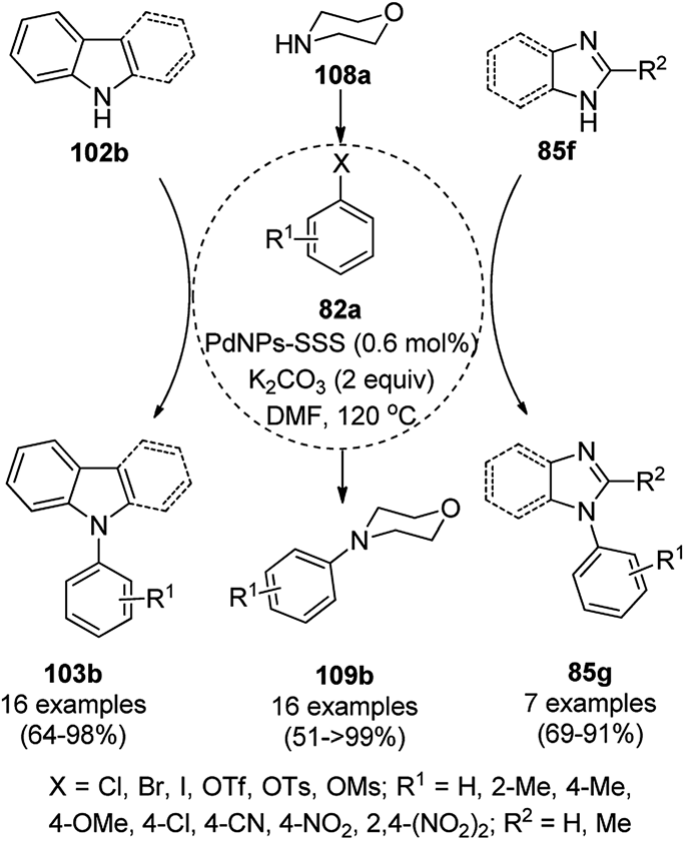
C–N cross coupling of aza-heterocycles catalyzed by PdNPs-SSS.

Silica chloride obtained *via* the treatment of silica with thionyl chloride was reacted with starch to yield a silica-starch substrate followed by the loading of Pd using palladium acetate to synthesize PdNPs immobilized on silica-starch substrate (PdNPs-SSS).^301^ PdNPs-SSS catalyzed the Buchwald–Hartwig *N*-arylation of indoles or carbazoles (102b), morpholines (108a) and (benz)imidazoles (85f) successfully using halobenzenes or phenyl triflate/tosylate/mesylates (82a, Scheme 166). The heterogeneous PdNPs were separated by filtration and reused for up to five catalytic runs with 89–92% yield of *N*-phenyl indole.

**Scheme 167:**
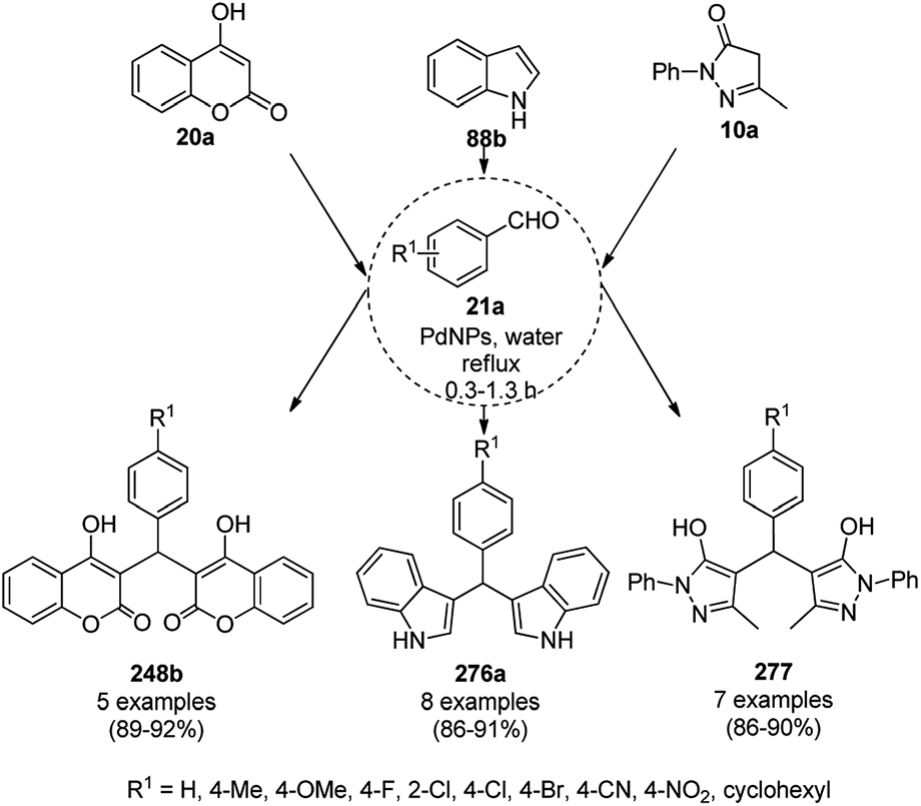
Green synthesis of bis(heterocyclyl)methanes in water catalyzed by PdNPs.

Pal *et al.* synthesized bis(heterocyclyl)methanes *via* the reaction of substituted benzaldehyde (21a) with 4-hydroxycoumarin (20a)/indole (88b)/3-methyl-1-phenyl-1*H*-pyrazol-5(4*H*)-one (10a) catalyzed by Pd(0)NPs in water under reflux in 86–92% yield (Scheme 167).^302^ The PdNPs were synthesized using palladium chloride (PdCl_2_), tetrabutyl ammonium bromide (TBAB) as a stabilizer and sodium carbonate (Na_2_CO_3_) as the base, and their particle size (20–50 nm) was determined *via* TEM and powder XRD. The yields of the final product obtained using the PdNPs were found to be superior in comparison with other reported protocols involving the catalytic use of molecular iodine (I_2_),^303^ phosphotungstic acid,^304^ and silica-supported sodium hydrogen sulfate (NaHSO_4_·SiO_2_)^305^ for the synthesis of bis(heterocyclyl) methanes. The effect of the solvent on the yield of the product was investigated, and aqueous conditions were found to be better than nonpolar solvents. The catalyst retained its catalytic activity for up to four catalytic runs. The PdNCs acted as a Lewis acid catalyst and promoted Knoevenagel condensation/Michael addition *via* the activation of carbonyl oxygen to bring the electrophile and nucleophile in close proximity.

**Scheme 168:**
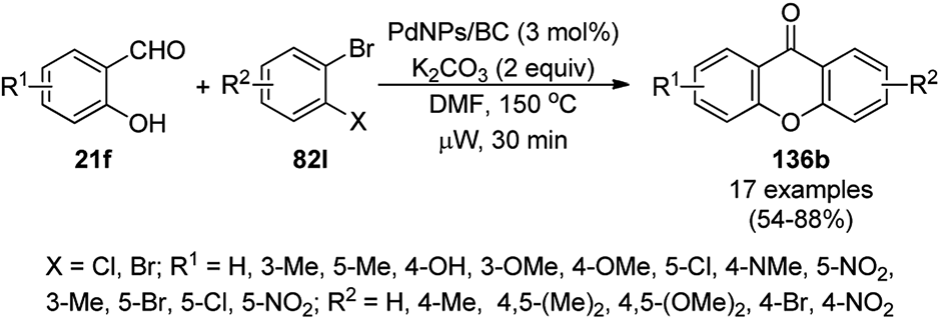
Synthesis of xanthones catalyzed by PdNPs.

Gerbino and co-workers reported the synthesis and characterization of efficient, novel and recyclable PdNPs anchored on green biochar (PdNPs/BC) through a precipitation-reduction method employing the use of PdCl_2_ as the precursor, biochar as the support, and the pyrolytic product of lignocellulosic biomass.^306^ The were employed in the microwave-mediated, ligand- and additive-free, regioselective synthesis of xanthones (136b) from substituted salicylaldehydes (21f) and *o*-bromo haloarenes (82l) (Scheme 168). The PdNPs/BC NCs were separated by filtration and reused for up to four cycles in 88–82% yield, where they maintained their initial activity, as confirmed by AAS and XRD.

**Scheme 169:**
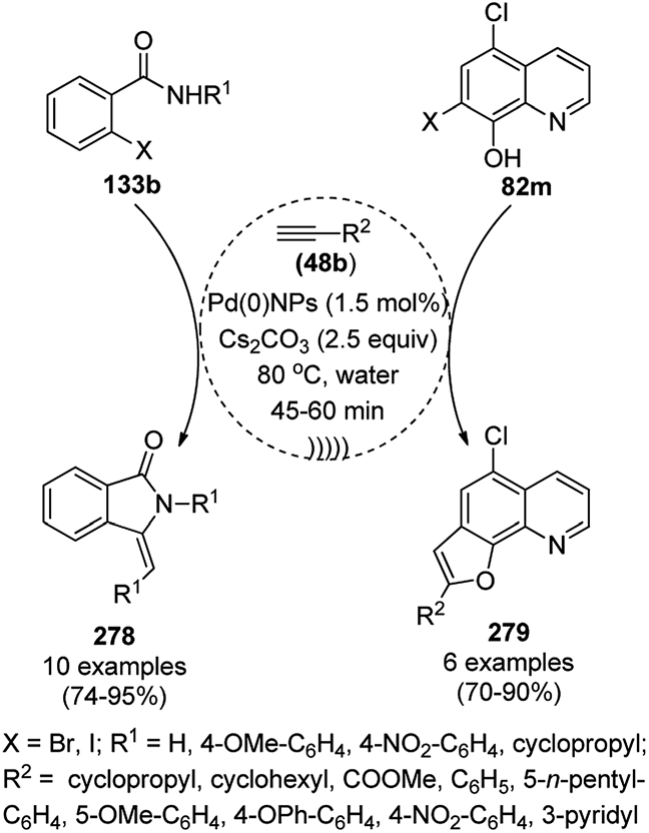
Regioselective and stereoselective synthesis of (*Z*)-3-methyleneisoindoline-1-ones (278) and furo[3,2-*h*]quinolines (279).

In 2016, Sarkar *et al.*^307^ synthesized PdNPs *via* the reduction of H_2_PdCl_4_ using hydroxylamine and d-glucose as a reducing agent and stabilizer, respectively. Further, they also investigated their catalytic potential for the one-pot domino Sonogashira-cyclisation (Scheme 169) of terminal alkynes (48b) with 2-halido-*N*-arylbenzamides (133b) for the stereoselective and regioselective synthesis of (*Z*)-3-methyleneisoindoline-1-ones (278). The same protocol was also extended for the synthesis of furo[3,2-h]quinolines (279) with 5-chloro-7-halo-8-hydroxy quinolines (82m). The PdNPs were recovered under centrifugation and recycled for up to five cycles, resulting in yields in the range of 95–87%, where the yield of 80% achieved in the sixth was attributed to the agglomeration of the NPs.

**Scheme 170:**
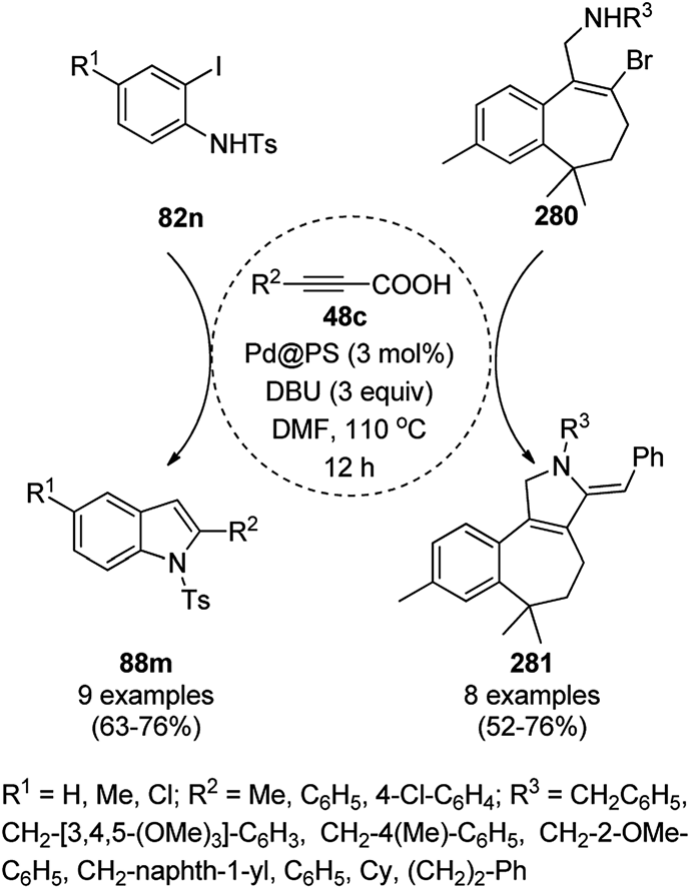
Pd@PS-catalyzed synthesis of 1,2-disubstituted indoles (88m) and 3-pyrolines (281).

The synthesis of PdNPs supported on polystyrene (Pd@PS) was reported by Das *et al. via* a reduction–deposition approach using Amberlite IRA 900 resin and palladium salt (Pd(OAc)_2_).^308^ This catalyst was used successfully for the synthesis of indoles (88m, Scheme 170) and 3-pyrolines (281) *via* the sequential decarboxylative coupling-cyclization of alkynyl carboxylic acids (48c) with aryl iodides (82n), and amino benzocycloheptene bromides (280), respectively. The amalgamation of the catalyst due to the addition of a drop of mercury (mercury drop test) stooped the progress of the reaction, thus revealing that the reaction occurred truly in a non-heterogeneous fashion.

**Scheme 171:**
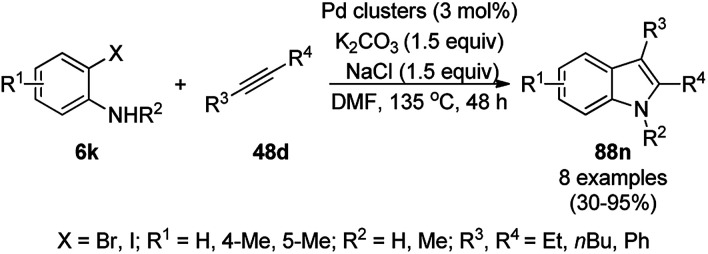
Larock indole synthesis catalyzed by Pd clusters.

DMF-stabilized Pd nanoclusters in a catalytic amount (3 mol%) were reported by Obora *et al.* for Larock indole ligand-free synthesis using *o*-haloanilines (6k) and symmetrical and asymmetrical alkynes (48d, Scheme 171) in DMF at 135 °C.^309^ They synthesized the Pd-nanoclusters *via* the reduction of PdCl_2_ with DMF following their previously reported protocols.^310^ The catalyst was recycled three times with a slight loss in activity, yielding 80%, 68%, and 63% of 88n (R^1^, R^2^ = H; R^3^, and R^4^ = Ph) in three consecutive cycles due to the leaching of the metal catalyst (ICP).

**Scheme 172:**
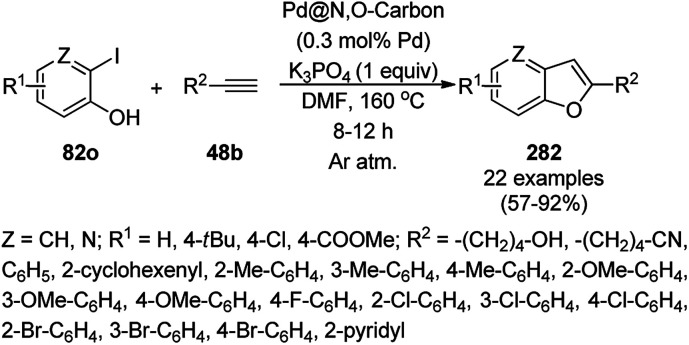
Synthesis of benzofurans catalyzed by PdNPs.

Nitrogen- and oxygen-doped porous carbons were prepared *via* the hydrothermal treatment and carbonization of finely powdered bamboo shoot. Subsequently, the porous carbons were loaded with Pd using Pd(NO_3_)_2_ and hydrazine as the reducing agent *via* an ultrasound-assisted reduction method to obtain the final NPs (Pd@N,O-Carbon).^311^ These NPs were used in a catalytic amount for the synthesis of 2-iodophenol (82o) and alkynes (48b) for the synthesis of 2-substituted benzofurans (282, Scheme 172) using potassium phosphate in DMF under an inert atmosphere. The durability of the catalyst was tested *via* recycling experiments with up to five runs without a notable reduction in the yield of benzoxazoles.

**Scheme 173:**
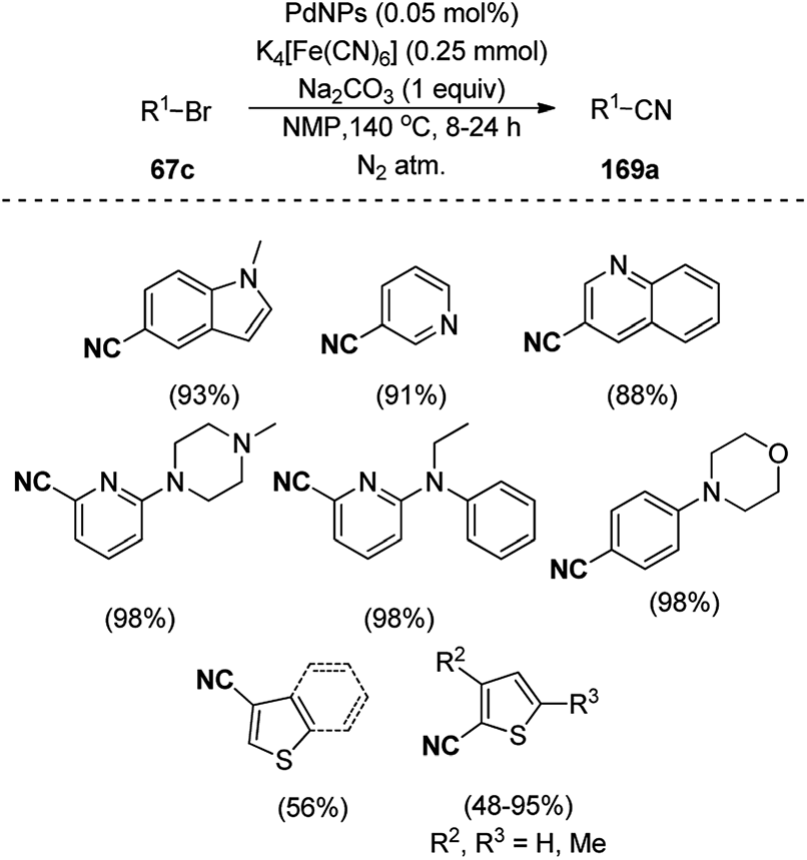
PdNP-catalyzed cyanation of bromo derivatives.

PdNPs (dichloro[bis{1-(dicyclohexylphosphanyl)piperidine}]palladium) obtained by the treatment of dichloro(1,5-cyclooctadiene)palladium(ii) with 1-(dicyclohexylphosphanyl)piperidine was reported by Frech *et al.* as a catalyst for the cyanation of bromo compounds (67c) using potassium ferrocyanide K_4_[Fe(CN)_6_] as the cyanating agent to synthesize cyano derivatives (Scheme 173).^312^ The catalytic performance of these NPs was reported to be superior compared to Pd salts for the cyanation of aryl and heteroaryl bromides.

**Scheme 174:**
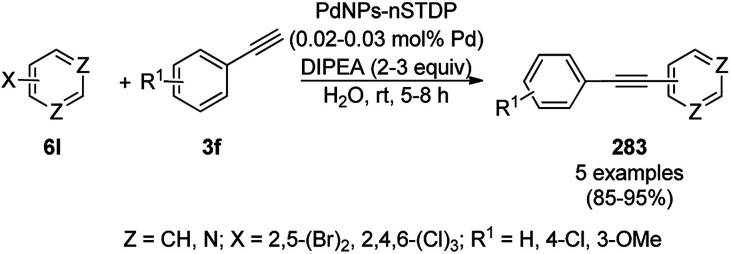
Sonogashira cross-coupling catalyzed by PdNPs-nSTDP.

The PdNP-supported nanosilica triazine dendritic polymer (PdNPs-nSTDP)-catalyzed Sonogashira cross-coupling of 2,6-dibromopyridine or 2,4,6-trichloromopyrimidine (6l) with substituted phenyl acetylenes (3f) was achieved successfully for the green synthesis of V- or star-shaped di- or trialkynylaromatics (283), respectively (Scheme 174) under aqueous conditions at rt using *N*,*N*-diisopropyl ethylamine (DIPEA) as the base.^313^ The same catalyst was also reported for the Suzuki–Miyaura cross-coupling, Heck coupling^314^ and C–S coupling.^315^ PdNPs-nSTDP was recycled and reused for up to ten times with 80–95% yield of the Sonogashira-coupled product of bromobenzene and phenylacetylene.

### 3.8 RuNP-catalyzed synthesis of heterocycles

**Scheme 175:**
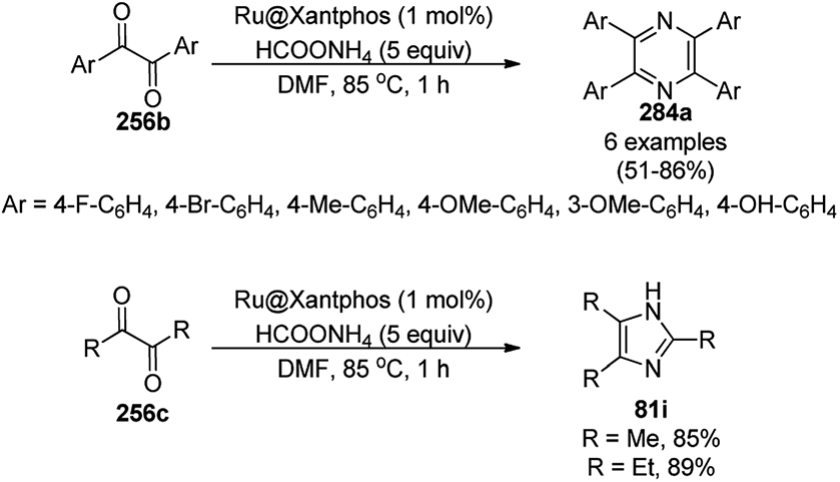
Phosphine-supported RuNP-catalyzed synthesis of substituted pyrazines (284a) and imidazoles (81i) from α-diketones.

van Leeuwen *et al.* reported phosphine-supported ruthenium nanoparticles (NPs) for the synthesis of substituted pyrazines (284a) and imidazoles (81i) from commercially available α-diketones (256b/c, Scheme 175).^316^ The RuNPs were reported to be a hydrogenation catalyst with a low catalyst loading (1 mol%), which could be removed by their adsorption on silica or alumina. This method was applied for the synthesis of a key intermediate of the marine cytotoxic natural product dragmacidin B.

**Scheme 176:**
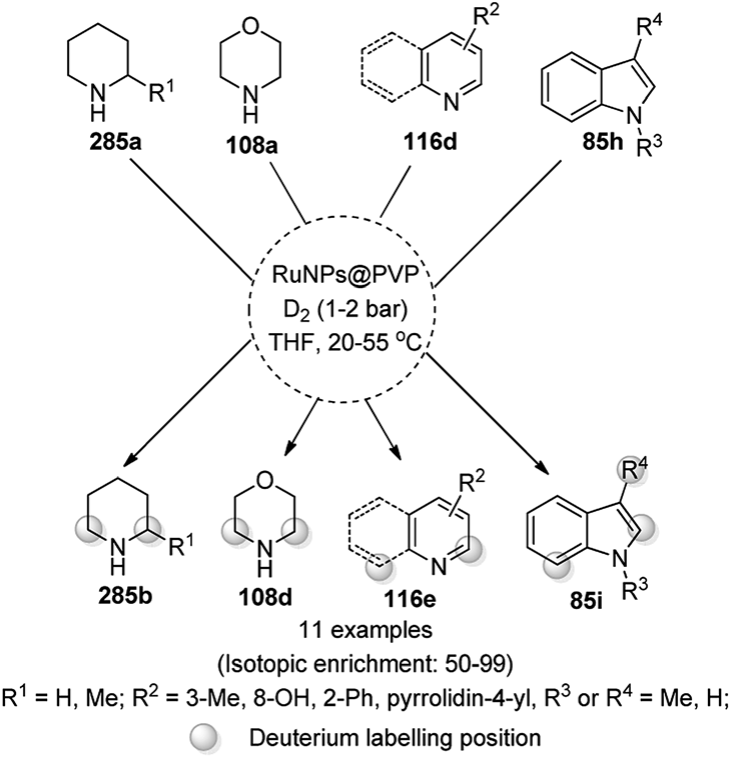
RuNP-catalyzed regioselective deuteration of N-containing heterocycles.

Rousseau *et al.* reported the RuNP-catalyzed regioselective and regiospecific deuteration of N-containing heterocyclic compounds such as piperidine (285a), morpholine (108a), quinoline or pyridine (116d) and benzimidazole (85h, Scheme 176).^317^ The developed protocol was successfully employed *via* the deuteration of many biologically active compounds such as nicotine, anabasine, papaverine, melatonin, dextromethorphan, imipramine, protriptyline and paroxetine in good yield with high chemo- and regioselectivities. The authors claimed that hydrogen–deuterium exchange was observed due to the direct coordination of nitrogen to ruthenium since they did not observe any deuteration in the oxygen donor group.

**Scheme 177:**
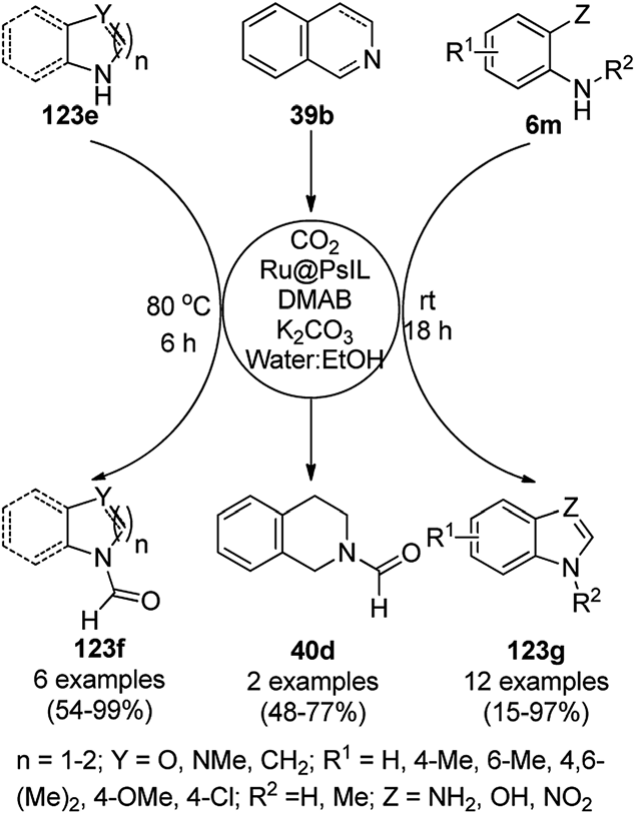
Ru@PsIL-catalyzed synthesis of *N*-formamides (123f/40d) and benzazoles (123g).

Bhanage *et al.* reported that ruthenium nanoparticles supported on polymeric ionic liquids (Ru@PsIL) catalyzed the synthesis of *N*-formamides (123f/40d) and benzazoles (123g) such as benzimidazole or benzoxazole from carbon dioxide and dimethylamine borane (DMAB) in water : ethanol (1 : 1) as a green solvent and K_2_CO_3_ as the base (Scheme 177).^318^ A polymeric ionic liquid (PsIL) were synthesized from Merrifield peptide resin and 1,2-dimethyl-1*H*-imidazole, which was treated with RuCl_3_ and NaBH_4_ for the immobilization of RuNPs onto PsIL. The good recyclability of the developed catalyst was observed up to the fifth catalytic run without loss in catalytic activity, and a negligible amount of leaching was observed. The high stability of the catalyst was confirmed from the SEM and TGA analysis of the recycled catalyst at the end of the fifth catalytic cycle. The same protocol was found to be successful with the formylation of many aromatic, alicyclic aliphatic and heterocyclic amines. The developed protocol gave the best results for benzimidazole from *o*-phenylene diamines and *o*-nitroanilines, but gave benzoxazole in poor yield.

**Scheme 178:**
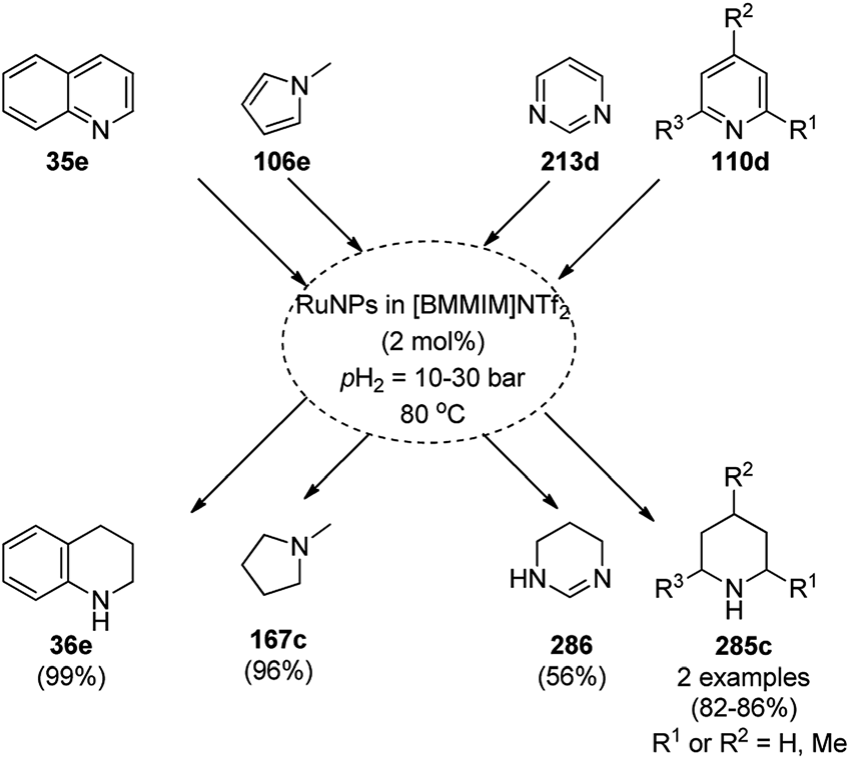
RuNPs in IL-catalyzed selective hydrogenation of N-containing aromatic heterocycles.

Prechtl *et al.* reported the selective hydrogenation of nitrogen-containing heterocycles using RuNCs in ionic liquids (Scheme 178).^319^ The selective hydrogenation of aromatic heterocycles is still a great challenge since sometimes it leads to either fully or partially hydrogenated products. For example, the hydrogenation of quinoline can lead to the formation of both 1,2,3,4-tetrahydroquinoline and 5,6,7,8-tetrahydroquinoline. The authors selectively optimized the reaction conditions to synthesize 1,2,3,4-tetrahydroquinoline (36e) in up to 99% yield using RuNPs in hydroxyl-functionalized ILs [C_1_C_1_(EG)IM]NTf_2_/[BMMIM]NTf_2_ at 80 °C. The reusability of the nanocatalyst was claimed by the authors without decay in its catalytic potential for up to six catalytic runs. The substrate scope of the reaction was also studied for other nitrogen-containing heterocycles such as pyrrole (106e), pyrimidine (213d), pyridine (110d), indole, 2-phenylpyridine, 1-phenyl-1*H*-pyrazole, and carbazole.

**Scheme 179:**
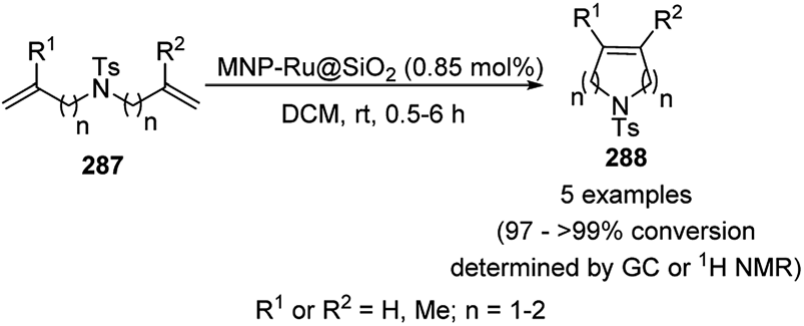
MNP-Ru@SiO_2_-catalyzed ring-closing metathesis of dienes (288).

Further Lee *et al.* reported the ring-closing metathesis of acyclic dienes (287), leading to the formation of nitrogen-containing unsaturated cyclic rings using dichloromethane as the solvent at rt in the presence of a catalytic amount of ionic magnetic NPs-supported Grubbs–Hoveyda MNP-Ru@SiO_2_ catalyst (Scheme 179).^320^ After the completion of the reaction, the recovery of the NPs was achieved using an external magnet, resulting in a clear reaction mixture. The recyclability of the catalyst was demonstrated by the authors for up to fourteen catalytic cycles without significant loss in its activity.

**Scheme 180:**
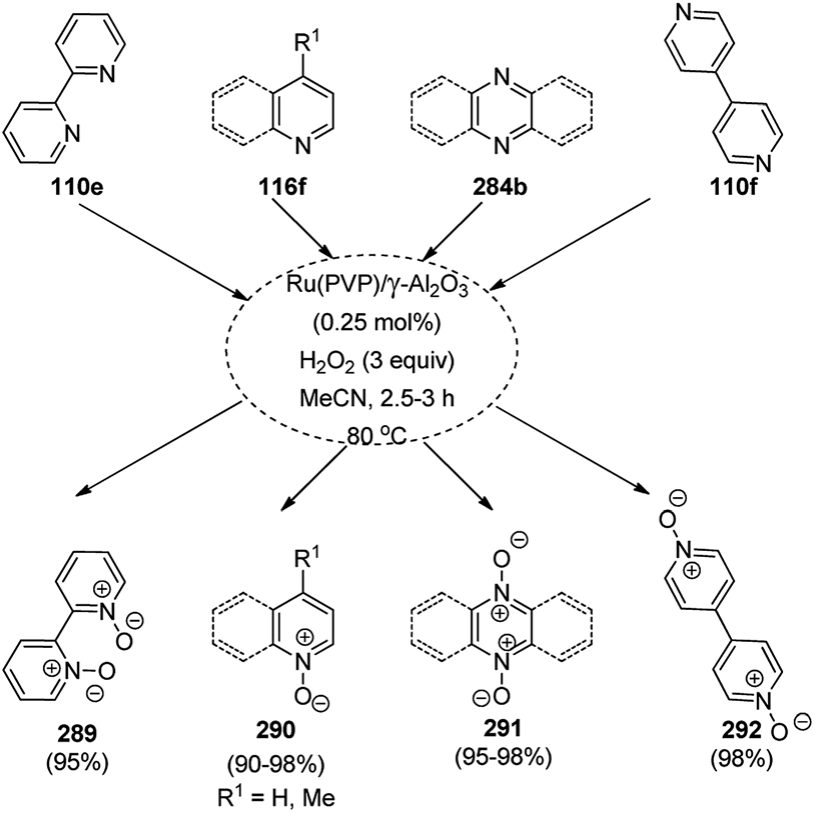
*N*-oxidation of tertiary aromatic azacyclic amines.

For the first time, RuNPs immobilized on γ-Al_2_O_3_ catalyzed the oxidation of tertiary heteroaromatic amines for the synthesis of corresponding *N*-oxides, which was reported by Rajagopal *et al.* (Scheme 180).^321^ This protocol was investigated successfully for the oxidation of 2,2′-bipyridine (110e), pyridine/quinoline (116f), pyrazine/quinoxalines/phenazine (284b), 4,4′-bipyridine (110f) and other non-heteroaromatic amines such as dimethyl anilines, and triphenyl amine. The catalyst was recycled three times without any considerable loss in its activity. These tertiary amines first bind to the surface of the RuNPs followed by interaction with H_2_O_2_ to oxidize the N-heterocycles. The present protocol was claimed to be more feasible, greener, and highly stable in water–acetonitrile compared to the other reported catalysts for the *N*-oxidation of pyridines such as silica-supported vanadium (V_*x*_Si_4*x*_O_6.4*x*_),^322^ RuCl_3_,^323^ RuCl_3_/bromamine-T,^324^ Au–Al_2_O_3_,^325^ [(C_18_H_37_)_2_(CH_3_)_2_N]_7_[PW_11_O_39_],^326^ and K_6_[PW_9_V_3_O_40_]·4H_2_O.^327^

### 3.9 SiNP-catalyzed synthesis of heterocycles

**Scheme 181:**
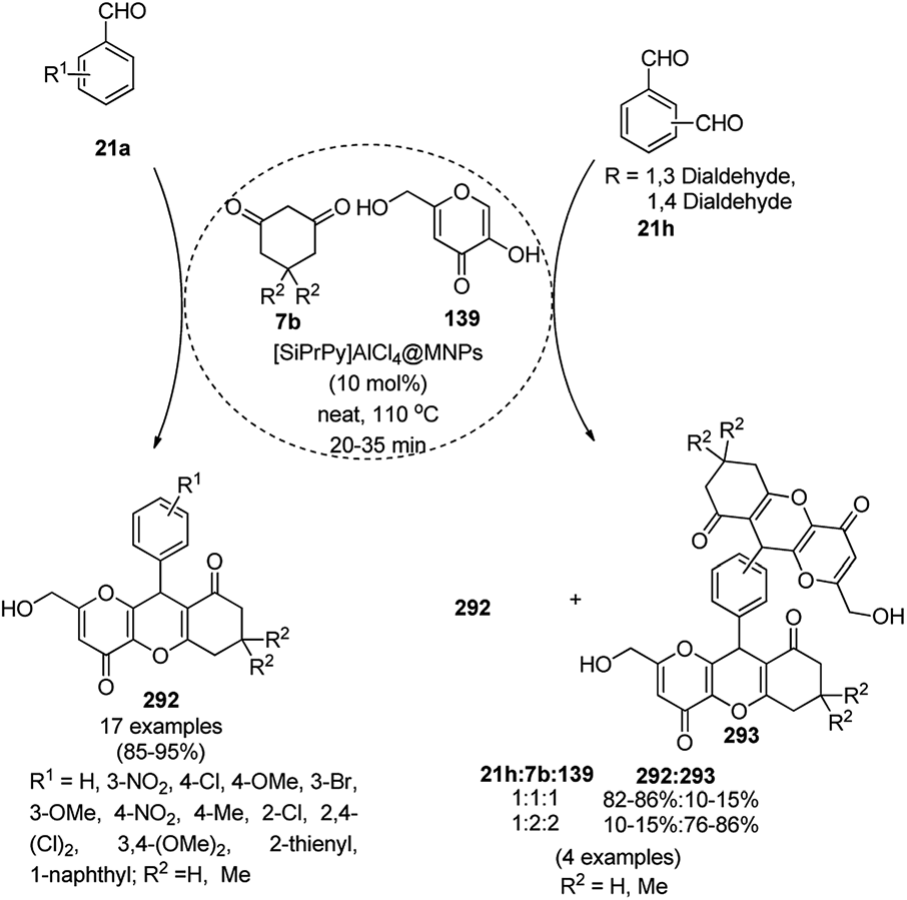
[SiPrPy]AlCl_4_@MNP-catalyzed one-pot synthesis of dihydropyrano[3,2-*b*]chromenediones (292/293) using three component of aromatic aldehydes (21a), 1,3-diones (7b) and Kojic acid (139).^329^

Varieties of silica-based NPs have been reported for the synthesis of biologically significant heterocycles for C–C and C–heteroatom bond formation.^328^ Estakhri *et al.* synthesized dihydropyrano[3,2-*b*]chromenediones (292) from aromatic aldehydes (21a), 1,3-diones (7b) and Kojic acid (139) catalyzed by chloroaluminate ionic liquid-modified silica-coated [SiPrPy]AlCl_4_ MNPs (10 mol%) at 110 °C under solvent-free conditions (Scheme 181).^329^ They also finely tuned the proportion of dialdehydes, 1,3-dione and Kojic acid (1 : 2 : 2) to selectively give bis-dihydropyrano[3,2-*b*]chromenediones (293) in 76–86% yield and all the reactants in a 1 : 1 : 1 proportion yielded 82–86% of dihydropyrano[3,2-*b*]chromenediones (292). The activity of the catalyst was found to be retained even after eight catalytic cycles with the recycled catalyst.

**Scheme 182:**
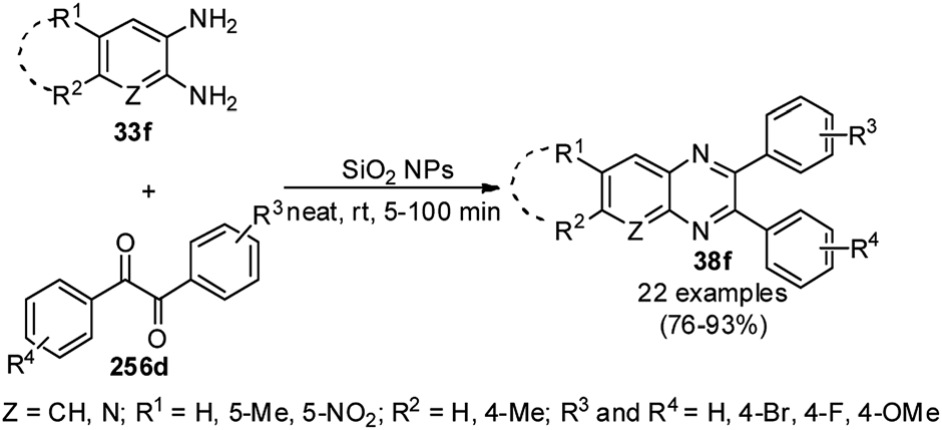
SiO_2_ NP-catalyzed synthesis of quinoxalines (38f) under solvent-free conditions.

**Scheme 183:**
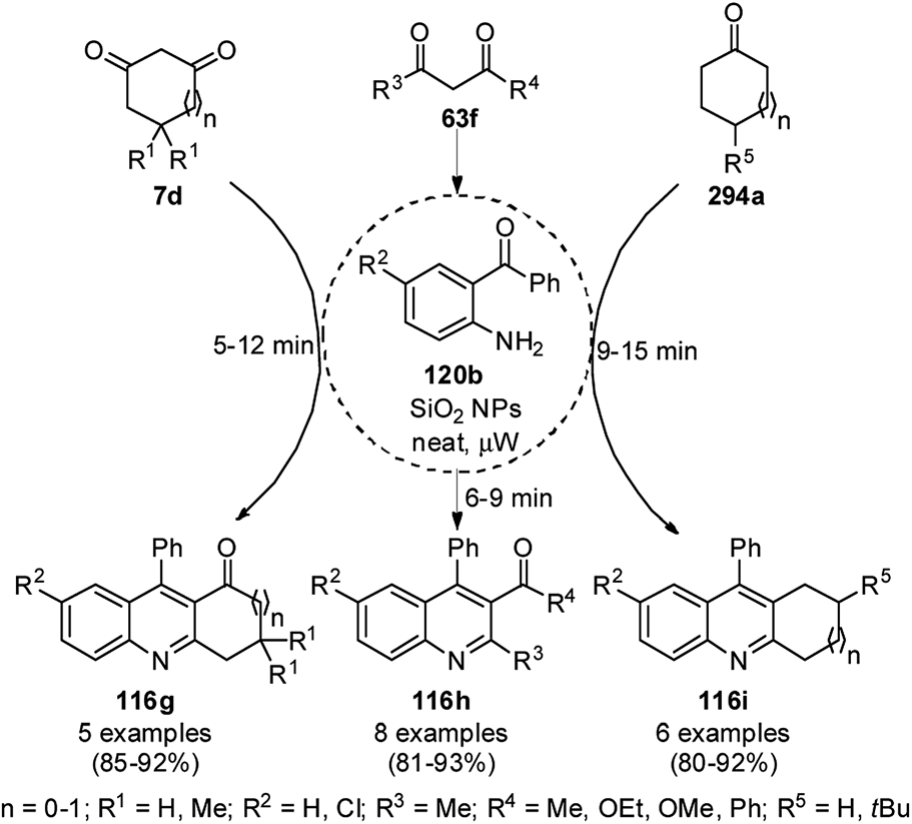
SiO_2_ NP-catalyzed synthesis of quinolines (116g/h/i).

Zare *et al.* reported the silica NP-catalyzed synthesis of quinoxalines (38f) from *o*-phenylene diamines (33f) and substituted benzil (256d) under solvent-free and mild reaction conditions (Scheme 182).^330^ The applicability of the SiO_2_ NPs was also demonstrated for the Friedländer synthesis of quinolines (116g/h/i) from *o*-aminobenzophenone (120b) and ketones (7d/63f/294a) under neat and microwave irradiation (Scheme 183). The scope of the Friedländer synthesis was explored by Zare *et al.* by utilizing various ketones such as dimedone, cyclohexan-1,3-dione, cyclopentan-1,3-dione, 2,4-pentandione, ethyl acetoacetate, methyl acetoacetate, 1-phenylbutane-1,3-dione, cyclohexanone, 4-*tert*-butyl cyclohexanone, cyclopentanone, and dimedone. They synthesized the SiO_2_ NPs following the reported protocol and NPs were characterized *via* SEM. The authors demonstrated the recyclability of the NPs for up to 15 catalytic cycles with good yields for the synthesis of quinoxalines. The silica NPs through its silanol group bondage with the reagents *via* hydrogen bonds bring the reactants closer to each other for the formation of the products.

**Scheme 184:**
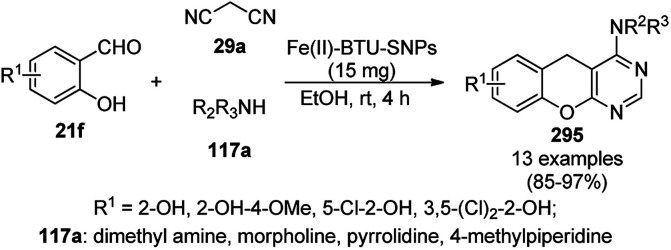
Silica NP-catalyzed synthesis of benzopyranopyrimidines (295) under mild reaction conditions.

**Scheme 185:**
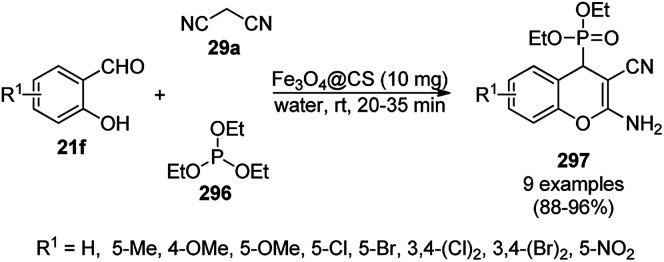
Synthesis of 2-amino-4*H*-chromen-4-yl phosphonates (297) by Fe_3_O_4_ NPs supported on sulfochitosan (Fe_3_O_4_@CS).

Kassaee *et al.* reported the one-pot synthesis of benzopyranopyrimidines using silica nanoparticles (SiNPs) grafted on a benzoylthiourea ferrous complex from substituted *o*-hydroxy benzaldehyde (21f), 29a and secondary alkyl amine (117a) in EtOH at rt (Scheme 184).^331^ SiNPs were coated with aminopropyltriethoxysilane and benzoyl isothiocyanate to immobilize benzoylthiourea on them following the treatment of the formed complex with FeCl_2_ to form Fe(ii)-BTU-SNPs. The synthesized NPs were characterized *via* FT-IR, TGA, EDX, SEM, and TEM. The catalyst promoted the Knoevenagel condensation between the substrate as a Lewis acid catalyst by increasing the electrophilic character of the carbonyl of *o*-salicylaldehydes. The reusability of the nanocatalyst was claimed by Kassaee *et al.* for up to five catalytic cycles without loss in its catalytic potential. Further, they have also reported Fe_3_O_4_ NPs supported on sulfochitosan (Fe_3_O_4_@CS) for the synthesis of 2-amino-4*H*-chromen-4-yl phosphonates (297, Scheme 185) from *o*-salicylaldehydes (21f), malononitrile (29a) and triethyl phosphite (296).^332^

**Scheme 186:**
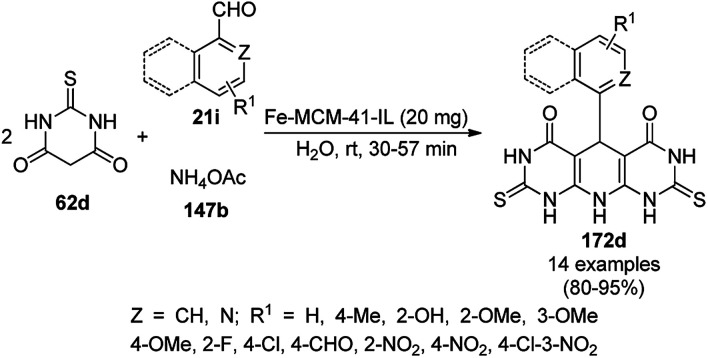
Green synthesis of 1,4-dihydropyrimidines (172d) in aqueous conditions.

IL-functionalized Fe-containing mesoporous SiNPs (Fe-MCM-41-IL) catalyzed cyclocondensation for the synthesis of pyrido[2,3-*d*:6,5-*d*]dipyrimidines was achieved successfully under aqueous conditions at rt from 2-thiobarbituric acids (62d), aryl or heteroaryl aldehydes (21i) and ammonium acetate (147b, Scheme 186).^333^ The required catalyst was synthesized by loading triazolium IL on iron containing mesoporous silica (MCM-41) following the reported procedure.^334^ The magnetically retrievable catalyst was reused for six consecutive runs in 93–95% yield.

**Scheme 187:**
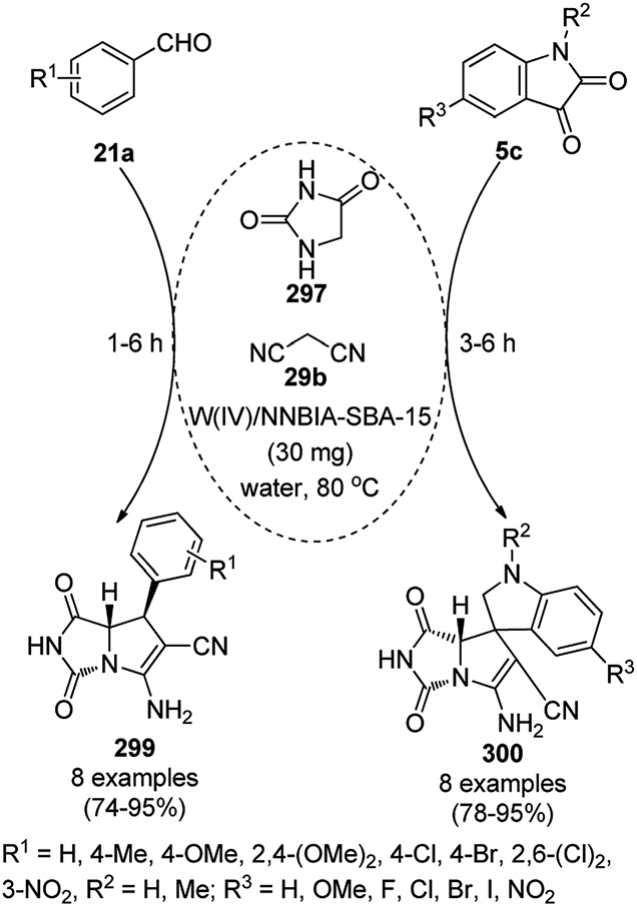
Synthesis of 2-azapyrrolizidine under aqueous conditions.

Tungsten immobilized on SBA-15 [W(iv)/NNBIA-SBA-15] catalyzed Knoevenagel–Michael-5-*exo*-dig cyclization from the MCR between hydantoin (298), 29b and benzaldehydes (21a) or isatins (5c) led to the synthesis of 7-phenyl-2,3,7,7*a*-tetrahydro-1*H*-pyrrolo[1,2-*c*]imidazole (299) or spirooxindole-2-azapyrrolizidine (300) (Scheme 187).^335^ Chlorofunctionalized SBA-15, which was synthesized by the treatment of SBA-15 and (3-chloropropyl)triethoxysilane, was later treated with *N*,*N*′-(ethane-1,2-diyl)bis(2-aminobenzamide) (NNBIA) and WCl_6_ for the covalent grafting of tungsten to obtain the final NPs. The filtered catalysts at the end of the reaction were recycled for up to five runs without loss in its catalytic activity.

**Scheme 188:**
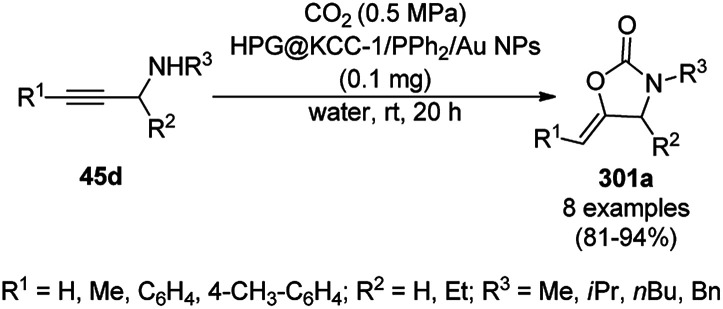
Synthesis of 2-oxazolidinones (300) catalyzed by SiNPs.

An Au(iii) phosphorus complex grafted on fibrous SiNPs (HPG@KCC-1/PPh_2_/Au NPs) catalyzed the carboxylation of substituted propargylic amines (45d) to achieve the successful synthesis of 2-oxazolidinones (301a) at rt under aqueous conditions (Scheme 188).^336^ KCC-1 NPs, which were synthesized from TEOS, were treated with glycidol and chlorodiphenylphosphine to obtain phosphite-functionalized organosilica (HPG@KCC-1/PPh_2_), which was subsequently treated with sodium tetrachloroaurate to obtain the final NCs. Following the ease of the recovery of the catalyst by filtration, it was recycled ten times with consistent catalytic activity.

**Scheme 189:**
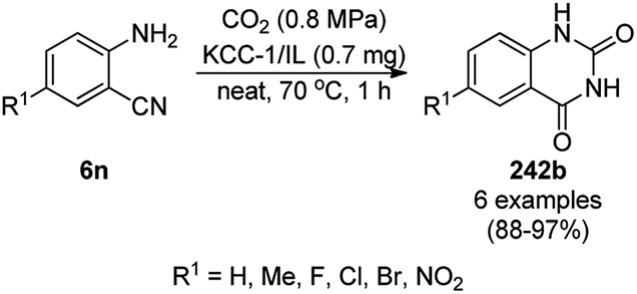
Synthesis of quinazoline-2,4-diones (242b) catalyzed by KCC-1.

IL immobilized on KCC-1-catalyzed carboxylation of *o*-amino benzonitrile (6n) for the synthesis of quinazoline-2,4-diones (242b) (Scheme 189) was reported by Sadeghzadeh *et al.*^337^ The prepared KCC-1 was silylated with 3-chloropropyltriethoxysilane followed by the loading of hexamethylenetetramine using sodium borohydride and potassium hydroxide. A comparison of the textual parameters of KCC-1 and KCC-1/IL revealed that the IL-loaded SiNPs possess a finer pore size, volume and surface area, which increased their catalytic capacity. The optimization of various reaction parameters led to the use of 0.7 mg KCC-1/IL, solvent-free conditions, with heating at 70 °C for 1 h as the best conditions to synthesize 242b. The reuse of the catalyst was attempted for up to ten cycles with more than 90% yield, indicating the catalytic stability of IL-loaded KCC-1, where the reused catalyst after the tenth run was found to possess a similar texture to the fresh catalyst (TEM and FT-IR). Further, Sadeghzadeh *et al.* claimed that their most recent protocol was superior^338–342^ since it functioned at a relatively low temperature, without the use of solvent, with the lowest pressure of CO_2_, lowest catalytic loading and shortest duration of treatment.

**Scheme 190:**
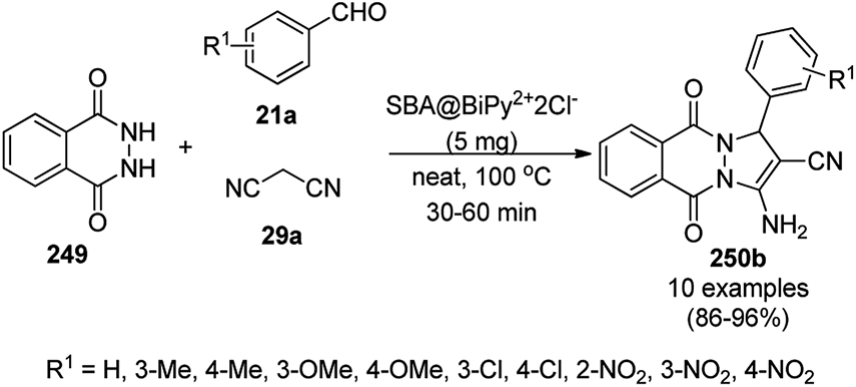
Environmentally benign synthesis of 1*H*-pyrazolo[1,2-*b*]phthalazine-5,10-diones (250b).

4,4′-Bipyridinium dichloride ordered mesoporous SiNPs (SBA@BiPy^2+^2Cl^−^) catalyzed Michael addition-cyclocondensation for the synthesis of 3-amino-1-phenyl-5,10-dioxo-5,10-dihydro-1*H*-pyrazolo-[1,2-*b*]phthalazine-2-carbonitriles (250b) in 86–96% yield from phthalhydrazide (249), malononitrile (29a), and aryl carbaldehydes (21a) (Scheme 190).^343^ The homocoupled product of 4,4′-bipyridine with 3-chloropropyltriethoxysilane was loaded on Pluronic P123, an amphiphilic surfactant, to obtain dicationic NCs (SBA@BiPy^2+^Cl^2−^). The retention of the catalytic activity was proven by investigating the recycling of the SiNPs for up to seven reuses, giving 91–96% yield in the model reaction of 249, 29a and benzaldehyde.

**Scheme 191:**
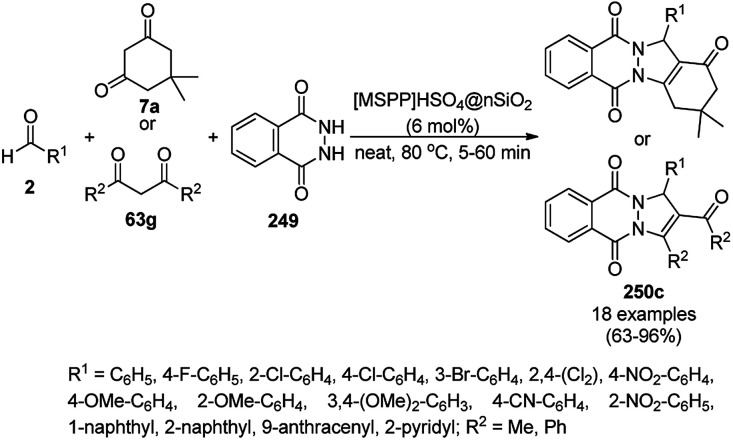
Synthesis of substituted pyrazolo[1,2-*b*]phthalazine-5,10-diones (250c) from aromatic aldehyde (2), 1,3-diketone (7a or 63g) and 2,3-dihydrophthalazine-1,4-dione (249).^344^

**Scheme 192:**
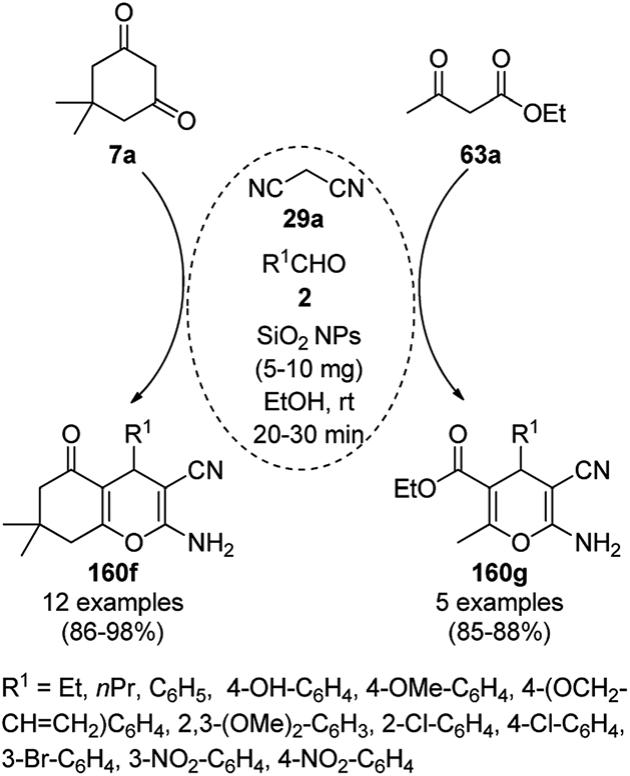
Synthesis of tetrahydrobenzo[*b*]pyrans (160f/g) catalyzed by SiO_2_ NPs.

Substituted pyrazolo[1,2-*b*]phthalazine-5,10-diones (250c) were synthesized from substituted aromatic aldehyde (2), dimedone or acyclic 1,3-diketone (63g) and 2,3-dihydrophthalazine-1,4-dione (249) in the presence of heterogeneous acidic ionic liquid 4-methyl-1-(3-sulfopropyl)pyridinium hydrogen sulfate [MSPP]HSO_4_@nSiO_2_ in 6 mol% at 80 °C under solvent-free conditions (Scheme 191).^344^ The newly developed catalyst was well characterized *via* elemental analysis, Fourier transform infrared spectroscopy (FT-IR) and scanning electron microscopy (SEM), and was recycled up to five times without decay in its catalytic activity.

**Scheme 193:**
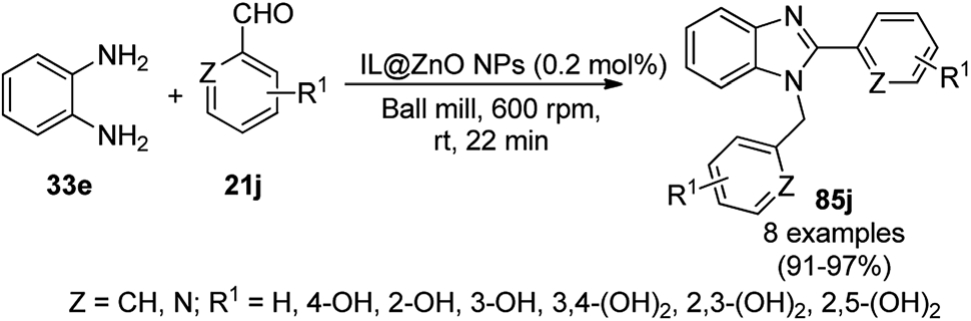
IL@ZnO NP-catalyzed synthesis of 1,2-disubstituted benzimidazoles (85j) reported by Singh *et al.*

The SiO_2_ NP-catalyzed multicomponent reaction of 3,3-dimethyl-1,3-cyclohexanedione (7a) or ethyl acetoacetate (63a) with malononitrile (29a) and carbaldehydes (2) in ethanol at rt led to the synthesis of 2-amino-3-cyano tetrahydrobenzo[*b*]pyrans (160f) or 2-methyl-4-aryl-4*H*-pyran-3-carboxylate (160g, Scheme 192) in good yields.^152^ It was also explored for the pseudo-four-component synthesis of hexasubstituted anilines using 29a (2 equiv.), 2, and substituted acetophenones. This environmentally benign catalyst was reused and recycled for up to eight runs.

### 3.10 ZnNP-catalyzed synthesis of heterocycles

Singh *et al.* reported that ionic liquid-coated zinc oxide nanoparticles (IL@ZnO NPs) catalyzed the green synthesis of 1,2-disubstituted benzimidazoles (85j) from *o*-phenylene diamine (33e) and substituted benzaldehydes (21j) under ball-milling conditions at rt in a short time under an argon environment (Scheme 193).^345^ Here, ZnO was found to form a 3D-network with 1-methyl-3-carboxymethylimidazole bromide because of the high affinity of ZnO with COOH. In general, the reaction of *o*-phenylene diamine with benzaldehyde derivatives can lead to the formation of 2-substituted benzimidazole and 1,2-disubstituted benzimidazole. However, the reported protocol was found to be selective for the synthesis of 1,2-disubstituted benzimidazole in excellent yields over 2-substituted benzimidazole. ZnO-NPs play a key catalytic role in the 1,3-hydrogen shift, with stronger affinity towards the imine bond. The ZnO NPs were recycled up to six times without loss in their catalytic reactivity, where after the seventh catalytic run, the catalyst lost its morphological character, as confirmed from SEM and DLS studies. The scalability of the synthesized catalyst was demonstrated by the authors for up to 80 mmol of *o*-phenylene diamine and salicylaldehyde with 90% yield of the target 1,2-disubstituted benzimidazole. The developed protocol with the IL@ZnO NPs exhibits the merits of high eco-scale value, low E-factor, high yield, shorter reaction time and simpler purification by washing with aqueous methanol in comparison with the literature reports.^346–353^

**Scheme 194:**
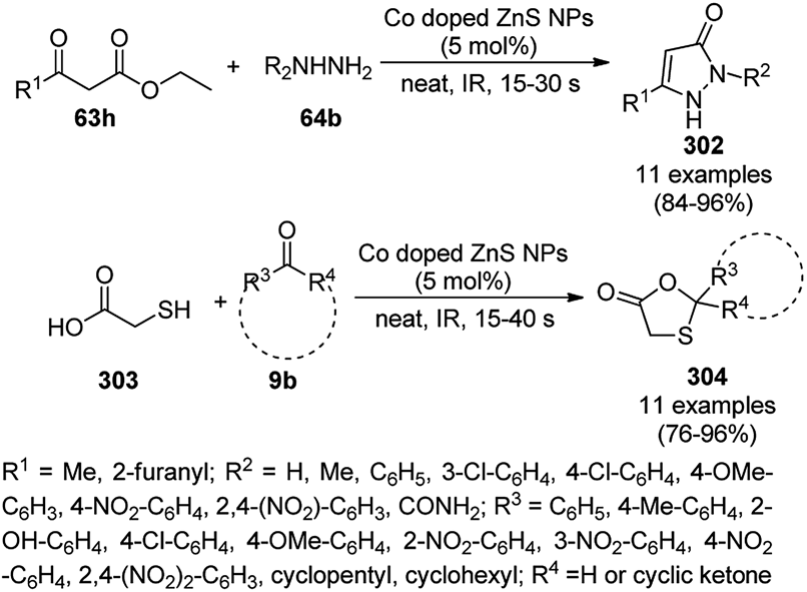
Co-doped ZnS NP-catalyzed synthesis of pyrazolones (302) and 1,3-oxathiolan-5-ones (304) under IR irradiation.

Dandia *et al.* reported the synthesis of pyrazolones (302) from substituted ethyl acetoacetate (63h) and substituted hydrazine (64b) under solvent-free conditions using IR irradiation catalyzed by cobalt-doped zinc sulphide nanoparticles (Co-doped ZnS NPs, Scheme 194).^354^ The same protocol was explored for the synthesis of 1,3-oxathiolan-5-ones (304) from 2-thioacetic acid (303) and substituted aldehyde or ketones (9b) in 76–96% yield. The co-doped ZnS NPs were prepared using a green aqueous chemical method following the literature reported procedures^355,356^ and characterized *via* XRD, TEM, EDAX, ICP-AES and UV-Vis spectroscopy. The investigation of the recyclability of the catalyst revealed that the catalyst recovered after sonication could be recycled for up to four times without loss in its catalytic activity and its morphological character remained the same, as confirmed by SEM and TEM.

**Scheme 195:**
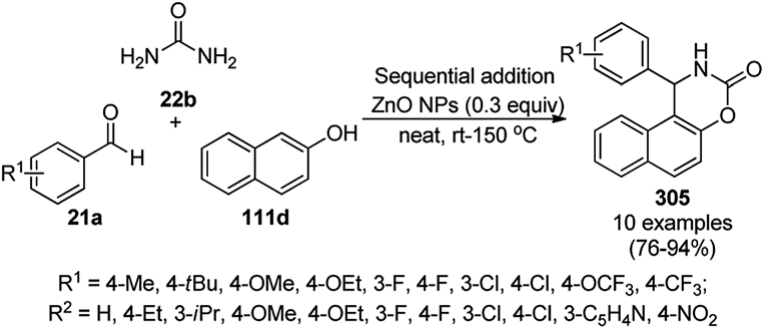
ZnO NP-catalyzed synthesis of 1,2-dihydro-1-arylnaphtho[1,2-*e*][1,3]oxazine-3-ones (305).

**Scheme 196:**
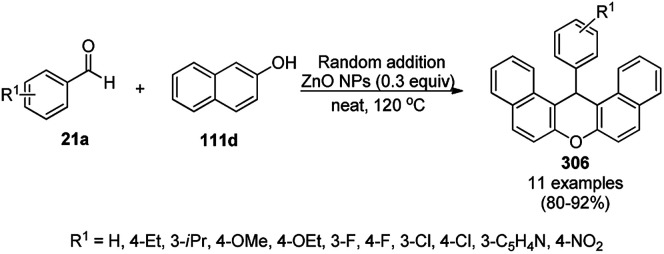
ZnO NP-catalyzed synthesis of 14-substituted-14*H*-dibenzo[*a*,*j*]xanthenes (306).

Kaushik *et al.* reported that commercially available zinc oxide nanoparticles (ZnO NPs) as a Lewis acid catalyzed the synthesis of 1,2-dihydro-1-arylnaphtho[1,2-*e*][1,3]oxazine-3-ones (305) and 14-substituted-14*H*-dibenzo[*a*,*j*]xanthenes (306, Scheme 195) under solvent-free conditions.^357^ The synthesis of 305 was achieved from substituted benzaldehyde (21a) and urea (22b) following the sequential addition of β-naphthol (111d) in 76–94% yield, whereas 306 was synthesized from substituted benzaldehyde (21a) and β-naphthol (111d) at 120 °C *via* random addition in 80–92% yield (Scheme 196). The recyclability of the catalyst was also investigated and the yield of the final product was found to be up to 68% in the fourth catalytic reuse of the catalyst after increasing the duration of the reaction.

**Scheme 197:**
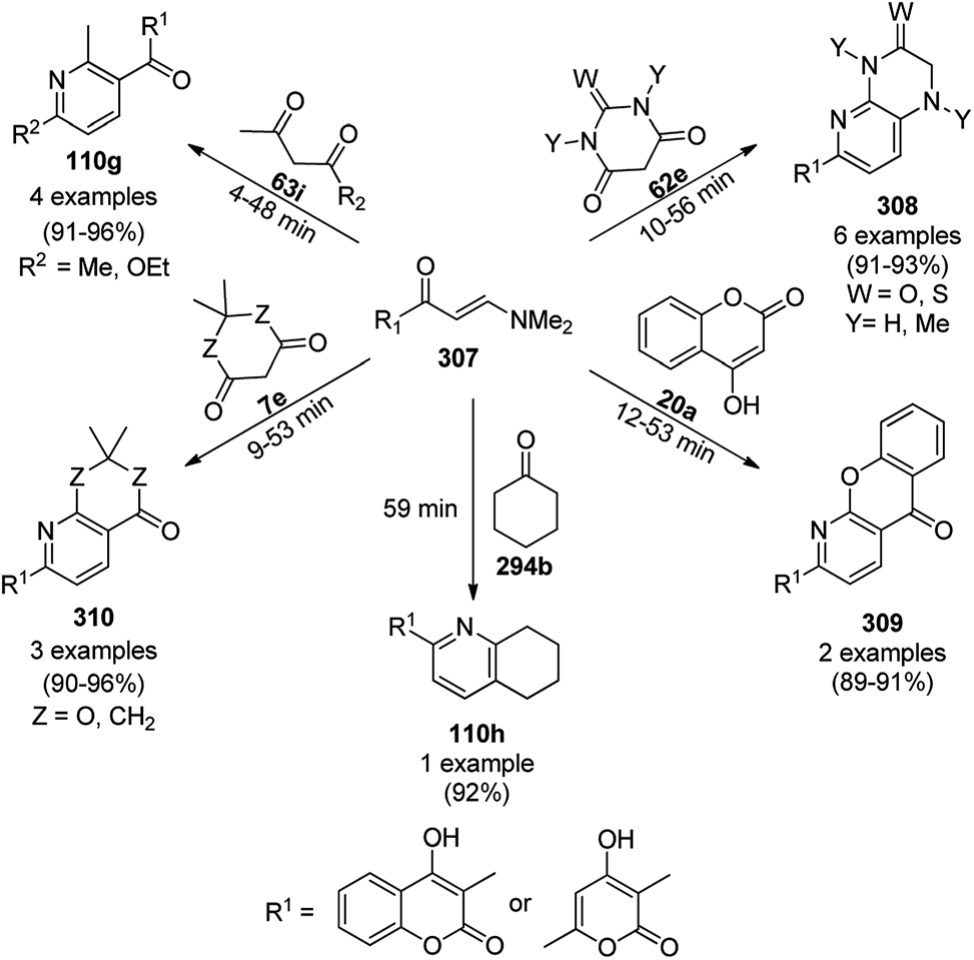
Synthesis of the pyridines catalyzed by ZnO NPs (10 mol%) at 70 °C.

Siddiqui *et al.* reported the synthesis of new pyridines *via* the multi-component reactions of β-enaminones (206), active methylene compounds and ammonium acetate using a catalytic amount of ZnO NPs in 10 mol% (Scheme 197) at 70 °C.^358^ The ZnO NPs were synthesized *via* the sol–gel method and characterized using XRD, SEM, and TEM. This catalyst was reused for the synthesis of the target compound from β-enaminones, ethyl acetoacetate and ammonium acetate for up to six catalytic cycles without loss in its catalytic activity, as was evident by the XRD spectrum of the ZnO NPs.

**Scheme 198:**
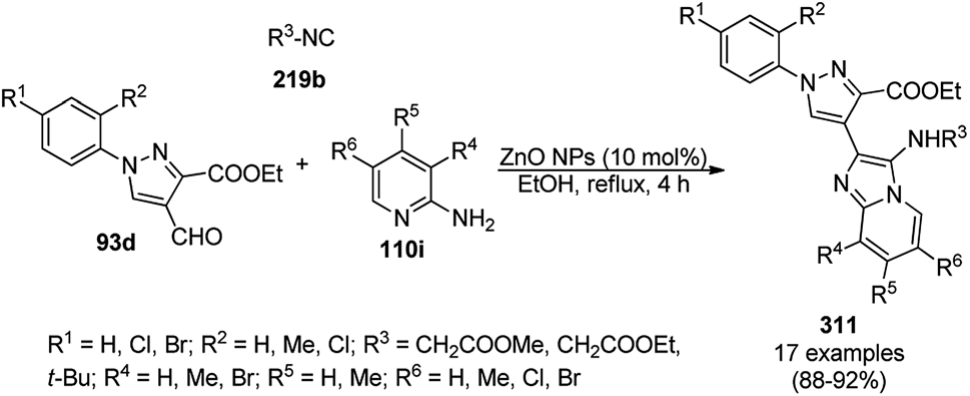
ZnO NP-catalyzed synthesis of imidazo[1,2-*a*]pyridines (311).

The heterogeneous ZnO NP-catalyzed one-pot synthesis of pyrazole-coupled imidazo[1,2-*a*]pyridine (311) *via* the three-component reaction (Scheme 198) of ethyl pyrazole-3-carboxylate (93d), 2-aminopyridine (110i) and isocyanide (219b) was developed by Shrivastava *et al.* in a benign solvent.^359^ The ZnO NPs were prepared following the literature method^360^ using zinc acetate and ammonium carbonate and characterized *via* TEM, SEM, and XRD. The catalyst was separated *via* simple filtration and found to lose its catalytic activity after eight catalytic runs due to agglomeration, which after calcination at 450 °C for 4 h regained its activity, as confirmed by its reuses in five catalytic cycles. The ZnO NPs catalyzed this condensation *via* HB formation by the hydroxyl groups present on their surface with the carbonyl oxygen or iminic nitrogen of the formed intermediates.

**Scheme 199:**
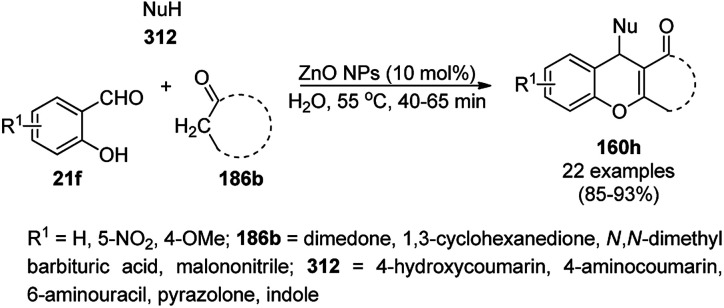
ZnO NP-catalyzed synthesis of densely functionalized 4*H*-chromenes (160h).

ZnO NPs prepared though the bottom-up method^355^ under green conditions were found to be successful for the synthesis of 4*H*-chromenes (160h, Scheme 199) *via* the three-component reaction of salicylaldehydes (21f), active methylene compounds (186b) and nitrogen- and oxygen-bearing nucleophiles (312).^361^ The reused NPs were recycled for up to six times with >85% of the Knoevenagel–Michael-cyclization adduct.

**Scheme 200:**
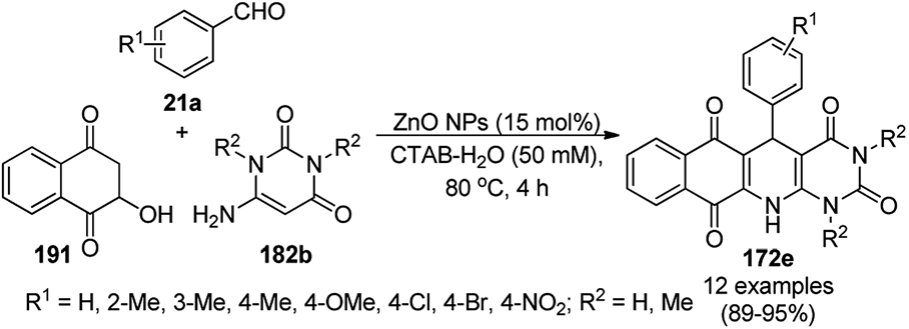
Synthesis of benzo[*g*]pyrimido[4,5-*b*]-quinoline-2,4,6,11(1*H*,3*H*)-tetraone (172e).

Siddiqui *et al.*^362^ reported the synthesis of nano ZnO^361^ using zinc acetate dehydrate and potassium hydroxide and characterized its nano nature (15–25 nm) *via* EDS, TEM and XRD. Further, they also used the nano ZnO for the synthesis of pyrimido[4,5-*b*]quinolines (172e, Scheme 200) using benzaldehydes (21a), 2-hydroxynaphthalene-1,4-dione (191) and 6-aminouracil (182b) in a green admicellar aqueous solution of CTAB as an emulsifying agent. After the completion of the reaction, ethyl acetate was added to the reaction mixture and the catalyst was recycled for up to five catalytic runs.

**Scheme 201:**
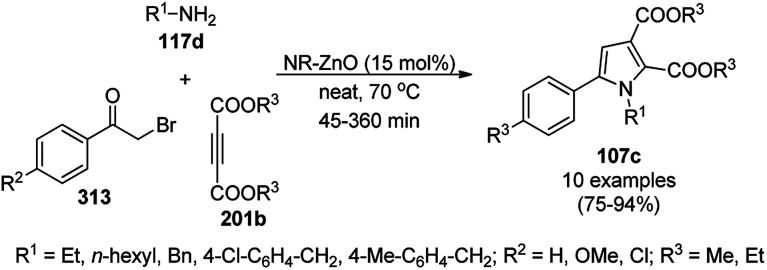
Catalytic applications of nanorod-ZnO in the synthesis of polysubstituted pyrroles (107c).

The synthesis of tetra-substituted pyrroles (107c, Scheme 201) *via* the three-component reaction of aliphatic amines (117d), dimethyl or diethyl acetylenedicarboxylates (201b) and phenylacetyl bromide (313) was achieved using a catalytic amount of ZnO nanorods. ZnO NPs were prepared *via* the neutralization of zinc acetate dihydrate with caustic soda at 80 °C and treated with SDS in aqueous NaOH to obtain ZnO rods. However, the reaction failed with less nucleophilic anilines, and the more electrophilic ethyl bromopyruvate.^363^

**Scheme 202:**
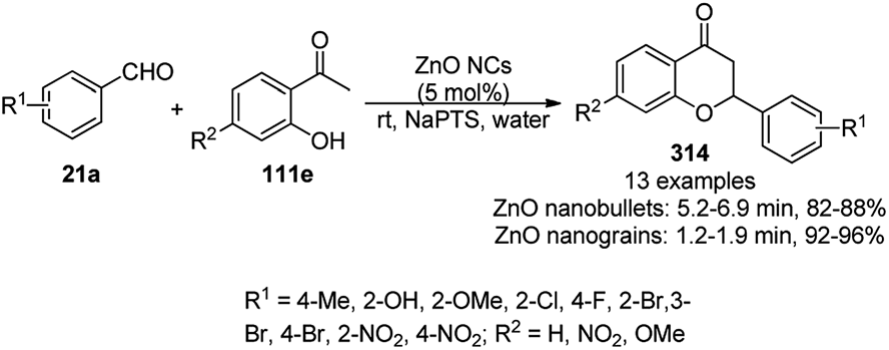
Synthesis of flavanones (314) under green benign conditions.

The first aldol condensation at rt between *o*-hydroxy acetophenone (111e) and benzaldehydes (21a) driven under aqueous hydrotropic and basic medium formed by ZnO nanobullets or nanograins and sodium *p*-toluenesulfonate (NaPTS) was designed for the brisk synthesis of flavanones (314, Scheme 202).^364^ The ZnO NPs were obtained from zinc chloride and NaOH *via* co-precipitation and 90–96% yield of flavanones was obtained during the five times recycling of the ZnO NPs at pH 12. ZnO nanograins with a finer particle size of 90 nm were found to give higher yields of flavanones in a shorter time than ZnO nanobullets (particle size of 600 nm × 110 nm). Compared to the reported methodology for the synthesis of flavanones such as Fe(HSO_4_)_3_/SiO_2_ (ref. 365) and hydromagnesite,^366^ the ZnO NP-catalyzed protocol is operational at rt in a shorter time.

**Scheme 203:**
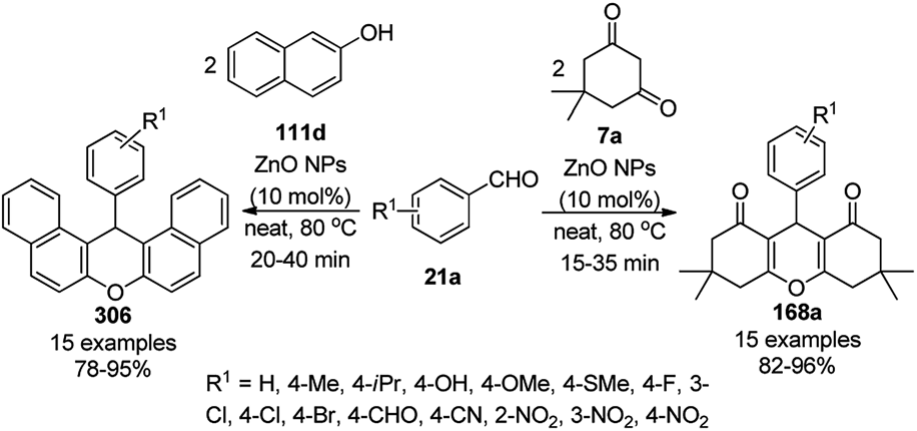
Synthesis of 14-phenyl-14*H*-dibenzo[*a*,*j*]xanthenes (306) and 1,8-dioxooctahydroxanthenes (168a).

ZnO NPs as a Lewis acid catalyzed the condensation of aldehydes (21a) with β-naphthols (111d) and dimedone (7), which led to the efficient synthesis of 14-phenyl-14*H*-dibenzo[*a*,*j*]xanthenes (306) and 1,8-dioxooctahydroxanthenes (168a), respectively (Scheme 203). Grinding of zinc acetate and oxalic acid in an agate mortar for 1 h at rt led to the formation of ZnC_2_O_4_·2H_2_O NPs, which were calcined at 450 °C to obtain the final NPs (20–30 nm, as confirmed by XRD, SEM and EDAX).

**Scheme 204:**
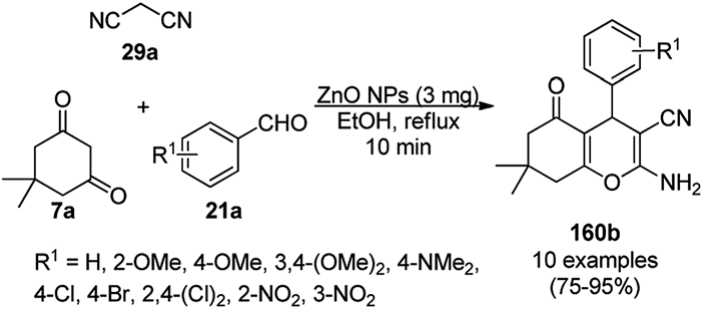
ZnO NP-catalyzed synthesis of 2-amino-4*H*-chromenes (160b).

Zavar *et al.* reported the catalytic use of ZnO NPs or the synthesis of 2-amino-4*H*-chromenes (160b) *via* the multicomponent reaction of dimedone (7a), malononitrile (29a) and aromatic benzaldehyde (21a, Scheme 204).^154^ The ZnO NPs were prepared from zinc acetate and urea in the presence of SDS and characterized *via* TEM, SEM and XRD.

**Scheme 205:**
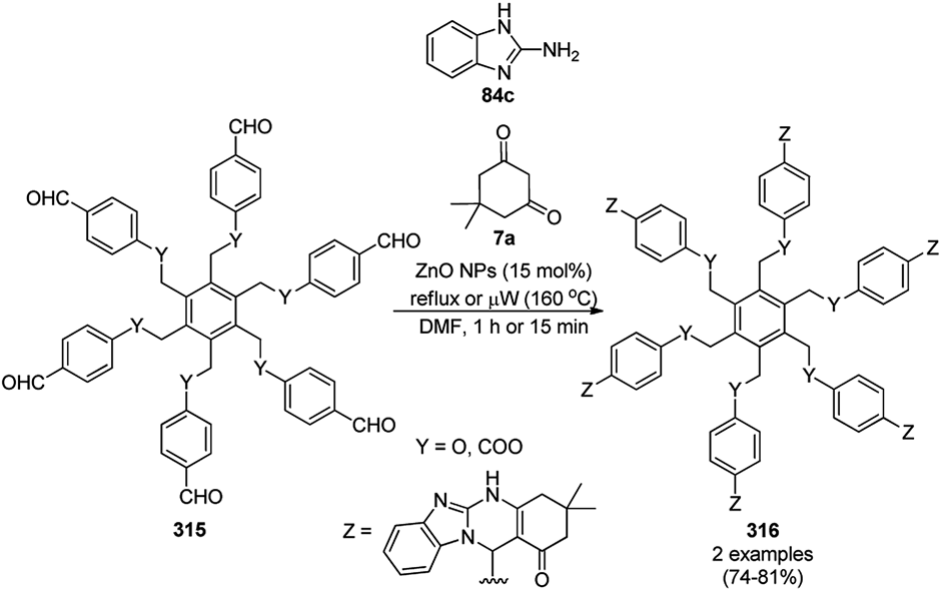
ZnO NP-mediated synthesis of multi-armed poly(tetrahydrobenzimidazo[2,1-*b*]quinazolin-1(2*H*)-ones) (316).

Commercially available ZnO NP-catalyzed Knoevenagel–Michael-cyclization for the synthesis of multi-armed poly(tetrahydrobenzimidazo[2,1-*b*]quinazolin-1(2*H*)-ones) attached with phenyl *via* benzoyloxy or phenoxymethyl linkers (316) was successfully achieved using 2-amino benzimidazoles (84c), dimedone (7a) and hexakis-aldehydes (315, Scheme 205).^367^ The same protocol was also observed to be successful with previously synthesized tris-aldehydes and tetrakis-aldehydes for the synthesis of novel polypodal compounds.

**Scheme 206:**
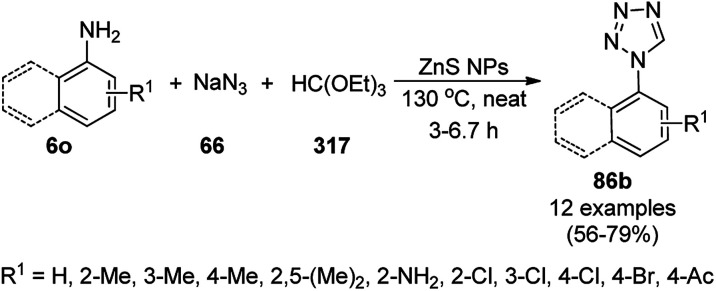
ZnS NPs as heterogeneous catalyst in [3 + 2] cycloaddition.

Zinc sulphide nanoparticle (ZnS NP)-catalyzed [3 + 2] cycloaddition for the synthesis of 1-substituted tetrazoles (86b) was reported using primary aromatic amine (6o), 66 and triethyl orthoformate (317) at 130 °C in 56–79% yield under solvent-free conditions (Scheme 206).^368^ The reaction proceeded without any side reactions and evolution of hydrazoic acid. The catalyst preserved its crystalline behavior even after seven cycles of reuse, as evident from the XRD pattern of the recycled catalyst.

### 3.11 Other MNP-catalyzed synthesis of heterocycles

**Scheme 207:**
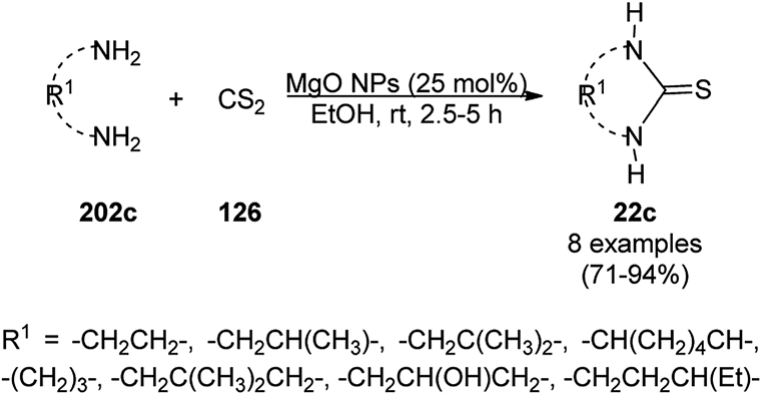
Synthesis of cyclic thioureas (22c) catalyzed by MgO NPs.

The MgO NP-catalyzed synthesis of cyclic thioureas (22c) such as imidazoline-2-thiones and tetrahydropyrimidone-2-thiones was reported by Beyzaei *et al.* using 1,2- or 1,3-diaminoalkanes (202c) and carbon disulfide (126) in ethanol in 71–84% yield (Scheme 207).^369^ Further, the synthesized compounds (22c) also showed good anti-microbial activity against Gram-positive and Gram-negative pathogenic bacteria. The present protocol for the synthesis of cyclic thiourea has the advantages of operational at rt and proceeds without the formation of pernicious hydrogen sulfide gas in comparison with that in the literature.^370–375^

**Scheme 208:**
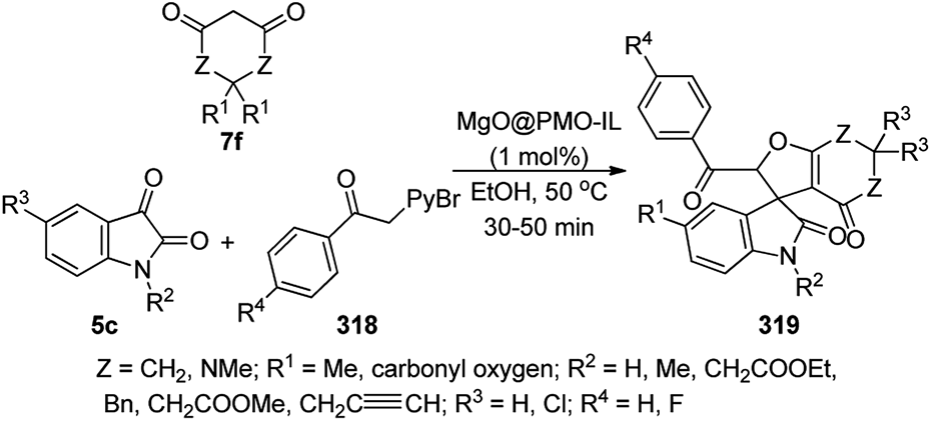
Synthesis of spirooxindole-furan derivatives (319).

MgO NPs immobilized on IL-based periodic mesoporous organosilica (Mg@PMOL-IL) catalyzed the cyclocondensation of isatins (5c), 1,3-dicarbonyl compounds such as dimedone or 1,3-dimethylpyrimidine-2,4,6(1*H*,3*H*,5*H*)-trione (7f) and *N*-phenylacetyl pyridinium bromides (318), providing a reasonable approach for the synthesis of spirooxindole-furan derivatives (Scheme 208) in excellent yields.^376^ The key catalyst was synthesized *via* the deposition of MgO NPs on PMOL-IL, pre-synthesized from tetramethoxysilane and 1,3-bis(3-trimethoxysilylpropyl) imidazolium chloride using Pluronic P123 as a structure-directing agent. The novel catalyst was well characterized *via* FT-IR, TGA, BET, SEM, and TEM. After the completion of the reaction, the catalyst was separated by filtration and reused seven times, retaining its catalytic potential for the synthesis of spirocyclic compounds (319).

**Scheme 209:**
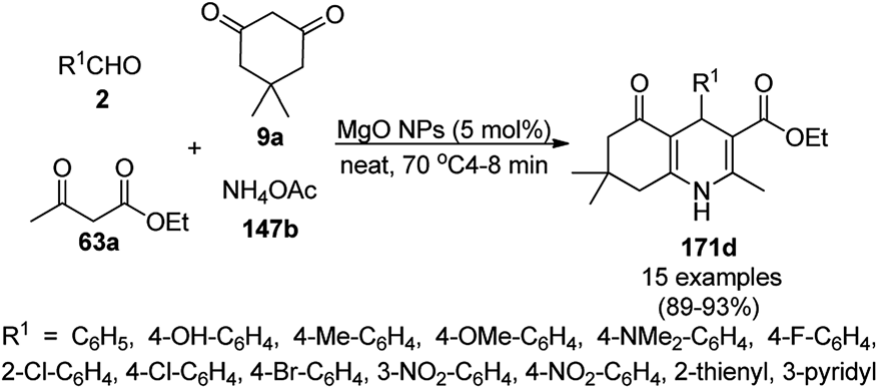
Synthesis of polyhydroquinolines (171d) under neat conditions catalyzed by MgO NPs.

Reza *et al.* reported the MgO NP-catalyzed synthesis of polyhydroquinoline (171d, Scheme 209) from aromatic aldehydes (2), 9a, 63a and 147b under solvent-free conditions in 89–93% yield.^377^ They synthesized MgO NPs from MgCl_2_ and NaOH in PEG sonochemically. The catalyst was recovered by centrifugation and recycled using a model reaction involving benzaldehyde, 9a, 63a and 147b for up to seven cycles with 85–92% yield of 171d.

**Scheme 210:**
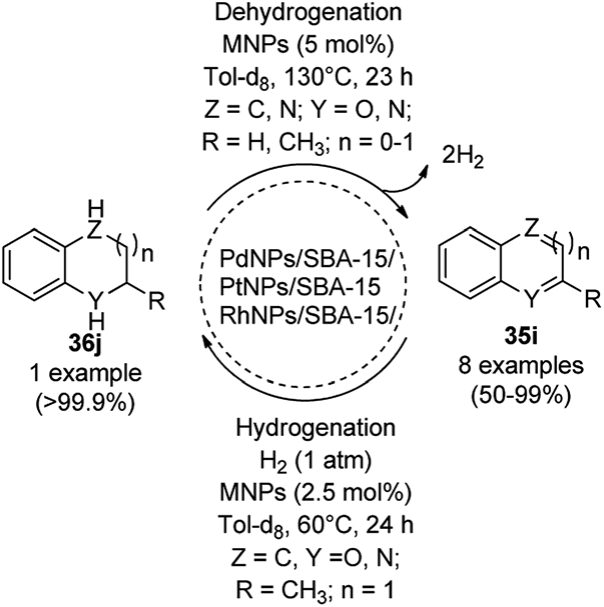
MNP-catalyzed dehydrogenation and hydrogenation.

Somorjai *et al.* reported the successful dehydrogenation of tetrahydroquinoline (36j) using MNPs/SBA-15 (5 mol% using metals such as Pd, Pt and Rh) as heterogeneous metallic nanoparticles (MNPs) in deuterated toluene-*d*_8_ as the solvent at 130 °C for 23 h (Scheme 210).^157^ The same reaction was found to be inferior with Rh, Pt and Pd salts such as rhodium chloride (RhCl_3_), rhodium(iii) acetylacetonate (Rh(acac)_3_), potassium tetrachloroplatinate(II), Pt/C and palladium chloride (PdCl_2_). The hydrogenation of 2-methyl quinoline using hydrogen gas (1 atm) and MNPs/SBA-15 (2.5 mol%), in toluene-*d*_8_ solvent at 60 °C for 24 h gave >99.9% yield with Pt and Pd MNPs. No catalytic decay was observed for the PdNPs/SBA-15 for up to three catalytic runs.

Various coumarin derivatives have been reported as important scaffolds having diversified biological activities such as anticancer,^378^ anti-HIV,^379^ anti-inflammatory,^380^ anti-microbial^381^ activities. The synthesis of 5-oxo-4*H*,5*H*-pyrano[3,2-*c*]chromene-3-carbonitrile derivatives (160a) was performed using 4-hydroxycoumarin (20a), 29a and substituted arylaldehydes (21a), employing molybdenum oxide nanoparticles (MoO_3_-NPs, 5 mol%) at 80 °C using EtOH : H_2_O as a co-solvent (4 : 1, Scheme 211). These NPs efficiently catalyzed the reaction for up to six cycles without loss in their catalytic activity.^382^

**Scheme 211:**
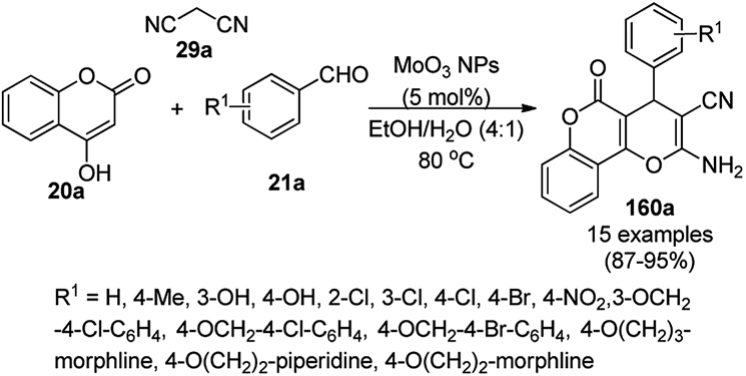
One-pot synthesis of 4-phenyl-substituted pyrano-fused coumarins (160a) catalyzed by MoO_3_ NPs under green conditions.

**Scheme 212:**
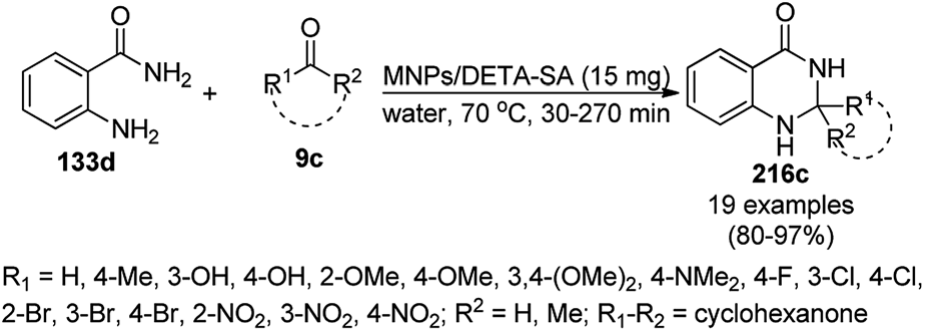
Synthesis of 2,3-dihydroquinazolin-4(1*H*)-one (216c).

**Scheme 213:**
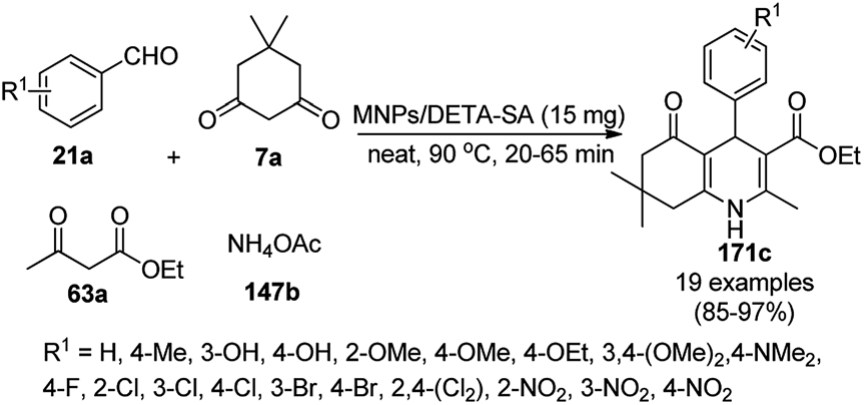
Synthesis of polyhydroquinolines (171c) using the MNP/DETA-SA catalyst.

Shiri *et al.* synthesized sulfamic acid immobilized on amino-functionalized magnetic nanoparticles (MNPs/DETA-SA) and used them as a catalyst for the synthesis of 2,3-dihydroquinazoline-4(1*H*)-ones (216c) and polyhydroquinoline derivatives (171c, Scheme 212).^383^ The MNPs/DETA-SA catalyst was synthesized by loading sulfamic acid on amino-functionalized MNPs. The MNPs were prepared *via* a co-precipitation procedure followed by treatment with 3-chloropropyltrimethoxysilane (CPTMS) and diethylenetriamine (DETA). 216c was synthesized *via* the cyclocondensation of 133d with aldehydes/ketones (9c) in the presence of MNPs/DETA-SA (15 mg for 1 mmol of reaction) in water at 70 °C in 80–97% yield. The same catalyst was used to catalyze the reaction of 21a, 7a, 63a and 147b at 90 °C to give 171c in 85–97% yield (Scheme 213). The efficiency of the present protocol was compared with the some of the previously reported methods catalyzed by palladium chloride PdCl_2_,^384^ K_7_[PW_11_CoO_40_],^385^ Cu-SPATB/Fe_3_O_4_,^386^ [TBA]_2_[W_6_O_19_], KAl(SO_4_)_2_.E_12_H_2_O,^387^ and silica-bonded *N*-propylsulfamic acid (SBNPSA).^388^ It was claimed that the MNPs/DETA-SA-catalyzed synthesis has the advantages of higher yields, shorter reaction times, and milder or green conditions.

**Scheme 214:**
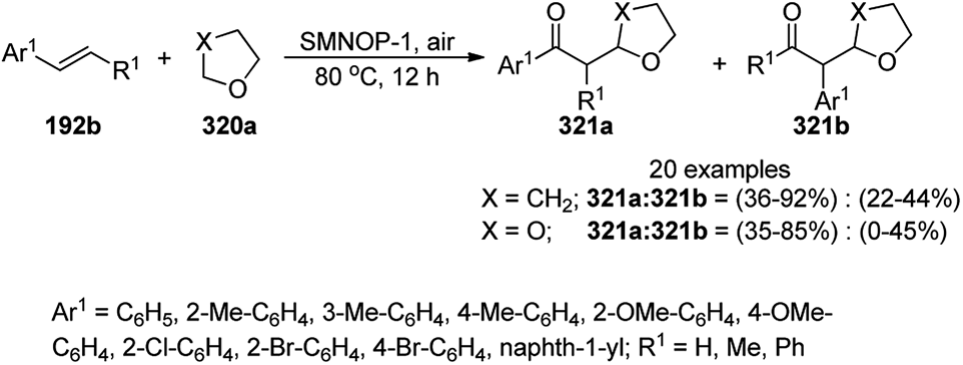
Scope of SMNOP-1 catalyzed oxyalkylation of vinylarenes (192b).

Wang *et al.* reported the regioselective oxyalkylation of vinylarenes (192b) with tetrahydrofuran or dioxolane (320a) catalyzed by diatomite-supported manganese oxide (Mn_3_O_4_) nanoparticles (SMNOP-1) in the presence of air at 80 °C in 12 h using tetrahydrofuran (THF) as the solvent (Scheme 214).^389^ The SMNOP-1 NPs were synthesized using Mn(OAc)_2_·4H_2_O, diatomite, cetyltrimethylammonium bromide (CTAB) in DMSO, and further been characterized *via* TEM and XRD. The recyclability of SMNOP-1 was successfully demonstrated by the authors for the model reaction involving the oxyalkylation of styrene and THF for up to five catalytic cycles without loss in its catalytic activity. The structure of SMNOP-1 remained intact as confirmed by the XRD and TEM images of the catalyst taken before and after four consecutive catalytic runs. SMNOP-1 helps in the catalytic oxidation of tetrahydrofuran to tetrahydrofuran free radical and oxidation of the alcohol intermediate to the final ketone product.

**Scheme 215:**
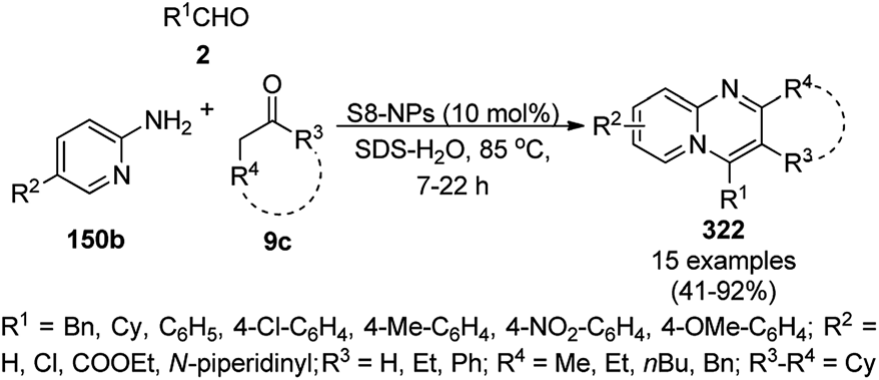
Sulfur NP-catalyzed synthesis of substituted 4*H*-pyrido[1,2-*a*]pyrimidines (322) in SDS-water medium.

Siddiqui *et al.* reported the sulfur nanoparticle (S8 NP)-catalyzed synthesis of substituted 4*H*-pyrido[1,2-*a*]pyrimidines (322) using an aqueous micellar medium of sodium dodecyl succinate (SDS), substituted 2-aminopyridine (150b), aldehydes (2) and methylene ketones (9c) at 85 °C (Scheme 215).^390^ The S8 NPs were synthesized using elemental sulfur and characterized *via* XRD, TEM, and EDX. The sulfur NPs provided a nanocatalytic surface in the micellar environment for the reagents to interact with each other. The recyclability of the NPs was demonstrated for up to five catalytic runs. However, the catalytic potential of the NPs declined to 66% yield of the final product during fifth catalytic run because of their aggregation, as confirmed by TEM.

**Scheme 216:**
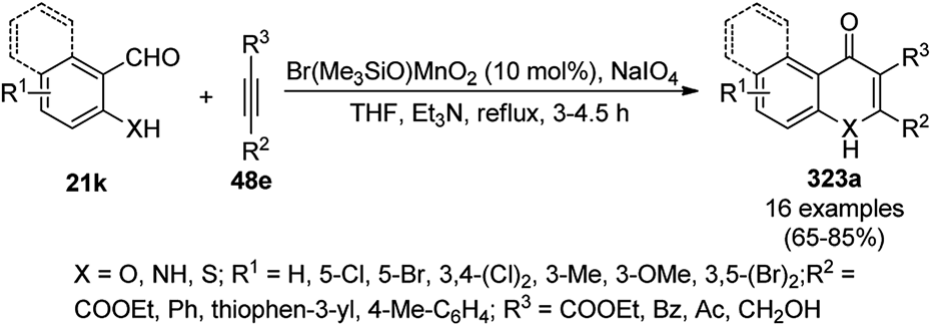
MnNP-catalyzed synthesis of flavones (323a).

Maiti *et al.* reported the Mn(vi) nanoparticle (MnNP)-catalyzed synthesis of flavones (323a) from substituted aldehydes (21k) and substituted acetylenes (48e) in THF under reflux using sodium as a stoichiometric oxidant and triethylamine (Et_3_N) as the base in moderate to good yields (Scheme 216).^391^ The required MnNPs were synthesized using KMnO_4_ and characterized *via* HR-TEM, TEM, STEM-EELS, XPS, and EPR spectroscopy. The MnNPs actively participate in the catalytic cycle in the oxidative C–C coupled annulation process.

**Scheme 217:**
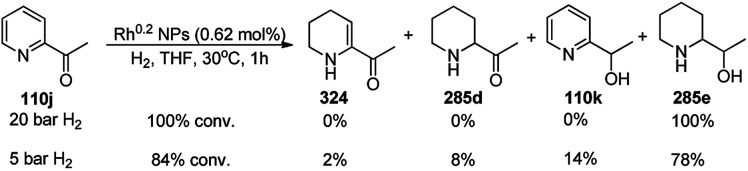
Selective reduction of 1-(pyridin-2-yl)ethan-1-one (110j) using RhNPs.

Godard *et al.* reported the synthesis of RhNPs stabilized by N-heterocyclic carbenes (NHCs) *via* the decomposition of [Rh(η^3^-C_3_H_5_)_3_] under an H_2_ atmosphere, and using Rh NPs, they performed the selective reduction of 1-(pyridin-2-yl)ethanone (110j) at 30 °C in the presence of hydrogen gas in THF (Scheme 217).^392^ At 20 bar pressure of H_2_ gas, 100% conversion to 1-(piperidin-2-yl)ethanol was observed. The authors also reported the selective reduction of phenol to cyclohexanol/cyclohexanone and quinoline to 1,2,3,4-tetrahydroquinoline or decahydroquinoline *via* the fine tuning of the reaction conditions.

**Scheme 218:**
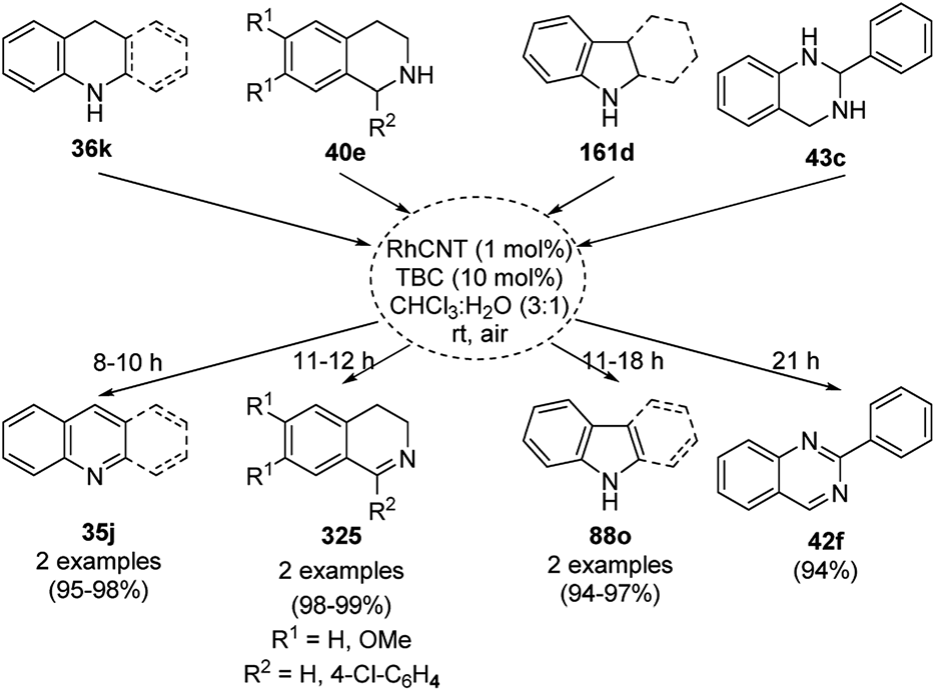
RhNPs as a co-catalyst in the dehydrogenation of N-heteroaromatics.

Another interesting application of RhNPs immobilized on carbon nanotubes (CNTs) as co-catalysts was demonstrated for the cooperative dehydrogenation of azaheterocycles such as 1,2,3,4-tetrahydroquinoline or 9,10-dihydroacridine (36k), 1,2,3,4-tetrahydroisoquinoline (40e), indoline or 2,3,4,4a,9,9a-hexahydro-1*H*-carbazole (161d) and 2-phenyl-1,2,3,4-tetrahydroquinazoline (43c) catalyzed by 4-*tert*-butylcatechol (TBC, Scheme 218).^393^ The catalyst was reused five times and no notable reduction in catalytic activity was observed (93–95%). The RhNPs enabled the conversion of TBC into its oxidized hydroquinone form, which could help in the dehydrogenation of azaheterocycles. The present protocol could also oxidize dibenzyl amine to its imine form.

**Scheme 219:**
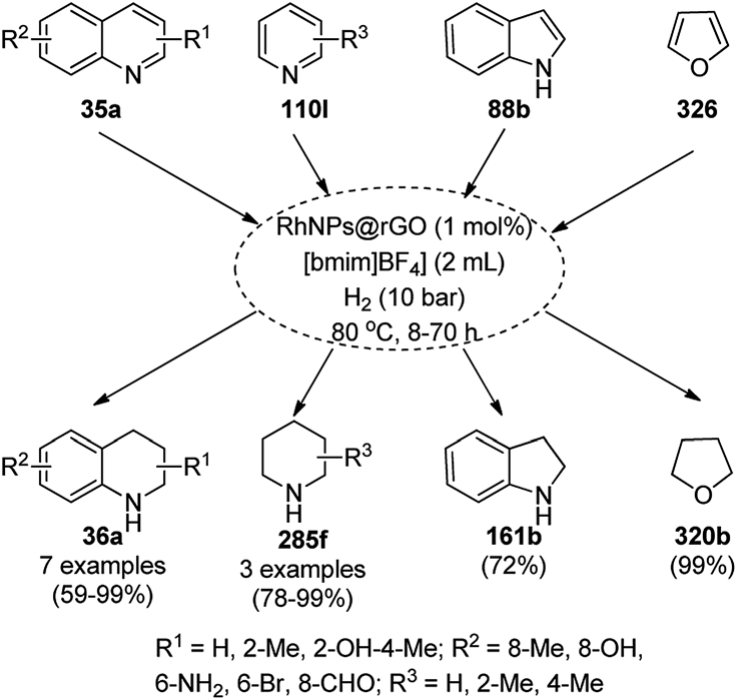
Hydrogenation of nitrogen- and oxygen-containing heterocyclic scaffolds.

The hydrogenation of aza-heterocycles such as quinolines (35a), pyridines (110l), indole (88b) and oxa-heterocycle such as furan (326) was reported using a catalytic amount of RhNPs supported on rGO (Scheme 219) by Dyson *et al.* using an ionic liquid as the reaction medium.^394^ The hydrogenation of natural constituents having benzofurans such as visnagin, 8-methoxy psoralen, and khellin was achieved successfully using this catalyst with selective reduction of the furan rings. The catalyst was recycled up to five times with 98–91% yield of 1,2,3,4-tetrahyroquinolines.

**Scheme 220:**
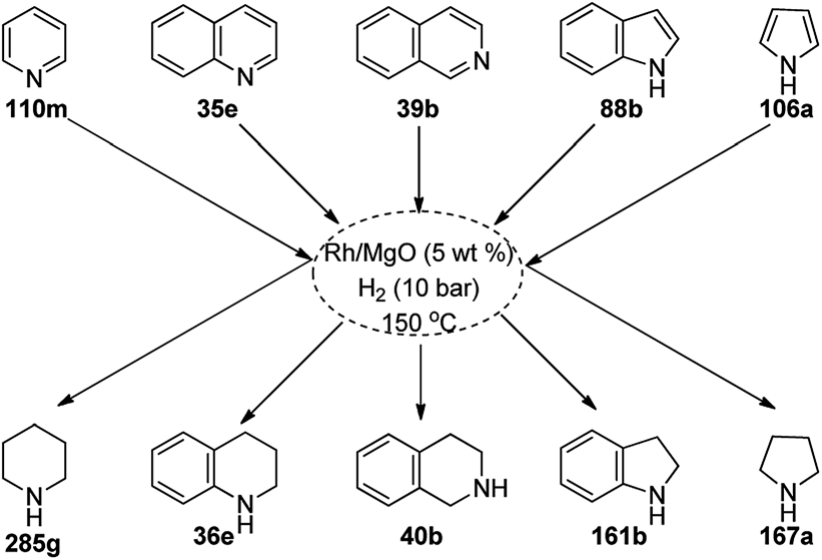
Hydrogenation of aza-heterocycles catalyzed by RhNPs supported on MgO. Complete product distribution (100%) was reported in each instance.

Delgado *et al.* reported the hydrogenation of N-heterocycles such as pyridine (110m), quinoline (35e), isoquinoline (39b), indole (88b) and pyrrole (106a) catalyzed by RhNPs supported on alkaline magnesium oxide (MgO) at 150 °C with a high turnover frequency (TOF) of 58 900–18 500 h^−1^ (Scheme 220).^395^ The same protocol was also reported for the successful hydrogenation of olefins and arenes. The catalyst was recycled up to four times with a high TOF in the range of 11 500–10 500 h^−1^ for the catalytic hydrogenation of toluene, where it retain maintained its catalytic activity without any notable structural change, as confirmed by TEM.

**Scheme 221:**
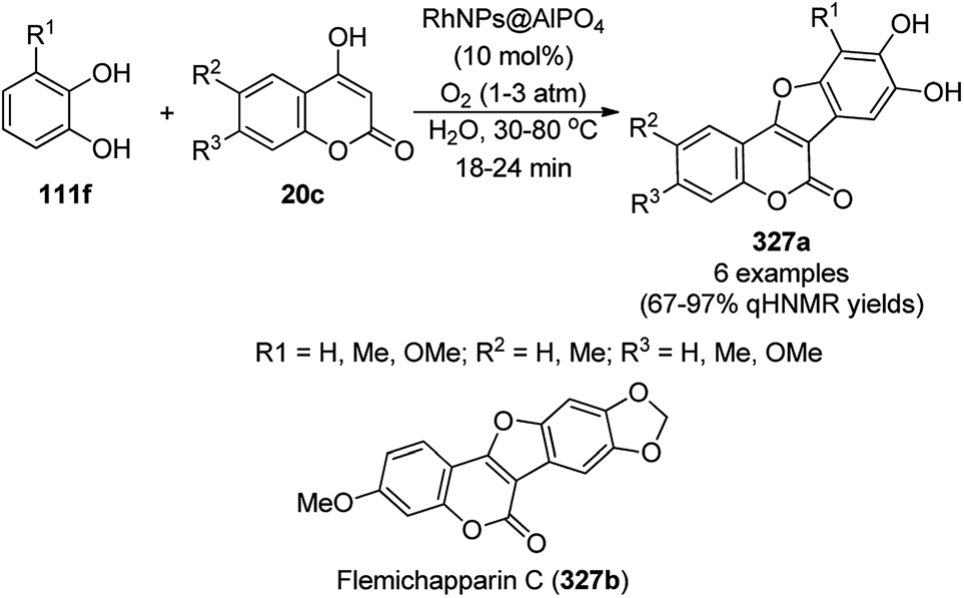
RhNP-catalyzed synthesis of benzofurans and flemichapparin C.

Recently, Jitsukawa *et al.* reported the catalytic use of RhNPs supported on aluminium phosphate (RhNP@AlPO_4_) for the synthesis of benzofurans (327a) *via* the oxidative cross-coupling of catechols (111f) and hydroxy coumarins (20c) (Scheme 221).^396^ RhNPs were obtained *via* the treatment of Rh(acac)_3_ with AlPO_4_ followed by calcination to obtain grey powdered NPs. Further, these NPs were employed in the total synthesis of the natural product flemichapparin C (327b) in 46% yield. The *o*-benzoquinones generated *in situ via* the oxidative dehydrogenation of 111f by the RhNPs underwent nucleophilic addition by 20c, followed by cyclization to yield 327a.

**Scheme 222:**
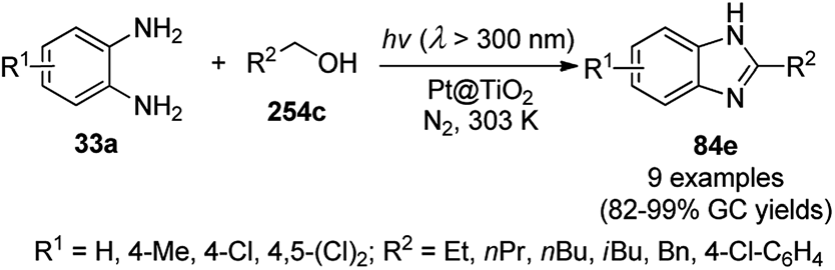
Pt@TiO_2_-catalyzed one-pot synthesis of benzo[*d*]imidazoles (84e) from *o*-phenylene diamine (33a) and substituted alcohols (254c).

The one-pot synthesis of benzimidazoles (84e) from *o*-phenylene diamine (33a) and substituted alcohols (254c) was reported by Shiraishi *et al. via* platinum-assisted photocatalytic oxidation on the surface of TiO_2_ (Scheme 222). This methodology has certain key features, where it is free from acids and oxidants, generates innocuous by-products, namely water and H_2_, and is operational at rt. The heterogeneous catalyst was comprised of TiO_2_ semiconductor loaded with Pt. The yields of the final products were found to be significantly better in the presence of Pt, which was claimed for the oxidative conversion of benzimidazoline, the key intermediate, into benzimidazole. Photo-activated TiO_2_ enables the oxidation of alcohol to aldehyde. This protocol was selective for the synthesis of 2-substituted benzimidazole rather than *N*,1-disubstituted benzimidazole (1-(1-ethoxyethyl)-2-methyl-1*H*-benzimidazole).^397^

**Scheme 223:**
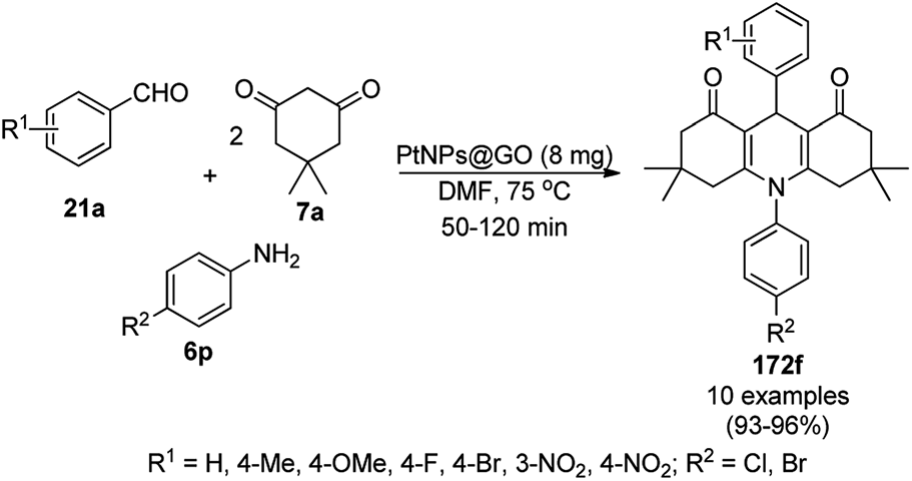
One-pot synthesis of 1,4-dihydropyridines (172f) catalyzed by PtNPs@GO.

PtNPs supported on graphene oxide were employed in the one-pot synthetic preparation of acridinediones (172f) using aldehydes (21a), dimedone (7a), and 4-halosubstituted anilines (6p) in excellent yields *via* three successive aldol and Michael and cyclizations.^398^ Among the tested conditions, 8 mg PtNPs, and DMF as the solvent at 75 °C were found to be successful for the synthesis of 172f (Scheme 223). The PtNPs were prepared *via* the reduction of PtCl_4_ using super hydride and ethanol using octyl amine as the ligand under ultrasonication until the formation of a brown-black solution, which was later mixed with graphene oxide to obtain PtNPs@GO. The reuse of the catalyst was demonstrated for up to six cycles with 88–93% yield.

**Scheme 224:**
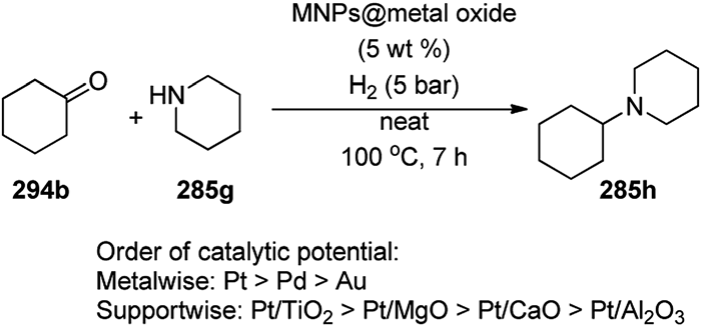
Reductive amination of cyclohexanone (294b) with piperidine (285g) catalyzed by PtNPs supported on charcoal or metal oxides.

Domine *et al.* studied the catalytic use of Pt, Pd and Au NPs supported on several oxides such as Al_2_CO_3_, TiO_2_, CeO_2_, ZnO, MgO, CaO, and ZrO_2_ or charcoal for the hydrogenation-mediated reductive amination of a few ketones such as cyclohexanone (294b), 2-hexanone and 2-octanone with piperidine (285g) *via* high-throughput experimentation (Scheme 224).^399^ Among the various attempts, they observed that PtNPs was the best catalyst for the reductive amination with high turnover numbers (TONs), better catalytic activity and selectivity. They also found that the efficiency of the reductive amination depends on the properties of the used metal, solid support and their types. Further, they also reported that the treatment of the support before metal loading and after calcination also affected the catalytic efficiency enormously for the reductive amination of 294b with 285g.^400^

**Scheme 225:**
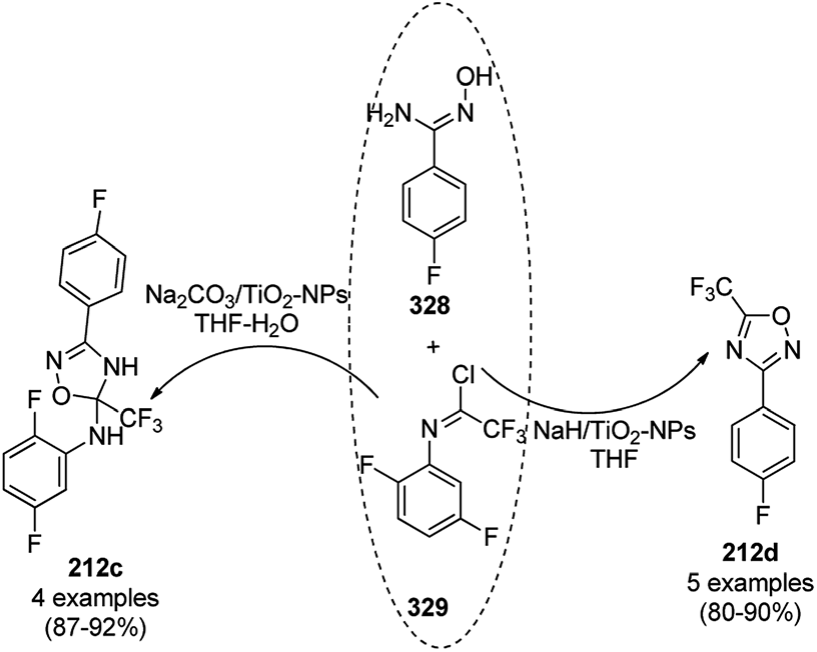
TiO_2_-nanoparticle-catalyzed synthesis of trifluoromethyl-4,5-dihydro-1,2,4-oxadiazoles (212c) and trifluoromethyl-1,2,4-oxadiazoles (212d).^401^

The selective synthesis of trifluoromethyl-4,5-dihydro-1,2,4-oxadiazoles (212c, Scheme 225)^401^ and trifluoromethyl-1,2,4-oxadiazoles (212d) was carried out using amidoximes (328) and trifluoroacetimidoyl chlorides (329). Also, 212c was synthesized using Na_2_CO_3_ as the base, THF–H_2_O as the co-solvent system at rt, and titanium dioxide nanoparticles as the catalyst, whereas 212d was synthesized using amidoximes and trifluoroacetimidoyl chlorides with NaH as the base, THF as the solvent, and titanium dioxide nanoparticles as the catalyst at rt.

**Scheme 226:**
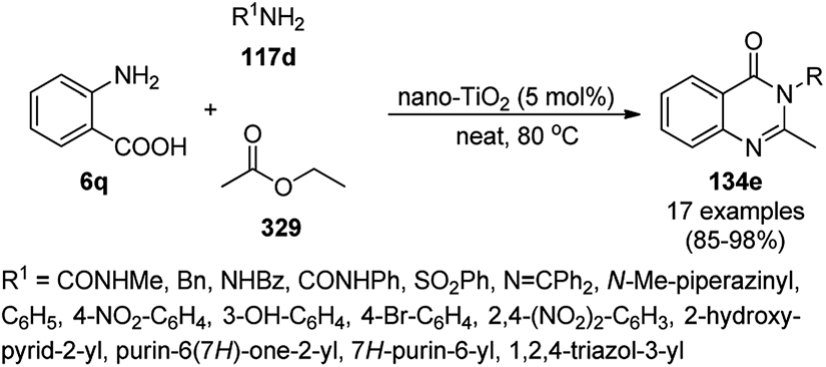
Nano-TiO_2_ NP-catalyzed three-component reaction of *o*-amino benzoic acid (6q), ethyl acetate (329) and amine (117d).

Panahi *et al.* reported the one-pot titanium dioxide nanoparticle (TiO_2_ NP)-catalyzed synthesis of quinazolines (134e) from *o*-amino benzoic acid (6q), ethyl acetate (329) and substituted amines (117d) under solvent-free conditions at 80 °C (Scheme 226).^402^ The authors observed that this reaction could not proceed without the nanoform, of TiO_2_ and further the synthetic targets were observed under solvent-free conditions. Other titanium salts such as titanium chloride (TiCl_4_) and titanium isopropoxide [Ti(Oi-Pr)_4_] failed to give the products. The recovery of the catalyst was studied for up to four catalytic runs. Further, the synthesized compounds were evaluated for *in vitro* vasorelaxant activity using thoracic rat aorta, and the IC_50_ of a few compounds was found as good as that of acetylcholine.

**Scheme 227:**
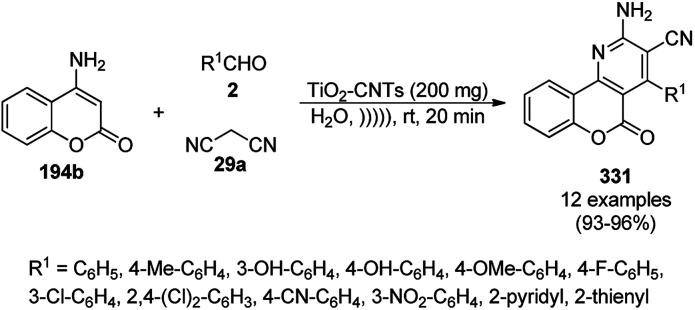
Synthesis of chromeno[*b*]pyridines (331) catalyzed by TiO_2_ NPs.

TiO_2_ NPs immobilized on CNTs (TiO_2_-CNTs) catalyzed Knoevenagel–Michael-cyclization for the green synthesis of chromeno[*b*]pyridines (331) using 4-amino coumarin (194b), aryl aldehyde (2) and malononitrile (29a) mediated by ultrasonic irradiation (Scheme 227).^403^ The TiO_2_-NCTs were synthesized *via* the sonochemical treatment of multi-walled CNTs with tetraethyl orthotitanate using SDS as a stabilizer. The NC was separated by centrifugation and recycled for up to four runs with excellent activity in 94%, 93%, 93% and 92% yield for the model reaction between 194b, benzaldehyde and 29a.

**Scheme 228:**
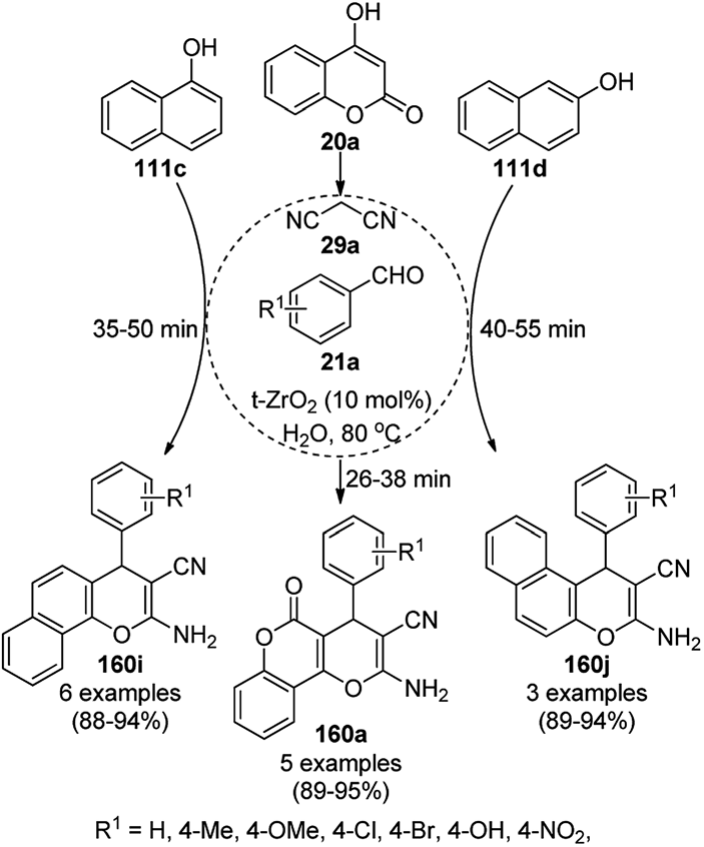
Synthesis of pyran-chromenes catalyzed by fluorescent t-ZrO_2_ NPs.

The tetragonal ZrO_2_ (t-ZrO_2_) NP-catalyzed Knoevenagel–Michael-cyclization for the aqueous synthesis of pyran-chromenes (160i/a/j) was successfully achieved using malononitrile (29a), substituted benzaldehydes (21a) and hydroxy compounds such as α-naphthol (111c) or β-naphthol (111d) or 4-hydroxycoumarin (20a, Scheme 228).^153^ The t-ZrO_2_ NPs acted as a Lewis acid catalyst and coordinated with the carbonyl of 21a, cyano of the intermediates and hydroxy of 111c/20a/111d to form the products (160i/a/j), respectively. The catalyst was recycled up to ten times with a slight loss (12%) in its catalytic performance compared to that in the initial run with the fresh catalyst. The t-ZrO_2_-catalyzed protocol was claimed to be comparable with reported catalysts such as triazine-functionalized mesoporous organosilica (TFMO-1),^404^ basic alumina,^405^ and disodium calcium diphosphate (Na_2_CaP_2_O_7_)^406^ for the synthesis of 160i.

**Scheme 229:**
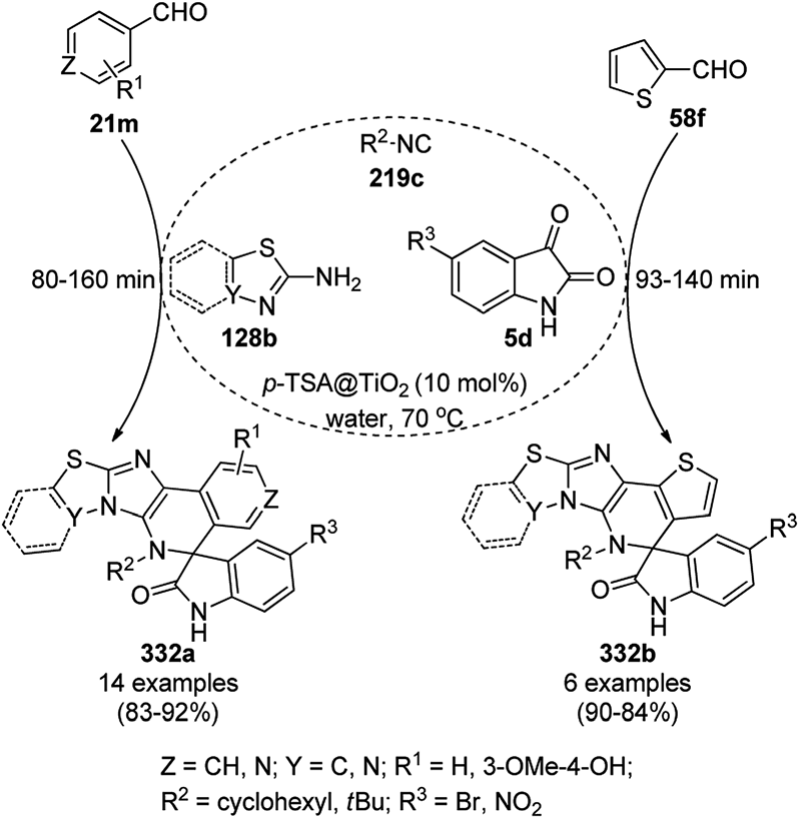
Synthesis of spirooxindoles (332a/332b) catalyzed by *p*-TSA@TiO_2_ NPs.

*p*-Toluenesulfonic acid (*p*-TSA)-modified TiO_2_ (*p*-TSA@TiO_2_)-catalyzed the Groebke–Blackburn–Bienaymé (GBB) and its post-modified Pictet–Spengler reaction for the aqueous synthesis of spirooxindoles (332a/b) using aromatic carbaldehydes (21m/58f), isocyanides (219c), 2-amino benzo[*d*]thiazole (128b) and 5-substituted isatins (5d) were achieved by Kumar *et al.* (Scheme 229) for the first time.^407^ The catalyst was synthesized following a thermo-reversible sol–gel method involving the use of titanium tetraisopropoxide (Ti(O^i^Pr)_4_) and *p*-TSA.^408^ The catalyst was recovered by filtration and reused for up to eight times, which yielded 82–90% of spirooxindoles.

**Scheme 230:**
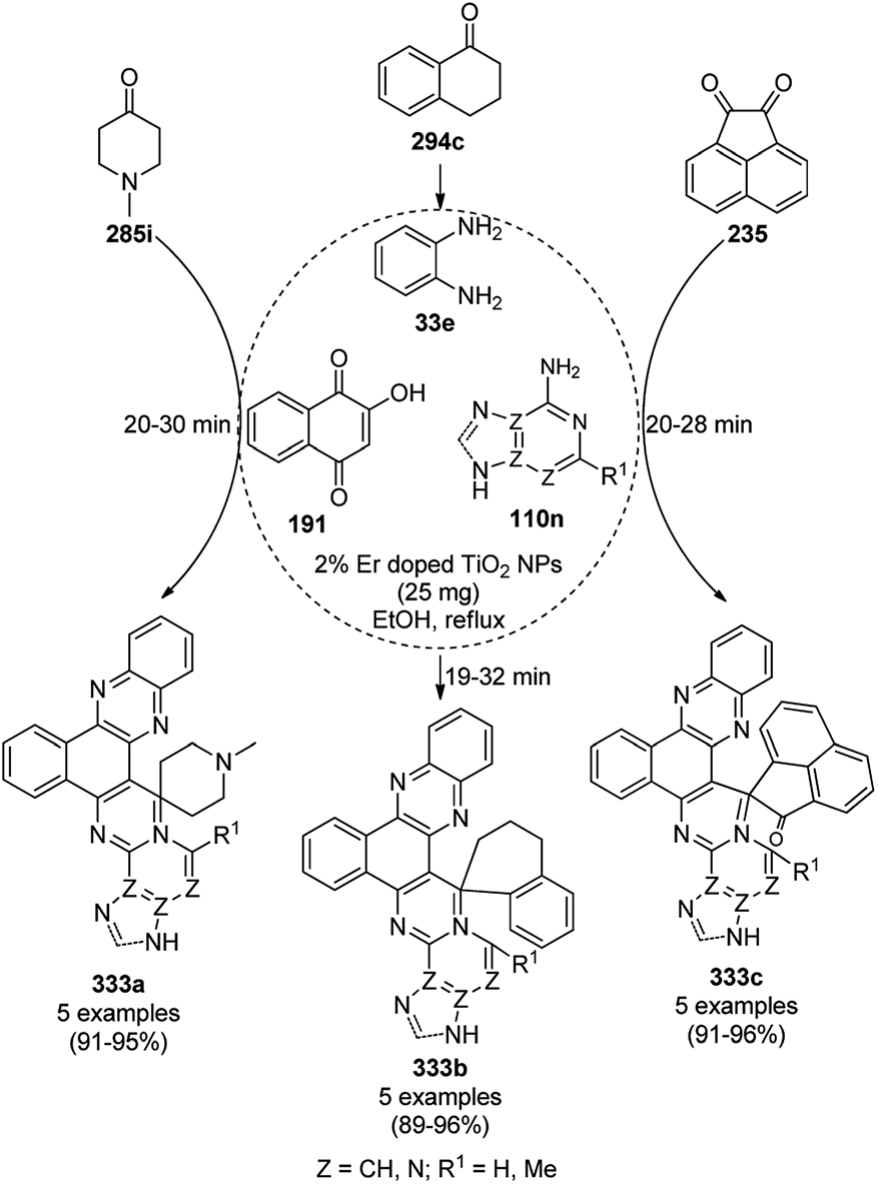
Synthesis of spiroannulated pyrimidophenazines (333a/b/c) catalyzed by Er-doped TiO_2_ NPs.

2% Er-doped TiO_2_ NP-catalyzed Michael addition–cyclization for the synthesis of spiroannulated pyrimidophenazines (333a/b/c) was reported by Kumar *et al.* (Scheme 230) in ethanol under reflux using 2-hydroxynaphthalene-1,4-dione (191), *o*-phenylene diamine (33e), aminopyridines derivatives (110n) and cyclic ketones (285i/294c/235).^409^

**Scheme 231:**
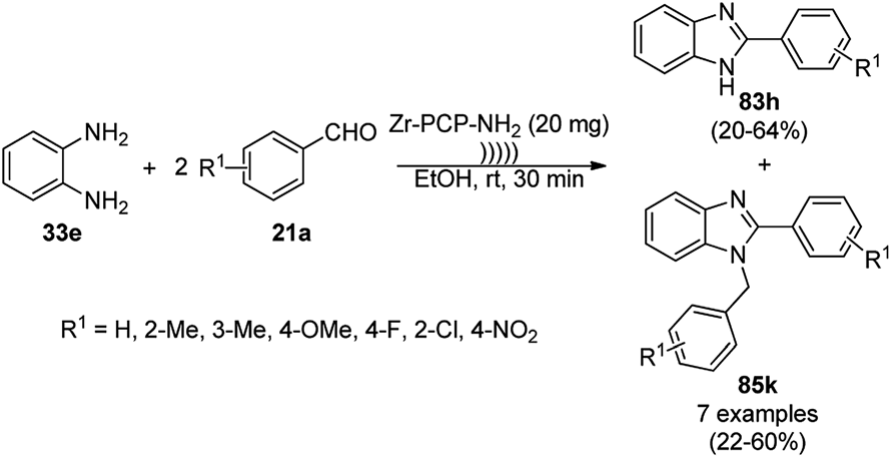
Ultrasound-mediated synthesis of benzo[*d*]imidazoles catalyzed by Zr-PCP-NH_2_.

The Zr-dependent porous coordination polymer ligated with amino-terephthaline (Zr-PCP-NH_2_)-catalyzed ultrasound-mediated synthesis of 2-phenyl benzimidazoles (83h), and 1,2-disubstituted benzimidazoles (85k) was reported recently by Mahmoudi *et al. via* the cyclocondensation of *o*-phenylene diamines with aryl carbaldehydes (21a) (Scheme 231).^410^ The yields of 83h and 85k were found to be higher under ultrasonic conditions rather that high speed stirring conditions. However, they did not compare the present non-selective protocol with selective methodology for the synthesis of 85k previously reported by Chakraborti *et al.* under aqueous conditions.^411^

**Scheme 232:**
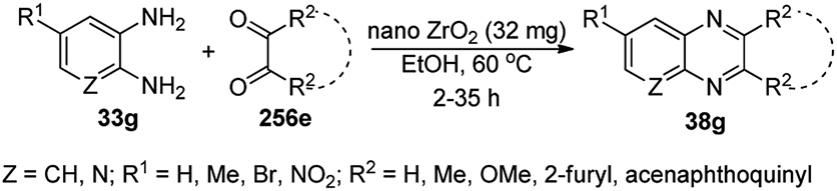
Synthesis of quinoxalines (38g) catalyzed by nano-ZrO_2_.

The zirconia NP-catalyzed synthesis of 2,3-disubstituted quinoxalines was successfully achieved *via* the ring closure of 1,2-diaminobenzenes (33g) with 1,2-diketones (256e) in ethanol at 60 °C (Scheme 232).^412^ However, the reaction with an electron-withdrawing nitro group containing 33g gave 38g in poor yields. The monoclinic-shaped NPs were prepared using ZrOCl_2_·8H_2_O, ethylene glycol and citric acid *via* the sol–gel process. The heterogeneous catalyst was separated *via* centrifugation and recycled for up to five runs.

**Scheme 233:**
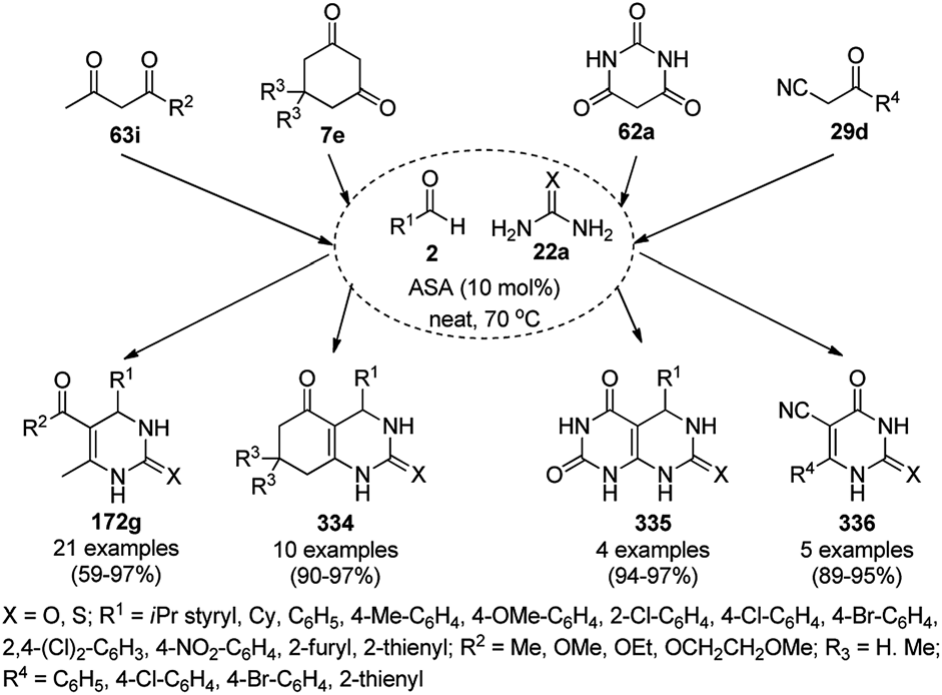
Synthesis of Biginelli and Biginelli-like products catalyzed by ASA.

Aluminosulfonic acid (ASA) NPs as a Brønsted acid catalyzed the Biginelli reactions of β-ketoesters (63i), aldehydes (2) and (thio)urea (22a) for the synthesis of dihydropyrimidinones (172g) under solvent-free conditions at 70 °C (Scheme 233).^413^ The same protocol was also explored for the synthesis of Biginelli-like products such as octahydroquinazolinones (334), pyrimido[4,5-*d*]pyrimidines (335) and tetrahydropyrimidines (336) in excellent yields. The treatment of sodium aluminate with chlorosulfonic acid resulted in the formation of ASA. The catalyst was recycled up to six times without a noticeable drop in its catalytic activity.

**Scheme 234:**
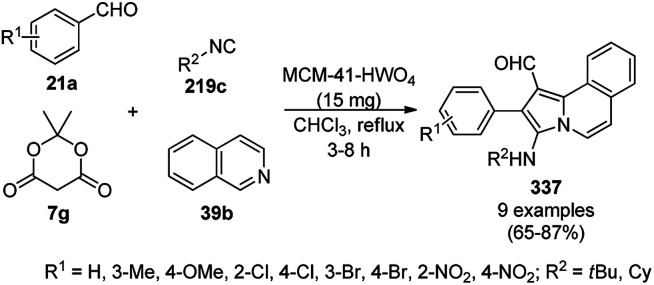
MCM-41-HWO_4_-catalyzed synthesis of pyrrolo[2,1-*a*]isoquinolines (337).

Karami *et al.* reported the preparation of tungstic acid-decorated MCM-41 (MCM-41-HWO_4_) NPs *via* the loading of tungstic acid with their previously prepared^414^ MCM-41, and their catalytic role in the synthesis of pyrrolo[2,1-*a*]isoquinolines (337) *via* the multi-component reactions of benzaldehydes (2), Meldrum's acid (7g), isoquinoline (39b) and isocyanides (219c) in reasonably good yields (Scheme 234).^415^ The durable catalyst was recycled up to six times with consistent yields.

**Scheme 235:**
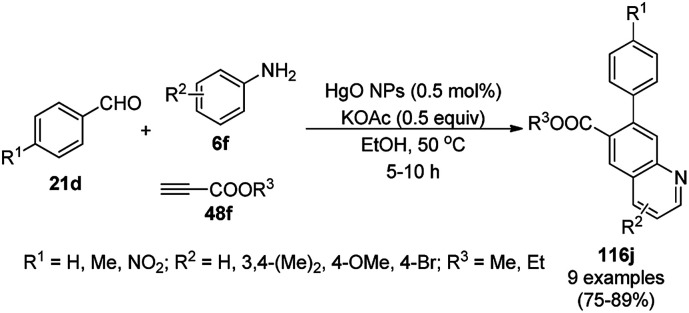
Synthesis of quinolines (116j) catalyzed by tetraphenylcyclopentandienone@HgO NPs.

A supramolecular assembly of tetraphenylcyclopentandienone with HgO NPs was reported for the synthesis of quinolines (116j) using benzaldehydes (21d), anilines (6f) and acetylene carboxylates (48f) (Scheme 235).^416^ This protocol was also extended for the synthesis of quinolones *via* C–H activation, which was used in synthesis of anti-inflammatory kynurenic acid methyl esters. The catalyst was recycled and reused up to three times without appreciable loss in its activity.

### 3.12 Bimetallic NP-catalyzed synthesis of heterocycles

**Scheme 236:**
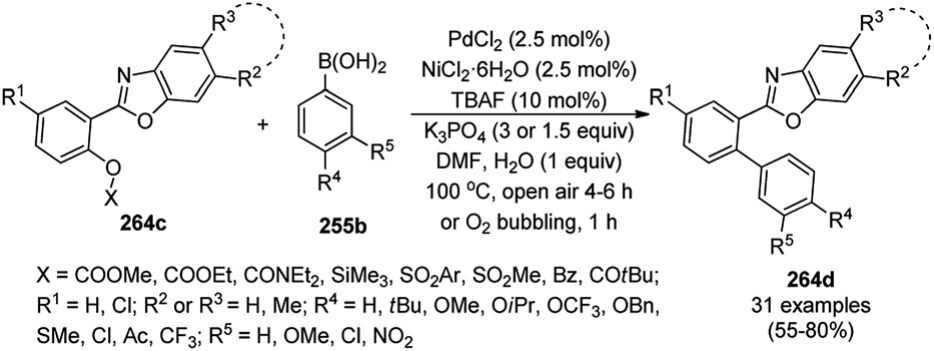
Synthesis of benzoxazole Ni–Pd binary NCs for C–O bond activation for the Suzuki–Miyaura cross-coupling of *o*-heterocycle-tethered sterically hindered aryl ester (264c), silyl ether, sulfonates, carbamate, and carbonates with aryl boronic acids (255b).

Chakraborti *et al.* utilized nanocatalysts for the synthesis of benzoxazoles (264d). Ni–Pd binary metallic NCs were reported as an effective catalyst system for C–O bond activation for the Suzuki–Miyaura cross-coupling reaction of *o*-benzoxazole-tethered aryl ester, silyl ether, sulfonate, carbamate, and carbonate (264c) as the electrophilic coupling partners (Scheme 236) with aryl boronic acids (255b).^417^ The protocol reported by Chakraborti *et al.* involved PdCl_2_ (2.5 mol%) and NiCl_2_·6H_2_O (2.5 mol%) as catalysts, tetrabutyl ammonium fluoride (TBAF, 10 mol%) as the stabilizer, potassium phosphate (K_3_PO_4_) as the base and dimethyl formamide (DMF) as the solvent. The versatility of the reaction was demonstrated on various substituted benzoxazoles and boronic acids. This reported protocol was found to be superior to the various Pd/Ni complexes reported for the Suzuki–Miyaura cross-coupling reaction of *o*-benzo[*d*]oxazole-tethered sterically demanding substrates compared to the conditions reported by Buchwald *et al.*,^418^ Fu *et al.*,^419^ Garg *et al.*,^420^ and Shi *et al.*^421^

**Scheme 237:**
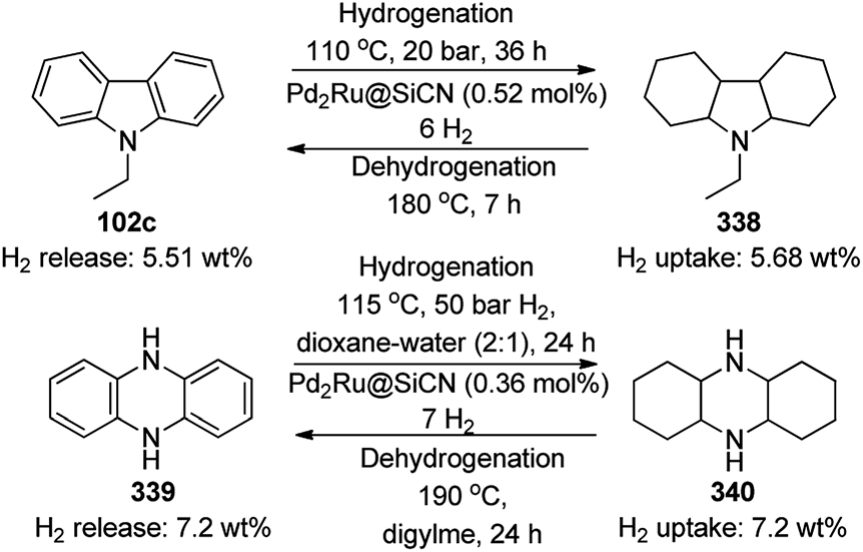
Reversible hydrogen uptake and release catalyzed by Pd_2_Ru@SiCN bimetallic catalyst.

Kempe *et al.* recently reported the reversible hydrogenation of *N*-ethyl carbazole (102c) into dodecahydro *N*-ethylcarbazole (338) using Pd_2_Ru NPs loaded on a silicon carbonitride (SiCN) matrix (Scheme 237).^422^ The same protocol was screened with various NPs, where Pd_2_Ru@SiCN was found to be superior for the desired conversion. The bimetallic NPs were synthesized *via* the cross linking and pyrolysis of a commercially available Ru complex and aminopyridinato Pd complex with polysilazane HTT180, which were further characterized *via* TEM, EDX, XRD, and HAADF. The same catalyst was further used for the hydrogenation of phenazine (339) and dehydrogenation of tetradecahydrophenazine (340).

**Scheme 238:**
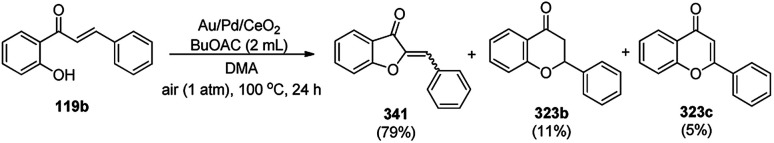
Synthetic transformation from chalcone (2′-hydroxychalcone, 119b) into aurone (341), flavanone (323b), and flavones (323c) using Au and Pd metal catalysts.^423^

Chalcone has been considered as the backbone of flavanoids from a synthetic point of view. Cyclization of chalcone yields aurone (341), flavanone (323b) and flavones (323c), but the synthesis of aurone is very difficult *via* 5-*exo-trig* cyclisation. This intramolecular α-olefinic C–H functionalization *via* Pd-catalyzed Wacker-type cyclization was achieved successfully using Au (5 mol%), Pd (5 mol%), conc. CeO_2_, and BuOAc (2 mL) in open air (1 atm) at 100 °C to yield aurone in 79% (Scheme 238). Here, the Pd catalyst assists in the transformation of aurone from chalcone, Au improves the catalytic activity and longevity, CeO_2_ inhibits 6-*endo-trig* cyclization, leading to the synthesis of flavone, and Pd on Au together inhibit the Au-catalyzed synthesis of flavone.^423^

**Scheme 239:**
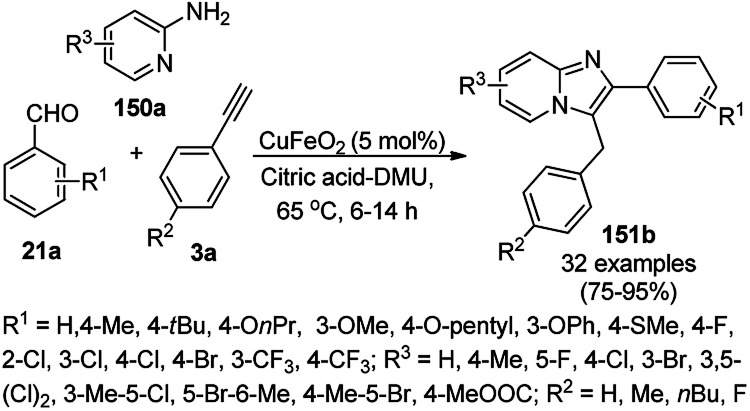
CuFeO_2_ NP-catalyzed synthesis of imidazo[1,2-*a*]pyridines (151b).

Zhang *et al.* reported the CuFeO_2_ NP-catalyzed green synthesis of imidazo[1,2-*a*]pyridines (151b) *via* the three-component reaction of 2-aminopyridine (150a), substituted benzaldehyde (21a) and substituted phenyl acetylene (3a) in a deep eutectic solvent such as citric acid-dimethyl urea (DMU) at 65 °C (Scheme 239).^424^ The NPs were prepared *via* a sol–gel process, followed by sequential annealing of a mixture of copper acetate Cu(CH_3_COO)_2_·H_2_O and ferric nitrate Fe(NO_3_)_3_·9H_2_O in ethanol and triethanolamine. Further, the NPs were completely characterized *via* XRD, TEM, SEM, FT-IR, and VSM. The reusability of the catalyst was studied for up to six catalytic runs, where a negligible 10% loss in product was observed after the sixth catalytic run. However, even after the sixth catalytic run, the catalyst retained its nanoparticulate character, as evident from the TEM images of the NPs after and before the catalytic runs.

**Scheme 240:**
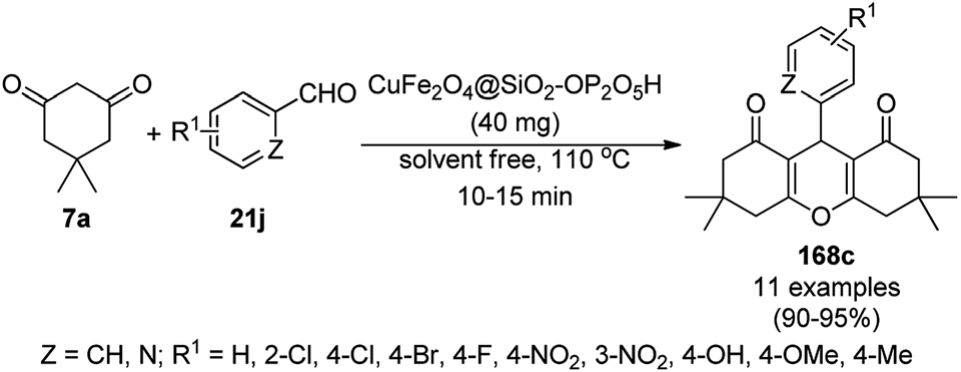
Synthesis of 1,8-dioxo-octahydroxanthenes (168c) using CuFe_2_O_4_@SiO_2_-OP_2_O_5_H MNP catalyst.

Copper ferrite (CuFe_2_O_4_) NPs have been identified as key catalysts for the synthesis of several heterocyclic compounds such as spiro-oxindoles,^425^ polysubstituted pyrroles,^426^ imidazo[1,2-*a*]pyridines^427^ and azaarenes.^428^ Davoodnia *et al.* synthesized CuFe_2_O_4_@SiO_2_-OP_2_O_5_H MNPs *via* the fusion of CuFe_2_O_4_@SiO_2_ and (P_2_O_5_)_2_, characterized them using various spectroscopic techniques, and further used them for the synthesis of 1,8-dioxo-octahydroxanthenes (168c) in excellent yields without solvent at 110 °C in a short reaction time (Scheme 240).^429^ The catalyst could be recovered through magnetic decantation and was activated after washing with solvent and drying at 60 °C for 1 h. The catalyst was found to retain its catalytic activity, yielding 91% 1,8-dioxo-octahydroxanthenes after the fourth catalytic run.

**Scheme 241:**
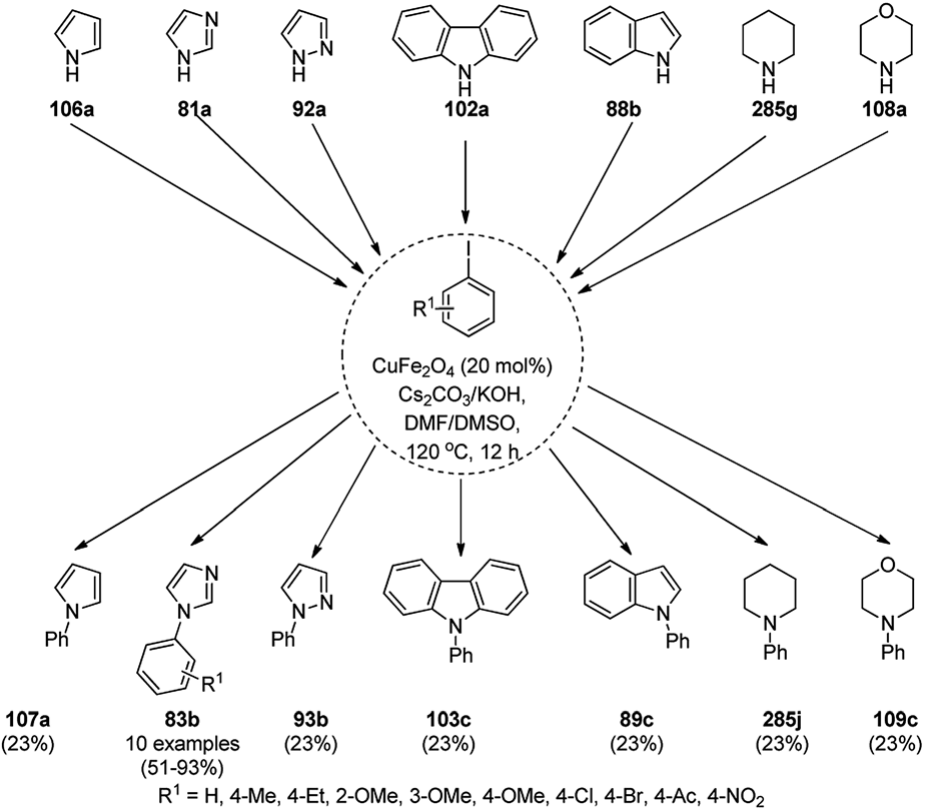
Synthesis of *N*-arylated heterocycles catalyzed by Cu_2_FeO_4_ MNPs.

Sun *et al.* reported the CuFe_2_O_4_ MNP-catalyzed synthesis of *N*-arylated heterocycles such as pyrrole (106a), imidazole (81a), pyrazole (92a), carbazole (102a), indole (88b), piperidine (285g) and morpholine (108a) *via* C–N bond formation using substituted iodobenzenes in DMF-Cs_2_CO_3_/DMSO-KOH at 120 °C under ligand-free conditions in moderate to excellent yields (Scheme 241).^430^ The MNPs were prepared *via* the treatment of Fe(NO_3_)_3_·9H_2_O with Cu(NO_3_)_2_·*X*H_2_O and citric acid followed by calcination at high temperature for 2 h and characterized *via* XRD, FT-IR, TEM, BET, and VSM. Various metallic ferrite MNPs were screened for the synthesis of the NPs, and CuFe_2_O_4_ resulted in the best catalyst. The CuFe_2_O_4_ MNPs were reused eight times for the *N*-arylation of imidazole with iodobenzene without loss in the yield of the *N*-phenyl imidazole.

**Scheme 242:**
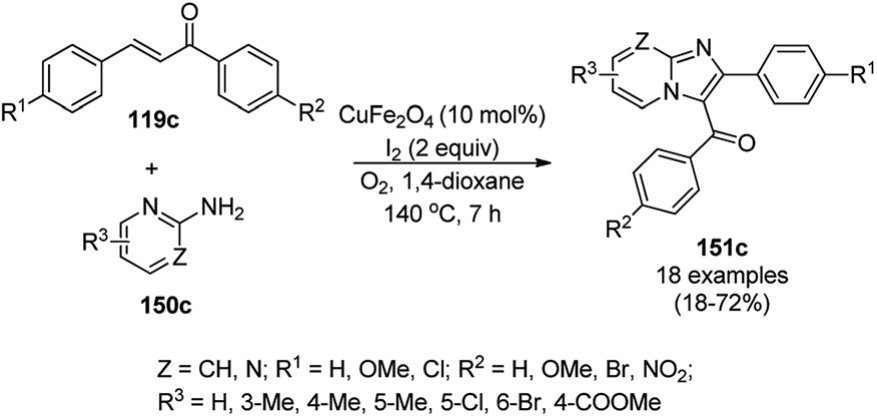
CuFe_2_O_4_ NP-catalyzed synthesis of aroylimidazo[1,2-*a*]pyrimidine/aroylimidazo[1,2-*a*]pyridines.

Commercially available copper ferrite MNP-catalyzed nucleophilic addition–cyclization–aromatization for the synthesis of aroylimidazo[1,2-*a*]pyrimidine/aroylimidazo[1,2-*a*]pyridines (151c) was achieved by Phan *et al.* (Scheme 242) using chalcones (119c) and 2-aminopyridines/pyrimidines (150c).^431^ CuFe_2_O_4_ maintained almost similar catalytic activity and intact structure (XRD) even after five catalytic reuses. This protocol was claimed to be superior compared to the reported protocols since it does not require ligands such as 1,10-phenanthroline,^432^ base such as K_2_CO_3_,^433^ and moisture-sensitive material such as AlCl_3_.^434^

**Scheme 243:**
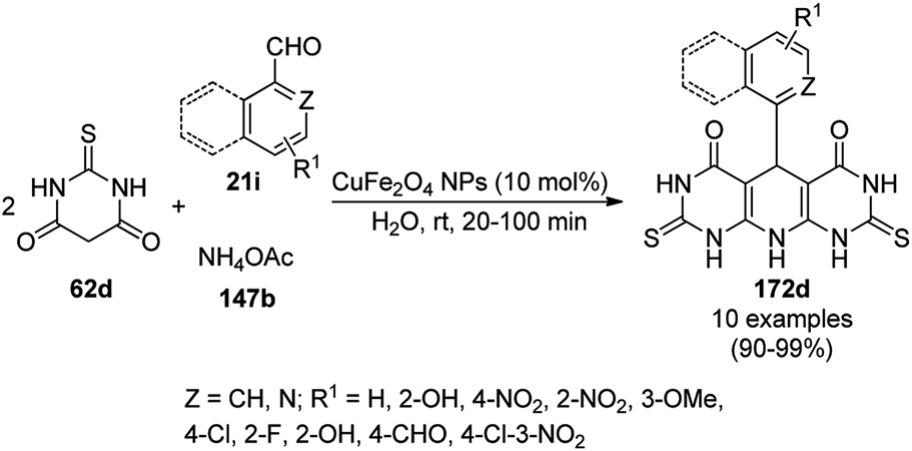
Synthesis of pyrido[2,3-*d*:6,5-*d*′]dipyrimidines (172d) catalyzed by CuFe_2_O_4_ MNPs.

Naeimi *et al.* reported the one-pot synthesis of pyrido[2,3-*d*:6,5-*d*′]dipyrimidines (172d) catalyzed by copper ferrite NPs under aqueous conditions at rt in excellent yields from the four-component reaction among substituted benzaldehyde (21i), 2-thiobarbituric acid (62d) and ammonium acetate (147b, Scheme 243).^435^ The MNPs were synthesized using cobalt nitrate (Cu(NO_3_)_3_·3H_2_O) and ferric chloride (FeCl_3_·3H_2_O) *via* the co-precipitation method in the presence of alkali, and their nanoparticulate behavior was confirmed *via* spectroscopic techniques such as AAS, XRD, field emission SEM, VSM, and TEM. Naeimi *et al.* demonstrated the separation and recovery of the catalyst using magnetic decantation for up to four catalytic cycles, giving excellent yields of the final product. Also, the present protocol catalyzed by CuFe_2_O_4_ was found to be superior to the Fe(iii)-doped, IL matrix-immobilized SiNP (Fe-MCM-41-IL)-catalyzed protocol.^333^

**Scheme 244:**
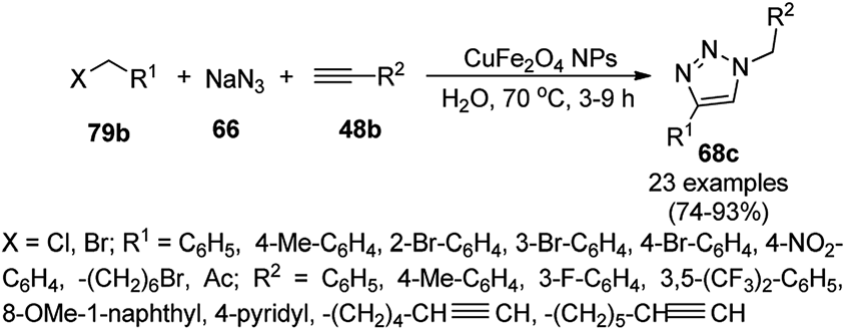
Cu_2_Fe_2_O_4_ NP-catalyzed Click reaction for the synthesis of 1,4-disubstituted 1,2,3-triazoles (68c).

Nageswar *et al.* reported the copper ferrite CuFe_2_O_4_ NP-catalyzed click reaction for the synthesis of 1,4-disubstituted 1,2,3-triazoles (96c) using alkyl or aryl alkyne (96b), aryl alkyl bromide or chloride (96a) and 10b using water as a green solvent at 70 °C in 74–93% yield (Scheme 244).^436^ The recyclability of the catalyst was studied for up to three catalytic runs with significant yields of the final product together with the significant recovery of the catalyst *via* magnetic decantation, which has become an alternative to centrifugation for the recovery of NCs. The SEM images of the fresh and recycled catalyst reflected that the morphological character of the catalyst remained intact even after the fourth catalytic run.

**Scheme 245:**
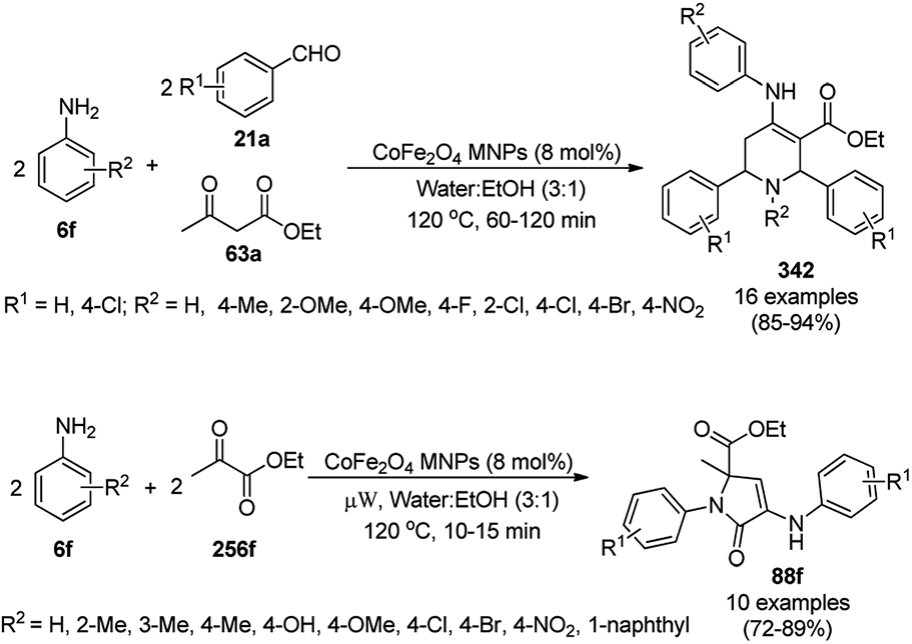
Synthesis of tetrahydro pyridines (342) and 1*H*-pyrrole derivatives (343) using microwave irradiation catalyzed by CoFe_2_O_4_.

El-Remaily *et al.* reported CoFe_2_O_4_ nanoparticles as a catalyst (0.08 mmol) for the synthesis of tetrahydropyridines (342) and substituted 1*H*-pyrrole derivatives (343) in water : EtOH as a co-solvent mixture (3 : 1) at 120 °C in good to excellent yields (Scheme 245).^437^ The CoFe_2_O_4_ MNPs were synthesized using Co(NO_3_)_2_·6H_2_O and Fe(NO_3_)_3_·9H_2_O followed by treatment with PEG-400 as a stabilizer or surfactant, and the prepared NPs were characterized *via* PXRD, TEM, HR-TEM, SAED, SEM, EDS, TGA and vibrating sample magnetometry. The synthesis of pyrrole derivatives was also attempted under reflux and microwave irradiation, where under microwave conditions, the reaction proceeded at a faster rate with the formation of the product in significant yields. The catalyst promoted the Mannich reaction by coordinating with the carbonyl oxygen and isolated from the reaction mixture *via* magnetic separation and used for up to five catalytic cycles without decay in its catalytic activity.

**Scheme 246:**
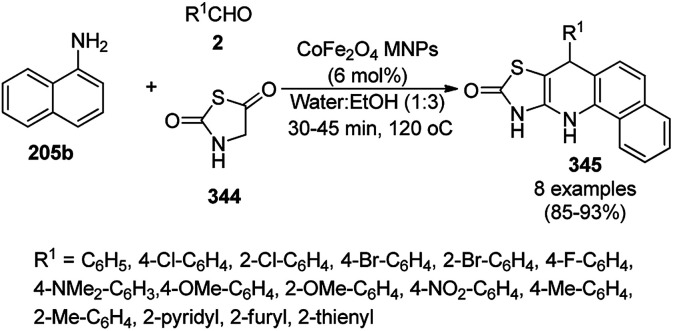
Synthesis of tetrahydrobenzo[*h*][1,3]thiazolo[4,5-*b*]quinolin-9-ones (345) reported by Hamad *et al.*

Hamad *et al.* reported superparamagnetic CoFe_2_O_4_ MNPs as a new catalyst for the synthesis of tetrahydrobenzo[*h*][1,3]thiazolo[4,5-*b*]quinolin-9-ones (345) using naphthalen-1-amine (205b), substituted aryl aldehyde (2) and thiazolidinediones (344) at 120 °C in aqueous ethanol as the ultimate green solvent (Scheme 246).^438^ The synthesis of the cobalt ferrite (CoFe_2_O_4_) MNPs was achieved by Hamad and his co-workers *via* a sonochemical and co-precipitation method using CoCl_2_·6H_2_O, FeSO_4_·7H_2_O and Fe_2_(SO_4_)_3_·7H_2_O and well characterized *via* XRD, SEM, EDX, TEM, FT-IR and VSM. After the completion of the reaction, the NCs were isolated by magnetic decantation and successfully utilized for up to six catalytic cycles without loss in their activity. With respect to the reaction mechanism, the NCs act as a Lewis acid catalyst and activate the carbonyl carbons of aldehydes and thiazolidinedione to expedite the rate of the Knoevenagel condensation.

**Scheme 247:**
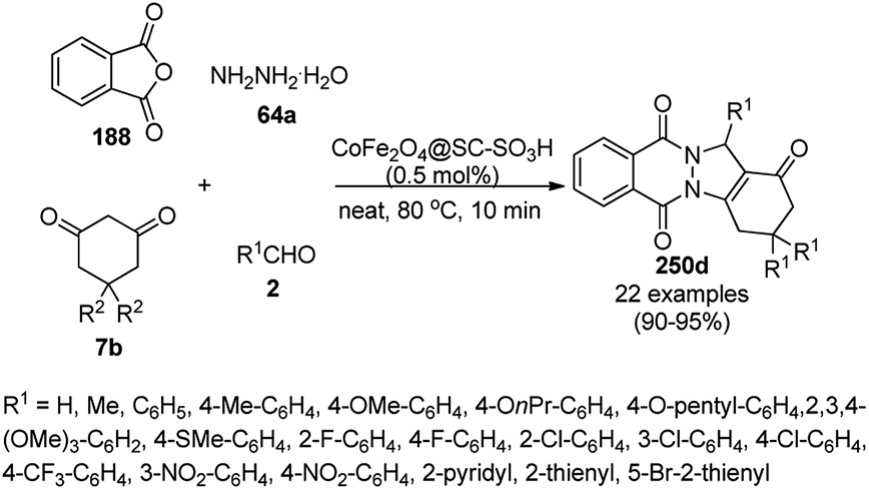
One-pot synthesis of 2*H*-indazolo[2,1-*b*]phthalazine-triones (250d) using CoFe_2_O_4_@CS-SO_3_H.

The one-pot, green and efficient synthesis of 2*H*-indazolo[2,1-*b*]phthalazine-triones (250d) *via* a multicomponent reaction was reported by Zhao *et al.*^439^ using phthalic anhydride (188), 64d, 1,3-cyclohexanediones (7b) and aryl aldehydes (2) with magnetic CoFe_2_O_4_ chitosan sulfonic acid nanoparticles (CoFe_2_O_4_@SC-SO_3_H) as the nanocatalyst. Various attempts were made to optimize the reaction conditions, and the highest yield (95%) of 2*H*-indazolo[2,1-*b*]phthalazine-triones was observed under solvent-free conditions at 80 °C in 10 min using 0.5 mol% of nanocatalyst (Scheme 247). The heterogeneous catalyst was reused after separation using a strong external permanent magnet and reactivation. No obvious loss in catalytic activity was observed over the five catalytic runs using the recovered catalyst. The authors also claimed the better efficiency of the developed method with higher yields in a shorter period with a lower catalytic loading in comparison with the reported literature^440–443^ for the synthesis of 2*H*-indazolo[2,1-*b*]phthalazine-triones.

**Scheme 248:**
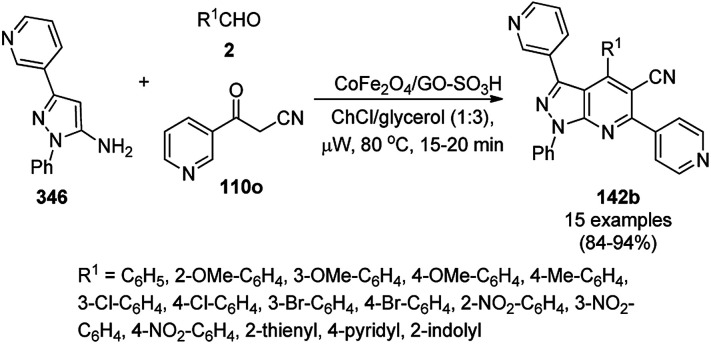
Green synthesis of 3,6-di(pyridin-3-yl)-1*H*-pyrazolo[3,4-*b*]pyridine-5-carbonitriles (142b) reported by Zhang *et al.*

Zhang *et al.* reported that graphene oxide sulfonic acid nanoparticles (CoFe_2_O_4_/GO-SO_3_H) as a Lewis acid catalyzed synthesis of 3,6-di(pyridin-3-yl)-1*H*-pyrazolo[3,4-*b*]pyridine-5-carbonitriles (142b) from 1-phenyl-3-(pyridin-3-yl)-1*H*-pyrazol-5-amines (346), aryl or heteroaryl aldehyde (2) and 3-oxo-3-(pyridin-3-yl)propanenitrile (110o) using a deep eutectic co-solvent mixture of choline chloride ChCl/glycerol (1 : 3) under microwave irradiation at 80 °C in 84–94% yield (Scheme 248).^444^ Graphene oxide (GO) was prepared following the reported protocol by Hummers and Offeman^445^ treated with FeCl_3_·H_2_O and CoCl_2_·6H_2_O to obtain CoFe_2_O_4_-GO NPs, which were further anchored with chlorosulfonic acid to obtain CoFe_2_O_4_/GO-SO_3_H MNPs. The final MNPs were well characterized *via* XRD, SEM, TEM and VSM. Zhang *et al.* reported the efficiency of the developed MNPs for up to eight consecutive catalytic runs without appreciable loss in its catalytic activity.

**Scheme 249:**
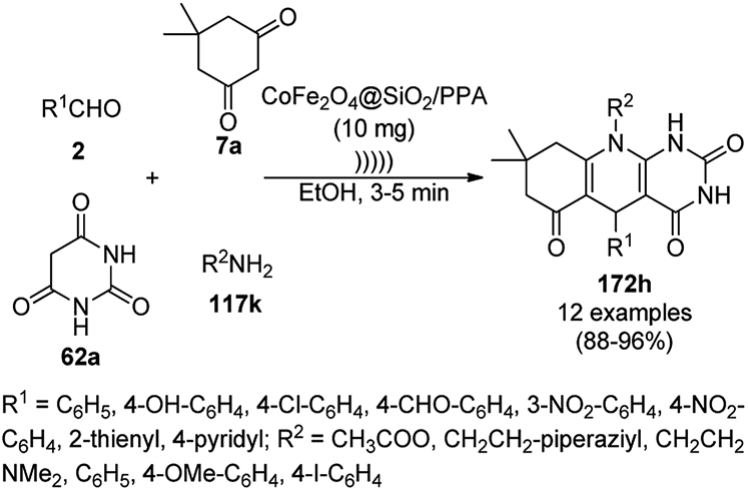
Ultrasonic wave-mediated synthesis of dihydropyrimido[4,5-*b*]quinolinetriones (172h).

Cobalt ferrite NPs were prepared *via* the co-precipitation of FeCl_3_·6H_2_O and CoCl_2_·6H_2_O in aqueous caustic soda and treated with TEOS, and polyphosphoric acid to obtain polyphosphoric acid-functionalized silica-coated CoFe_2_O_4_ NPs (CoFe_2_O_4_@SiO_2_/PPA).^446^ They were used in the rapid synthesis of dihydropyrimido[4,5-*b*]quinolinetriones (172h) *via* the four-components reaction of carbaldehydes (2), barbituric acids (62a), amines (117k) and dimedone (7a, Scheme 249). Hot filtration tests revealed that the reaction could not proceed without the assistance of the catalyst.

**Scheme 250:**
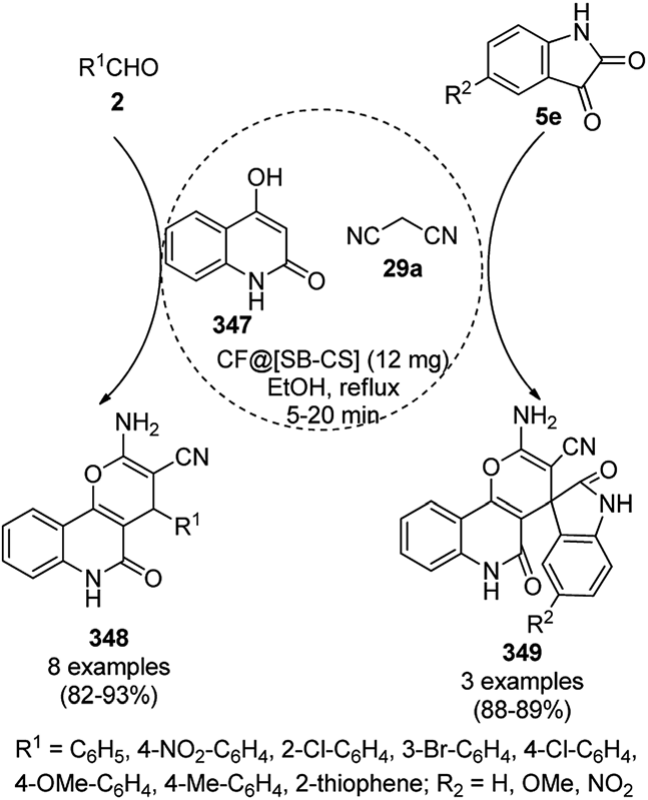
Synthesis of pyrano[3,2-*c*]quinolines (348) and spiro-oxindoles (349) catalyzed by cobalt-ferrite NPs.

The catalytic use of cobalt ferrite NPs encapsulated in a chitosan-derived shell (CF@[SB-CS]) was reported for the successful synthesis of pyrano[3,2-*c*]quinolines (348) and spiro-oxindoles (349) using 4-hydroxyquinolin-2-one (347), 29a, aldehydes (2) and 5-substituted isatins (5e, Scheme 250).^447^*N*-(4-sulfonylbutyl)chitosan (SB-CS) in 3% acetic acid was treated with a basic solution of FeCl_3_·6H_2_O and CoCl_2_·6H_2_O to obtain the final CF@[SB-CS]. The recyclable catalyst was tested for up to six runs using the model reaction among 347, 29a, and 4-nitrobenzaldehyde with 89–93% yield of pyrano[3,2-*c*]quinoline. The array of amino and hydroxyl groups on the surface of the NCs facilitates the concerted proton exchange through HB and Thorpe–Ziegler-type cyclization.

**Scheme 251:**
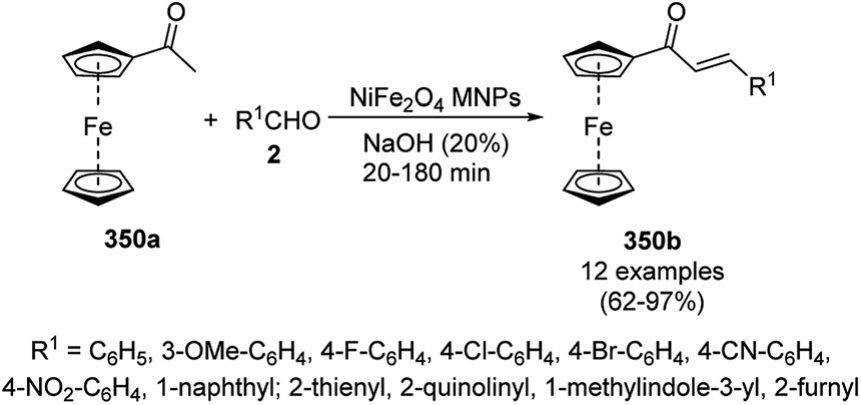
NiFe_2_O_4_ MNP-catalyzed Claisen–Schmidt condensation for the synthesis of the chalcones (350b).

Among the ferrite NPs, nickel ferrite (NiFe_2_O_4_) NPs have been also reported for the synthesis of aza-heteroarenes such as spiro[indoline-3,3′-pyrrolizine],^448^ pyrimido[1,2-*a*]benzimidazoles,^449^ pyrazolonethioethers,^450^ 2-alkoxyimidazo[1,2-*a*]pyridines,^451^ and pyrano[3,2-*c*]chromen-5(4*H*)-ones.^452^ Abu-Dief *et al.* reported the synthesis of acetylferrocene chalcones (350b) *via* the Claisen–Schmidt condensation of aromatic or heteroaromatic aldehydes (2) with acetyl ferrocene (350a) using a catalytic amount (10 mol%) of as-prepared nickel ferrite (NiFe_2_O_4_) NPs as the Lewis acid catalyst (Scheme 251).^453^ The authors prepared the NiFe_2_O_4_ nanoparticles (2–7 nm) *via* a hydrothermal route and characterized them using powder XRD, SEM, EDX and TEM. The scope of the optimized protocol was extended for the synthesis of acetylferrocene chalcones using a variety of substituted aromatic aldehydes (2) with electron-donating, electron-withdrawing and halogen-containing groups and also aldehydes having a heteroaromatic nucleus such as thiophene, quinoline, indole and furan. The catalyst was recovered using magnetic decantation and could be reutilized successfully for up to six catalytic cycles. The authors also demonstrated the scope of the synthesized chalcones for anti-tumor activity against colon cancer, breast cancer and liver cancer.

**Scheme 252:**
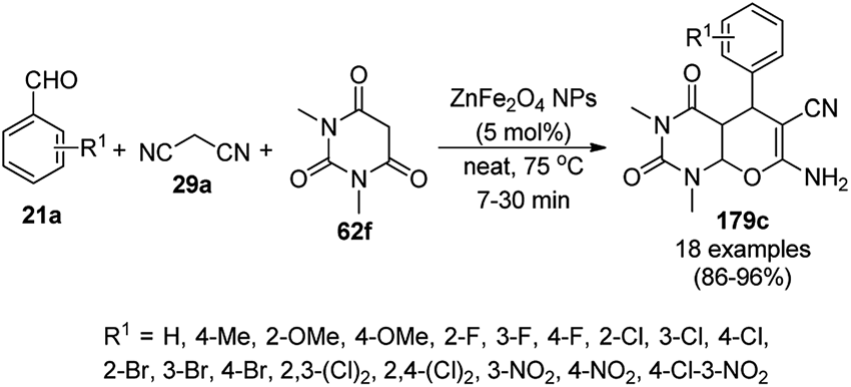
ZnFe_2_O_4_ NP-catalyzed synthesis of pyrano[2,3-*d*]pyrimidines (179c) reported by Khazaei *et al.*

Khazaei *et al.* reported the synthesis of pyrano[2,3-*d*]pyrimidines (179c) *via* Knoevenagel condensation catalyzed by ZnFe_2_O_4_ nanoparticles under neat conditions at 75 °C using aromatic aldehydes (21a), 29a and 1,3-dimethylpyrimidine-2,4,6-triones (62f, Scheme 252).^454^ They synthesized ZnFe_2_O_4_ NPs *via* the reaction of ZnCl_2_ and FeCl_3_ 6H_2_O and characterized them *via* FT-IR, solid-state UV, XRD, EDS and SEM. However, the authors did not report the reusability of the developed catalyst, which can increase the value of this protocol for one-pot three-component substrates.

**Scheme 253:**
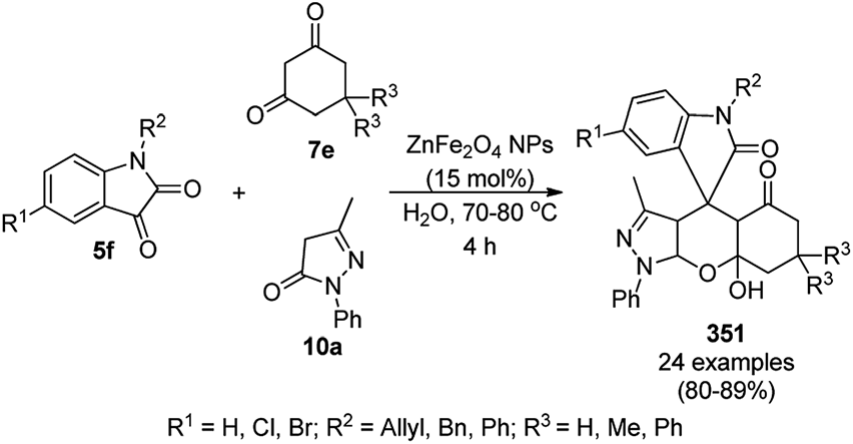
Aqueous pyrazole-fused chromenes (351) catalyzed by zinc ferrite NPs.

ZnFe_2_O_4_ NP-catalyzed Knoevenagel–Michael-cyclization for the synthesis of pyrazole-fused chromenes (351) was achieved *via* the three-component reaction among isatins (5f), dimedone (7e) and *N*-phenyl pyrazolones (10a) (Scheme 253).^455^ The catalyst could promote the above reaction *via* its Lewis acid co-coordination ability with the carbonyl or enolic oxygen of the reactants or formed intermediates. The stable catalyst was recycled up to eight times.

**Scheme 254:**
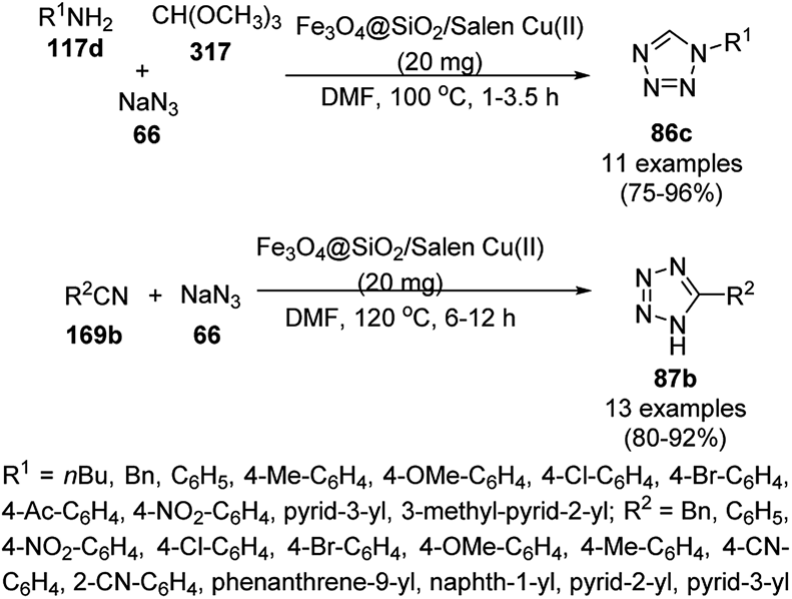
Synthesis of 1-and 5-substituted tetrazoles (86c/87b) catalyzed by the salen complex of Cu(ii) supported on superparamagnetic Fe_3_O_4_@SiO_2_ NPs.

Sardarian *et al.* reported the synthesis of 1-substituted tetrazoles (86c) from substituted anilines (117d), triethyl orthoformate (317) and 66 using a salen complex of Cu(ii) decorated on Fe_3_O_4_@SiO_2_ NPs^456^ in DMF as the solvent at 100 °C (Scheme 254). The same protocol was also explored for the synthesis of 5-substituted tetrazoles (87b) from substituted benzonitriles (169b) and 66. The salen complex of Cu(ii) was prepared using a Schiff base and copper acetate, which were treated with Fe_3_O_4_ NPs, and these NPs were characterized *via* TEM, SEM, FE-SEM, DLS, and VSM. The catalyst was recycled under magnetic influence up to seven times without significant catalytic decay using the model reaction of *p*-methoxy aniline, 317, and 66. A negligible amount of leached copper was observed after the first and seventh catalytic runs from the reaction mixture, as confirmed by ICP. The comparison of the literature and the present work developed by Sardarian *et al.* reflected the significance of the Fe_3_O_4_@SiO_2_ NPs over other catalysts such as natrolite zeolite,^457^ and indium triflate In(OTf)_3_ (ref. 458) reported in the literature.

**Scheme 255:**
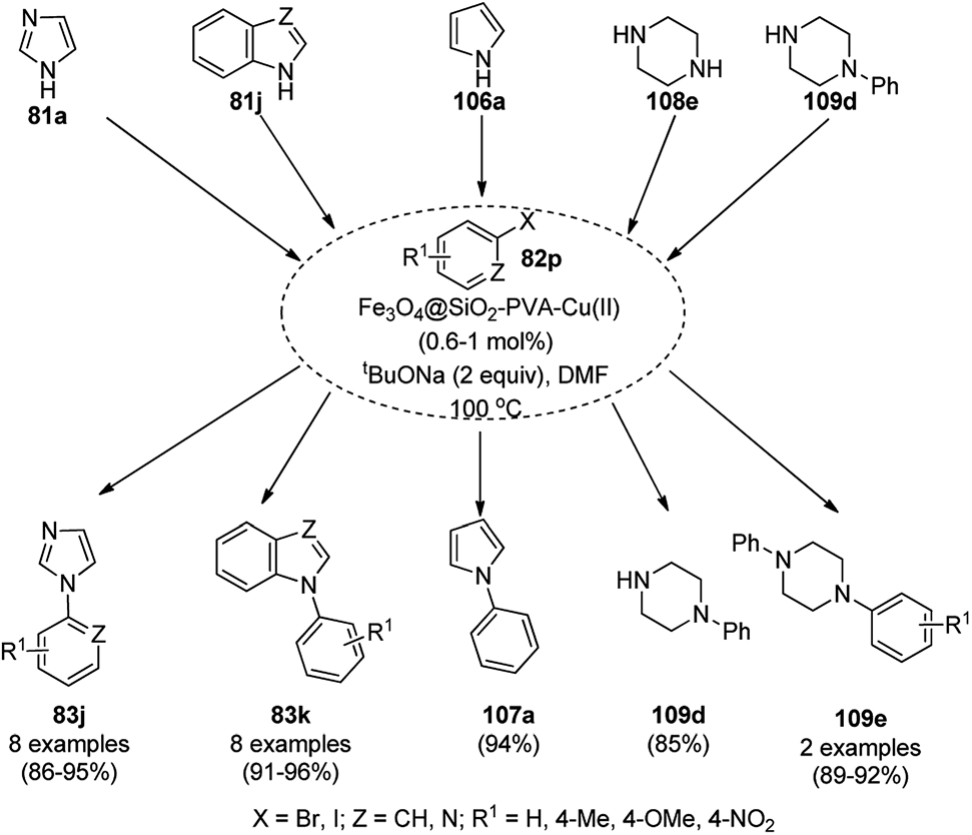
Synthesis of *N*-arylated heterocycles catalyzed by Fe_3_O_4_@SiO_2_-TCT-PVA-Cu(ii).

**Scheme 256:**
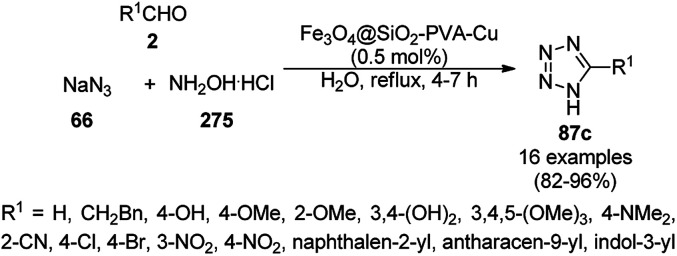
Synthesis of 5-aryl tetrazoles (87c) catalyzed by Fe_3_O_4_@SiO_2_-TCT-PVA-Cu(ii).

Esmaeilpour *et al.* reported the catalytic use of Fe_3_O_4_@SiO_2_-TCT-PVA-Cu(ii) for the *N*-arylation of N-containing heterocycles such as imidazoles (81a), indoles (81j), pyrrole (106a), piperazine (108e) and *N*-phenyl piperazines (109d) using aryl halides (82p) as the aryl coupling partner and sodium *tert*-butoxide as the base in DMF at 100 °C in 86–96% yield (Scheme 255).^459^ The same protocol was also extended for the synthesis of 5-phenyl 1*H*-tetrazoles (87c) by the reaction among substituted benzaldehyde (2), sodium azide (66) and hydroxylamine hydrochloride (275) in water under reflux *via* click reaction (Scheme 256). The NPs were prepared by coating Fe_3_O_4_ NPs with silica followed by their treatment with (3-chloropropyl)trimethoxysilane and 3-(3-hydroxy-propylamino)-propan-1-ol to obtain NPs having free hydroxyl groups. Further treatment of these NPs with 2,4,6-trichlorotriazone (TCT), polyvinyl alcohol (PVA) and Cu(OAc)_2_ yielded Fe_3_O_4_@SiO_2_-TCT-PVA-Cu(ii). The final characterization was achieved with FT-IR, XRD, TEM, FE-SEM, UV-Vis, EDX, and TGA. The catalyst was recovered using an external magnetic field and reused for up to seven cycles without appreciable loss in its catalytic activity, where it retained its nano-size and shape (FE-SEM), copper content (ICP) and structural integrity (FT-IR). The Cu catalyzed Ullmann coupling for the synthesis of 1-phenyl-1*H*-imidazole (83j: R^1^ = H, Z = CH)^95,108–110,460,461^ and 5-substituted-1*H*-tetrazoles (87c)^462–464^ was claimed to be operational at a lower temperature in a greener solvent under milder conditions in comparison with that in previous reports. Previously, Esmaeilpour *et al.* attempted the synthesis of *N*-arylated heterocycles and 5-aryl tetrazole following a similar approach using silica-coated Fe_3_O_4_ NPs grafted with 1,4-dihydroxyanthraquinone-Cu(ii) in 0.5–0.8 mol%.^465^ In continuation, they further reported theophylline-supported Fe_3_O_4_@SiO_2_ NPs for the one-pot synthesis of spirooxindoles and phenazines.^466^

**Scheme 257:**
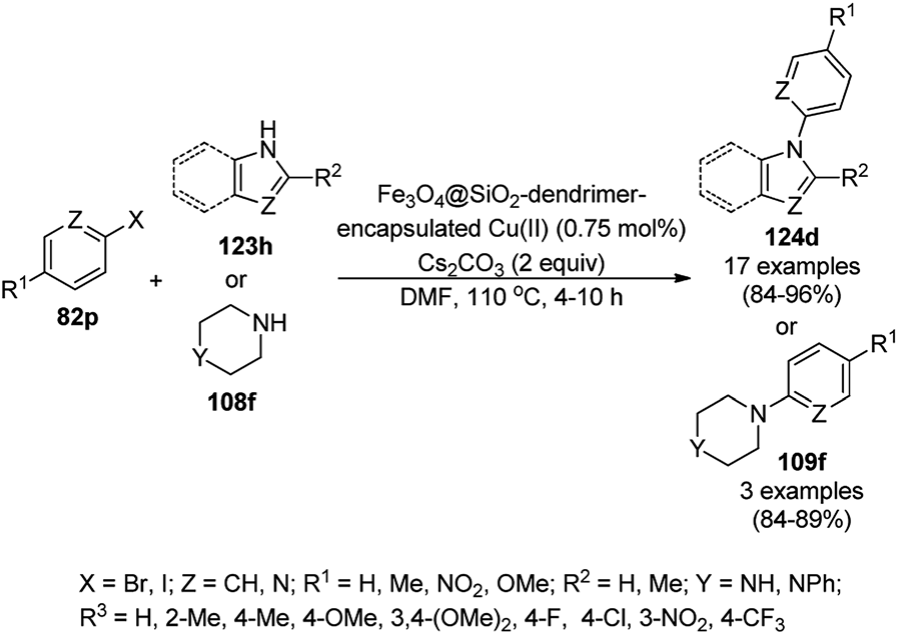
Synthesis of arylated N-containing heterocycles (124d/109f) using Fe_3_O_4_@SiO_2_-dendrimer-encapsulated Cu(ii) catalyst.

**Scheme 258:**
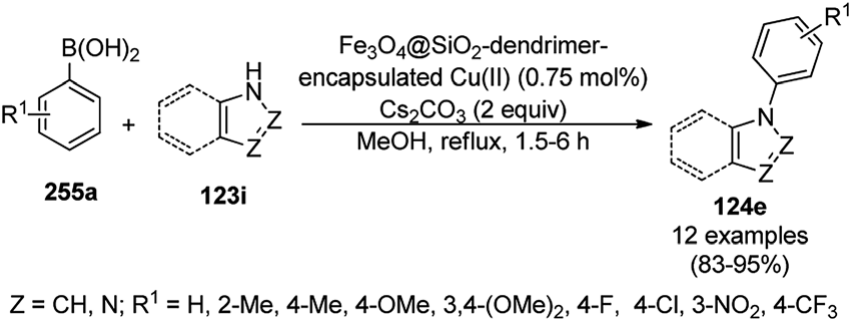
Fe_3_O_4_@SiO_2_-dendrimer-encapsulated Cu(ii)-catalyzed synthesis of benzazoles (124e).

**Scheme 259:**
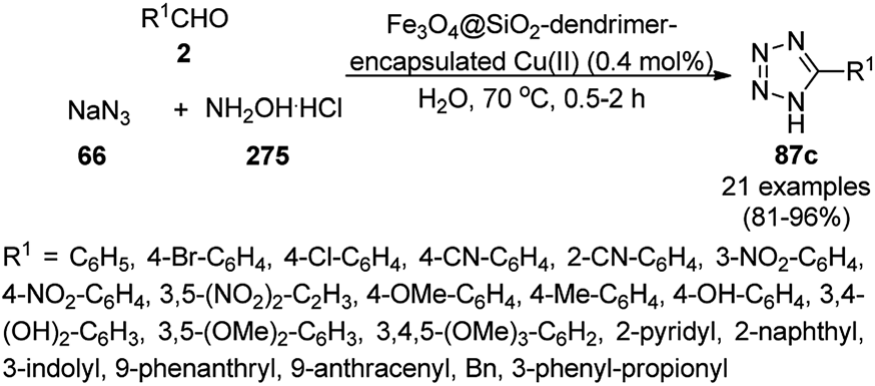
Synthesis of arylated 1,2,3,4-tetrazoles (87c).

*N*-arylation of various N-containing heterocycles (123h/108f) with aryl halides (82p) was attempted using Fe_3_O_4_@SiO_2_-dendrimer-encapsulated Cu(ii) as the catalyst, Cs_2_CO_3_ (2 equiv.) as the base and DMF as the solvent to yield twenty different arylated N-containing heterocycles (124d/109f) in 84–96% yield (Scheme 257). The same methodology was also applied for the synthesis of twelve different arylated N-containing heterocycles (123i) using aryl boronic acids (255a) with methanol as the solvent under reflux in 83–95% yield in 1.5–6 h (Scheme 258). Twenty-one substituted aryl tetrazoles (87c) were synthesized using substituted benzaldehyde (2), 66 and hydroxylamine hydrochloride (275) with same catalyst in water at 70 °C in 81–96% yield (Scheme 259). The oxidative addition of the aryl halide generated a transient Cu(iii) species and then C–N bond product was formed *via* reductive elimination together with the regeneration of the Cu(i) species. The authors claimed that the use of a magnetic filtration pad allows easier separation, which can be recycled without loss in its catalytic activity.^467^ The authors also compared the catalytic efficiency of the previously reported protocols catalyzed by CuFAP,^92^ silica-immobilized Cu complexes,^460^ cellulose-supported Cu(0),^468^ polyaniline-supported CuI,^112^ Cu(ii)–NaY zeolite,^95^ Cu_2_O,^109^ nano-CuO,^110^ metformin/CuI complex,^469^ bis(μ-iodo)bis((−)-sparteine)dicopper(i) [Cu_2_I_2_((−)-sparteine)_2_],^470^ Cu(OAc)_2_ H_2_O/8-hydroxyquinoline^471^ and CuI with ligand magnetic nanoparticle-supported proline (MNP-3)^99^ for the *N*-arylation of imidazoles with iodobenzene with their developed protocols and claimed that the Fe_3_O_4_@SiO_2_-dendrimer-encapsulated Cu(ii) catalyst is superior since it can complete the reaction within 4 h with 96% yield of *N*-phenyl imidazole.

**Scheme 260:**
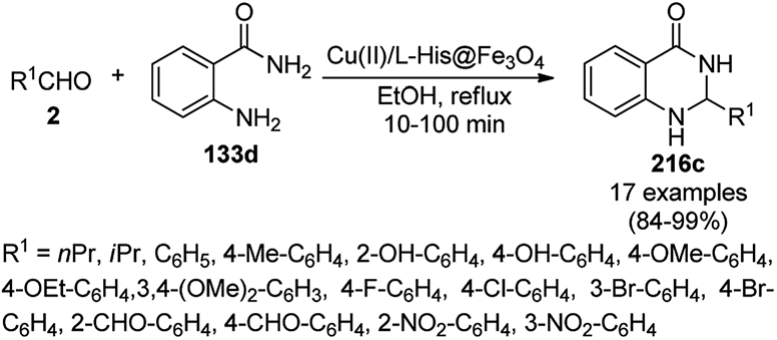
Synthesis of 2,3-dihydroquinazolin-4(1*H*)-ones (216c) catalyzed by Cu(ii)/l-His@Fe_3_O_4_ NCs.

**Scheme 261:**
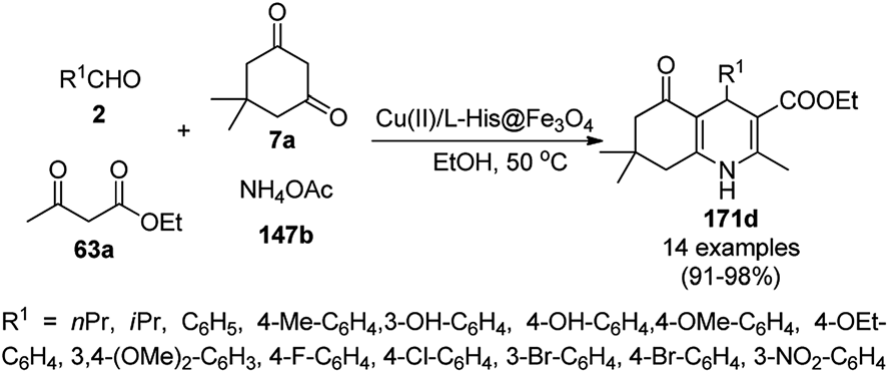
Synthesis of polyhydroquinolines (171d) catalyzed by Cu(ii)/l-His@Fe_3_O_4_ NCs.

**Scheme 262:**
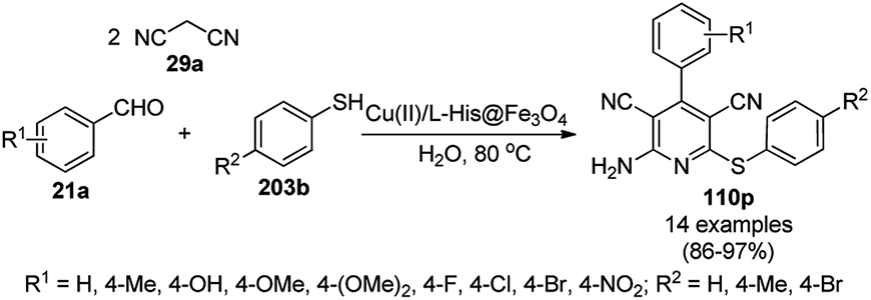
Synthesis of 2-amino-3,5-dicarbonitrile-6-thio-pyridines (110p) catalyzed by Cu(ii)/l-His@Fe_3_O_4_ NCs.

Ghorbani-Choghamarani *et al.* reported the synthesis of 2,3-dihydroquinazolin-4(1*H*)-ones (216c), polyhydroquinolines (171d) and 2-amino-3,5-dicarbonitrile-6-thio-pyridines (110p) using copper(ii)-l-histidine supported on Fe_3_O_4_ [Cu(ii)/L-His@Fe_3_O_4_] in green solvents (Scheme 260).^472^ The synthesis of 216c was achieved from aryl or aliphatic aldehydes (2) and *o*-aminobenzamides (133d) in ethanol under reflux in good to excellent yields. 171d was synthesized using 2, 7a, 63a and 147b in ethanol at 50 °C (Scheme 261), whereas the synthesis of 110p was achieved using 29a, substituted thiophenols (203b), and 21a with water as the solvent at 80 °C in 86–97% yield (Scheme 262). l-His@Fe_3_O_4_ was prepared by treating acryloyl chloride-coated MNPs having Fe_3_O_4_ (MNP-acryloxyl) with l-histidine and CuCl_2_. The final NPs were fully characterized *via* TGA, VSM, EDS, XRD, FT-IR and SEM. The reusability of the catalyst was studied for up to six catalytic cycles without significant loss in its catalytic activity for the synthesis of 2,3-dihydroquinazolin-4(1*H*)-ones and polyhydroquinolines.

**Scheme 263:**
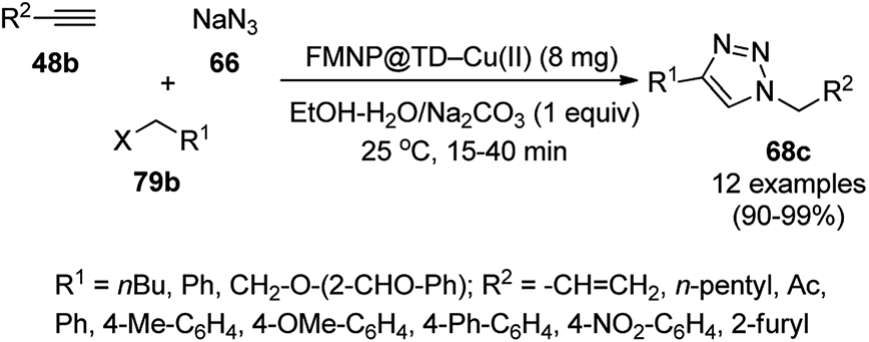
FMNP@TD–Cu(ii) catalyzed synthesis of regioselective 1,4-disubstituted 1,2,3-triazoles (68c).

Bahrami *et al.* reported the one-pot three-component regioselective synthesis of 1,4-disubstituted 1,2,3-triazoles (68c) *via* click synthesis using a heterogeneous nanocatalyst, copper-immobilized ferromagnetic triazine dendrimer FMNP@TD-Cu(ii) nanoparticles, with 66, arylalkyl/alkyl halides (79b) and alkynes (48b, Scheme 263).^473^ The synthesized catalyst was well characterized *via* TEM, SEM, XRD, FT-IR and EDX. The catalyst was recovered *via* simple filtration and drying. The activity of the catalyst was found to be retained up to 93% after the sixth catalytic run. The present catalytic protocol developed by Bahrami *et al.* could enhance the yield and reduce the reaction time for the synthesis of derivatives of 68c (R^1^ = R^2^ = Ph)^474,475^ and of 68c (R^1^ = Ph, R^2^ = -COPh)^475,476^ compared to that previously reported.

**Scheme 264:**
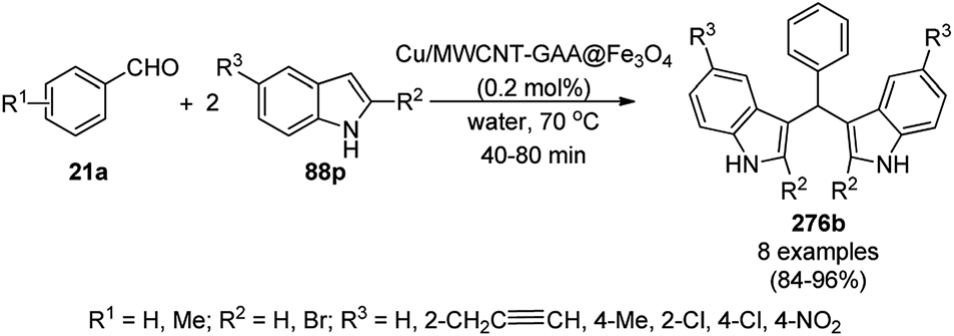
Aqueous synthesis of bis(indolyl)methanes (276b).

**Scheme 265:**
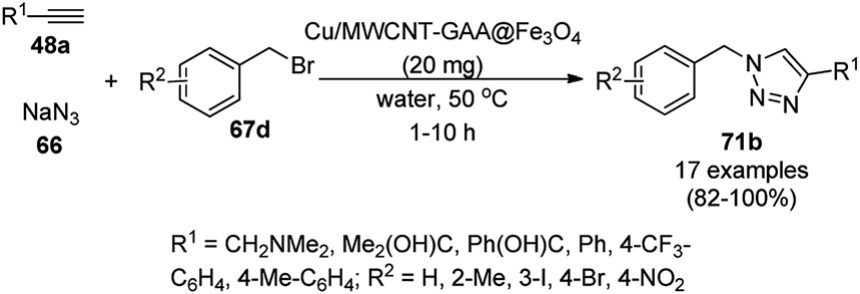
Aqueous synthesis of *N*-arylalkyl 1,2,3-triazoles (71b).

Shaabani *et al.* reported the catalytic use of CuNP-tethered magnetite guanidine acetic acid-decorated MWCNTs (Cu/MWCNTGAA@Fe_3_O_4_) for the synthesis of bis(indolyl)methanes (276b) *via* condensation of two equivalents of indoles (88p) and benzaldehydes (21a, Scheme 264) and 1,2,3-triazoles (71b) *via* 1,3-dipolar cycloaddition using alkynes (48a), azide (66) and lachrymator benzyl bromides (67d, Scheme 265).^477^ To synthesized final NPs, they loaded Fe_3_O_4_ NPs on MWCNTs followed by co-ordination with copper using CuCl_2_·2H_2_O. The stability and reusability of the NPs were observed for up to four runs for the click reaction. Subsequently, Voskressensky *et al.* used the same catalyst for the cycloaddition of 1-aroyl-3,4-dihydroisoquinoline and ynones.^478^

**Scheme 266:**
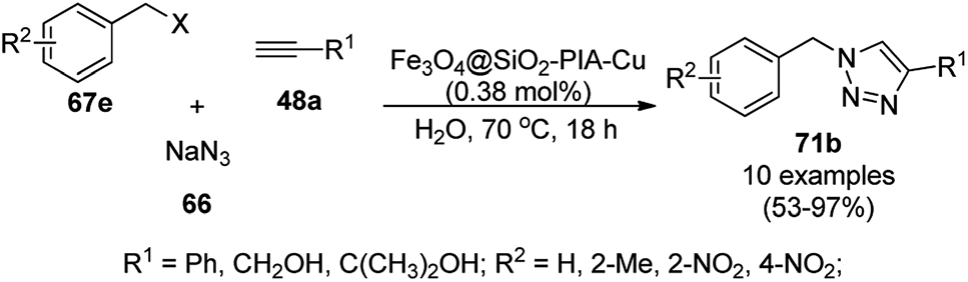
Synthesis of triazole *via* the click reaction among alkyl halides (67e), alkynes (48a) and 66 in the presence of a catalyst such as Cu(ii) complex on Fe_3_O_4_@SiO_2_ NPs.^479^

Following the former approach (Scheme 265), the click reaction of alkynes (48a), alkyl halides (67e), and 66 using Cu(ii) complexes such as Fe_3_O_4_@SiO_2_ NPs as the catalyst was carried out in water as the solvent to yield 1,4-substituted-1*H*-1,2,3-triazoles (71b) in 53–97% yield (Scheme 266).^479^

**Scheme 267:**
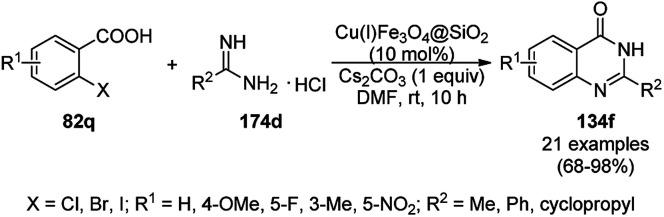
Synthesis of quinazolinones (134f) catalyzed by Cu(i)Fe_3_O_4_@SiO_2_.

The copper(i)-complexed silica-coated magnetite NP- [Cu(i)Fe_3_O_4_@SiO_2_]-catalyzed synthesis of quinazolinones (134f) was achieved *via* the cyclocondensation of *o*-halobenzoic acids (82q) and substituted guanidine hydrochloride (174d) in 68–98% (Scheme 267).^480^ The catalyst was recycled for more than ten runs without loss in its catalytic performance. Silica-coated Fe_3_O_4_ NPs were reacted with [3-(2-aminoethyl)aminopropyl]trimethoxysilane followed by copper(i) salt to obtain Cu(i)Fe_3_O_4_@SiO_2_ NPs. However, the authors did not compare their catalyst with other reported protocols.

**Scheme 268:**
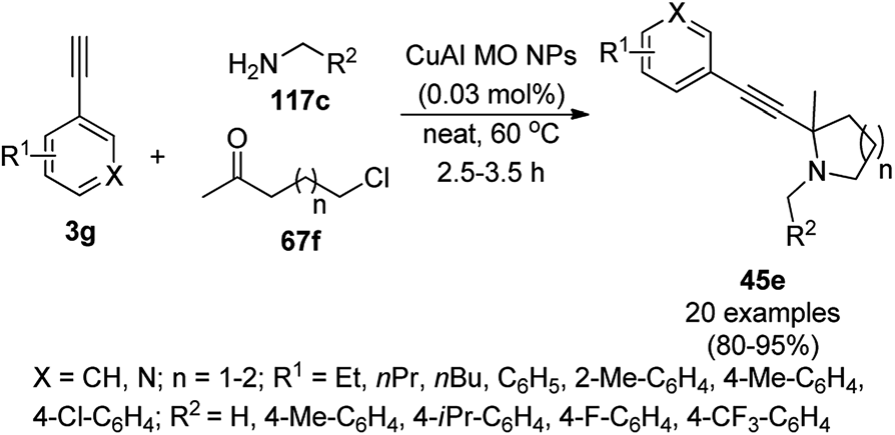
Synthesis of 2-alkynylpyrrolidines/piperidines (45e) reported by Rawat *et al.*

Copper–aluminium mixed oxide nanocomposites (CuAl MO NPs) were reported to be successful for the synthesis of 2-alkynylpyrrolidines/piperidines (45e) from substituted phenyl acetylenes (3g), aliphatic or arylalkyl amines (117c) and methyl ketones (67f, Scheme 268).^481^ CO_2_ gas was bubbled through a co-precipitated aqueous solution of Cu(NO_3_)_2_·6H_2_O and Al(NO_3_)_3_·9H_2_O at pH 8 to obtain copper–aluminium hydrotalcite-like composites, which were calcined to obtain CuAl MO NPs. The obtained NPs were characterized *via* XRD, FESEM, HR-TEM, XPS, and BET analysis. The authors further estimated the parameters for green chemistry metrics such as E-factor, process mass efficiency (PMI), reaction mass efficiency (RME), atom economy (AE), and carbon efficiency (CE), which were found to be very close to ideal values. The hot filtration test revealed that no active catalyst leached into the reaction mixture during the progress of the reaction.

**Scheme 269:**
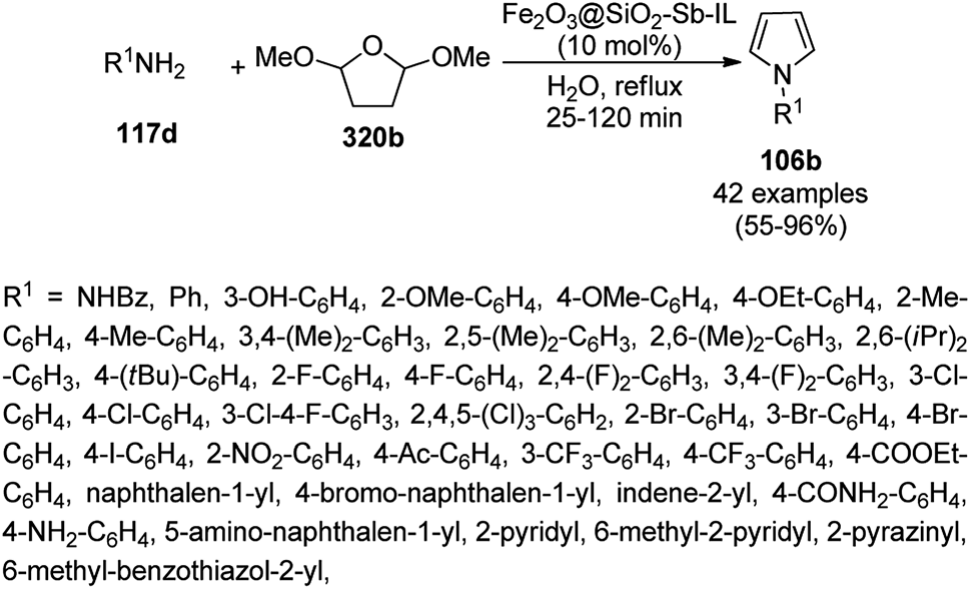
*N*-substituted pyrroles (106b) synthesized in water using nano γ-Fe_2_O_3_@SiO_2_–Sb-IL.

The Clauson-Kaas reaction to synthesize *N*-substituted arylated or heteroarylated pyrroles (106b) was reported by Zhang *et al.* using an MNP-supported antimony nanocatalyst (γ-Fe_2_O_3_@SiO_2_–Sb-IL, 10 mol%) in aqueous medium (Scheme 269).^482^106b was synthesized using tetrahydro-2,5-dimethoxyfuran (320b) and aryl or heteroarylamine (117d) in moderate to excellent yields. The synthesized catalyst was fully characterized *via* EDS, SEM, TEM, FT-IR and XRD. Various conditions were investigated to optimize the reaction, and water was found to be the best solvent under reflux. The activity of the catalyst after the sixth catalytic cycle was found to be identical with the fresh nanocatalyst, as confirmed *via* the TEM images of the catalyst recovered after the sixth catalytic run. The results obtained through inductively coupled plasma mass spectrometry (ICP-MS) revealed that Sb and Fe were not leached from the catalyst during or after the recovery.

**Scheme 270:**
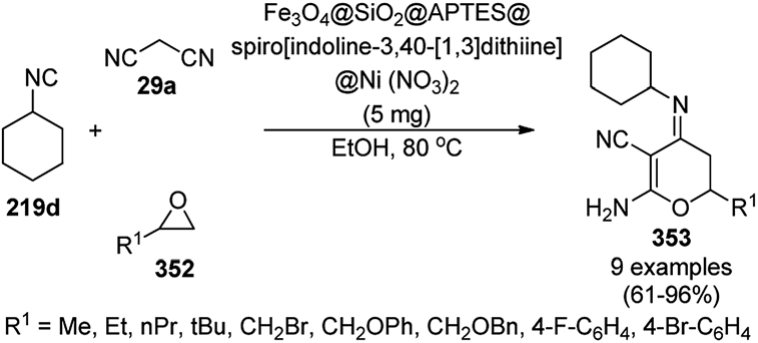
Synthesis of 3,4-dihydro-2*H*-pyrans (353).

Spiro[indoline-3,4′-[1,3]dithiine]@Ni(NO_3_)_2_ grafted on silica-coated magnetite NPs was used as a novel catalyst for the synthesis of 3,4-dihydro-2*H*-pyrans (353) using cyclohexyl isocyanide (219d), malononitrile (29a) and substituted epoxide (352, Scheme 270).^483^ The MNPs synthesized by the treatment of silica-coated magnetite NPs with spiro[indoline-3,4′-[1,3]dithiine] and nickel nitrate were also evaluated for their anti-bacterial and anti-fungal activity, revealing their potential as bioactive MNPs.

**Scheme 271:**
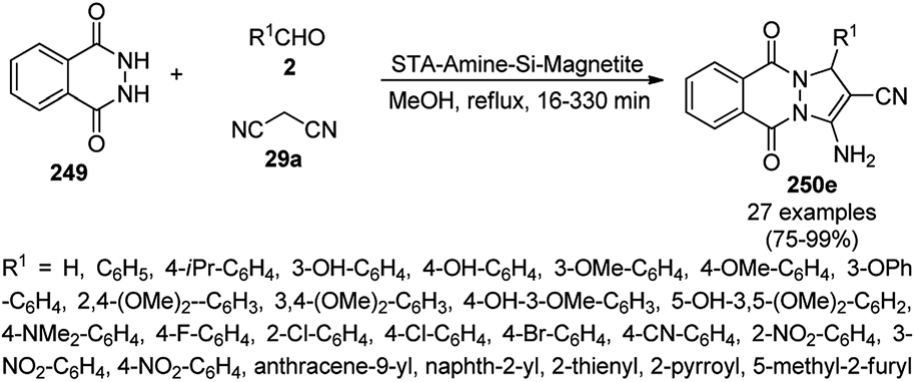
Synthesis of 1*H*-pyrazolo[1,2-*b*]phthalazinediones (250e) reported by Arora *et al.*

Arora *et al.* reported the coated silicotungstic acid (STA, H_4_[W_12_SiO_40_]) on amino-functionalized Si-magnetite nanoparticle-catalyzed synthesis of 1*H*-pyrazolo[1,2-*b*]phthalazinediones (250e) in methanol under reflux using 2,3-dihydrophthalazine-1,4-dione (249), 29a and substituted aryl aldehyde (2, Scheme 271).^484^ The MNPs were synthesized *via* co-precipitation followed by ultrasonication and silica coating using the Stöber method,^485,486^ and the STA-Amin-Si-MNPs were synthesized *via* the wet impregnation method. In the absence of the catalyst, the reaction proceeded with 55% yield and longer reaction time (435 min), which was reduced to a shorter reaction time (45 min) with better 98% yield. The catalyst maintained its activity since the yield of the product after the sixth catalytic run was obtained without significant loss in activity, and authors also claimed the superiority of the developed protocol over the other reported procedures^487–493^ with respect to the higher yield of 1*H*-pyrazolo[1,2-*b*]phthalazinediones, shorter reaction time, greener solvent, and lower catalytic loading and temperature. Recently, following a similar approach, the synthesis of 250e was reported by Bodaghifard *et al.* using a basic IL supported on titania-coated NiFe_2_O_4_ NPs in PEG800 at 90 °C in a shorter period (10–70 min).^494^

**Scheme 272:**
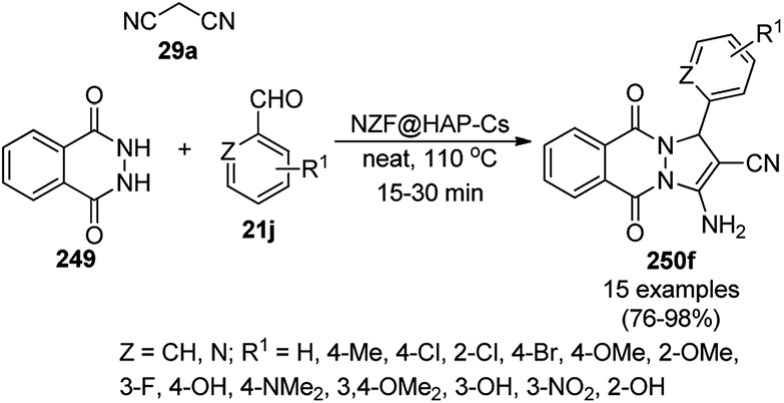
1*H*-pyrazolo[1,2-*b*]phthalazinediones (250f) reported by Maleki *et al.*

Next, Maleki *et al.* reported the similar synthesis of the pyrazolo[1,2-*b*]phthalazinediones (250f) using similar starting materials (249, 29a, and 21j), as shown in Scheme 272, using different Cs_2_CO_3_ catalysts supported on hydroxyapatite-encapsulated Ni_0.5_Zn_0.5_Fe_2_O_4_ NPs under solvent-free conditions at 110 °C.^495^ The Ni_0.5_Zn_0.5_Fe_2_O_4_ NCs were prepared using NiCl_3_, ZnCl_2_, and FeCl_3_, which were further treated with hydroxyapatite and caesium carbonate to obtain the final NPs. The catalysts were characterized *via* FT-IR, SEM, TEM, XRD, and vibrational sample magnetometry. The reusability of the catalysts for the model reaction was demonstrated for up to five catalytic cycles without appreciable loss in catalytic efficiency. The FT-IR spectra of the recovered and fresh NPs revealed that the catalyst maintained its structure, even after five recycles.

**Scheme 273:**
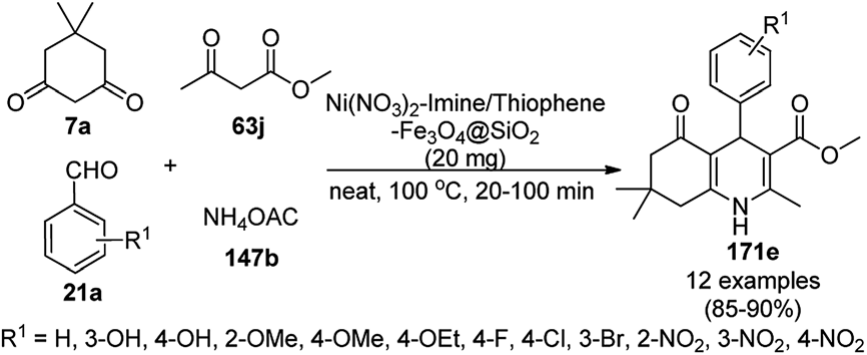
Ni(NO_3_)_2_-imine/thiophene-Fe_3_O_4_@SiO_2_ NP-catalyzed synthesis of polyhydroquinolines (171e).

**Scheme 274:**
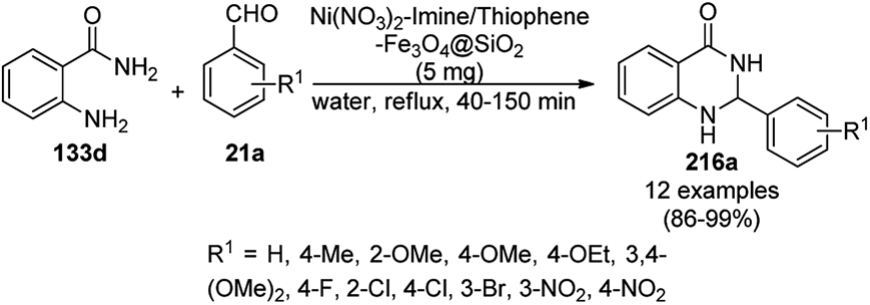
Ni(NO_3_)_2_-imine/thiophene-Fe_3_O_4_@SiO_2_ NP-catalyzed synthesis of 2,3-dihydroquinazolin-4(1*H*)-ones (216a).

Kazemi *et al.* synthesized Ni(NO_3_)_2_-imine/thiophene-Fe_3_O_4_@SiO_2_ NPs and used them as a nanocatalyst in the synthesis of polyhydroquinolines (171e) and 2,3-dihydroquinazolin-4(1*H*)-ones (216a, Scheme 273). The synthesized nanocatalyst was characterized thoroughly *via* FT-IR, SEM, EDX, XRD, vibrating sample magnetometer (VSM) and atomic absorption spectroscopy (AAS). The synthesis of polyhydroquinolines (Scheme 274) was attempted *via* a four-component reaction using 7a, 63j, substituted benzaldehydes (21a) and 147b under solvent-free conditions in 20–100 min at 100 °C in the presence of the nanocatalyst (20 mg for 1 mmol reaction). The synthesis of 2,3-dihydroquinazolin-4(1*H*)-ones (216a) was attempted using water as a green solvent under reflux *via* the ring closure of 133d with 21a using Ni(NO_3_)_2_-imine/thiophene-Fe_3_O_4_@SiO_2_ (5 mg). The developed method was claimed to be superior for the synthesis of polyhydroquinolines and 2,3-dihydroquinazolin-4(1*H*)-ones with higher yields and shorter reaction time in comparison with the reported methods.^385–387^ The final products were purified *via* recrystallisation, avoiding the use of column chromatography. The magnetic NPs can be separated easily and have been demonstrated to have catalytic efficiency even after seven cycles.^496^

**Scheme 275:**
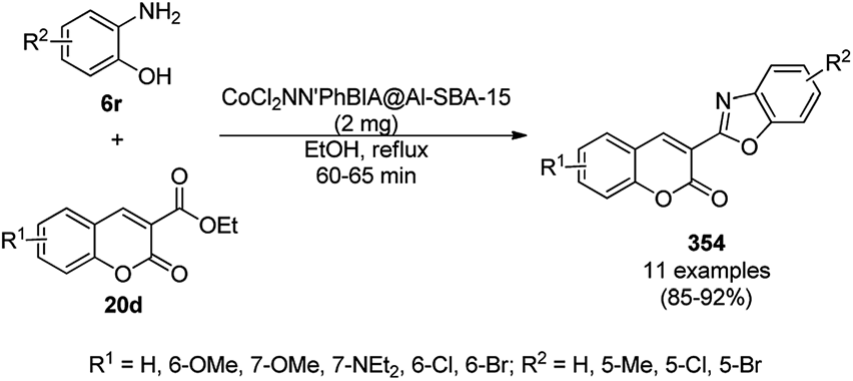
Synthesis of 3-benzoxazol-2-yl-chromen-2-ones (354) assisted by Co–Si NCs.

Safaei-Ghomi *et al.* synthesized 3-benzoxazol-2-yl-chromen-2-ones (354) catalyzed by dichloro *N*,*N*′-(1,2-phenylene)bis(2-aminobenzamide) cobalt(ii)@Al-SBA-15 (CoCl_2_NN′PhBIA@Al-SBA-15) *via* the cyclocondensation of *o*-aminophenol (6r) and coumarin-3-carboxylate (20d) in good to excellent yields (Scheme 275).^497^ The key catalyst was synthesized *via* the treatment of TEOS, aluminium triisopropoxide with a micellar solution of Pluronic P123 to form Al-SBA-15, which was subsequently reacted with CPTMS, *N*,*N*′-(1,2-phenylene)bis(2-aminobenzamide) and cobalt chloride to obtain the final NCs. To catalyze this cyclocondensation, the Co-based NC acted as a Lewis acid *via* the coordination with the carbonyl oxygen of 20d and formed intermediates to form 354. The catalyst was reused eight times with slight loss in its catalytic performance, as demonstrated for the model reaction between ethyl coumarin-3-carboxylate and 2-aminophenol. Previously, they also reported SnO NPs for the synthesis of chromeno[2,3-*b*]pyridines by condensation of malononitrile, aryl or arylalkyl thiols and benzaldehydes.^498^

**Scheme 276:**
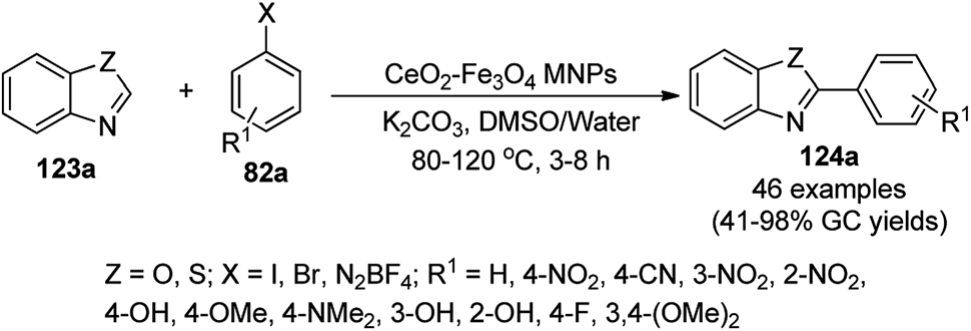
CeO_2_–Fe_3_O_4_ MNP-catalyzed C–H activation of benzoxazole and benzothiazoles (124a).

Nagarkar *et al.* reported the ligand-free direct C–H activation of benzo[*d*]oxazoles and benzo[*d*]thiazoles (123a) catalyzed by nano CeO_2_–Fe_3_O_4_ MNPs using potassium carbonate (K_2_CO_3_) as the base at 80–120 °C using CeO_2_–Fe_3_O_4_ MNPs for the synthesis of 2-aryl benzo[*d*]oxazole and 2-arylbenzo[*d*]thiazole (124a, Scheme 276).^499^ The MNPs were synthesized *via* the wet impregnation method and fully characterized *via* SEM, TEM, EDAX, XRD, FT-IR, DSC-TGA and ICP-MS analysis. The catalyst was recovered up to ten catalytic run without loss in its catalytic activity and stability. The decline in GC yields of 2-phenyl benzo[*d*]oxazole after the removal of the MNPs by magnetic separation after one hour of the reaction between benzo[*d*]oxazole and iodobenzene clearly revealed that the reaction could not proceed without catalytic assistance. The ICP-MS analysis also revealed that no leaching of cerium occurred.

**Scheme 277:**
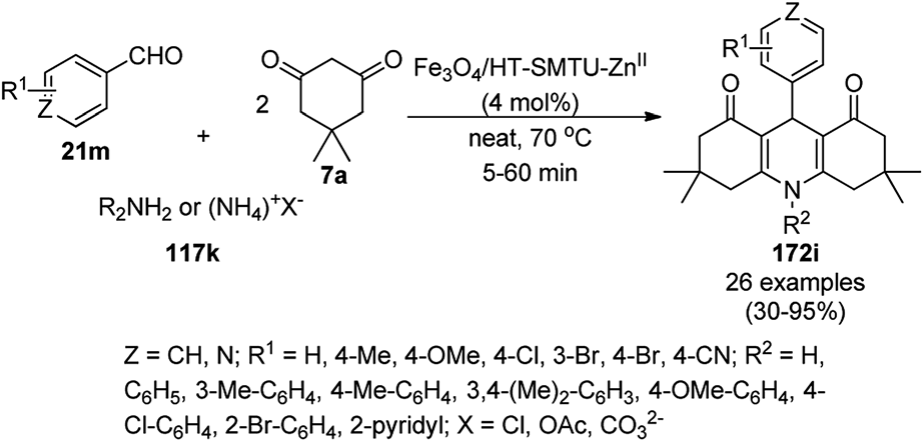
Synthesis of acridinediones (172i) catalyzed by bifunctional nanocatalysts.

The interesting synthesis of acridinediones (172i) was observed under the catalytic effect of zinc doped and supported on magnetic hydrotalcite (Fe_3_O_4_/HT-SMTU-Zn^II^) using aldehydes (21m), dimedone (7a) and arylamine (117k) or ammonium salts (Scheme 277).^500^ The recycling of the catalyst was studied for up to six catalytic reuses, giving 85–95% yield of 172i (Z= CH, R^1^ = H, and R^2^ = H).^500^ The hot filtration test and poisoning test by ethylenediamine tetraacetic acid (EDTA) revealed that no leaching of the heterogeneous catalyst occurred during the progress of the reaction. The developed protocol was also claimed to be green and efficient since it is operative at a relatively low temperature,^501^ free from toxic solvents,^398^ in a short time,^502,503^ avoiding tedious purification^504–506^ for the synthesis of 172i.

**Scheme 278:**
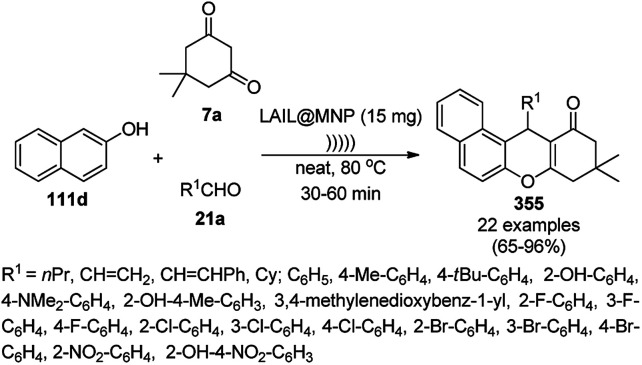
LAIL@MNP- catalyzed three-component and one-pot synthesis of benzoxanthenes (355).

**Scheme 279:**
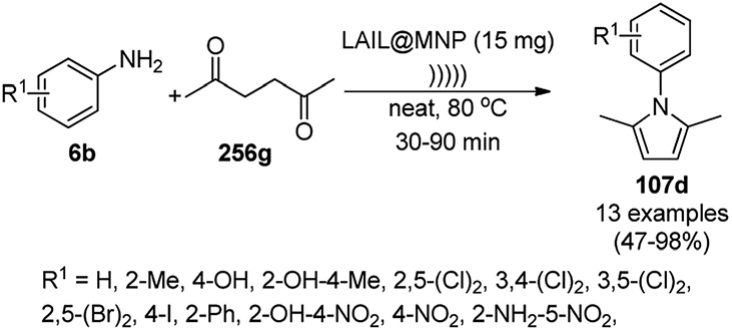
Paal–Knorr reaction catalyzed by LAIL@MNP.

Tran *et al.* reported the catalytic use of Lewis acid-based ILs supported on Fe_3_O_4_ NPs LAIL@MNP for the synthesis of benzoxanthenes (355) and *N*-aryl pyrroles (107d) *via* multicomponent reactions (Scheme 278) and Paal–Knorr reaction (Scheme 279), respectively.^507^ They synthesized NPs *via* the loading of 3-(3-(trimethoxysilyl)propyl)-1*H*-imidazol-3-ium chlorozincate(ii) IL on magnetite NPs. The durability of the catalyst was demonstrated for up to five re-runs.

**Scheme 280:**
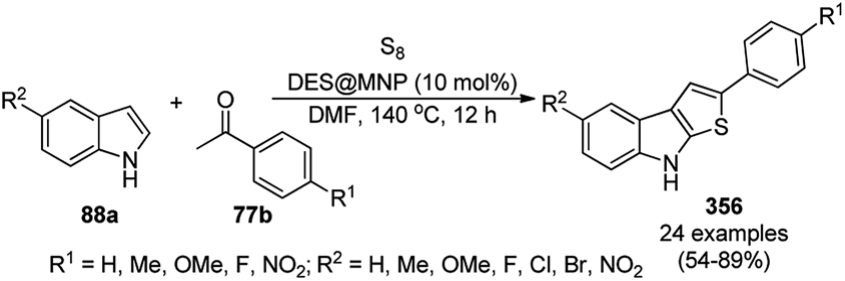
Synthesis of 2-phenyl-8*H*-thieno[2,3-*b*]indoles (356) using DES@MNP.

Further, Tran *et al.* also reported the synthesis of 2-phenyl-8*H*-thieno[2,3-*b*]indoles (356) using 5-substituted indole (88a), sulfur, and 4-substituted acetophenone (77b) in DMF (Scheme 280).^508^ The above one-pot synthesis was achieved using recyclable silica-coated magnetite NPs supported on a deep eutectic solvent (DES@MNP), where it acted as a Lewis acid catalyst *via* the Willgerodt–Kindler reaction to form thirenium intermediate, which reacted with 88a to form 356. The loading of the silica-coated Fe_3_O_4_ NPs with urea and ZnCl_2_-derived deep eutectic solvent led to the formation of DES@MNP. However, they used a high equivalent of sulfur (8 equiv.), which is the drawback of this methodology.

**Scheme 281:**
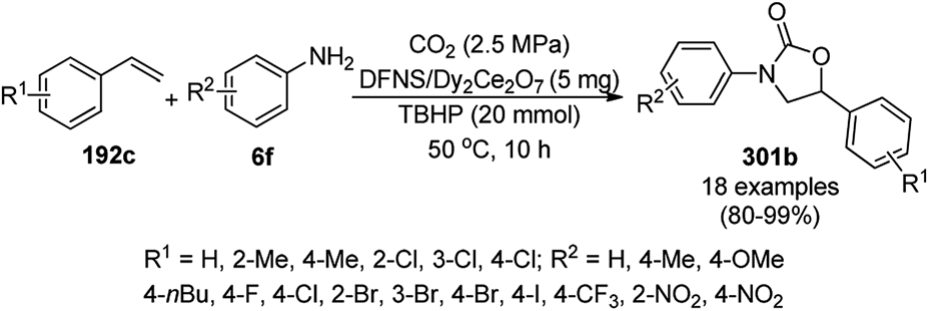
Synthesis of 3-phenyl-2-oxazolidinones from styrenes (192c), anilines (6f) and gaseous carbon dioxide.

The synthesis of 2-oxazolidinones (301b) was achieved *via* cycloaddition of carbon dioxide catalyzed by dendritic fibrous nanosilica (DFNS)-based NC (DFNS/Dy_2_Ce_2_O_7_)-substituted styrene (192c) and aniline (6f) using *tert*-butyl hydroperoxide (TBHP) as oxidizing agents at 50 °C (Scheme 281).^509^ The silanol bonds of DFNS were decorated first with 3-APTES followed by treatment with the gum of *Ferula assa-foetida* of dysprosium nitrate [Dy(NO_3_)_3_] and ceric ammonium nitrate [(NH_4_)_2_Ce(NO_3_)_6_], leading to the green synthesis of an NC. The catalyst was found to be very effective after recycling for more than nine times and hot filtration tests. We were surprised to find that for the synthesis of 301b with 1 mmol of 6f, 10 mmol of 192c, and 20 mmol of oxidant were used with a bimetallic NC, leading to poor atom economy and waste generation.

**Scheme 282:**
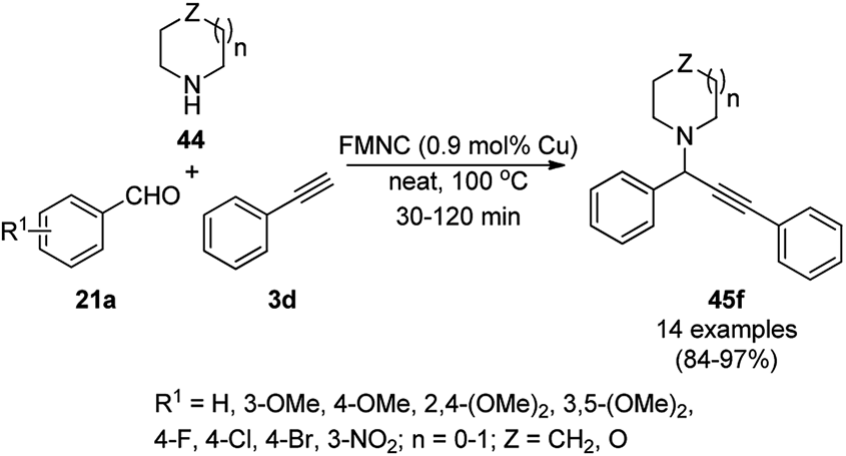
Cu-catalyzed A^3^ coupling for the synthesis of propargylamines (45f).

Super-paramagnetic MCM-41 with a dendrite copper complex (FMNC) catalyzed A^3^ coupling for the synthesis of propargylamines (45f) from substituted benzaldehydes (21a), alicyclic amines (44), and phenyl acetylene (3d, Scheme 282) was reported by Abdollahi-Alibeik *et al.*^510^ FMNC was synthesized *via* the functionalization of MCM-41 including amination with diethyl amine and formation of the complex using copper acetate. The catalyst was separated by magnetic decantation and recycled for up to three runs with slight loss in its catalytic performance during a prolonged reaction time together with a slight loss in Cu content. However, the protocol reported by Abdollahi-Alibeik *et al.* can be considered superior considering its shorter reaction time, lower catalytic loading, rapid synthesis, isolated yields and slight loss in yields upon recycling compared to reported catalysts, namely Au(iii) supported on poly ILs coated on magnetic nanoparticles (MNP@PILAu),^511^ Cu-MCM-41,^141^ silver–graphene nano-composite (Ag-G),^55^ and CuNPs supported on micro-starch (CuNPs@MS).^138^

**Scheme 283:**
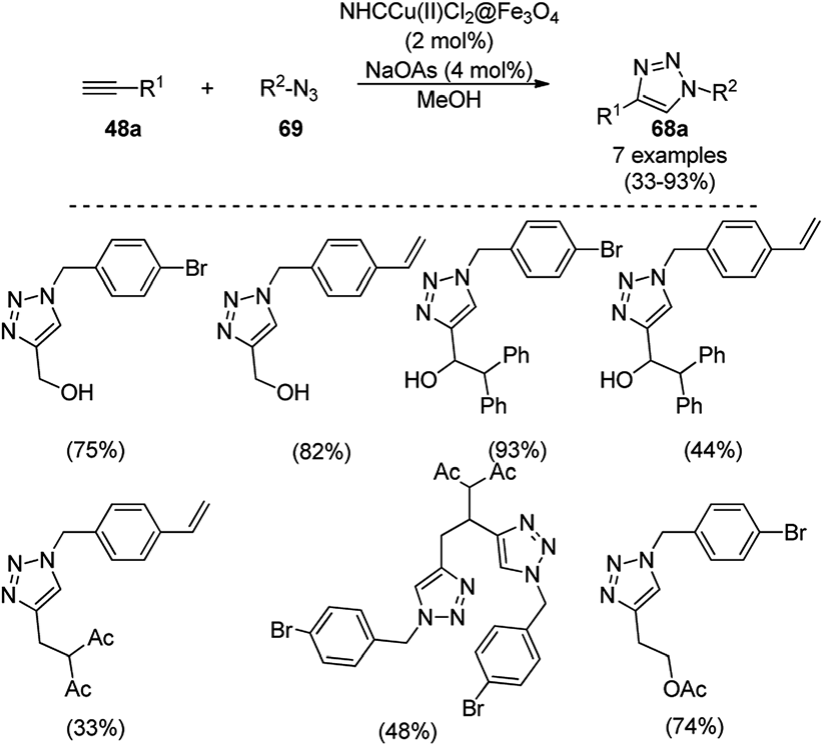
CuAAC reactions of alkynes (48a) and azides (69).

The copper-catalyzed azide–alkyne cycloaddition (CuAAC) for the synthesis of triazoles (68a) was achieved using NHC-copper complexes supported on magnetic NPs (Scheme 283) using alkyne (48a) and azide (69) in methanol with sodium ascorbate (NaOAs) as a reducing agent.^512^ Magnetic NPs synthesized using FeCl_2_·4H_2_O and FeCl_3_·6H_2_O were treated with APTMS and triethyl orthoformate to obtain a formamidinium intermediate. The latter was reacted with 2,6-bis(diphenylmethyl)-4-methylaniline to form NHC, which was complexed with CuCl_2_ to obtain the final MNPs *via* magnetic separation. However, the catalyst did not exhibit suitable yields during its reuses for up to three runs (53–80%) due to its deactivation.

## 4 Conclusions and future directions

Historically, catalysis, a fascinating topic, has been always investigated on a trial and error basis. The use of catalysts in a reaction system not only helps to achieve synthetic transformations, but also to reduces the quantity of substrates or reagents, and the cost of chemical conversions. The recent advancements in material chemistry has enabled a new era in the design of sophisticated nanocatalysts.

The synthesis of heterocycles containing nitrogen^513^ and oxygen has increased exponentially in the scientific community in search of novel molecules or scaffolds of biological importance. In the last decade, metal NPs have been reported for the catalysis of a huge number of organic transformations to construct C–C, C–X, and C–N bonds for the synthesis of small to complex or macromolecules. For the synthesis of heterocyclic scaffolds, knowledge of synthetic organic chemistry acts as a toolbox for chemists. However, the major concern for challenging synthetic tasks is the construction of bonds between two dissimilar starting materials, where the high energy of the transition state is the restricting factor to achieve the successful conversion. Nanocatalysts play a vital role in this context by increasing the surface area of active catalysts, bringing electrophiles and nucleophiles in closer proximity, reducing the energy gap between the substrate and product, providing the suitable hydrogen bonding interactions, holding the reactants together with dual ionic or hydrogen bonding interaction, *etc.* Besides individual MNPs, encapsulated MNPs have also been recognized for their potential in prominent catalysis.^514^ Due to the advancement of sophisticated technology, the complete characterization of NPs can be achieved with several techniques to confirm their integrity and nanoparticulate behavior during a reaction. NPs have several advantages such as low catalytic loading, high reactivity, high selectivity, complete recyclability, sufficient mechanical stability and green applicability, making them desirable and ideal catalytic systems. The recyclability of catalysts is a significant issue in the pharmaceutical industry, where the cost of reagents and catalysts is a key factor for pilot-scale reactions. Accordingly, use of nanocatalysts represents a suitable protocol for the pilot-scale synthesis of targets since NCs can be recycled several times without loss in their chemical integrity, nanoparticulate behavior, yield of product and turnover frequency.

In the present review, the ever-growing broad applications of several MNPs have been reflected for the synthesis of five-, six- and seven-membered monocyclic, bicyclic, tricyclic and tetracyclic heteroaromatic or heteroaliphatic scaffolds. AuNPs are generally used for the synthesis of substituted quinolines and quinoxalines; CuNPs and or FeNPs for triazoles and 2,3-dihydroquinazolin-4(1*H*)-ones; PdNPs for dehalogenation, and RuNPs for deuteration and ring-closing metathesis. For hydrogenation and dehydrogenation, PdNPs, PtNPs, and RhNPs are highly utilized. The successful *N*-arylation of nitrogen-containing heterocycles can be achieved with the help of CuNPs and or FeNPs. The recent trend and strategy of combining two or more metallic NPs in a common catalytic system has emerged as an innovative and constructive way to tackle challenging synthetic transformations. The ever-expanding field of material chemistry has led to significant advancements in the design of catalysts and their synthetic applications in achieving organic transformations.

## List of abbreviations

AAS: Atomic absorption spectroscopy
AES: Atomic emission spectrometry
AFM: Atomic force microscopy
AgNPs: Silver nanoparticles
ANDSA: 7-Aminonaphthalene-1,3-disulfonic acid
APTMS: (3-Aminopropyl)triethoxysilane
AuNPs: Gold nanoparticles
BET: Brunauer, Emmett and Teller
BTU: Benzoylthiourea
CNTs: Carbon nanotubes
CoNPs: Cobalt nanoparticles
CPTMS: 3-Chloropropyltrimethoxysilane
CPTES: 3-Chloropropyltriethoxysilane
CTAB: Cetyltrimethylammonium bromide
DABCO: 1,4-Diazabicyclo[2.2.2]octane
DETA: Diethylenetriamine
DLS: Dynamic light scattering
DMAB: Dimethylamine borane
DRIFTS: Diffuse reflectance infrared Fourier transform spectroscopy
EDX: Energy-dispersive X-ray microanalysis
EG: Ethylene glycol
EGFR: Epidermal growth factor receptor
EPR: Electron paramagnetic resonance
FE-SEM: Field emission scanning electron microscopy
FT-IR: Fourier-transform infrared spectroscopy
GC-MS: Gas chromatography-mass spectroscopy
GO: Graphene oxide
HAADF-STEM: High-angle annular dark-field scanning transmission electron microscopy
HB: Hydrogen bond
ICP-MS: Inductively coupled plasma-mass spectrometry
IL: Ionic liquid
MCR: Multicomponent reaction
MNPs: Metal nanoparticles
[MSPP]HSO_4_: 4-Methyl-1-(3-sulfopropyl)pyridinium hydrogen sulfate
NCs: Nanocatalysts
NHCs: N-Heterocyclic carbenes
NMR: Nuclear magnetic resonance
NPs: Nanoparticles
PdNPs: Palladium nanoparticles
PEI: Poly(ethylenimine)
PsIL: Polymeric ionic liquids
PVP: Polyvinyl pyrrolidone
PXRD: Powder X-ray diffraction
QLES: Quasi-electron light scattering
RGO: Reduced graphene oxide
RuNCs: Ruthenium nanocatalysts
SAED: Selected area electron diffraction
SEM: Scanning electron microscopy
SMNOP-1: Diatomite-supported manganese oxide (Mn_3_O_4_) nanoparticles
SMNP: Silica coated magnetic nanoparticles
SiNPs: Silica nanoparticles
TBAF: Tetrabutylammonium fluoride
TEM: Transmission electron microscopy
TOF: Turnover frequency
TON: Turnover number
TPPTS: Tris(3-sulfophenyl)phosphine trisodium salt
USFDA: United States Food and Drug Association
UV-Vis: Ultraviolet visible spectroscopy
VSM: Vibrating sample magnetometer
XANES: X-ray absorption near-edge structure
XPS: X-ray photoelectron spectroscopy
XRD: X-ray diffraction

## Conflicts of interest

There are no conflicts of interest to declare.

## Supplementary Material
